# Coupled Rotary and Oscillatory Motion in a Second-Generation Molecular Motor Pd Complex

**DOI:** 10.1021/jacs.2c08267

**Published:** 2023-01-05

**Authors:** Lukas Pfeifer, Charlotte N. Stindt, Ben L. Feringa

**Affiliations:** † Stratingh Institute for Chemistry, University of Groningen, Nijenborgh 4, 9747 AG Groningen, The Netherlands; ‡ Zernike Institute for Advanced Materials, University of Groningen, Nijenborgh 4, 9747 AG Groningen, The Netherlands

## Abstract

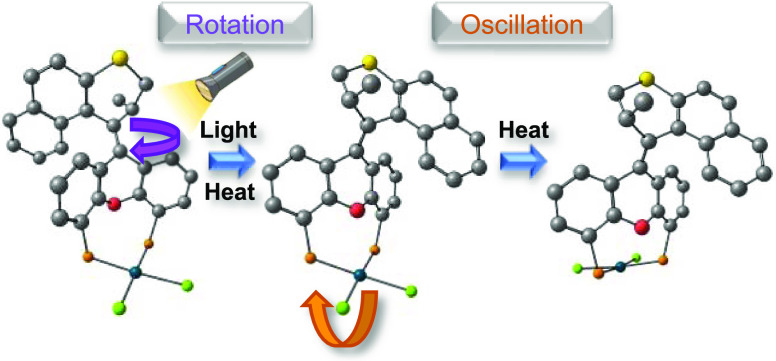

Molecular machines offer many opportunities for the development of responsive materials and introduce autonomous motion in molecular systems. While basic molecular switches and motors carry out one type of motion upon being exposed to an external stimulus, the development of molecular systems capable of performing coupled motions is essential for the development of more advanced molecular machinery. Overcrowded alkene-based rotary molecular motors are an ideal basis for the design of such systems as they undergo a controlled rotation initiated by light allowing for excellent spatio-temporal precision. Here, we present an example of a Pd complex of a second-generation rotary motor whose Pd center undergoes a coupled oscillatory motion relative to the motor core upon rotation of the motor. We have studied this phenomenon by UV–vis, NMR, and density functional theory calculations to support our conclusions. With this demonstration of a coupled rotation–oscillation motion powered by a light-driven molecular motor, we provide a solid basis for the development of more advanced molecular machines integrating different types of motion in their operation.

## Introduction

**Figure 1:**
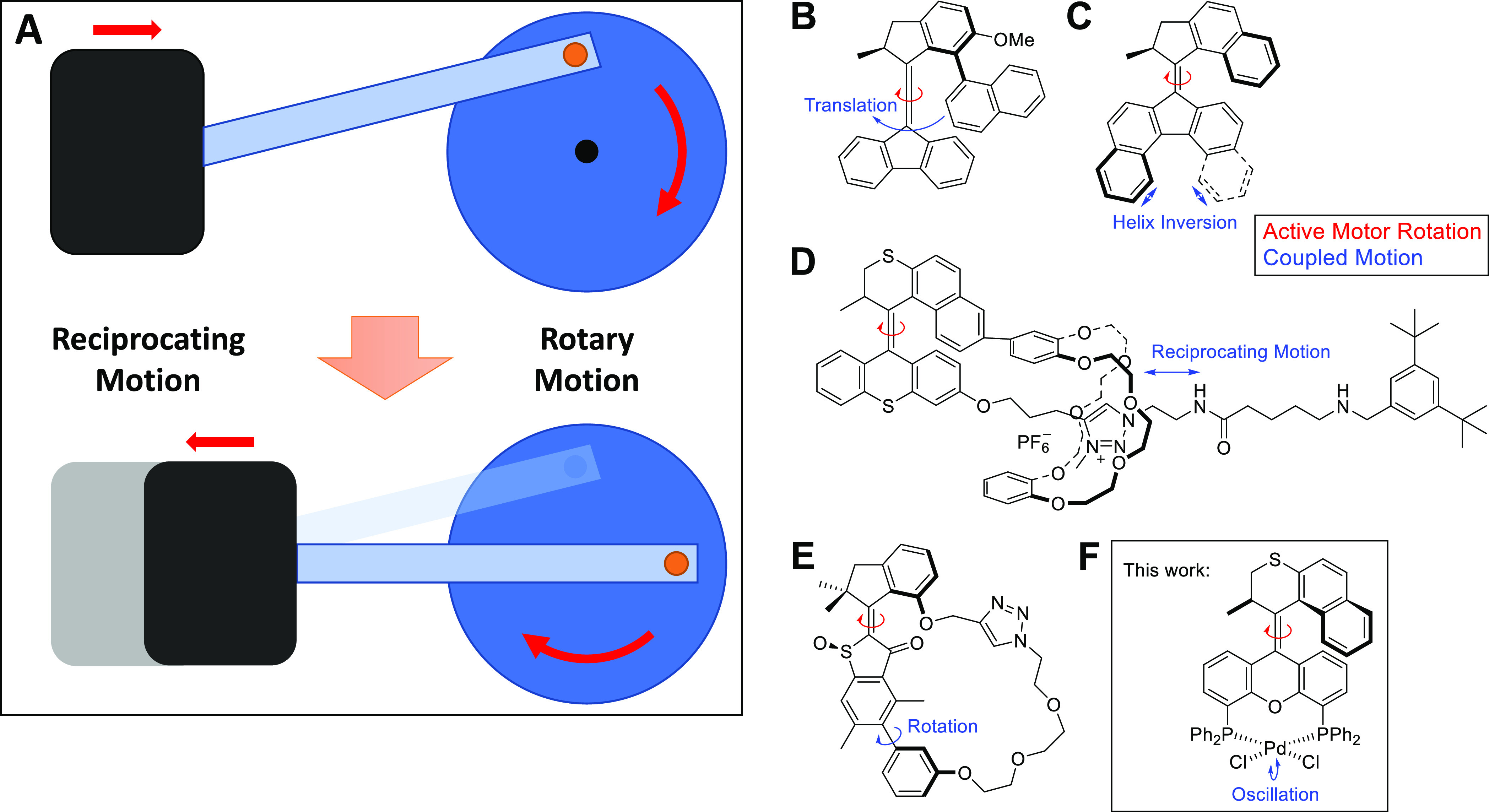
(A) Principle of coupled reciprocating and rotary motion. (B–E) Known examples of Feringa-type molecular motors whose actively controlled rotation is coupled to a secondary spontaneous motion. (F) Coupled rotation and oscillation in a second-generation rotary molecular motor presented in this work.

Molecular motors are nanoscale tools that allow the conversion of an energy input (e.g., chemical, light) into controlled, directional motion at the nanoscale.^1−7^ They are therefore envisioned to play an analogous role to macroscopic engines with respect to the development of more complex nanomachines,^8−11^ perhaps inspired by biological examples such as adenosine triphosphatase (ATPase)-mediated transport^12,13^ and the bacterial flagellar motor.^14^ As is the case in the macroscopic world to achieve this goal, it will be necessary to develop strategies for coupling the motors’ primary motion to secondary motions relaying the movement to other parts of the assembly. These junctions furthermore provide the possibility to interconvert different types of motion. For example, a crankshaft converts the reciprocating motion of an engine’s pistons into the final rotary motion output, which is suitable for powering vehicles (Figure 1A).

Molecular systems displaying coupled rotary motion, like cogwheels, turnstiles, and bevel-gear systems, based on molecular rotors have been reported^15^ without actively driving their rotation or controlling directionality, setting them apart from molecular motors.^8^ An early system showing coupling between the active rotation of a second-generation artificial rotary molecular motor^16^ and the passive rotation of a covalently attached biaryl rotor was disclosed in 2005.^17^ In this case, the molecular motor acted as a switch controlling the speed of rotation of the biaryl rotor, although without actively driving its rotation.

In 2017, our group demonstrated coupled rotary and translational motion in a second-generation motor with a pendant naphthyl substituent (Figure 1B).^18^ Upon rotation around the central alkene double bond, this substituent was shown to describe a circular translational movement around the motor’s lower half, always facing the motor core with the same side. A study on a different second-generation motor featuring an extended aromatic core revealed a helix inversion coupled to its rotation as a byproduct of the steric clash in the newly introduced extended lower half (Figure 1C).^19^ Light-driven mechanical threading of a flexible tetraethylene glycol chain through a macrocycle was realized in a hemithioindigo-based molecular motor.^20^ Similarly, coupling between rotary and reciprocating motion on the nanoscale was demonstrated using a [1]rotaxane whose two components (macrocycle and shaft) were connected to the two halves of a molecular motor (Figure 1D),^21^ mimicking a reverse reciprocating engine, powered not by the reciprocating but by the rotary motion. An earlier example used a stiff-stilbene^22^ switch for driving a rotaxane’s translation. Coupling between an actively controlled and a spontaneous rotary motion was described by Dube and co-workers by demonstrating active and unidirectional acceleration of biaryl rotation using a molecular motor (Figure 1E).^23^ The same group recently presented a molecular gearing system enabling the translation of a light-driven 180° rotation into a 120° rotation around an axis at a 120° angle of that of the primary rotation.^24^ Finally, our “nanocar”, capable of directional linear motion on a Cu(111) surface powered by the concerted action of four molecular motor units^25^, is an example of correlated rotary and linear translational motion.

Actively controlled rotary motion of an artificial molecular motor has therefore been coupled to passive rotary,^23,24^ reciprocating,^19,21^ and translational motion,^18,25^ comprising three of the four regular, fundamental types of motion, not taking irregular motion into consideration. In this study, we are presenting the missing link of spontaneous oscillation to active rotary motion using a PdCl_2_ complex of a novel second-generation diphosphine motor.

Specifically, we present a PdCl_2_ complex of a XantPhos-derived motor where the actively controlled rotary motion of the motor backbone is coupled to a spontaneous oscillating motion carried out by the PdCl_2_ group (Figure 1F). Furthermore, the effects of Pd-coordination on the spectral as well as rotary properties of this motor ligand are discussed. The motor compounds have been studied by NMR and UV–vis spectroscopies, mass spectrometry, and single-crystal X-ray diffraction, and experimental results are backed up by density functional theory (DFT) calculations.

## Results and Discussion

### Design and Synthesis of MotorPhos and [(**MotorPhos**)PdCl_2_]

For the design of our novel motorized ligand, **MotorPhos**, we took XantPhos, a proven good ligand for Pd(II), as inspiration since it is based on xanthene, which is a common lower half in second-generation molecular motors. The two methyl groups in the 9-position of XantPhos were replaced by a six-membered ring upper half containing a sulfur atom for added steric hindrance. This introduces an sp^2^ center in the 9-position, which is expected to lead to some minor changes in the structure of the motor’s lower half but should not change the coordination behavior of the phosphine groups. Upon complexation with PdCl_2_, we would, therefore, obtain a complex [(**MotorPhos**_**s**_)PdCl_2_] where the PdCl_2_ moiety assumes an approximately 90° angle with respect to the motor backbone, as is the case in [(XantPhos)PdCl_2_]. This is a prerequisite for the desired coupled motion, as this orientation allows maximum sensitivity to changes in the upper half upon isomerization, which we anticipated should promote the latter to swing to the other side of the motor backbone and induce oscillation in the lower half.

**Figure 2:**
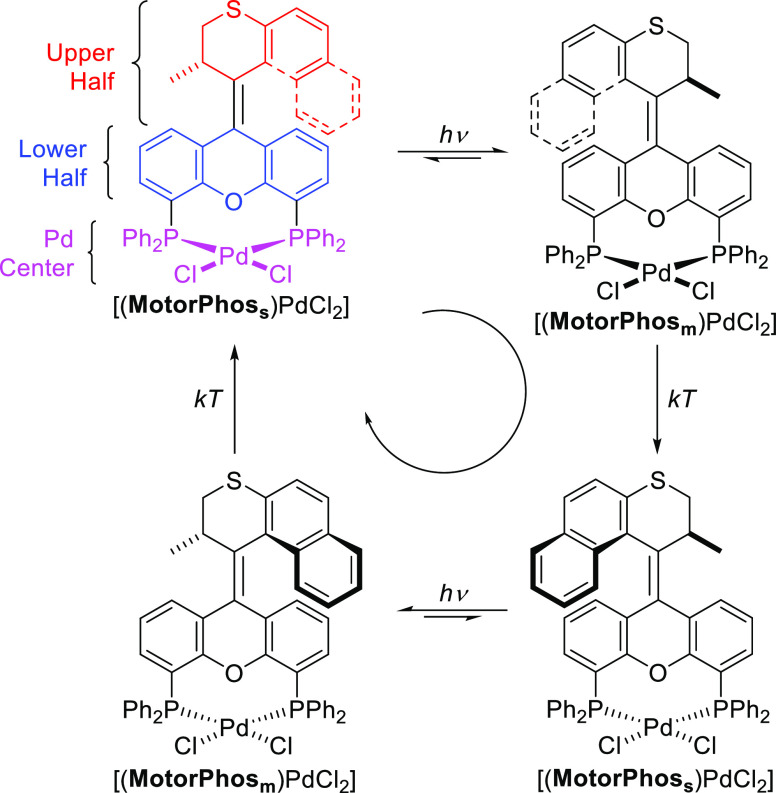
Schematic representation of a 360° rotation of the upper half of [(**MotorPhos**)PdCl_2_] with respect to the lower half accompanied by a back-and-forth oscillation of the PdCl_2_ moiety.

The envisioned mode of operation including the desired coupled motion of [(**MotorPhos**)PdCl_2_] is shown in Figure 2.

Starting from a stable isomer [(**MotorPhos**_**s**_)PdCl_2_], photochemical *E*/*Z* isomerization leads to the formation of a metastable isomer [(**MotorPhos**_**m**_)PdCl_2_], as is commonly observed for Feringa-type molecular motors. In a second, thermal step, the so-called thermal helix inversion (THI), this metastable isomer forms a second stable isomer. During this thermal step, the PdCl_2_ is expected to swing to the other side of the motor backbone describing an oscillatory motion, which is powered by and, therefore, coupled to the rotation of the motor. These steps and the corresponding motions are repeated in the next half of the 360° rotary cycle.

**Scheme 1:**
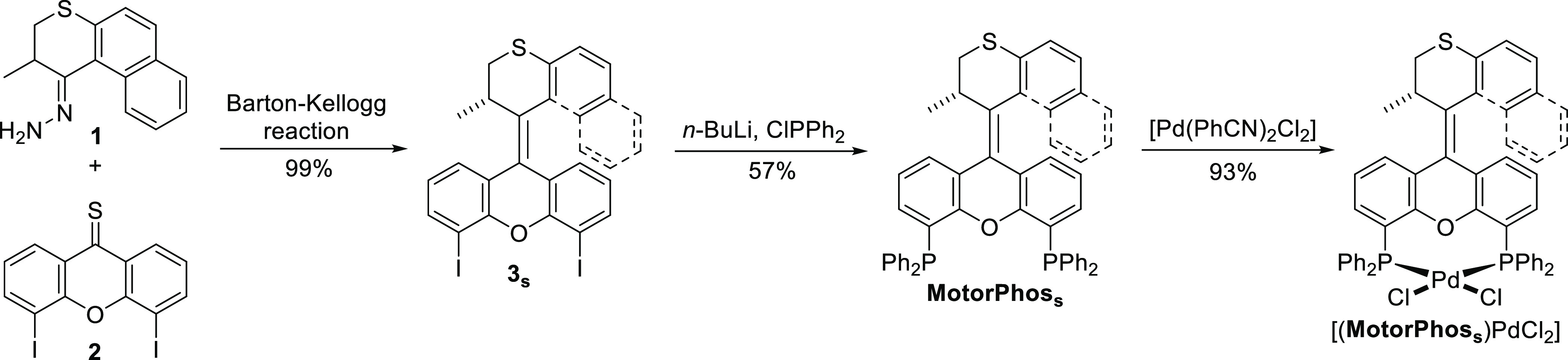
Synthesis of **MotorPhos**_**s**_ and Its PdCl_2_ Complex [(**MotorPhos**_**s**_)PdCl_2_]

The diphosphine motor **MotorPhos** was prepared in five linear steps from commercially available materials with an overall yield of 23%. The key step was Barton–Kellogg olefination using hydrazone **1** and thioketone **2** (Scheme 1), which was followed by double Li–I exchange and quenching with ClPPh_2_ to give the stable isomer of the desired free diphosphine motor, **MotorPhos**_**s**_. Finally, [(**MotorPhos**_**s**_)PdCl_2_] was prepared via double ligand exchange on [Pd(PhCN)_2_Cl_2_].

[(**MotorPhos**_**s**_)PdCl_2_] was obtained as an orange solid indicating that the rotation of this motor could potentially be driven using visible light (vide infra). The design of molecular rotary motors, which can be driven using visible and near-infrared light, has also attracted intense interest recently, as this is a requirement for many potential applications of molecular machines, especially in biological settings.^19,26−33^

### Solid-State Structure

**Figure 3:**
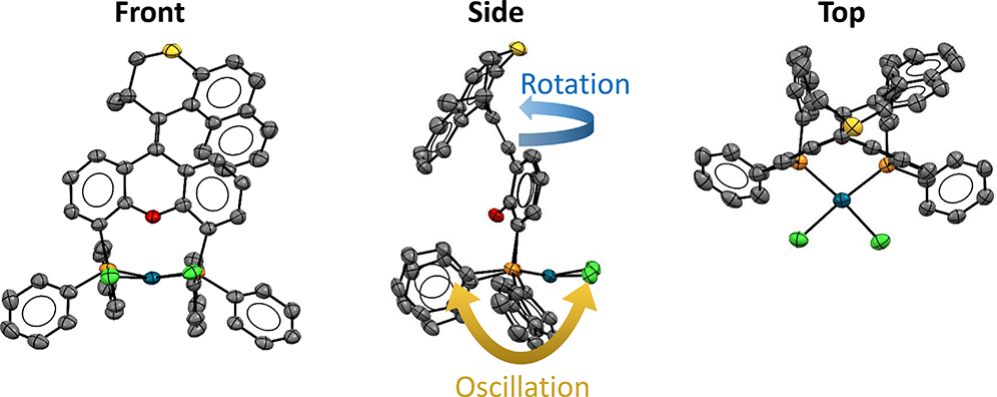
Front, side, and top view of an ORTEP diagram (50% probability, hydrogens omitted for clarity) of polymorph 2 of [(**MotorPhos**_**s**_)PdCl_2_] as determined by single-crystal X-ray diffraction.

For the envisioned oscillatory motion (Figure 2) of the PdCl_2_ unit to be feasible, it needs to form an angle >0° with the xanthene stator half of the motor, but ideally it would approach 90° to ensure sufficient interactions with the upper half upon motor rotation. To determine the relative orientation of the PdCl_2_ unit in the stable isomer of [(**MotorPhos**)PdCl_2_], single crystals of [(**MotorPhos**_**s**_)PdCl_2_] were grown by slow diffusion of pentane into a saturated DCM solution at room temperature and analyzed by X-ray diffraction. Under these conditions, [(**MotorPhos**_**s**_)PdCl_2_] was found to crystallize in two different polymorphs with space groups *P*-1 (polymorph 1) and *P*21/*c* (polymorph 2), respectively. The conformation of [(**MotorPhos**_**s**_)PdCl_2_] was found to be highly preserved in both polymorphs with the main difference being the presence of two DCM molecules in the asymmetric unit of polymorph 1, which were absent in polymorph 2. Three representative views of the asymmetric unit of polymorph 2 are shown in Figure 3 (see also Figure S1).

The angle between a line connecting the central C and O atoms of the xanthene moiety and the plane formed by the Pd center and its four neighboring atoms is 79.67(9) and 80.97(13)° in polymorphs 1 and 2, respectively. These values are similar to the published solid-state structure of [(XantPhos)PdCl_2_] with an angle of 79.6(3)°.^34^ They are also large enough to anticipate sufficient interactions with the upper half upon formation of the second stable isomer following a 180° rotation. This should cause the envisioned oscillatory motion, where the PdCl_2_ swings to the other side of the motor, to take place (Figure 2).

No interaction between O and Pd could be inferred from the solid-state structures, which would have potentially hindered the movement of the PdCl_2_ unit upon motor rotation. Two of the Ph rings, one from each phosphine, engage in π-stacking, which helps to stabilize the described conformation of this Pd complex with the remaining two Ph rings forming a pocket for the PdCl_2_ unit to sit in. The Pd center forms an almost perfect square planar arrangement with its four substituents, whereby the P–Pd–P angle was found to be the largest at 100.73(3)° (polymorph 1) and 100.67(5)° (polymorph 2) compared to values ranging from 83.27 to 88.00° (polymorph 1) and from 83.84 to 88.70° (polymorph 2) between the other neighboring ligand pairs. The P–Pd–P angle represents the diphosphine bite angle, and it is only marginally smaller than the one found in [(XantPhos)PdCl_2_] (101.18(10)°). In all three structures, the sum of the four angles is ∼360°. In both solid-state structures of [(**MotorPhos**_**s**_)PdCl_2_], the Pd atom sits slightly outside of the plane formed by its four neighboring atoms (polymorph 1: 0.1919(3) Å; polymorph 2: 0.2143(4) Å). The folding angle of the lower half is 49.23(17) and 48.4(2)° for polymorphs 1 and 2, respectively. This is significantly different from [(XantPhos)PdCl_2_] (38.5(5)°) and is due to the sp^2^ hybridization of the C atom connecting the two motor halves compared to the corresponding sp^3^ center in XantPhos as well as the presence of the naphthalene moiety sterically clashing with the xanthene half.

### Experimental Evaluation of Coupled Motion in [(**MotorPhos**)PdCl_2_]

**Figure 4:**
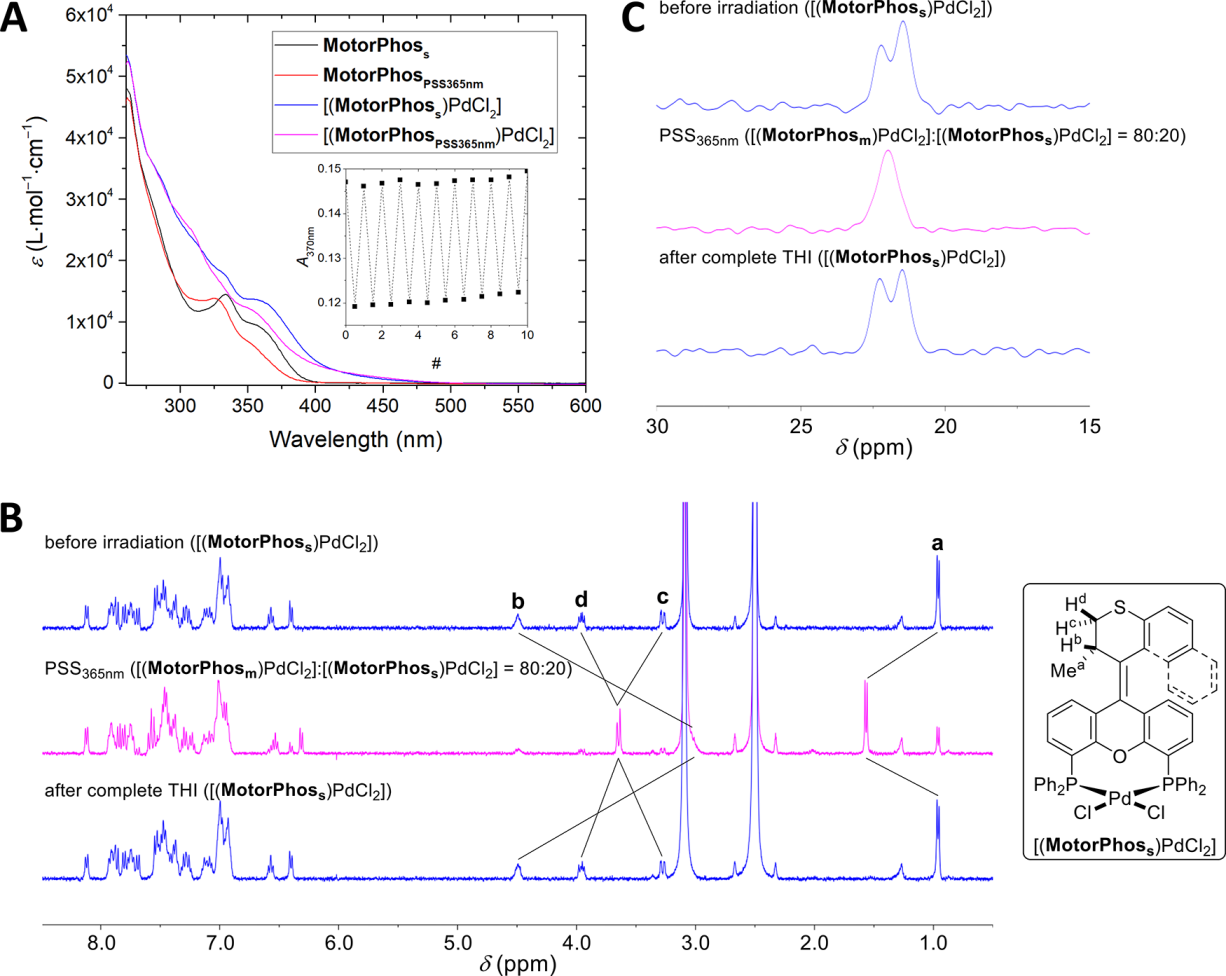
(A) Absorption spectra of stable isomers of **MotorPhos** and [(**MotorPhos**)PdCl_2_] and after irradiation to PSS with a 365 nm LED. Conditions: DMSO, 20 °C, 1.5 ×10^–5^ M. Inset: fatigue study on [(**MotorPhos**)PdCl_2_] showing *A*_370nm_ over the course of 10 consecutive irradiations to PSS, followed by complete THI. Conditions: DMSO, 100 °C, 1.5 × 10^–5^ M. (B) Stack of ^1^H NMR spectra of [(**MotorPhos**)PdCl_2_] before irradiation, after irradiation to PSS with a 365 nm LED, and after complete subsequent THI. Conditions: DMSO-*d*_*6*_, 80 °C, 5.0 × 10^–3^ M. (C) Stack of ^31^P NMR spectra of [(**MotorPhos**)PdCl_2_] before irradiation, after irradiation to PSS with a 365 nm LED, and after complete subsequent THI. Conditions: DMSO-*d*_*6*_, 80 °C, 5.0 × 10^–3^ M.

After having established the structure of [(**MotorPhos**_**s**_)PdCl_2_], we set out to study its rotation by means of UV–vis absorption and NMR spectroscopy. By measuring the absorbance of [(**MotorPhos**_**s**_)PdCl_2_] in DMSO, no local maxima were found, and only a saddle point at 355 nm and a long tail extending to ∼480 nm were observed (Figure 4A).

This is consistent with the color of [(**MotorPhos**_**s**_)PdCl_2_], DFT calculations (Figure S17), and the spectral properties of structurally related [(XantPhos)PdCl_2_].^35^ On the other hand, for parent compound **MotorPhos**_**s**_, an absorption maximum was observed at 333 nm with a shoulder at ∼355 nm and the onset at ∼400 nm. Pd(II) complexation, therefore, shows potential for red-shifting motors’ absorption, similar to prior results with Ru(II).^26^ Irradiation to the respective photostationary states (PSSs) with a 365 nm LED leads to a decrease in absorbance from the onset of the absorption spectra until 328 nm (**MotorPhos**) and between 417 and 315 nm ([(**MotorPhos**)PdCl_2_]), followed by a slight increase until 296 and 293 nm, respectively. For both compounds, these two spectral regions are separated by a clean isosbestic point (Figure S3A,C), confirming the selective and unimolecular nature of these photochemical *E*/*Z* isomerizations. The rate of formation of [(**MotorPhos**_**m**_)PdCl_2_] was independent of the temperature, confirming a barrierless reaction in line with the proposed *E*/*Z* isomerization and ruling out an alternative THI in the backward direction (Figure S5).

By keeping these samples at elevated temperatures after irradiation to PSS, the spectra revert to the ones of the stable isomers following complete THI and showing the same isosbestic points as for the photochemical *E*/*Z* isomerizations (Figure S3B,D). This observation confirms that, following photochemical isomerization and subsequent THI, species identical to those before irradiation are formed for both motors. In the case of [(**MotorPhos**_**s**_)PdCl_2_], this requires the PdCl_2_ unit to swing to the other side of the motor backbone. This result is also in contrast to two earlier reported first-generation molecular motors whose capacity for photoisomerization was lost upon complexation to Pd.^36,37^ Note that during our experiments, no photodissociation of [(**MotorPhos**)PdCl_2_] was observed, which would have been detectable by a blue shift of the absorption spectrum. Thermal *Z*/*E* isomerization as an alternative mechanism was ruled out on the basis of an earlier report on a closely related second-generation molecular motor featuring an unsymmetric lower half.^38^

To further study the isomerization behavior of our novel motors, we followed the photochemical as well as thermal parts of the cycle by ^1^H NMR (Figures 4B and S8 and S9). Spectra were recorded before irradiation, after irradiation to PSS with a 365 nm LED, and after complete THI. In both cases, irradiation gives rise to one new species, which can be assigned to the metastable isomer by comparison with earlier reports.^38^ Upon removal of the light source, the spectra revert to the original ones, confirming no lasting structural change is taking place during these isomerizations. For [(**MotorPhos**)PdCl_2_], we also recorded ^31^P NMR spectra at each stage (Figures 4C and S10). The measured shifts of 22 ppm and 23 ppm for the stable isomer match well with those of *cis*-[(XantPhos)PdCl_2_] (23 ppm)^34^ and *cis*-[(XantPhos)PdI_2_] (20 ppm), whereas *trans*-[(XantPhos)PdI_2_] shows a shift of 9 ppm^39^ (*trans*-[(XantPhos)PdCl_2_] has not been reported). The fact that no significant difference is observed after irradiation demonstrates that there is no change to the phosphines and by extension to the Pd moiety, ruling out the involvement of the lower half oxygen in the isomerization as well as potential ligand dissociation or *cis*–*trans* isomerization at the Pd center upon formation of the metastable isomer.

Removing the light source allows the spectrum to relax back to the original one further demonstrating the full reversibility of the system. The ratios of metastable/stable isomers after irradiation at room temperature until no further change was observed, i.e., at PSS, were studied for both compounds using ^1^H NMR with different-wavelength LEDs (Table S2). Using our standard 365 nm LED, ratios of 91:9 and 88:12 (metastable/stable) were obtained for **MotorPhos** and [(**MotorPhos**)PdCl_2_], respectively. Using 455 nm excitation, no isomerization was observed for bare **MotorPhos**_**s**_, whereas [(**MotorPhos**_**s**_)PdCl_2_] still showed the formation of 80% metastable isomer at PSS, confirming its operation with visible light. This further decreased to 73% at 470 nm, and finally, no isomerization could be detected using a 490 nm LED.

Activation parameters of the thermal isomerization of the free diphosphine ligand and Pd complex were obtained by Eyring analysis of the kinetic profile of the recovery of the stable isomers from the metastable isomers at different temperatures, followed by ^1^H NMR (Table 1). Δ^‡^*G*_*Exp*_(20 °C) was found to be ∼8 kJ·mol^–1^ higher after complexation to PdCl_2_.

**Table 1:** Summary of Activation Parameters Obtained from Eyring Analysis of the Thermal Isomerization of Metastable Isomers **MotorPhos**_**m**_ and [(**MotorPhos**_**m**_)PdCl_2_] in DMSO-*d*_*6*_

	Δ^‡^*G*_exp_(20 °C) (kJ·mol^–1^)	Δ^‡^*H*_exp_(20 °C) (kJ·mol^–1^)	Δ^‡^*S*_exp_(20 °C) (J·mol^–1^·K^–1^)	*t*_1/2_(20 °C) (min)
**MotorPhos**_**m**_	95.3 ± 1.1	109.4	48.2	181 ± 11
[(**MotorPhos**_**m**_)PdCl_2_]	103.1 ± 1.0	113.5	35.7	4477 ± 515

Fatigue studies of 10 consecutive irradiations (365 nm LED) to PSS, followed by complete THI, equaling a 180° rotation each, were performed at 85 and 100 °C for **MotorPhos** (Figure S4) and [(**MotorPhos**)PdCl_2_] (Figure 4A, inset), respectively. Both compounds show good fatigue resistance, confirming their stability under irradiation and prolonged heat stress.

### DFT Study of Thermal Relaxation Pathways

**Figure 5:**
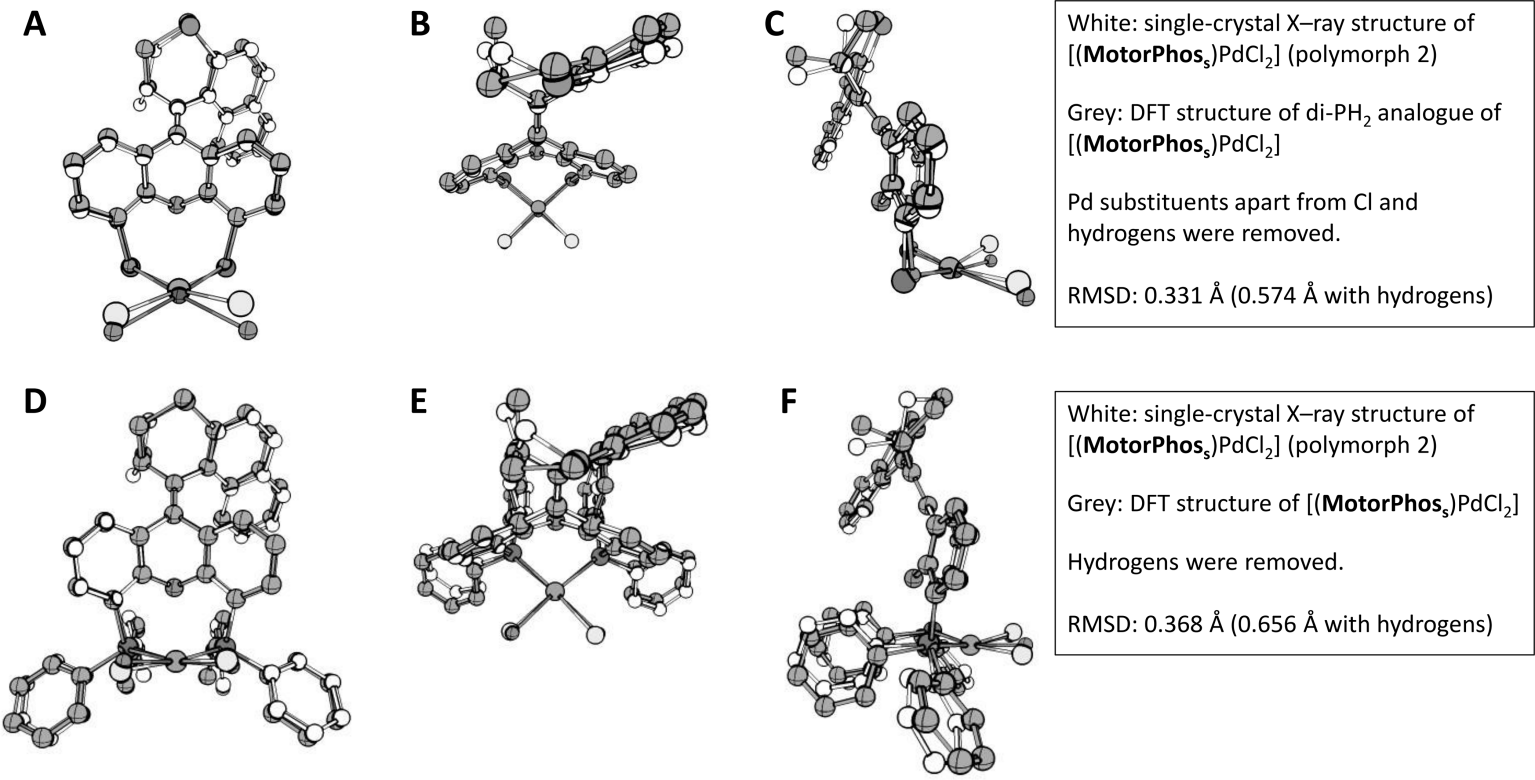
(A–C) Front, top, and side views of overlaid structures of the single-crystal X-ray structure of [(**MotorPhos**_**s**_)PdCl_2_] (polymorph 2) and the DFT-optimized PH_2_ analogue (ωB97X-D/C,H,O:6-31G(d,p), Cl,P,S:6-311G(2d,p), Pd:SDD). Pd substituents apart from Cl were removed from the experimental structure for comparison. A weighted root-mean-square deviation (RMSD) of 0.331 Å was determined. (D–F) Front, top, and side view of overlaid structures of the single-crystal X-ray structure of [(**MotorPhos**_**s**_)PdCl_2_] (polymorph 2) and the DFT-optimized structure (ωB97X-D/C,H,O:6-31G(d,p), Cl,P,S:6-311G(2d,p), Pd:SDD). A weighted RMSD of 0.368 Å was determined.

To present a complete picture of the thermal isomerization of our system, we conducted a DFT study on a model compound containing PH_2_ instead of PPh_2_ groups to reduce the computational cost. Initially, to confirm that the obtained structure is representative, we optimized the structures of both the stable isomer of the PH_2_ analogue (ωB97X-D/C,H,O:6-31G(d,p), Cl,P,S:6-311G(2d,p), Pd:SDD) (Figure 5A–C) and, based on this structure, the complete [(**MotorPhos**_**s**_)PdCl_2_] complex (ωB97X-D/C,H,O:6-31G(d,p), Cl,P,S:6-311G(2d,p), Pd:SDD) (Figure 5D–E) and compared them to the single-crystal X-ray structure discussed earlier.

As can be taken from Figure 5, good matches were found for both comparisons with weighted RMSD values of 0.331 and 0.368 Å. This confirms that the structures obtained by DFT are representative for the experimental structures, and results obtained based on these calculations can be used to study this system. Using B3LYP instead of ωB97X-D gave worse matches between calculation and experiment.

**Figure 6:**
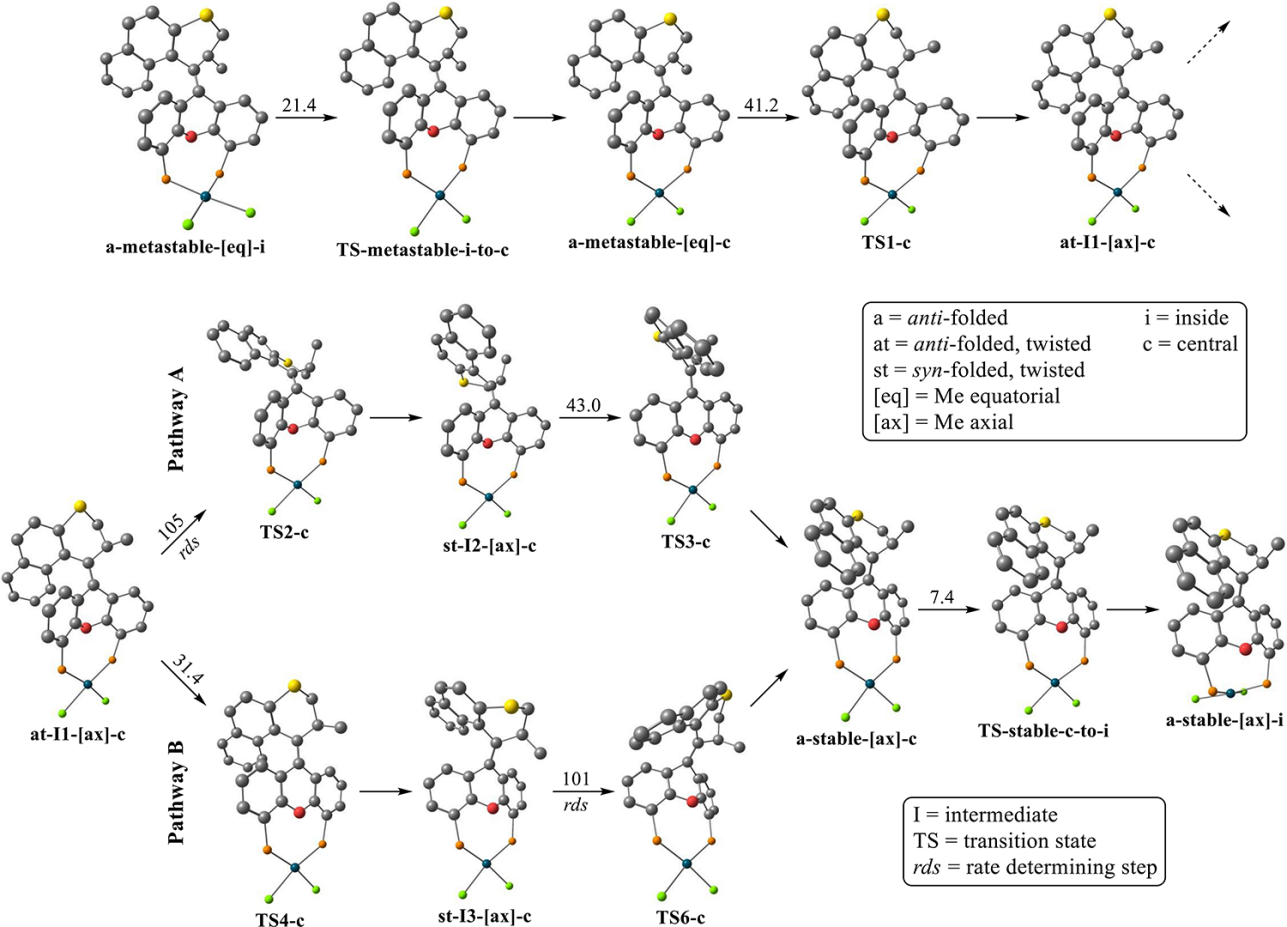
Thermal isomerization pathways with the lowest barriers for the rate-determining step leading from the metastable isomer obtained after photoisomerization (a-metastable-[eq]-i) to the global minimum stable isomer (a-stable-[ax]-i). The PPh_2_ groups of [(**MotorPhos**)PdCl_2_] were replaced with PH_2_ to reduce the computational cost (ωB97X-D/C,H,O:6-31G(d,p), Cl,P,S:6-311G(2d,p), Pd:SDD). Activation barriers for each step are given. Hydrogens were omitted for clarity. Pathways (A) and (B) differ in the order of steps; in (A), sliding of the naphthalene unit over the lower half is followed by a ring flip in the lower half with (B) following the reverse order.

With these results in hand, we conducted our study into the thermal isomerization pathway of the di-PH_2_ compound. We based this study on a recent publication where the thermal isomerization pathway of a series of related motors was studied by DFT.^40^ Two pathways A and B could be established, differing only in the order of steps (Figure 6).

Both pathways start with the PdCl_2_ moiety being pushed down from an inside position, relative to the folding of the lower half, to a central position pointing downward. This is followed by a ring flip of the upper half thiopyran moving the Me group on the stereogenic center from an equatorial to an axial position reaching intermediate I1. At this point, the two pathways diverge; in pathway A, the naphthalene unit first slides over the lower half via TS2 before a ring flip in the lower half leads to a stable isomer with the PdCl_2_ moiety still in the central position. In pathway B, these two steps are reversed. In both cases, the naphthalene unit sliding over the lower half represents the rate-determining step with barriers of 105 kJ·mol^–1^ (pathway A, TS2) and 101 kJ·mol^–1^ (pathway B, TS6). These energies match well with the experimental activation barrier for THI (Table 1). From the stable isomer that is obtained, where the two pathways merge, the global minimum is reached after the PdCl_2_ group swings back into the inside position, thereby completing the thermal isomerization.

It should be mentioned that the two parts of the movement, rotation and oscillation, have distinct transition states, meaning that one could also find alternative pathways where the oscillatory steps are interjected at different points in the rotation (Figure S15A). However, the activation barriers for the rate-determining steps in both pathways were found to be the lowest when the PdCl_2_ group is in the central position. Optimizing the geometries of the three distinct conformers of the stable and metastable isomers of the [(**MotorPhos**_**s**_)PdCl_2_] complex in DMSO using the SMD solvent model revealed the ones with the PdCl_2_ group in the inside position to be the most stable, analogous to the di-PH_2_ compound (Table S5).

## Summary and Conclusions

The design of systems capable of performing coupled motions is a central part in the development of molecular machines capable of carrying out more advanced tasks than simple molecular motors, as they allow for the conversion of one kind of motion into another. We present here an example where the rotation of a second-generation molecular motor is coupled to an oscillatory motion. This is achieved by placing two phosphine groups in the lower half, which are then used to form a complex with PdCl_2_, with the PdCl_2_ moiety assuming an almost perfectly perpendicular position relative to the motor backbone. During thermal relaxation following photoisomerization, the PdCl_2_ group is gradually pushed to the other side of the xanthene lower half describing an oscillatory movement. In addition, the system described in this study is simple in its preparation and shows exceptional stability. We therefore believe it has great potential to serve as the foundation for the design of more complex functional systems requiring the conversion of rotary to oscillatory motion.

## Supporting Information Available

Experimental details, synthesis procedures, UV–vis spectra, NMR spectra, single-crystal X-ray spectroscopy, and details for DFT calculations; cartesian coordinates and energies of calculated structures (PDF)
