# Supplementary material for: Coupled Rotary and Oscillatory Motion in a Second-Generation Molecular Motor Pd Complex
Source: J Am Chem Soc. 2023 Jan 5;145(2):822–9. doi: 10.1021/jacs.2c08267 (PMC9853862; doi:10.1021/jacs.2c08267)
Supplement: Supplementary file 1 — ja2c08267_si_001.pdf [file ja2c08267_si_001.pdf]

SUPPORTING INFORMATION

Coupled Rotary and Oscillatory Motion in a Second-Generation Molecular Motor Pd Complex

*Lukas Pfeifer[1‡], Charlotte N. Stindt[1], Ben L. Feringa[1,2]**

* Corresponding author. Email: b.l.feringa@rug.nl (B.L.F.);

[1]Stratingh Institute for Chemistry, University of Groningen, Nijenborgh 4, 9747 AG Groningen, The Netherlands.

[2]Zernike Institute for Advanced Materials, University of Groningen, Nijenborgh 4, 9747 AG Groningen, The Netherlands.

[‡]Present Address: Laboratory of Photonics and Interfaces, Department of Chemistry and Chemical Engineering, École Polytechnique Fédérale de Lausanne; 1015 Lausanne, Switzerland.

Table of Contents

1. General Information 3

2. Preparation and Characterisation of Compounds 3

3. Single-Crystal X-Ray Diffraction 9

4. UV-vis Absorption Studies 11

5. NMR Studies 15

5.1. ROESY NMR Studies 15

5.2. Following 180° Rotations by ^{1}H NMR 16

5.3. *Determination of PSS Compositions* 19

5.4. *Eyring Analysis of THIs* **$MotorPhos_m$** and [(**$MotorPhos_m$**)$PdCl_2$] 20

6. Computational Analysis 22

7. References 28

8. NMR Spectra 29

9. Coordinates and Energies of DFT Structures 35

9.1. PH_2 Analogue of [(MotorPhos)$PdCl_2$] 35

9.2. [(MotorPhos)$PdCl_2$] 108

9.3. *[(MotorPhos)$PdCl_2$] in DMSO* 117

1. General Information

Reagents were purchased from Sigma Aldrich, Acros or TCI Europe and were used as received. Solvents were reagent grade and used without prior water removal unless otherwise indicated. Anhydrous solvents were obtained from an MBraun SPS-800 solvent purification system or directly bought from Acros. Solvents were degassed by three freeze-pump-thaw cycles. Solvents used for UV-vis absorption studies were of spectrophotometric grade.

Flash column chromatography was performed on a Büchi Reveleris purification system using Büchi silica cartridges. Thin layer chromatography was carried out on aluminium sheets coated with silica gel 60 F254 (Merck). Compounds were visualized with a UV lamp and/or by staining with $KMnO_4$, CAM or vanillin.

^{1}H and ^{13}C NMR spectra were recorded on a Varian Mercury-Plus 400 or a Bruker Avance 600 NMR spectrometer at 298 K unless otherwise indicated. Chemical shifts are given in parts per million (ppm) relative to the residual solvent signal. Multiplets in ^{1}H NMR spectra are designated as follows: s (singlet), d (doublet), t (triplet), q (quartet), p (pentet), s (sextet), m (multiplet), br (broad). High resolution mass spectrometry (ESI) was performed on an LTQ Orbitrap XL spectrometer. Steady-state UV-vis absorption spectra were recorded on an Agilent 8453 UV-vis Diode Array System, equipped with a Quantum Northwest Peltier controller, in 10 mm quartz cuvettes. Irradiation experiments were performed using fiber-coupled LEDs (M365F1, M455F1, M470F3, M490F3) obtained from Thorlabs Inc..

2. Preparation and Characterisation of Compounds

2-Methyl-3-(naphthalen-2-ylthio)propanoic acid (S1)

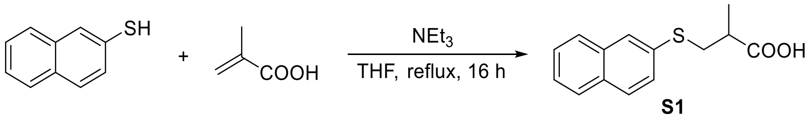

S1

To a solution of 2-naphthalenethiol (8.01 g, 50.0 mmol) in THF (100 mL) was added NEt_3 (13.94 mL, 100.0 mmol) and methacryclic acid (8.48 mL, 100 mmol) before heating the mixture to reflux for 16 h. After cooling to room temperature it was diluted with EtOAc and washed with 1 M aq. HCl. The organic layer was dried over Na_2SO_4 before removing volatiles under reduced pressure to obtain the crude product as a colourless oil. Hexane was added and the mixture was sonicated to induce formation of a white precipitate. This was filtered off and rinsed with hexane to give **S1** (9.127 g, 37.05 mmol, 74%) as a white solid. Data is in accordance with literature.[1]

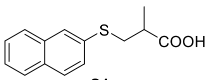

S1

^{1}H NMR (600 MHz, $CDCl_3$) δ 7.84 – 7.81 (m, 1H), 7.81 – 7.78 (m, 1H), 7.78 – 7.72 (m, 2H), 7.51 – 7.41 (m, 3H), 3.39 (dd, *J* = 13.5, 6.9 Hz, 1H), 3.02 (dd, *J* = 13.5, 7.1 Hz, 1H), 2.76 (s, *J* = 7.0 Hz, 1H), 1.33 (d, *J* = 7.1 Hz, 3H); **^{13}C NMR** (151 MHz, $CDCl_3$) δ 180.3, 133.9, 133.0, 132.2, 128.8, 128.5, 128.1, 127.9, 127.4, 126.8, 126.1, 39.6, 37.1, 16.7.

2-Methyl-2,3-dihydro-1*H*-benzo[*f*]thiochromen-1-one (S2)

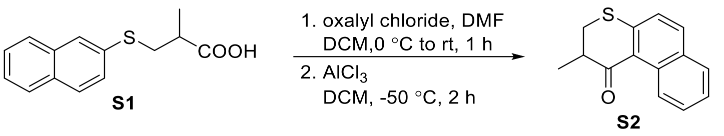

In a dried flask a solution of **S1** (4.43 g, 18.0 mmol) and DMF (3 drops) in DCM (70 mL) was prepared and cooled to 0 °C. Oxalyl chloride (3.05 mL, 36.0 mmol) was added dropwise, the resulting solution was allowed to warm up to room temperature before stirring for 1 h. Volatiles were removed under reduced pressure and the remaining solid was dissolved in DCM (70 mL). The resulting solution was cooled down to –50 °C and $AlCl_3$ (3.60 g, 27.0 mmol) was added portionwise over 20 min before stirring for further 2 h. The mixture was warmed up to 0 °C and quenched by careful addition of 1 M aq. HCl. The layers were separated and the aqueous layer was extracted with DCM. The combined organic layers were dried over Na_2SO^4 before removing volatiles under reduced pressure. Flash column chromatography (SiO_2, dry load on celite, 5% EtOAc in pentane) gave **S2** (3.864 g, 16.92 mmol, 94%) as a yellow oil. Data is in accordance with literature.[1]

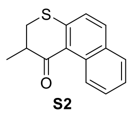

^{1}H NMR (600 MHz, $CDCl_3$) δ 9.13 – 8.95 (m, 1H), 7.77 (d, *J* = 8.6 Hz, 1H), 7.75 – 7.71 (m, 1H), 7.62 – 7.53 (m, 1H), 7.49 – 7.39 (m, 1H), 7.25 – 7.22 (m, 1H), 3.29 – 3.22 (m, 1H), 3.22 – 3.15 (m, 1H), 3.14 – 3.07 (m, 1H), 1.40 (d, *J* = 6.7 Hz, 3H); **^{13}C NMR** (151 MHz, $CDCl_3$) δ 199.4, 144.2, 133.5, 132.6, 131.8, 129.1, 128.6, 125.9, 125.7, 125.5, 125.2, 43.0, 33.0, 15.5.

(*E*)-(2-Methyl-2,3-dihydro-1*H*-benzo[*f*]thiochromen-1-ylidene)hydrazine (1)

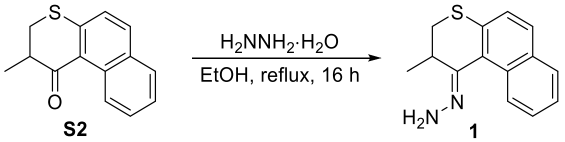

A flask was charged with **S2** (1.83 g, 8.00 mmol), EtOH (4.7 mL) and hydrazine monohydrate (4.70 mL). The mixture was put under a nitrogen atmosphere and heated at reflux for 16 h. After cooling to room temperature a first crop of **1** was filtered off as light yellow crystals. DCM and H_2O were added to the filtrate and the two layers were separated. The aqueous layer was extracted with DCM and the combined organic phases were dried over Na_2SO_4 before removing volatiles under reduced pressure. Flash column chromatography (SiO_2, dry load on celite, 85:10:5–30:65:5 pentane/EtOAc/NEt_3) gave a second crop of **1** (combined yield: 1.116 g, 4.605 mmol, 58%) as a light yellow solid. Data is in accordance with literature.[1]

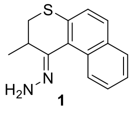

^{1}H NMR (600 MHz, $CDCl_3$) δ 8.42 (d, *J* = 8.6 Hz, 1H), 7.76 (d, *J* = 8.1 Hz, 1H), 7.65 (d, *J* = 8.5 Hz, 1H), 7.48 (ddd, *J* = 8.5, 6.7, 1.4 Hz, 1H), 7.41 (ddd, *J* = 8.0, 6.8, 1.2 Hz, 1H), 7.34 (d, *J* = 8.5 Hz, 1H), 5.59 (br s, 2H), 3.79 – 3.31 (m, 1H), 3.20 (dd, *J* = 12.8, 6.0 Hz, 1H), 2.71 (dd, *J* = 12.8, 9.7 Hz, 1H), 1.33 (d, *J* = 6.8 Hz, 3H); **^{13}C NMR** (151 MHz, $CDCl_3$) δ 149.8, 136.0, 133.2, 132.3, 131.0, 128.2, 128.0, 127.0, 126.4, 126.1, 125.4, 36.7, 34.4, 15.0.

9,9-Dimethoxy-9H-xanthene (S3)

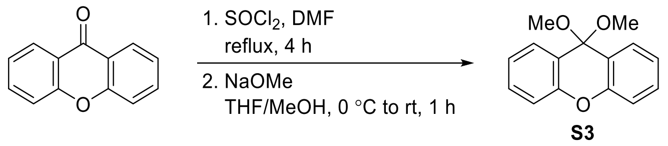

A mixture of xanthone (12.8 g, 65.0 mmol), $SOCl_2$ (25.9 mL) and DMF (0.33 mL) was heated at reflux for 4 h before cooling down to room temperature and removing volatiles under reduced pressure. Residual $SOCl_2$ was removed by co-evaporation with toluene. The obtained solid was dissolved in THF (65 mL) and the resulting solution was slowly added to a solution of NaOMe (25% in MeOH, 49.0 mL, 215 mmol) at 0 °C under nitrogen atmosphere. The resulting mixture was warmed up to room temperature and stirred for 1 h before removing volatiles under reduced pressure. The obtained solid was dissolved in DCM and sat. aq. $NaHCO_3$, the layers were separated and the aqueous layer was extracted with DCM. The combined organic layers were dried over Na_2SO_4 before volatiles were removed under reduced pressure to give **S3** (15.204 g, 62.76 mmol, 97%) as an off-white solid which was used without further purification. Data is in accordance with literature.[2]

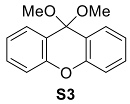

^{1}H NMR (400 MHz, $CDCl_3$) δ 7.73 (dd, J = 7.8, 1.6 Hz, 2H), 7.49 – 7.34 (m, 2H), 7.29 – 7.15 (m, 4H), 2.94 (s, 6H); **^{13}C NMR** (101 MHz, $CDCl_3$) δ 153.4, 130.3, 127.5, 123.5, 118.9, 116.6, 96.7, 52.0.

4,5-Diiodo-9H-xanthen-9-one (S4)

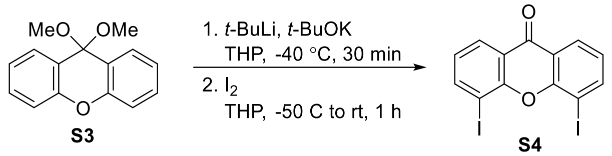

A flame dried Schlenk flask was charged with **S3** (242 mg, 1.00 mmol) and t-BuOK (337 mg, 3.00 mmol). THP (6.0 mL) was added and the resulting solution was cooled to –40 °C. t-BuLi (1.7 M in pentane, 1.76 mL, 3.00 mmol) was added dropwise and the resulting mixture was stirred for an additional 30 min. It was then cooled to –50 °C before adding a solution of I_2 (1.00 g, 3.94 mmol) in THP (2.0 mL). The cooling bath was removed and the reaction mixture allowed to warm to room temperature over 1 h. It was then quenched by adding H_2O and subsequent addition of 1 M aq. HCl until pH = 3 before extracting with EtOAc. The combined organic layers were washed with sat. aq. $Na_2S_2O_3$, H_2O and brine before drying over Na_2SO_4 and removing volatiles under reduced pressure. Flash column chromatography (SiO_2, dry load on celite, 20–60% DCM in pentane) gave **S4** (220 mg, 0.491 mmol, 49%) as a fluffy white solid. Data is in accordance with literature.[3]

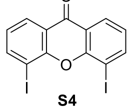

^{1}H NMR (600 MHz, $CDCl_3$) δ 8.31 (d, J = 7.9 Hz, 2H), 8.25 (d, J = 7.6 Hz, 2H), 7.19 (t, J = 7.7 Hz, 2H); **^{13}C NMR** (151 MHz, $CDCl_3$) δ 176.4, 155.5, 145.2, 127.3, 126.1, 122.2, 85.1.

4,5-diiodo-9H-xanthene-9-thione (2)

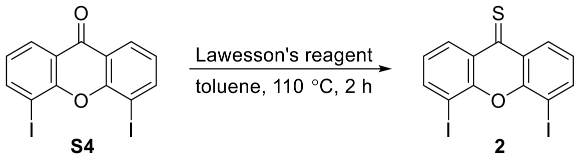

A flame dried Schlenk flask was charged with **S4** (896 mg, 2.00 mmol) and Lawesson's reagent (2.43 g, 6.00 mmol). Degassed (bubbling N_2 for 30 min) toluene (3.0 mmol) was added before heating at 110 °C for 30 min. The mixture was then cooled to room temperature and volatiles were removed under reduced pressure. Flash column chromatography (SiO_2, dry load on celite, 20–40% DCM in hexane) gave **2** (842 mg, 1.81 mmol,91%) as a green solid.

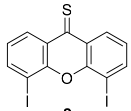
2

^{1}H NMR (600 MHz, DMSO-*d*$_6$) δ 8.55 (dd, *J* = 8.2, 1.5 Hz, 2H), 8.48 (dd, *J* = 7.5, 1.5 Hz, 2H), 7.46 – 7.16 (m, 2H); **^{13}C NMR** (151 MHz, DMSO-*d*$_6$) δ 203.9, 149.8, 145.6, 129.6, 128.8, 127.3, 86.8; **HRMS** (CI pos) *m/z* calcd for $C_{13}H_7I_2OS$ $[M+H]^+$ 464.83015, found 464.82931.

2-Methyl-2,3-dihydrodispiro[benzo[*f*]thiochromene-1,2'-thiirane-3',9''-xanthene] (S5)

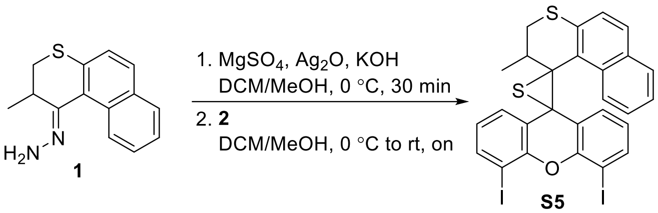

In a Schlenk flask a solution of **1** (242 mg, 1.00 mmol) in DCM (11.5 mL) was prepared. After cooling to 0 °C $MgSO_4$ (349 mg, 2.90 mmol), Ag_2O (463 mg, 2.00 mmol) and a sat. solution of KOH in MeOH (574 µL) were added and the resulting mixture was stirred at 0 °C for 30 min during which it turned red. Solids were removed by filtration over celite and the filtrate was placed in a second Schlenk flask and cooled to 0 °C. A solution of **2** (464 mg, 1.00 mmol) in DCM (17.2 mL) was added before allowing the mixture to warm up to room temperature and stirring overnight. Volatiles were removed under reduced pressure and subsequent flash column chromatography (SiO_2, dry load on celite, 5–15% DCM in pentane) gave **S5** (523 mg, 0.77 mmol, 77%) as an off-white solid.

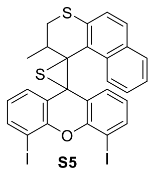
S5

^{1}H NMR (600 MHz, $CDCl_3$) δ 8.60 (d, *J* = 8.6 Hz, 1H), 7.87 (dd, *J* = 7.7, 1.5 Hz, 1H), 7.75 (dd, *J* = 7.8, 1.5 Hz, 1H), 7.72 (d, *J* = 8.1 Hz, 1H), 7.55 (ddd, *J* = 8.4, 6.7, 1.4 Hz, 1H), 7.44 – 7.38 (m, 2H), 7.36 (dd, *J* = 7.7, 1.5 Hz, 1H), 6.97 (d, *J* = 8.4 Hz, 1H), 6.95 (t, *J* = 7.8 Hz, 1H), 6.13 (dd, *J* = 7.9, 1.5 Hz, 1H), 5.88 (t, *J* = 7.8 Hz, 1H), 2.98 – 2.91 (m, 1H), 2.48 (dd, *J* = 12.5, 9.5 Hz, 1H), 2.21 (dd, *J* = 12.5, 7.0 Hz, 1H), 1.18 (d, *J* = 6.8 Hz, 3H); **^{13}C NMR** (151 MHz, $CDCl_3$) δ 154.0, 152.9, 141.0, 139.2, 138.2, 134.6, 132.7, 130.4, 130.0, 128.8, 127.9, 127.5, 126.4, 126.0, 125.0, 124.2, 123.5, 123.5, 123.0, 122.0, 84.6, 82.9, 65.0, 54.6, 42.0, 37.0, 21.4; **HRMS** (ESI pos) *m/z* calcd for $C_{27}H_{19}I_2OS_2$ $[M+H]^+$ 676.89612, found 676.89401.

4,5-Diiodo-9-(2-methyl-2,3-dihydro-1*H*-benzo[*f*]thiochromen-1-ylidene)-9*H*-xanthene (3_s)

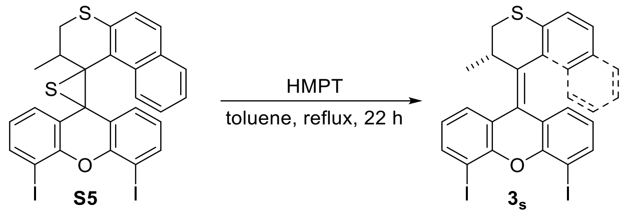

To a solution of **S5** (470 mg, 0.69 mmol) in toluene was added HMPT (884 µL, 4.86 mmol) before heating at reflux for 22 h. The resulting mixture was cooled to room temperature and volatiles were removed under reduced pressure. Flash column chromatography (SiO_2, dry load on celite, 5–15% DCM in pentane) gave **3_s** (439 mg, 0.68 mmol, 99%) as an off-white solid.

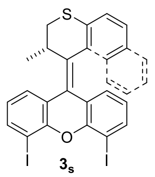

^{1}H NMR (600 MHz, $CDCl_3$) δ 7.87 (dd, *J* = 7.8, 1.4 Hz, 1H), 7.64 (d, *J* = 8.5 Hz, 1H), 7.60 (dd, *J* = 8.1, 1.3 Hz, 1H), 7.55 (dd, *J* = 7.7, 1.4 Hz, 1H), 7.37 (d, *J* = 8.5 Hz, 1H), 7.31 (dd, *J* = 7.8, 1.4 Hz, 1H), 7.21 (dd, *J* = 8.6, 1.1 Hz, 1H), 7.15 (ddd, *J* = 8.0, 6.7, 1.2 Hz, 1H), 7.06 – 6.98 (m, 2H), 6.16 (dd, *J* = 7.8, 1.4 Hz, 1H), 5.99 (t, *J* = 7.8 Hz, 1H), 4.21 (pd, *J* = 6.8, 2.4 Hz, 1H), 3.70 (dd, *J* = 11.0, 6.9 Hz, 1H), 3.17 (dd, *J* = 11.0, 2.4 Hz, 1H), 0.79 (d, *J* = 6.7 Hz, 3H); **^{13}C NMR** (151 MHz, $CDCl_3$) δ 154.6, 153.4, 138.4, 137.6, 137.2, 135.3, 131.6, 130.3, 130.2, 128.3, 128.2, 128.0, 127.4, 126.9, 126.3, 126.1, 125.6, 125.3, 124.8, 124.5, 123.8, 122.6, 84.8, 82.9, 36.9, 31.4, 18.7; **HRMS** (ESI pos) *m/z* calcd for $C_{27}H_{19}I_2OS$ $[M+H]^+$ 644.92405, found 644.92034.

(9-(2-Methyl-2,3-dihydro-1*H*-benzo[*f*]thiochromen-1-ylidene)-9*H*-xanthene-4,5-diyl)bis(diphenyl-phosphane) (MotorPhos$_s$)

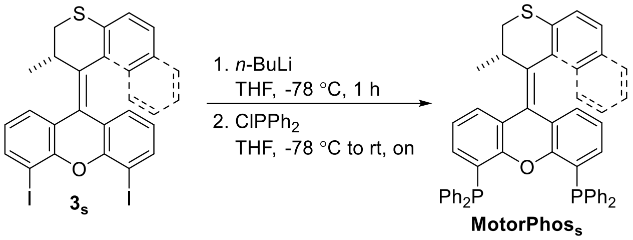

A solution of **3_s** (110 mg, 0.171 mmol) in degassed (three freeze-pump-thaw cycles) THF (4.0 mL) in a Schlenk flask was cooled to –78 °C before *n*-BuLi (235 µL, 0.376 mmol) was added dropwise. The mixture was stirred for 1 h at –78 °C before a solution of $ClPPh_2$ (69 µL, 0.38 mmol) in degassed (three freeze-pump-thaw cycles) THF (0.50 mL) was added dropwise. After stirring at –78 °C for 1 h the reaction mixture was allowed to warm to room temperature and stirred overnight. Volatiles were removed under reduced pressure and subsequent flash column chromatography (SiO_2, dry load on celite, 5–30% DCM in pentane) gave **MotorPhos$_s$** (74 mg, 0.097 mmol, 57%) as a white solid.

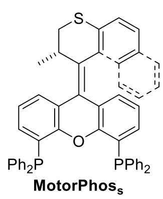

^{1}H NMR (600 MHz, $CDCl_3$) δ 7.64 (d, *J* = 8.6 Hz, 1H), 7.62 (d, *J* = 8.0 Hz, 1H), 7.58 (d, *J* = 7.6 Hz, 1H), 7.39 (d, *J* = 8.5 Hz, 1H), 7.36 – 7.15 (m, 20H), 7.14 (t, *J* = 7.6 Hz, 1H), 6.99 (qd, *J* = 7.6, 7.0, 1.3 Hz, 3H), 6.76 (ddd, *J* = 7.6, 3.6, 1.5 Hz, 1H), 6.23 (dd, *J* = 7.7, 1.6 Hz, 1H), 6.19 (ddd, *J* = 7.6, 3.9, 1.6 Hz, 1H), 6.11 (t, *J* = 7.6 Hz, 1H), 4.28 (pd, *J* = 6.7, 2.2 Hz, 1H), 3.75 (dd, *J* = 11.0, 7.0 Hz, 1H), 3.18 (dd, *J* = 11.0, 2.3 Hz, 1H), 0.78 (d, *J* = 6.7 Hz, 3H); **^{13}C NMR** (126 MHz, $CDCl_3$) δ 157.1 (d, *J* = 17.1 Hz), 155.5 (d, *J* = 18.5 Hz), 137.6 (d, *J* = 14.0 Hz), 137.4 (d, *J* = 12.8 Hz), 137.2 (d, *J* = 13.0 Hz, 2C), 135.3, 135.1, 134.4 (d, *J* = 20.0 Hz, 2C), 134.0 (d, *J* = 17.3 Hz), 133.9 (d, *J* = 17.5 Hz), 133.1, 132.3, 131.7, 131.3, 130.5, 129.0, 128.6, 128.5 (d, *J* = 5.6 Hz), 128.5 (2C), 128.4, 128.4 (d, *J* = 6.3 Hz), 128.3 (d, *J* = 6.1 Hz), 128.2 (d, *J* = 6.3 Hz), 128.0, 127.8, 127.5, 126.7 (d, *J* = 19.8 Hz), 126.2, 125.9 (d, *J* = 1.6 Hz), 125.8, 125.7 (d, *J* = 19.6 Hz), 124.6 (d, *J* = 2.0 Hz), 124.5, 124.2, 123.6 (d, *J* = 2.5 Hz), 123.5, 122.7, 37.2, 31.2, 18.8; **^{13}C-{^{31}P) NMR** (126 MHz, $CDCl_3$) δ 157.1, 155.5, 137.6, 137.4, 137.2 (2C), 135.3, 135.1, 134.4 (2C), 134.0, 133.9, 133.1, 132.3, 131.7, 131.3, 130.5, 129.0, 128.6, 128.5, 128.5 (2C), 128.4, 128.4, 128.3, 128.2, 128.0, 127.8, 127.5, 126.7, 126.2, 125.9, 125.8, 125.7, 124.5, 124.5, 124.2, 123.6, 123.5, 122.7, 37.2, 31.2, 18.8; **^{31}P NMR** (243 MHz, $CDCl_3$) δ –18.05 (d, *J* = 39.0 Hz), –18.94 (d, *J* = 39.0 Hz); **HRMS** (ESI pos) *m/z* calcd for $C_{51}H_{39}OP_2S$ $[M+H]^+$ 761.21914, found 761.22333.

(9-(2-Methyl-2,3-dihydro-1*H*-benzo[*f*]thiochromen-1-ylidene)-9*H*-xanthene-4,5-diyl)bis(diphenyl-phosphane)·$PdCl_2$ [(MotorPhos$_s$)$PdCl_2$]

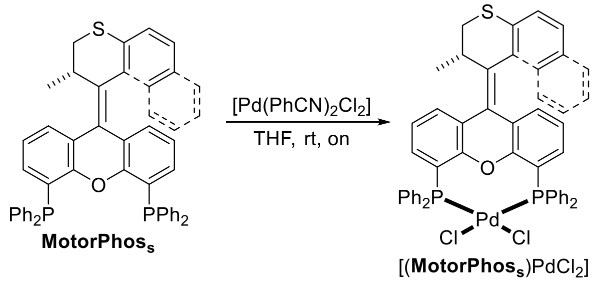

A Schlenk flask was charged with **MotorPhos$_s$** (60 mg, 0.079 mmol) and $[(PhCN)_2PdCl_2)$ (30 mg, 0.079 mmol). THF (3.8 mL) was added and the resulting solution was stirred at room temperature in the dark overnight. Volatiles were removed under reduced pressure and Et_2O (5.0 mL) was added. The mixture was sonicated until a uniform suspension was obtained. The solid was collected by filtration and rinsed with Et_2O and pentane. [(**MotorPhos$_s$**)$PdCl_2$] (69 mg, 0.074 mmol, 93%) was obtained as a light orange solid.

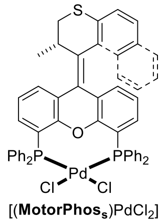

^{1}H NMR (600 MHz, DMSO-d_6) δ 8.11 (d, *J* = 7.7 Hz, 1H), 7.93 – 7.85 (m, 3H), 7.81 (d, *J* = 8.0 Hz, 1H), 7.76 – 7.70 (m, 2H), 7.67 (d, *J* = 8.3 Hz, 1H), 7.56 – 7.52 (m, 2H), 7.52 – 7.44 (m, 4H), 7.41 (d, *J* = 7.2 Hz, 1H), 7.38 (t, *J* = 7.2 Hz, 2H), 7.30 (dd, *J* = 6.9, 1.1 Hz, 1H), 7.26 (ddd, *J* = 8.3, 6.8, 1.5 Hz, 1H), 7.10 (t, *J* = 7.4 Hz, 1H), 7.05 (t, *J* = 7.4 Hz, 1H), 7.01 – 6.85 (m, 9H), 6.59 (td, *J* = 7.6, 1.3 Hz, 1H), 6.39 (dd, *J* = 8.0, 1.1 Hz, 1H), 4.44 (pd, *J* = 6.8, 2.5 Hz, 1H), 3.98 (dd, *J* = 11.6, 6.4 Hz, 1H), 3.33 – 3.31 (m, 1H), 0.93 (d, *J* = 6.5 Hz, 3H); **^{13}C NMR** (151 MHz, DMSO-d_6) δ 155.7 (d, *J* = 5.2 Hz), 154.6 (d, *J* = 4.9 Hz), 138.2, 136.3 (d, *J* = 9.5 Hz), 136.0 (d, *J* = 9.7 Hz), 135.5, 131.3, 131.2, 131.0, 130.8, 130.7, 130.5, 130.0, 129.9, 129.1 (d, *J* = 4.0 Hz), 128.8, 128.6, 128.6, 128.6, 128.5, 128.5, 128.4, 127.9, 127.8, 127.8, 127.8, 127.1 (d, *J* = 3.7 Hz), 125.9, 125.5 (d, *J* = 6.5 Hz), 125.4, 124.8, 124.1 (d, *J* = 7.1 Hz), 123.2, 122.0, 119.8, 119.4, 119.2, 118.8, 35.9, 30.6, 17.9; **^{31}P NMR**

(243 MHz, $CDCl_3$) δ 23.19, 22.21; **HRMS** (ESI pos) *m/z* calcd for $C_{51}H_{38}ClOP_2PdS$ $[M–Cl]^+$ 901.08365, found 901.08466.

3. Single-Crystal X-Ray Diffraction

A racemic mixture of [(**MotorPhos$_s$**)$PdCl_2$] was used for the preparation of single-crystals suitable for X-ray diffraction analysis. A saturated solution in DCM was prepared and upon slow diffusion of pentane vapour into this mixture the formation of clear, light yellow crystals of two different habits, blocks and planks, was observed.

Single-crystals were mounted on a cryoloop and placed in the nitrogen stream (100 K) of a Bruker-AXS D8 Venture diffractometer. Data collection and processing was carried out using the Bruker APEX3 software suite.[4] A multi-scan absorption correction was applied for polymorph 1, based on the intensities of symmetry-related reflections measured at different angular settings, while a numerical absorption correction was used for polymorph 2 (*SADABS*).[5] Both structures were found to be racemic crystallizing in space groups *P*–1 (polymorph 1) and $P2_1/c$ (polymorph 2), respectively. The structures were solved using *SHELXT*[6] and refinement was performed using *SHELXL*.[7] The hydrogen atoms were generated by geometrical considerations, constrained by idealized geometries and allowed to ride on their carrier atoms with an isotropic displacement parameter related to the equivalent displacement parameter of their carrier atoms. During the refinement of polymorph 1 four sites containing disordered solvent were identified. Two of them were modelled as DCM disordered over two sites whereas no suitable model could be found for the remaining two. The electron density in these remaining parts of the structure was removed using the *PLATON/SQUEEZE* routine.[8] Similarly, polymorph 2 also contained disordered solvent molecules in two sites for which no appropriate model could be found. The associated electron density was again removed using the *PLATON/SQUEEZE* routine.[8] No A- or B-level alerts were raised by CheckCIF for the fully refined structures.

Crystallographic data for polymorphs 1 and 2 of [(**MotorPhos$_s$**)$PdCl_2$] is summarized in Table S1. Final CIFs have been deposited in the Cambridge Structural Database (CSD 2194349–2194350).

Table S1. Crystallographic data for polymorphs 1 and 2 of [(**MotorPhos$_s$**)PdCl$_2$].

Nr.	LP-18072	LP-18073
Polymorph	# 1	# 2
Formula	C_{53} H_{42} Cl_6 O_1 P_2 Pd_1 S_1	C_{51} H_{38} Cl_2 O_1 P_2 Pd_1 S_1
Molecular Weight	1107.96	938.11
Crystal System	triclinic	monoclinic
T [K]	100(2)	100(2)
Space Group	*P* –1	*P* $2_1/c$
a [Å]	10.9310(2)	12.4622(5)
b [Å]	16.4047(4)	17.3455(6)
c [Å]	16.8778(4)	21.6146(8)
α [°]	113.3040(10)	90
β [°]	93.0200(10)	92.403(3)
γ [°]	103.6170(10)	90
V [Å^3]	2665.19(11)	4668.2(3)
Z	2	4
D_{calc} [g·cm^{-3}]	1.381	1.335
F(0 0 0)	1124	1912
h_{min}, h_{max}	–13, 13	15, 13
k_{min}, k_{max}	–20, 20	21, 17
l_{min}, l_{max}	–21, 20	26, 26
μ [mm^{-1}]	6.789	5.597
Crystal Size [mm]	0.13 x 0.10 x 0.08	0.16 x 0.16 x 0.02
Colour, Shape	clear_light_yellow block	clear_light_yellow plank
R_{int}	0.0822	0.1497
ϑ_{min}, ϑ_{max} [°]	2.889, 80.389	3.268, 74.479
Total Reflections (before merge)	136899	68013
Data (I>3 x sigma(I)) [Reflections, Parameters, Restraints]	11528, 616, 34	9125, 524, 0
S (=GooF)	1.032	1.023
Min. Residual Density [e/Å^3]	–0.828	–0.766
Max. Residual Density [e/Å^3]	1.039	1.257
Threshold Expression	I>2sigma(I)	I>2sigma(I)
R_1	0.0587	0.0997
wR_2	0.1208	0.1377

Figure S1 shows two views each of the fully refined structures of polymorphs 1 and 2 of [(**MotorPhos$_s$**)PdCl$_2$].

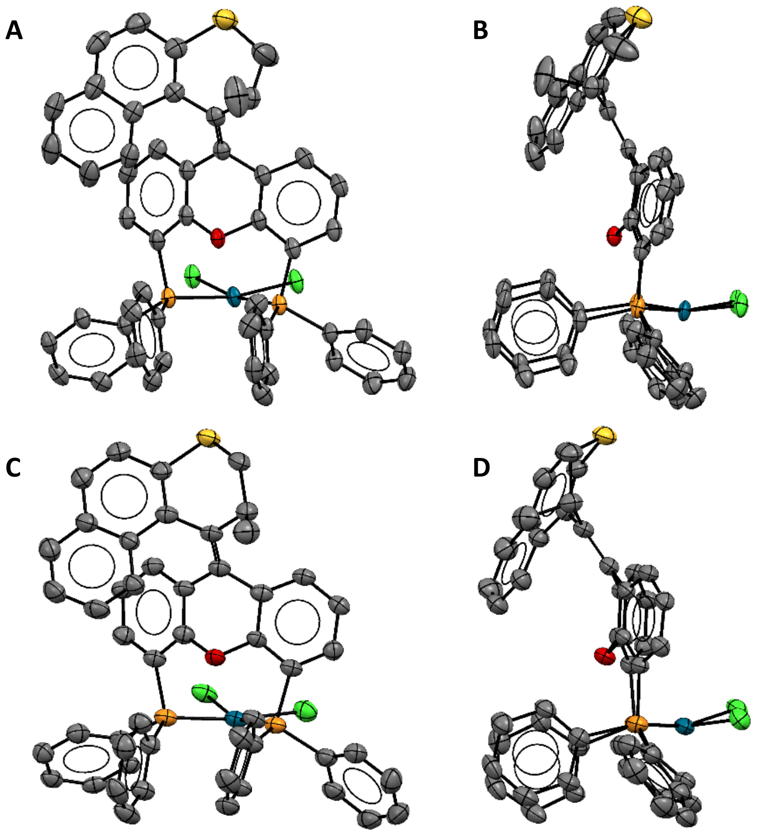

Figure S1. Back and lateral views of polymorphs 1 (**A**, **B**) and 2 (**C**, **D**) of [(**MotorPhos$_s$**)PdCl_2].

Both solid state structures of [(**MotorPhos$_s$**)PdCl_2] are closely related presenting a folded xanthene lower half and an almost perfect square planar coordination geometry around Pd centre formed by the two phosphine groups of **MotorPhos** and the chloride counterions. Their main difference lies in the conformation of the partially saturated ring of the upper half. In both cases, the sulphur atom lies in one plane with the naphthalene backbone whereas the sp^2 carbon forming the double bond with the xanthene half and the remaining sp^3 carbons are found on opposite sides of it. However, in polymorph 1 the sp^3 carbon carrying the methyl substituent is located at a greater distance from this central plane compared to the sp^3 carbon neighbouring the sulphur atom (Figure S1B), with a reversed situation in polymorph 2 (Figure S1D).

4. UV-vis Absorption Studies

In order to measure the absorption spectra of compounds **MotorPhos$_s$** and [(**MotorPhos$_s$**)PdCl_2] $1.5 \cdot 10^{-5}$ M solutions of each compound in degassed DMSO were prepared. Of each sample, a spectrum was recorded at 20 °C and it was subsequently irradiated to its photostationary state (PSS) with a 365 nm LED before a second spectrum was collected. Figure S2 shows a comparison of all four spectra obtained in this way.

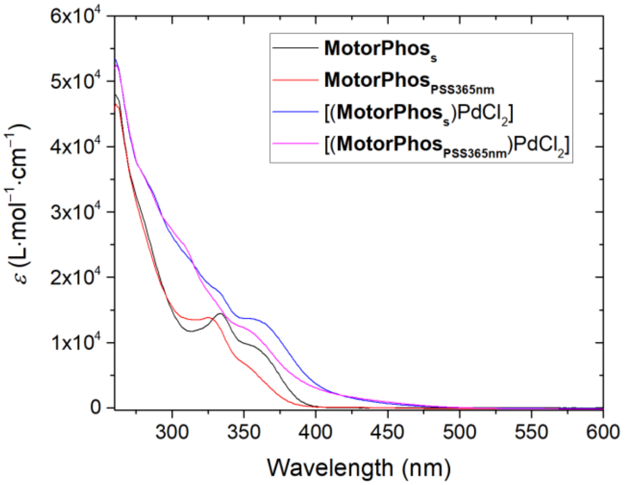

Figure S2. Absorption spectra of stable isomers of **MotorPhos** and [(**MotorPhos**)$PdCl_2$] and after irradiation to PSS with a 365 nm LED. Conditions: DMSO, 20 °C, $1.5 \cdot 10^{-5}$ M.

Figure S3 shows stacks of spectra recorded during irradiation to PSS and subsequent thermal isomerization of samples of **MotorPhos** and [(**MotorPhos**)$PdCl_2$]. The samples were prepared in the same way as for Figure S2.

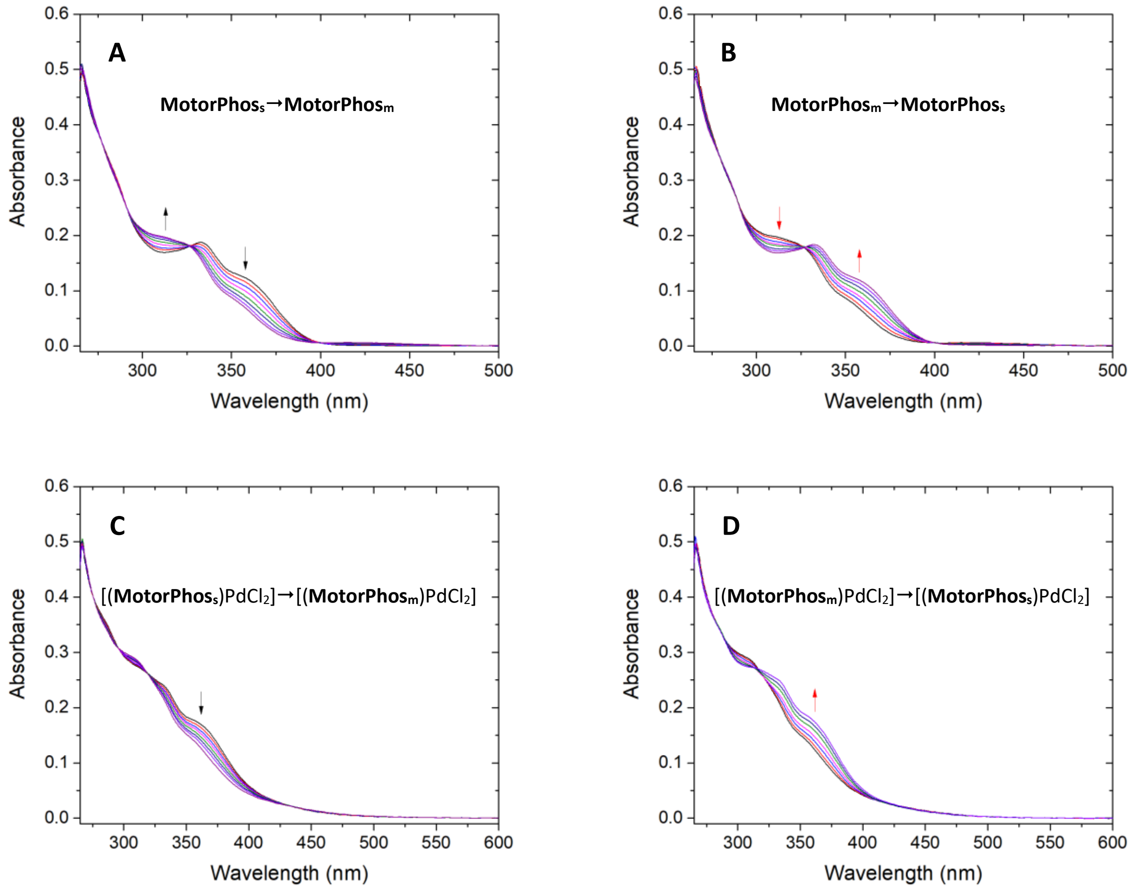

Figure S3. Spectral changes during irradiation to PSS (**A**, **C**) and subsequent thermal isomerization (**B**, **D**) of samples of **MotorPhos** (**A**, **B**) and [(**MotorPhos**)$PdCl_2$] (**C**, **D**) with a 365 nm LED. Conditions: DMSO, 85 °C (**MotorPhos**), 100 °C ([(**MotorPhos**)$PdCl_2$]), $1.5 \cdot 10^{-5}$ M.

Figure S4 shows the results of a fatigue study where samples of **MotorPhos**$_s$ and [(**MotorPhos**$_s$)$PdCl_2$] were 10 times irradiated to PSS at 85 °C and 100 °C, respectively, each time followed by complete thermal helix inversion (THI). The absorbances at 365 nm (**MotorPhos**) and 370 nm ([(**MotorPhos**)$PdCl_2$]) after each step are plotted.

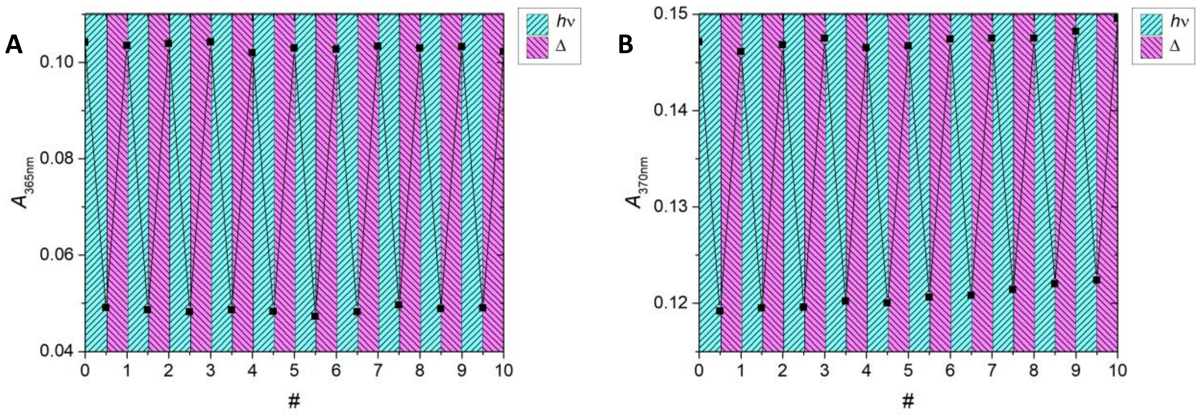

Figure S4. Fatigue studies on **MotorPhos** (**A**) and [(**MotorPhos**)$PdCl_2$] (**B**) where 10 irradiations to PSS with a 365 nm LED were followed by complete THI. Absorbances at 365 nm (**MotorPhos**) and 370 nm ([(**MotorPhos**)$PdCl_2$]) after each step are plotted. Conditions: DMSO, 85 °C (**MotorPhos**), 100 °C ([(**MotorPhos**)$PdCl_2$]), 1.5·10^{-5} M.

Instead of the barrierless *E*/*Z* isomerization commonly observed in molecular motors following photoexcitation, [(**MotorPhos$_s$**)$PdCl_2$] could also perform a THI step after breaking the alkene double bond, where the upper half slides past the lower half in a backwards fashion. To investigate this possibility, we followed the formation of [(**MotorPhos$_m$**)$PdCl_2$] until PSS at two different temperatures, 20 °C and 90 °C (Figure S5). The behaviour at both temperatures is almost identical ruling out an activation barrier in the photoisomerization reaction as would be the case for a THI.

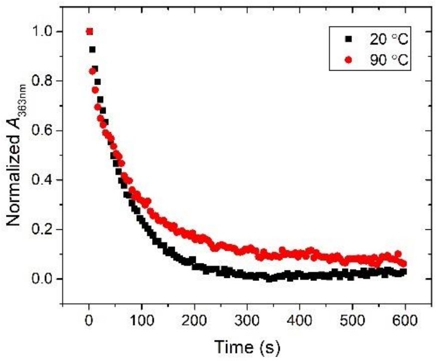

Figure S5. Kinetic plots for formation of PSS from [(**MotorPhos$_s$**)$PdCl_2$]. The normalized absorbance at 363 nm is plotted over the course of an irradiation with a 365 nm LED. Conditions: DMSO, 20 °C and 90 °C, 1.5·10^{-5} M.

5. NMR Studies

5.1. ROESY NMR Studies

ROESY spectra of compound [(**MotorPhos**)$PdCl_2$] before and after 2.5 h irradiation to PSS with a 365 nm LED were recorded in degassed DMSO-d_6 to assign alkyl proton signals of the stable and metastable isomer (Figure S6 and S7).

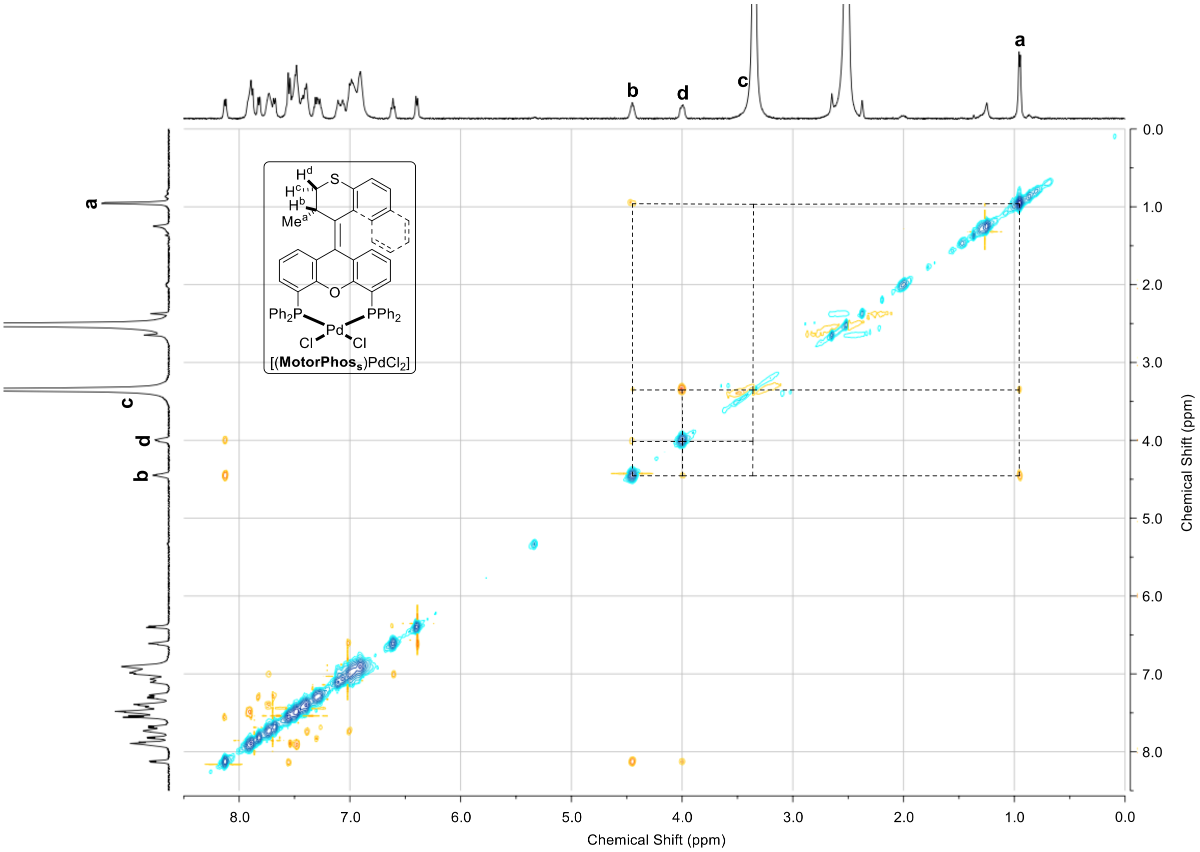

Figure S6. ROESY NMR spectrum of [(**MotorPhos$_s$**)$PdCl_2$] with notable cross peaks supporting the assignment of the labelled protons highlighted. Conditions: DMSO-d_6, 25 °C, $2.5 \cdot 10^{-3}$ M.

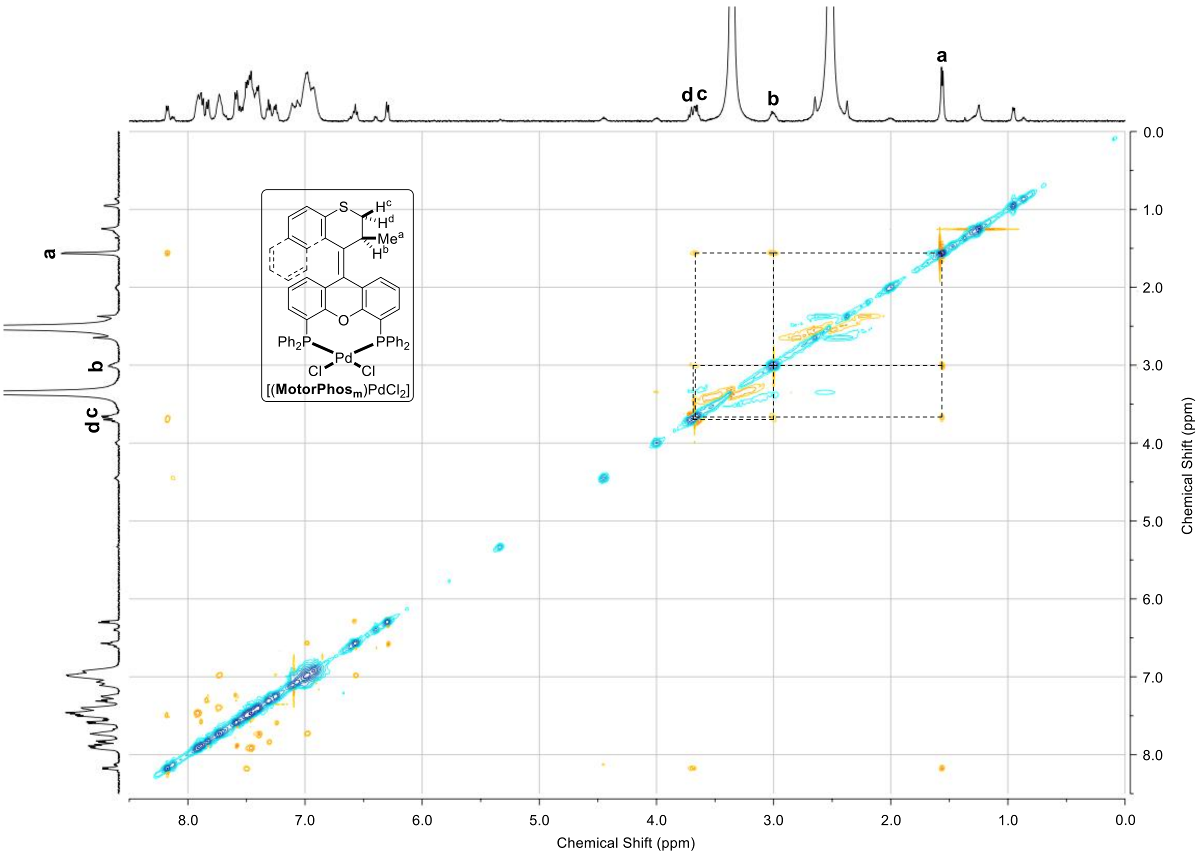

Figure S7. ROESY NMR spectrum of the PSS mixture between [(**MotorPhos$_m$**)PdCl$_2$] and [(**MotorPhos$_s$**)PdCl$_2$] obtained after irradiation of a sample of [(**MotorPhos$_s$**)PdCl$_2$] with a 365 nm light LED for 2.5 h. Notable cross peaks supporting the assignment of the labelled protons of [(**MotorPhos$_m$**)PdCl$_2$] are highlighted. Conditions: DMSO-*d$_6$*, 25 °C, 2.5·10^{-3} M (overall).

5.2. Following 180° Rotations by ^{1}H NMR

In order to follow the two subsequent steps of photochemical *E*/*Z* isomerization and THI by ^{1}H NMR, 500 µL of 2.5·10^{-3} M solutions of **MotorPhos$_s$** and [(**MotorPhos$_s$**)PdCl$_2$] in degassed DMSO-*d$_6$* were prepared. These samples were transferred into NMR tubes which were fitted with a glass fibre cable for *in situ irradiation* using a 365 nm LED. The samples of **MotorPhos$_s$** and [(**MotorPhos$_s$**)PdCl$_2$] were heated at 60 °C and 80 °C, respectively, inside the probe of a Varian Unity Plus 500 NMR spectrometer to increase the speed of THI. Quantitative ^{1}H NMR spectra were obtained before irradiation, after irradiation to the respective PSS and after completed THI following disconnection of the LED. Stacks comparing the spectra of **MotorPhos** and [(**MotorPhos**)PdCl$_2$] at these three stages in the rotation are shown in Figures S8 and S9, respectively. For [(**MotorPhos**)PdCl$_2$] ^{31}P NMR spectra were also recorded at each stage (Figure S10).

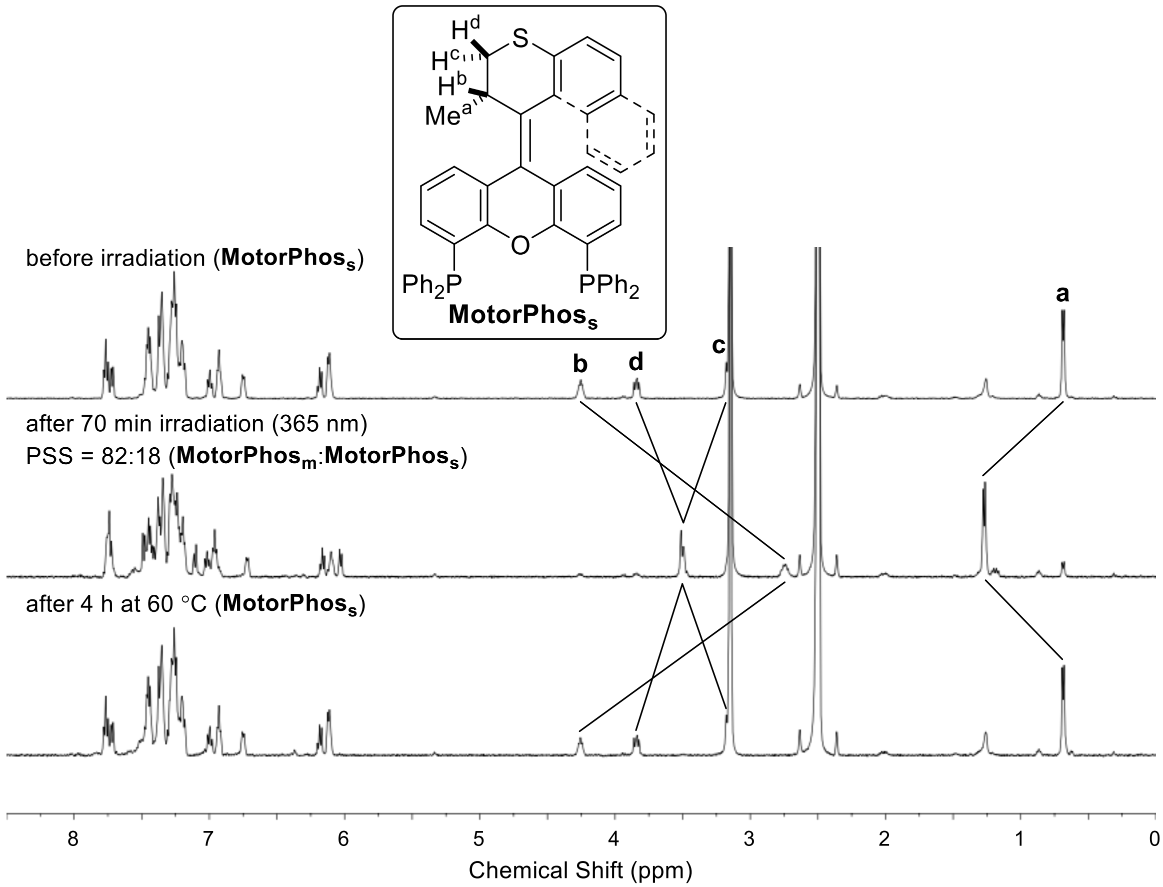

Figure S8. Stack of ^{1}H NMR spectra of **MotorPhos** obtained before irradiation with a 365 nm LED (**MotorPhos$_s$**), after irradiation to PSS and after completed subsequent THI. Conditions: DMSO-d_6, 60 °C, $5.0 \cdot 10^{-3}$ M.

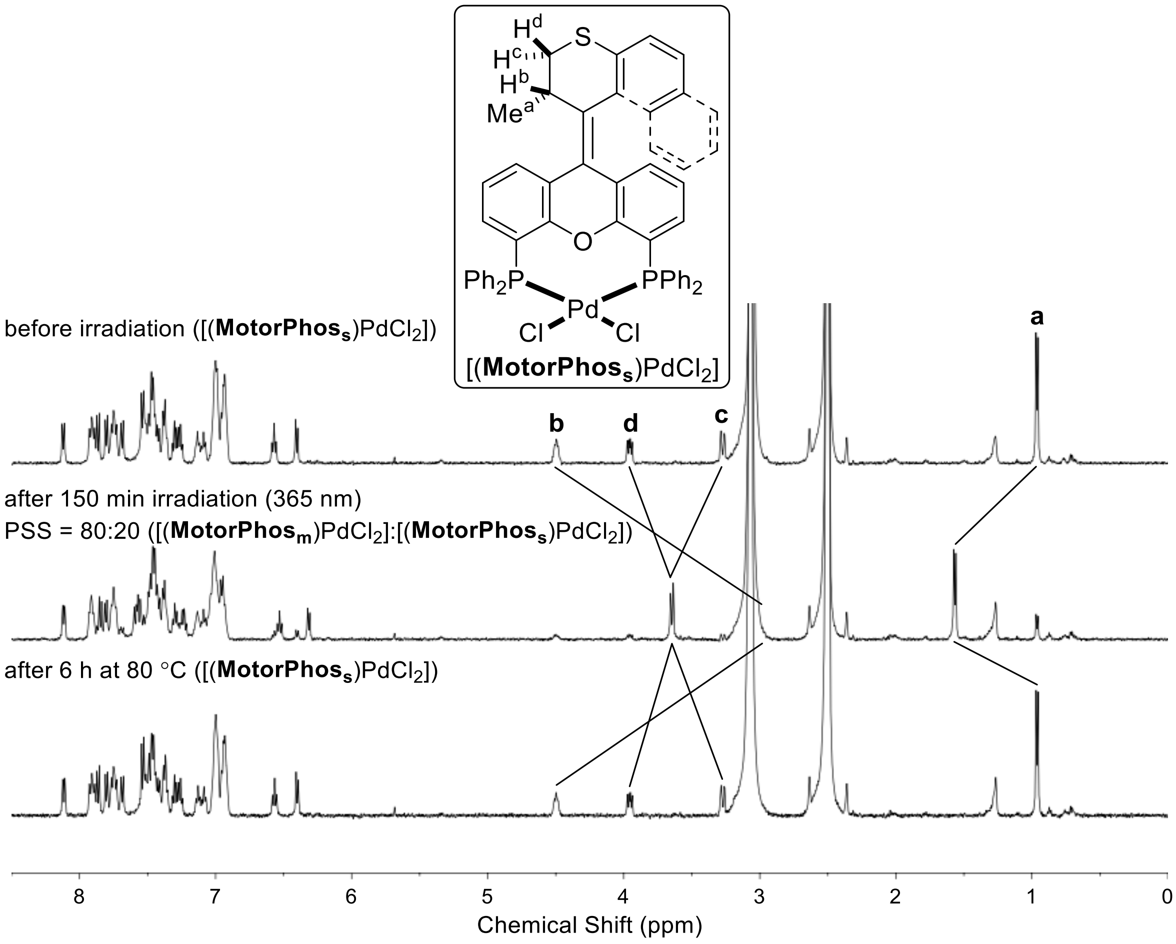

Figure S9. Stack of ^{1}H NMR spectra of [(**MotorPhos**)PdCl$_2$] obtained before irradiation with a 365 nm LED ([(**MotorPhos$_s$**)PdCl$_2$]), after irradiation to PSS and after completed subsequent THI. Conditions: DMSO-d_6, 80 °C, $5.0\cdot10^{-3}$ M.

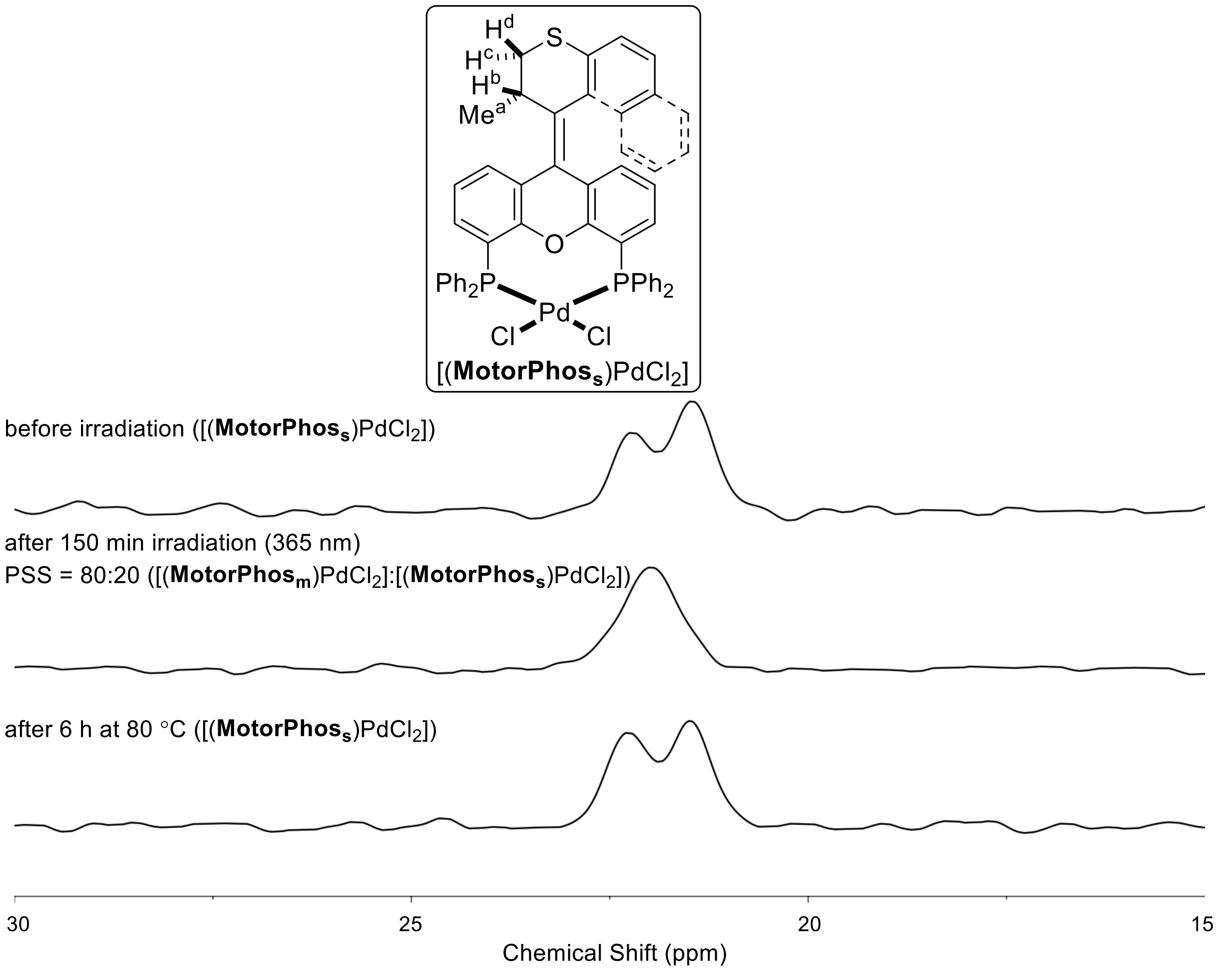

Figure S10. Stack of ^{31}P NMR spectra of [(**MotorPhos**)$PdCl_2$] obtained before irradiation with a 365 nm LED ([(**MotorPhos**$_s$)$PdCl_2$]), after irradiation to PSS and after completed subsequent THI. Conditions: DMSO-d_6, 80 °C, $5.0 \cdot 10^{-3}$ M.

5.3. Determination of PSS Compositions

To study the ratios of metastable:stable isomers after irradiation to PSS using LEDs of different wavelengths $2.5 \cdot 10^{-3}$ M solutions of **MotorPhos**$_s$ and [(**MotorPhos**$_s$)$PdCl_2$] in degassed DMSO-d_6 were prepared. For each experiment 500 µL aliquots were transferred into an NMR tube which was fitted with a glass fibre cable for *in situ* irradiation using LEDs of 365 nm, 455 nm, 470 nm and 490 nm. The samples were placed inside the probe of a Varian Unity Plus 500 NMR spectrometer which was kept at 25 °C for the duration of each experiment. Each sample was then irradiated for 1 h (**MotorPhos**$_s$) or 4 h ([(**MotorPhos**$_s$)$PdCl_2$]) using one of these LEDs to reach PSS and quantitative ^1H NMR spectra were recorded to determine the ratios of metastable:stable isomers. Table S2 contains a summary of these ratios and Figure S11 shows ^1H NMR spectra of PSS mixtures of [(**MotorPhos**)$PdCl_2$] obtained after irradiation with 455 nm and 470 nm LEDs.

Table S2. Summary of ratios of metastable:stable isomers of **MotorPhos** and [(**MotorPhos**)$PdCl_2$] at 25 °C after irradiation to PSS with LEDs of different wavelengths.

λ_{exc} (nm)	**MotorPhos$_m$:MotorPhos$_s$**	**[(MotorPhos$_m$)$PdCl_2$]: [(MotorPhos$_s$)$PdCl_2$]**
365	91:9	88:12
455	0:100	80:20
470	n.d.	73:27
490	n.d.	0:100

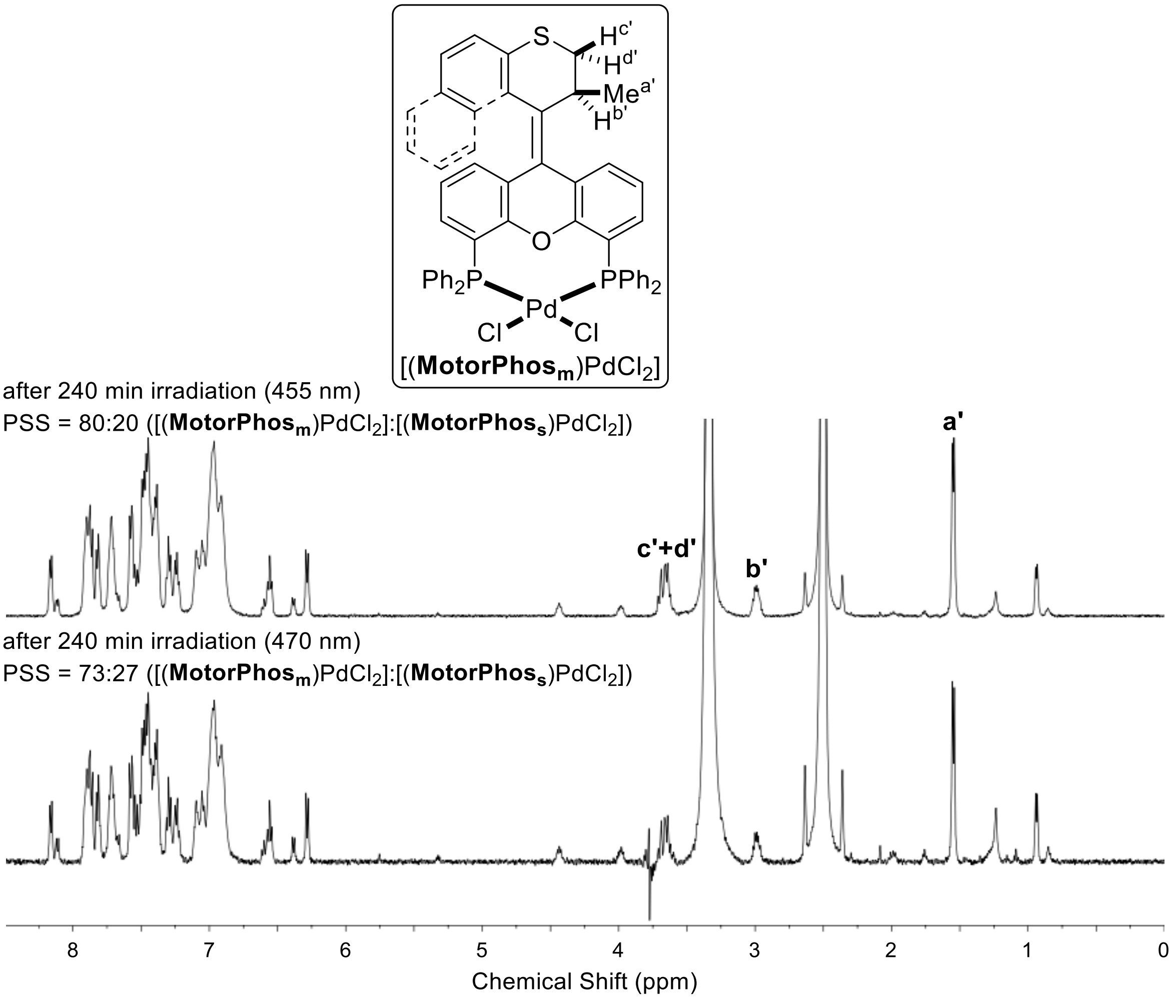

Figure S11. ^1H NMR spectra of PSS mixtures of [(**MotorPhos**)$PdCl_2$] obtained after irradiation with 455 nm and 470 nm LEDs. Conditions: DMSO-d_6, 25 °C, $2.5 \cdot 10^{-3}$ M.

5.4. Eyring Analysis of THIs of MotorPhos$_m$ and [(MotorPhos$_m$)$PdCl_2$]

To determine the Gibbs free energy of activation, $\Delta^{\ddagger}G$, for the THI of **MotorPhos$_m$** and [(**MotorPhos$_m$**)$PdCl_2$] Eyring analysis was performed. To this end, $2.5 \cdot 10^{-3}$ M stock solutions of **MotorPhos$_s$** and [(**MotorPhos$_s$**)$PdCl_2$] in degassed DMSO-d_6 were prepared of which 500 µL aliquots were used for each measurement. These were transferred into an NMR tube which was subsequently equipped

with a glass fibre cable for *in situ* irradiation. They were then heated at the indicated temperature inside the NMR probe before being irradiated to PSS using a 365 nm LED. A quantitative ^{1}H NMR spectrum was recorded, the LED was removed and quantitative ^{1}H NMR spectra were recorded at regular intervals over the course of the recovery of the according stable isomer. The percentage of metastable isomer was then plotted over the time since the removal of the light source (Figure S12) from which rate constants, *k*, could be obtained by fitting a 1st order rate law.

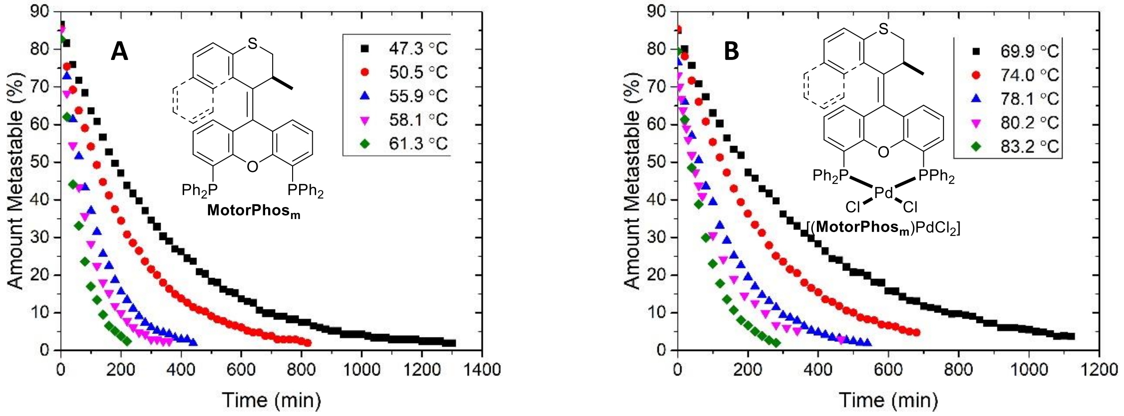

Figure S12. Temperature dependent decays of **MotorPhos**$_m$ (**A**) and [(**MotorPhos**$_m$)$PdCl_2$] (**B**) over time after irradiation to PSS using a 365 nm LED. Conditions: DMSO-*d*$_6$, $2.5{\cdot}10^{-3}$ M.

Activation parameters for THI of **MotorPhos**$_m$ and [(**MotorPhos**$_m$)$PdCl_2$] were obtained by fitting the linearized form of the Eyring equation (Figure S13, Table S3).

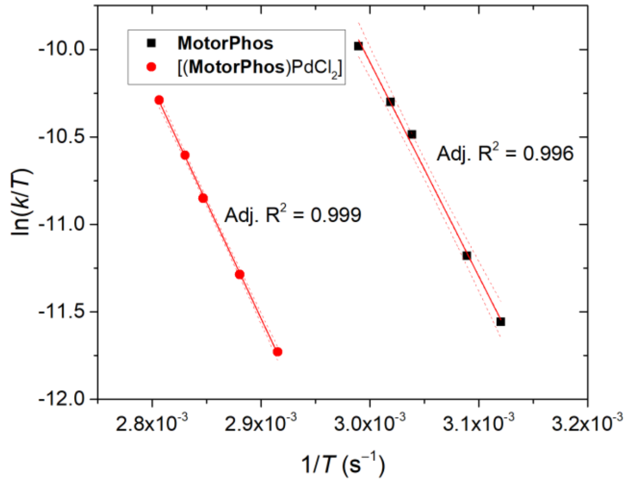

Figure S13. Linearized Eyring plots for the THI of **MotorPhos**$_m$ (black squares) and [(**MotorPhos**$_m$)$PdCl_2$] (red circles) based on data in Figure S12. Dashed lines represent 95% confidence intervals.

Table S3. Summary of activation parameters obtained from Eyring analysis of the THI of metastable isomers **MotorPhos$_m$** and [(**MotorPhos$_m$**)PdCl$_2$].

	$\Delta^‡G$(20 °C) (kJ·mol^{-1})	$\Delta^‡H$(20 °C) (kJ·mol^{-1})	$\Delta^‡S$(20 °C) (J·mol^{-1}·K^{-1})	$t_{1/2}$ (min)
**MotorPhos$_m$**	95.3±1.1	109.4	48.2	181±11
[(**MotorPhos$_m$**)PdCl$_2$]	103.1±1.0	113.5	35.7	4477±515

6. Computational Analysis

All calculations on [(**MotorPhos**)PdCl$_2$] and its PH$_2$ analogue were carried out using the Gaussian 16 Rev. B.01 software package.[9] Input files were prepared and output files analysed using GaussView 5.[10] Structures were optimized at the ωB97X-D/C,H,O:6-31G(d,p), Cl,P,S:6-311G(2d,p), Pd:SDD level of theory. Initially, the thermal isomerization mechanism was studied on the PH$_2$ analogue of [(**MotorPhos**)PdCl$_2$] to reduce computational costs. Afterwards, key structures were also optimized with the Ph substituents present on the phosphines to demonstrate the validity of the results obtained for the di-PH$_2$ system. All optimized structures were confirmed to be stationary points by the number of imaginary frequencies (0 for minima, 1 for transition states). A summary of the individual structures' energies is provided in Table S4. Labelling of intermediate structures according to our earlier publication.[11]

Table S4. Summary of the energies of the PH$_2$ and PPh$_2$ structures of [(**MotorPhos**)PdCl$_2$] (ωB97X-D/C,H,O:6-31G(d,p), Cl,P,S:6-311G(2d,p), Pd:SDD). a=*anti*-folded, at=*anti*-folded twisted, st=*syn*-folded twisted, [eq]=Me equatorial, [ax]=Me axial, i=inside, c=central, e=outside.

Phosphine	Structure	Number of Imaginary Frequencies	Sum of electronic and thermal free energies (Hartree)	Energy relative to global minimum (kJ·mol^{-1})
**PH$_2$**	a-metastable-[eq]-i	0	–3246.026041	22.29
	a-metastable-[eq]-c	0	–3246.020326	37.15
	a-metastable-[eq]-e	0	–3246.015182	50.52
	TS-metastable-i-to-c	1	–3246.017805	43.70
	TS-metastable-c-to-e	1	–3246.011913	59.02
	TS1-i	1	–3246.010558	62.54
	TS1-c	1	–3246.004474	78.36
	TS1-e	1	–3246.000939	87.55
	at-I1-[ax]-i	0	–3246.018306	42.40
	at-I1-[ax]-c	0	–3246.011891	59.08
	at-I1-[ax]-e	0	–3246.008154	68.79
	TS-I1-i-to-c	1	–3246.009629	64.96
	TS-I1-c-to-e	1	–3246.003946	79.73
	TS2-i	1	–3245.969106	170.32
	TS2-c	1	–3245.971597	163.84
	TS2-e	1	–3245.965333	180.13
	st-I2-[ax]-i	0	–3246.029263	13.91
	st-I2-[ax]-c	0	–3246.022692	30.99
	st-I2-[ax]-e	0	–3246.015680	49.23
	TS-I2-i-to-c	1	–3246.019983	38.04
	TS-I2-c-to-e	1	–3246.013022	56.14

	TS3-i-to-e	1	–3246.010539	62.59
	TS3-c-to-c	1	–3246.006158	73.98
	TS3-e-to-i	1	–3246.009379	65.61
	TS4-i-to-e	1	–3245.999333	91.73
	TS4-c-to-c	1	–3245.999809	90.49
	TS4-e-to-i	1	–3246.005699	75.18
	st-I3-[ax]-i	0	–3246.017708	43.95
	st-I3-[ax]-c	0	–3246.011041	61.29
	st-I3-[ax]-e	0	–3246.004102	79.33
	TS-I3-i-to-c	1	–3246.008849	66.99
	TS-I3-c-to-e	1	–3246.001445	86.24
	TS5-i	1	–3246.014511	52.27
	TS5-c	1	–3246.007701	69.97
	TS5-e	1	–3246.000614	88.40
	st-I4-[eq]-i	0	–3246.019071	40.41
	st-I4-[eq]-c	0	–3246.012727	56.90
	st-I4-[eq]-e	0	–3246.005710	75.15
	TS-I4-i-to-c	1	–3246.010752	62.04
	TS-I4-c-to-e	1	–3246.003358	81.26
	TS6-i	1	–3245.977960	147.30
	TS6-c	1	–3245.972254	162.13
	TS6-e	1	–3245.965277	180.27
	a-stable-[ax]-i	0	–3246.034613	0
	a-stable-[ax]-c	0	–3246.0.28779	15.17
	a-stable-[ax]-e	0	–3246.023424	29.09
	TS-stable-i-to-c	1	–3246.025930	22.58
	TS-stable-c-to-e	1	–3246.020020	37.94
**PPh_2**	st-I3-[ax]-c	0	–4169.645519	61.62
	TS6-c	1	–4169.610415	152.89
	a-stable-[ax]-c	0	–4169.664195	13.07
	a-stable-[ax]-i	0	–4169.669220	0

Figure S14 summarizes the two pathways for thermal isomerization of the PH_2 analogue of [(**MotorPhos**)$PdCl_2$].

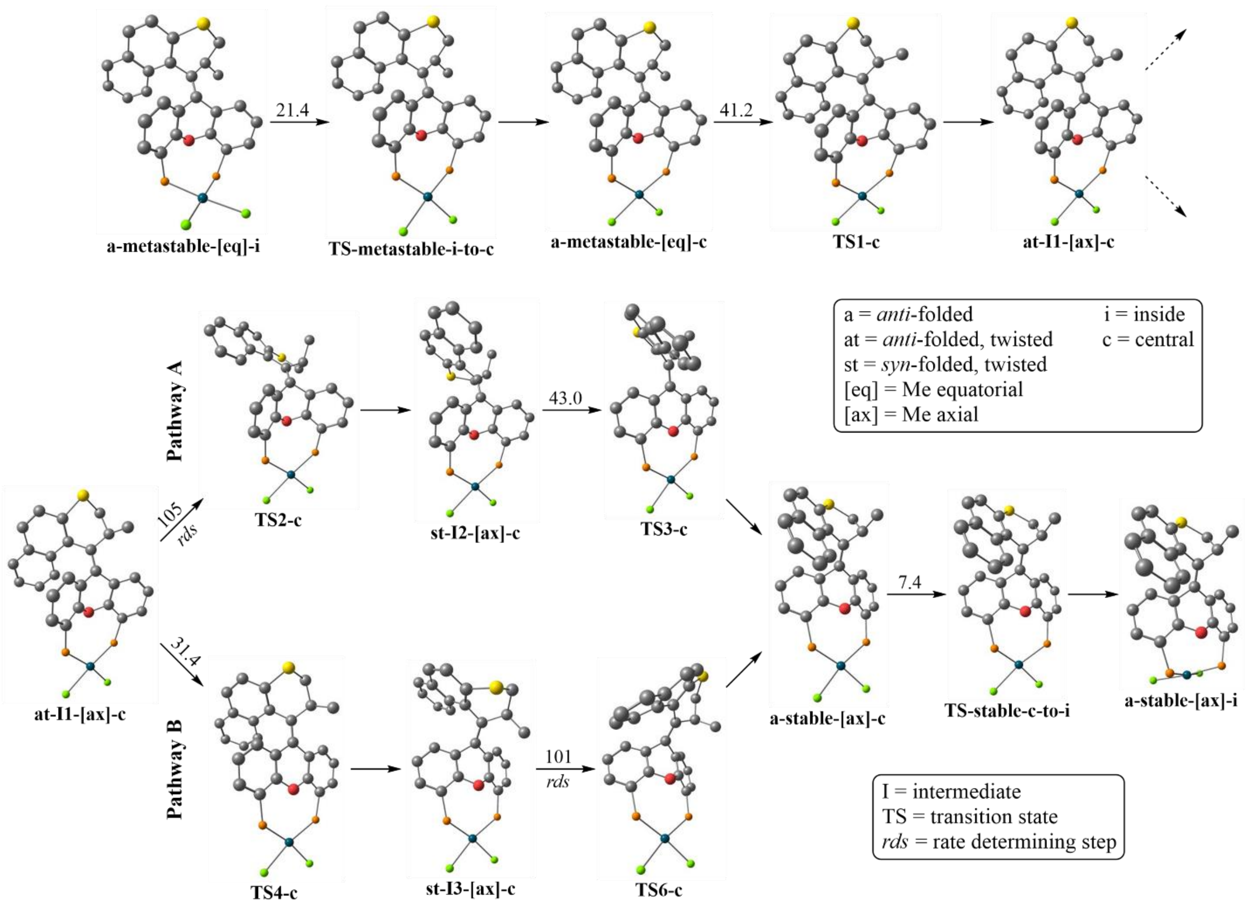

Figure S14. Thermal isomerization pathways with the lowest barriers for the rate determining step leading from the unstable isomer obtained after photoisomerization (a-unstable-[eq]-i) to the global minimum stable isomer (a-stable-[ax]-i). The PPh_2 groups of [(**MotorPhos**)$PdCl_2$] were replaced with PH_2 to reduce computational costs (ωB97X-D/C,H,O:6-31G(d,p), Cl,P,S:6-311G(2d,p), Pd:SDD). Activation barriers for each step are given in kJ·mol^{-1}. Hydrogens were omitted for clarity. Pathways **A** and **B** differ in the order of steps; in **A** sliding of the naphthalene unit over the lower half is followed by a ring flip in the lower half, with **B** following the reverse order.

Figure S15A shows how all the structures calculated for the PH_2 analogue of [(**MotorPhos**)$PdCl_2$] are connected visualizing the different pathways one can follow for the rotation and oscillation motions. Figure 15B and Figure 15C show the structures along two isomerization pathways describing either an oscillation (Figure 15B) or rotation movement (Figure 15C). XYZ files for all structure are provided separately.

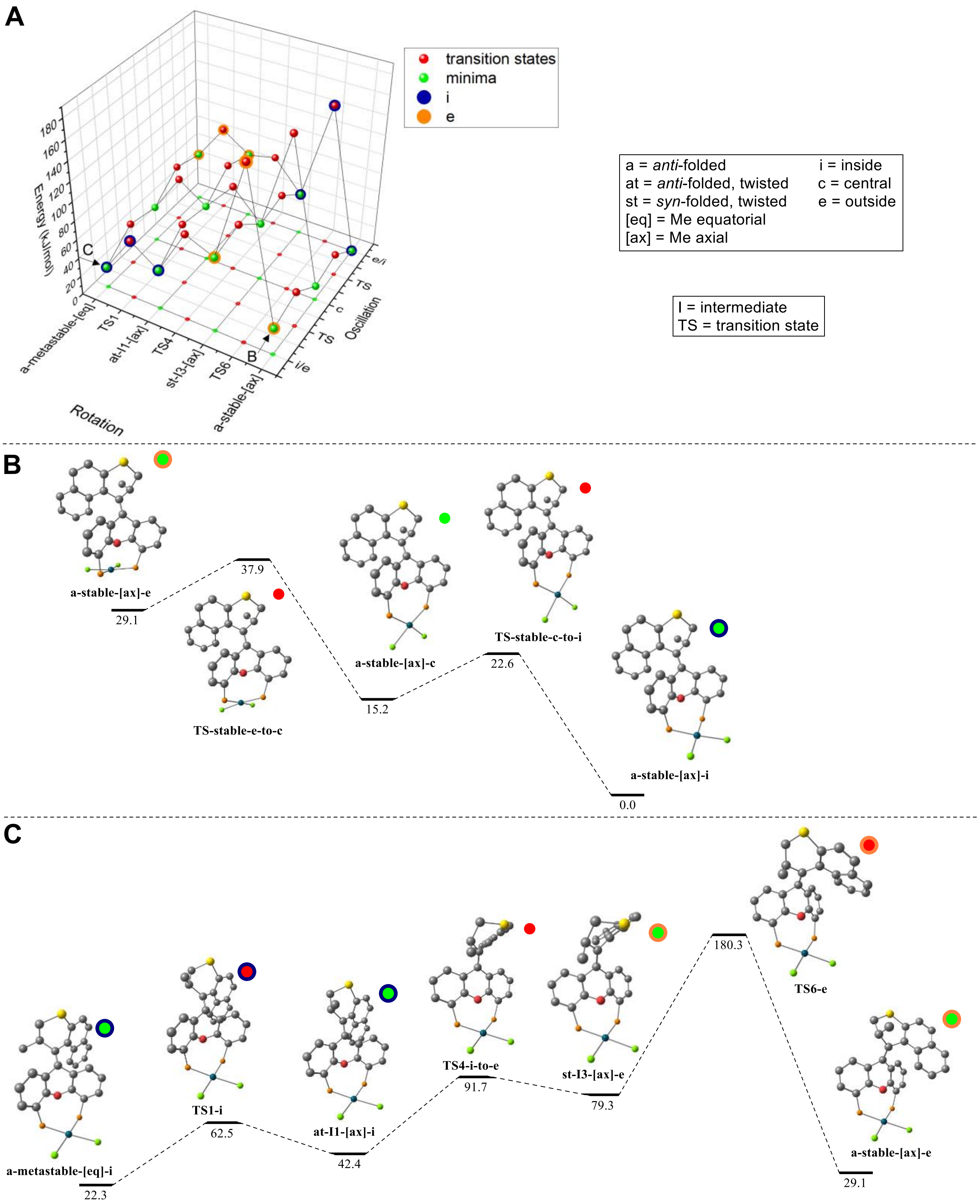

Figure S15. A Plot showing the different pathways for rotation and oscillation of the PH_2 analogue of [(**MotorPhos**)$PdCl_2$]. **B** Houk plot of an example of a pathway following the oscillation axis in **A**. The energies of the structures shown are reported in kJ·mol^{-1}. Hydrogens were omitted for clarity. **C** Houk plot of an example of a pathway following the rotation axis in **A**. The energies of the structures shown are reported in kJ·mol^{-1}. Hydrogens were omitted for clarity.

To compare the relative stabilities of the three possible conformers with regard to the $PdCl_2$ group of the stable and metastable isomers of [(**MotorPhos**)$PdCl_2$] in DMSO, these six structures were optimized by DFT at the ωB97X-D/C,H,O:6-31G(d,p), Cl,P,S:6-311G(2d,p), Pd:SDD level of theory using the SMD solvent model for DMSO. The final geometries are shown in Figure S16 and the energies are summarized in Table S5.

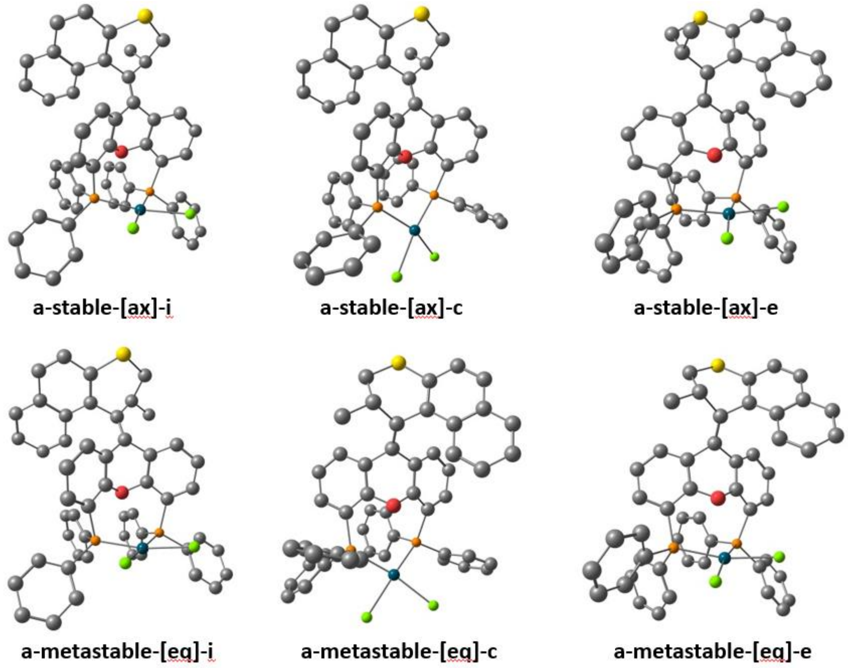

Figure S16. DFT-optimized (ωB97X-D/C,H,O:6-31G(d,p), Cl,P,S:6-311G(2d,p), Pd:SDD, DMSO (SMD)) geometries of stable and metastable isomers of [(**MotorPhos**)$PdCl_2$] taking into account different orientations of the $PdCl_2$ moiety.

Table S5. Summary of the energies of the three conformers of the stable and metastable isomers of [(**MotorPhos**)$PdCl_2$] shown in Figure S16 (ωB97X-D/C,H,O:6-31G(d,p), Cl,P,S:6-311G(2d,p), Pd:SDD, DMSO (SMD)). a=*anti*-folded, at=*anti*-folded twisted, st=*syn*-folded twisted, [eq]=Me equatorial, [ax]=Me axial, i=inside, c=central, e=outside.

Structure	*Sum of electronic and thermal free energies (Hartree)*	*Energy relative to global minimum (kJ·mol^{-1})*
a-stable-[ax]-i	-4169.735448	0
a-stable-[ax]-c	-4169.725504	25.85
a-stable-[ax]-e	-4169.729721	14.89
a-metastable-[eq]-i	-4169.727617	20.36
a-metastable-[eq]-c	-4169.719377	41.78
a-metastable-[eq]-e	-4169.721930	35.15

We also determined the UV-vis absorption and ECD spectra of the stable and metastable isomer of [(**MotorPhos**)$PdCl_2$] by TD-DFT analysis (30 states) at the ωB97X-D/C,H,O:6-31G(d,p), Cl,P,S:6-311G(2d,p), Pd:SDD level of theory using the SMD solvent model for DMSO. A comparison of the obtained UV-vis

absorption spectra with experimental results is provided in Figure S17, whereas the calculated ECD spectra are shown on Figure S18.

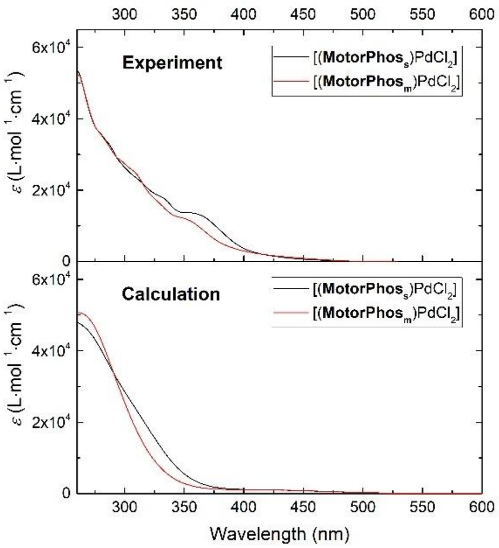

Figure S17. Comparison between the experimental (top) and calculated (bottom) UV-vis absorption spectra of [(**MotorPhos$_s$**)PdCl$_2$] and [(**MotorPhos$_m$**)PdCl$_2$]. Experimental conditions: DMSO, 20 °C, 1.5·10^{-5} M. Calculations: TD-DFT, ωB97X-D/C,H,O:6-31G(d,p), Cl,P,S:6-311G(2d,p), Pd:SDD, DMSO (SMD), 30 states.

The UV-vis spectrum of the pure metastable isomer in Figure S17 was calculated from the data presented in Figure S2 using the ratio of metastable:stable isomer in Table S2. Experimental and calculated spectra are in good agreement, both showing a redshift in absorption upon formation of the metastable isomer and a long weakly absorbing tail extending into the low-energy region.

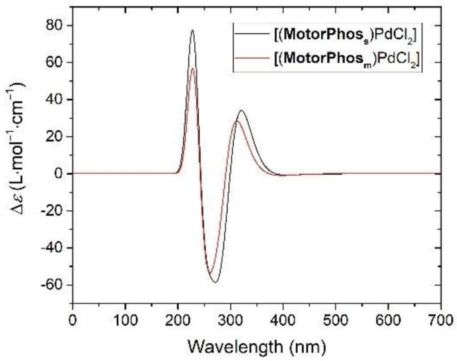

Figure S18. Calculated ECD spectra of [(**MotorPhos$_s$**)PdCl$_2$] and [(**MotorPhos$_m$**)PdCl$_2$]. Calculations: TD-DFT, ωB97X-D/C,H,O:6-31G(d,p), Cl,P,S:6-311G(2d,p), Pd:SDD, DMSO (SMD), 30 states.

7. References

[1] Kistemaker, J. C. M.; Pizzolato, S. F.; van Leeuwen, T.; Pijper, T. C.; Feringa, B. L. *Chem. Eur. J.* **2016**, *22*, 13478–13487.

[2] Reese, C. B.; Song, Q.; Yan, H. *Tetrahedron Lett.* **2001**, *42*, 1789–1791.

[3] Odrowaz-Sypniewski, M. R.; Tsoungas, P. G.; Varvounis, G.; Cordopatis, P.; *Tetrahedron Lett.* **2009**, *50*, 5981–5983.

[4] Bruker, APEX3 (V2019.1-0), SAINT (Version 8.40A) and SADABS (Version 2016/1). Bruker AXS Inc., Madison, Wisconsin, USA.

[5] Krause, L.; Herbst-Irmer, R.; Sheldrick, G. M.; Stalke, D. Comparison of silver and molybdenum microfocus X-ray sources for single-crystal structure determination. *J. Appl. Crystallogr.* **2015**, *48*, 3–10.

[6] Sheldrick, G. M. *SHELXT* – Integrated space-group and crystal-structure determination. *Acta Crystallogr. Sect. A Found. Adv.* **2015**, *71*, 3–8.

[7] Sheldrick, G. M. A short history of SHELX. *Acta Crystallogr Sect. A* **2008**, *A64*, 112–122.

[8] Spek, A. L. *PLATON* SQUEEZE: a tool for the calculation of the disordered solvent contribution to the calculated structure factors. *Acta Crystallogr. Sect. C Struct. Chem.* **2015**, *71*, 9–18.

[9] Gaussian 16, Revision B.01, Frisch, M. J.; Trucks, G. W.; Schlegel, H. B.; Scuseria, G. E.; Robb, M. A.; Cheeseman, J. R.; Scalmani, G.; Barone, V.; Petersson, G. A.; Nakatsuji, H.; Li, X.; Caricato, M.; Marenich, A. V.; Bloino, J.; Janesko, B. G.; Gomperts, R.; Mennucci, B.; Hratchian, H. P.; Ortiz, J. V.; Izmaylov, A. F.; Sonnenberg, J. L.; Williams-Young, D.; Ding, F.; Lipparini, F.; Egidi, F.; Goings, J.; Peng, B.; Petrone, A.; Henderson, T.; Ranasinghe, D.; Zakrzewski, V. G.; Gao, J.; Rega, N.; Zheng, G.; Liang, W.; Hada, M.; Ehara, M.; Toyota, K.; Fukuda, R.; Hasegawa, J.; Ishida, M.; Nakajima, T.; Honda, Y.; Kitao, O.; Nakai, H.; Vreven, T.; Throssell, K.; Montgomery, J. A., Jr.; Peralta, J. E.; Ogliaro, F.; Bearpark, M. J.; Heyd, J. J.; Brothers, E. N.; Kudin, K. N.; Staroverov, V. N.; Keith, T. A.; Kobayashi, R.; Normand, J.; Raghavachari, K.; Rendell, A. P.; Burant, J. C.; Iyengar, S. S.; Tomasi, J.; Cossi, M.; Millam, J. M.; Klene, M.; Adamo, C.; Cammi, R.; Ochterski, J. W.; Martin, R. L.; Morokuma, K.; Farkas, O.; Foresman, J. B.; Fox, D. J. Gaussian, Inc., Wallingford CT, 2016.

[10] Gauss View (Version 5), Dennington, R.; Keith, T.; Millam, J. Semichem Inc., Shawnee Mission, 2009.

[11] Cnossen, A.; Kistemaker, J. C. M.; Kojima, T.; Feringa, B. L. Structural Dynamics of Overcrowded Alkene-Based Molecular Motors during Thermal Isomerization. *J. Org. Chem.* **2014**, *79*, 927–935.

8. NMR Spectra

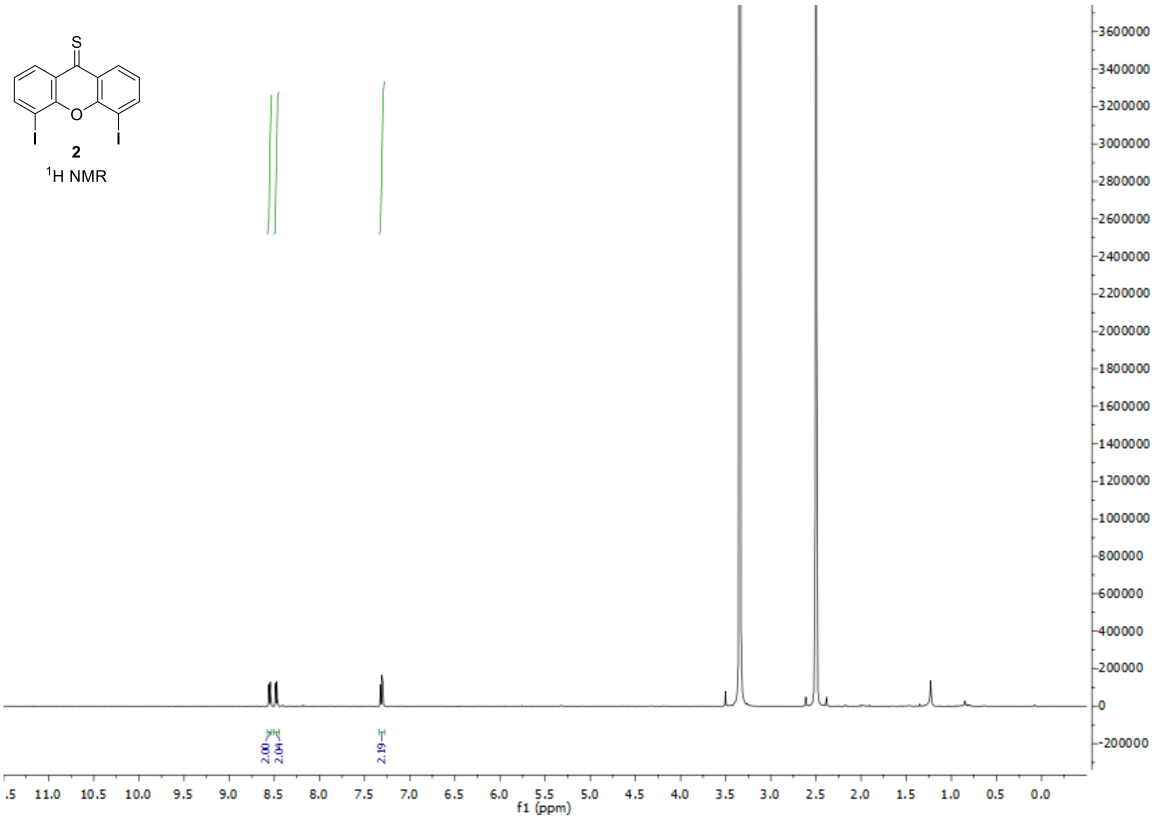

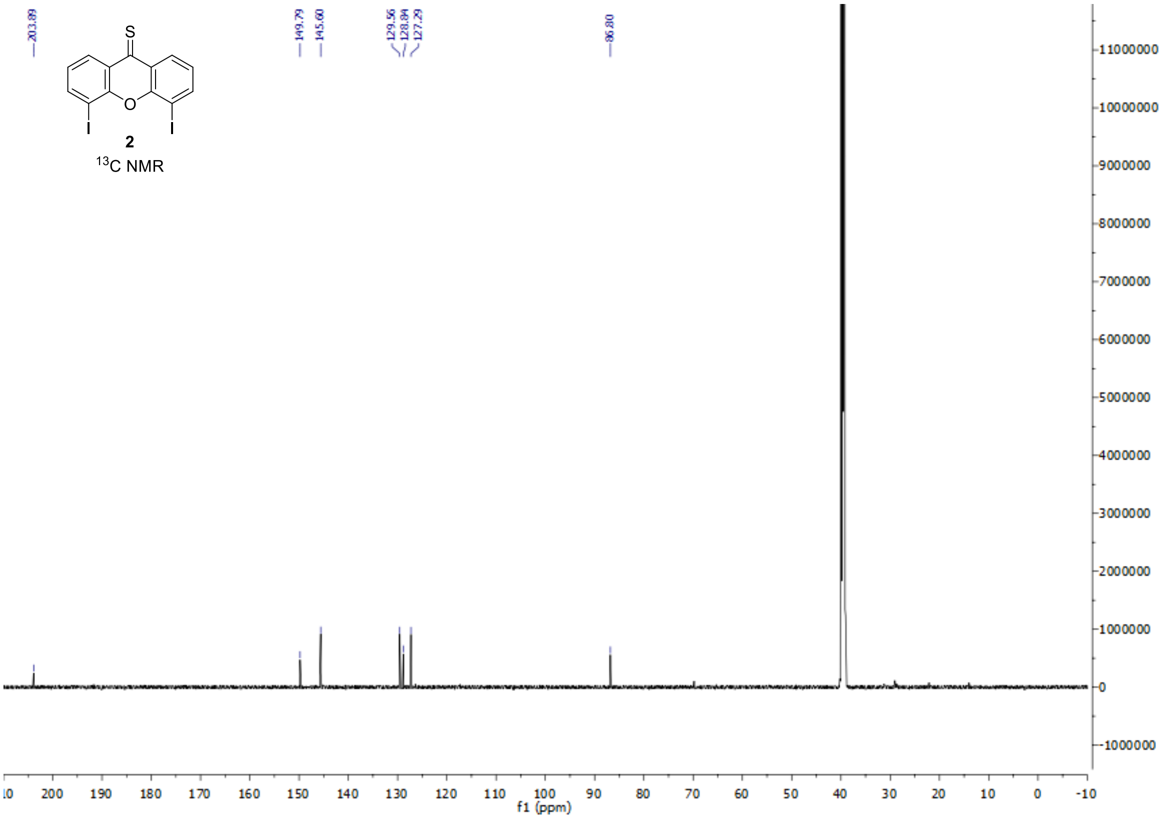

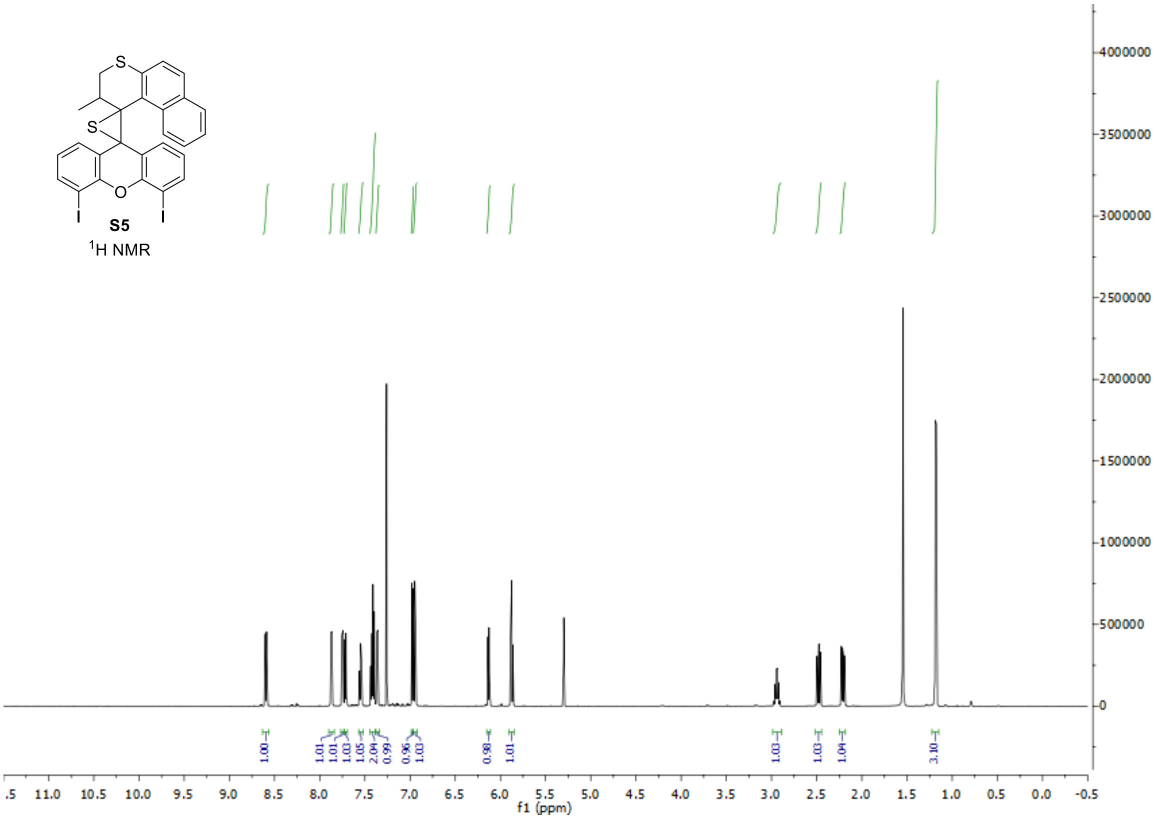
S
S
O
I
I
S5
^{1}H NMR
1.00
1.01
1.01
1.03
1.05
2.04
0.99
0.96
1.03
0.98
1.01
1.03
1.03
1.04
3.10
f1 (ppm)

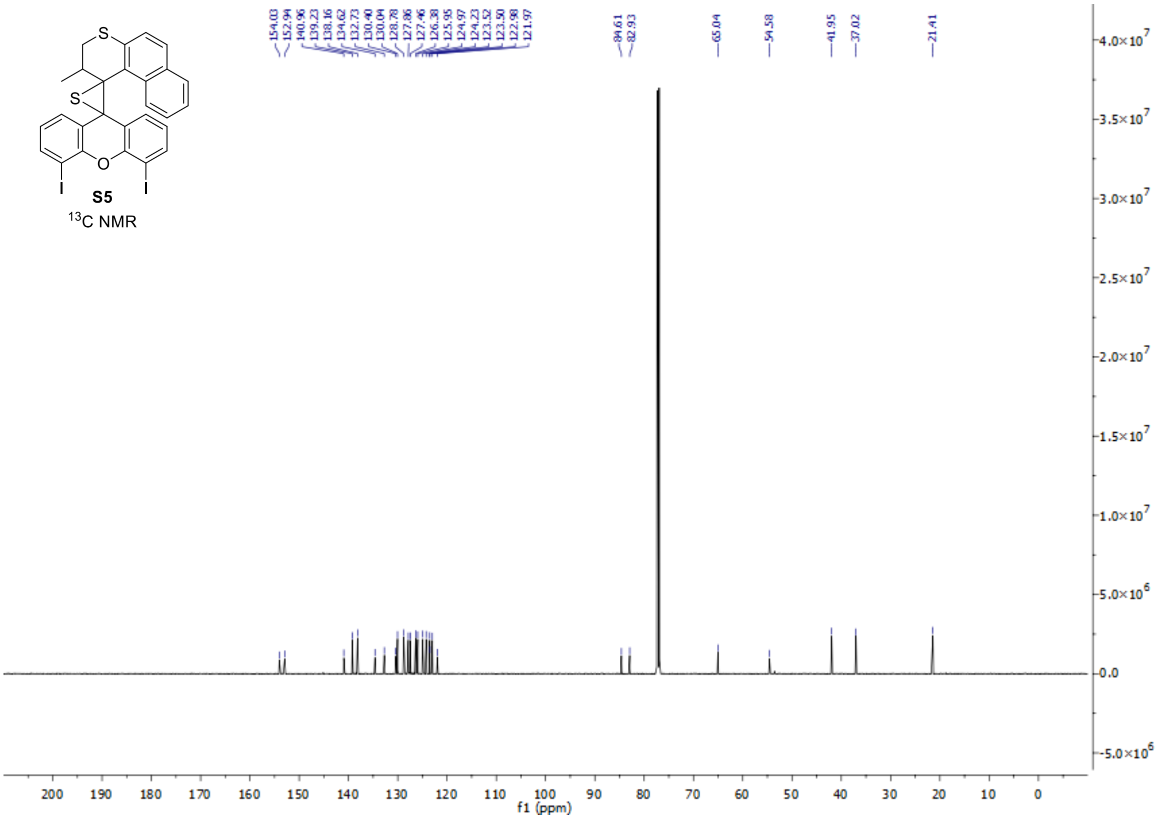
S
S
O
I
I
S5
^{13}C NMR
154.03
152.94
140.96
139.23
138.16
134.62
132.73
130.40
130.04
128.78
127.86
127.46
126.38
125.95
124.97
124.23
123.52
123.50
122.98
121.97
84.61
82.93
65.04
54.58
41.95
37.02
21.41
f1 (ppm)

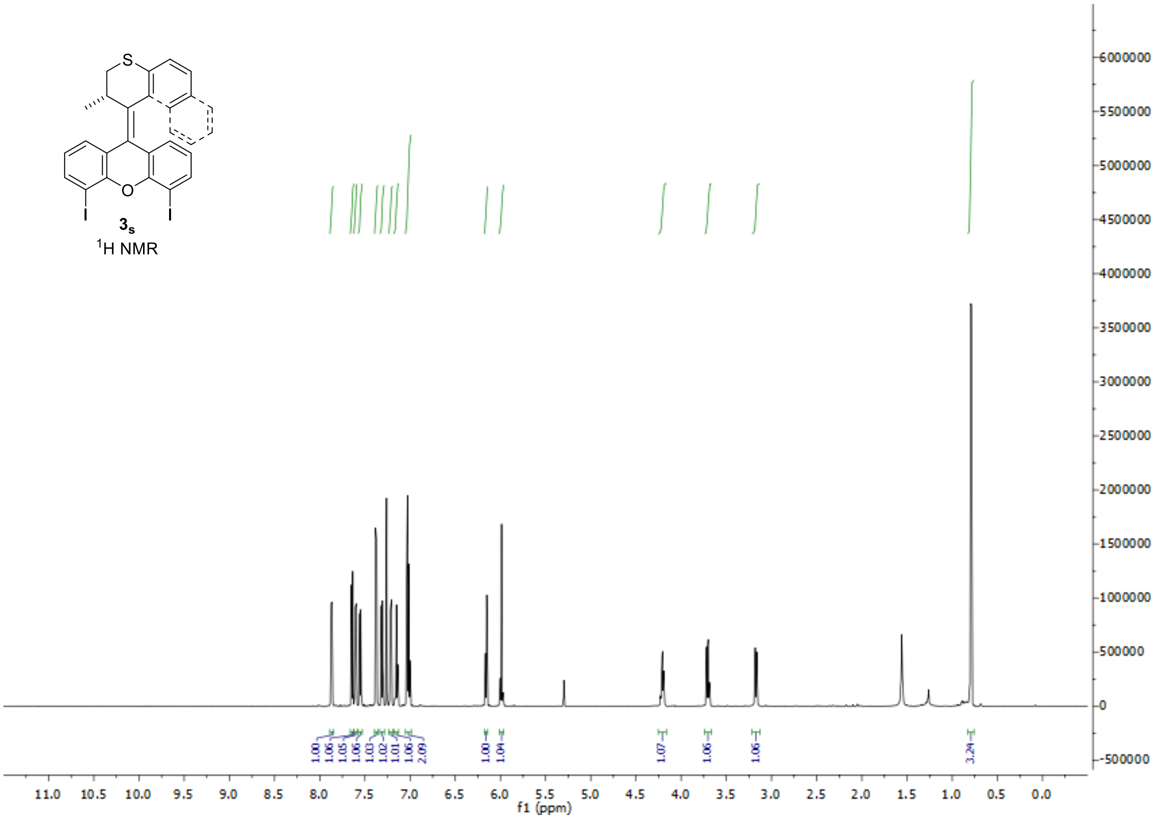

S
O
I
I
3s
1H NMR
1.00
1.06
1.05
1.06
1.03
1.02
1.01
1.06
2.09
1.00
1.04
1.07
1.06
1.06
3.24
f1 (ppm)

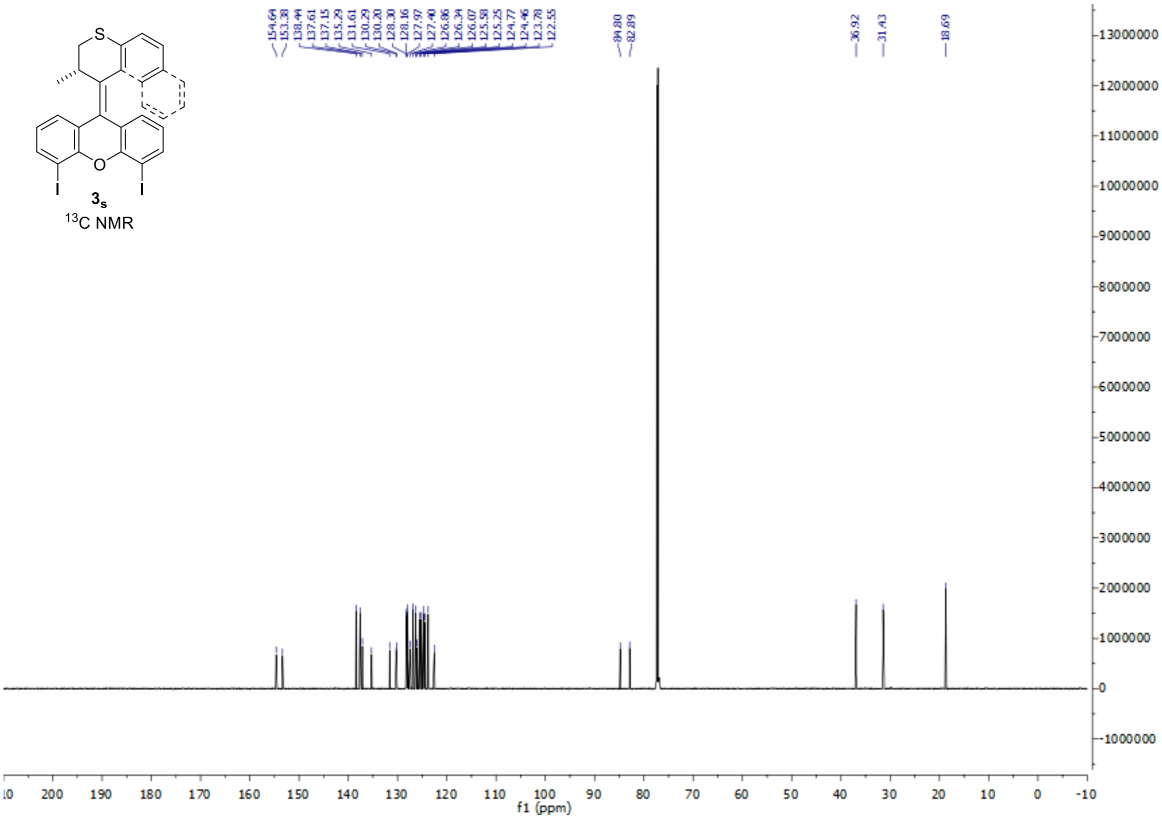

S
O
I
I
3s
13C NMR
154.64
153.38
138.44
137.61
137.15
135.29
131.61
130.29
130.20
128.30
128.16
127.97
127.40
126.86
126.34
126.07
125.58
125.25
124.77
124.46
123.78
122.55
84.80
82.89
36.92
31.43
18.69
f1 (ppm)

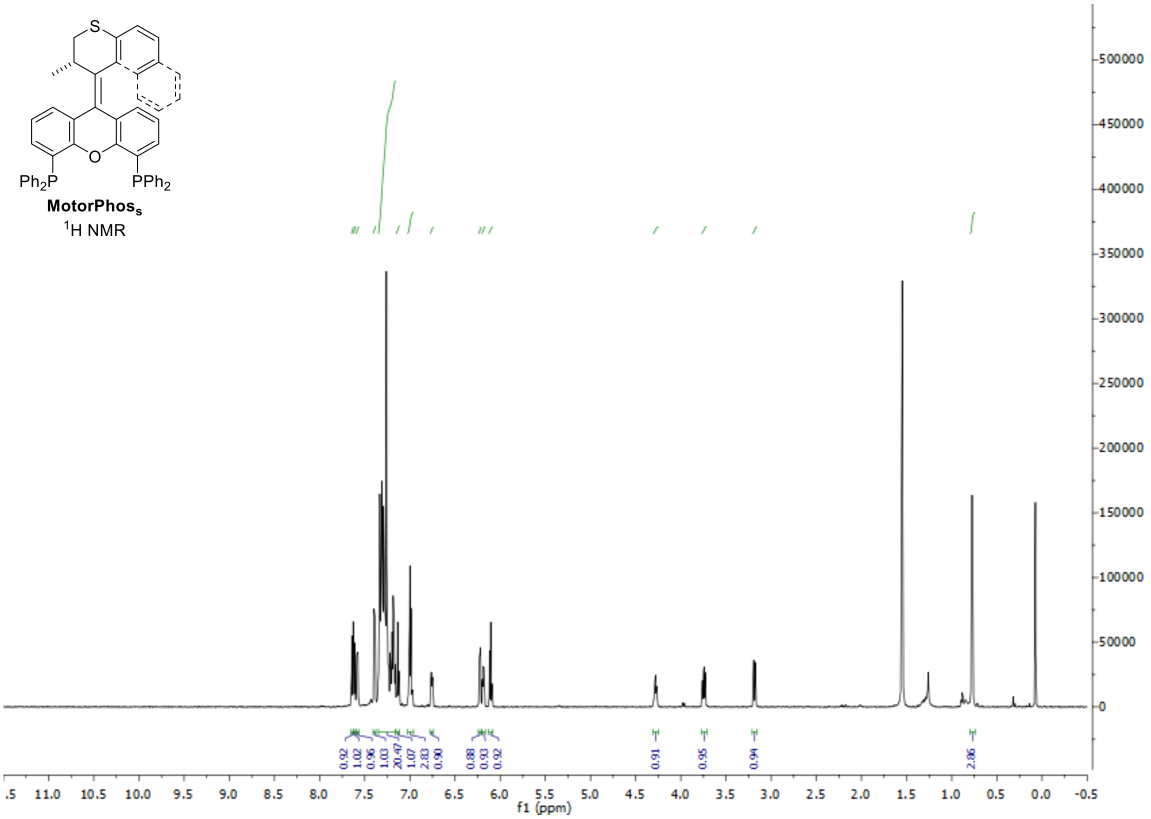
S
Ph2P
O
PPh2
MotorPhos s
1H NMR
0.92
1.02
0.96
1.03
20.47
1.07
2.83
0.90
0.88
0.93
0.92
0.91
0.95
0.94
2.86
f1 (ppm)

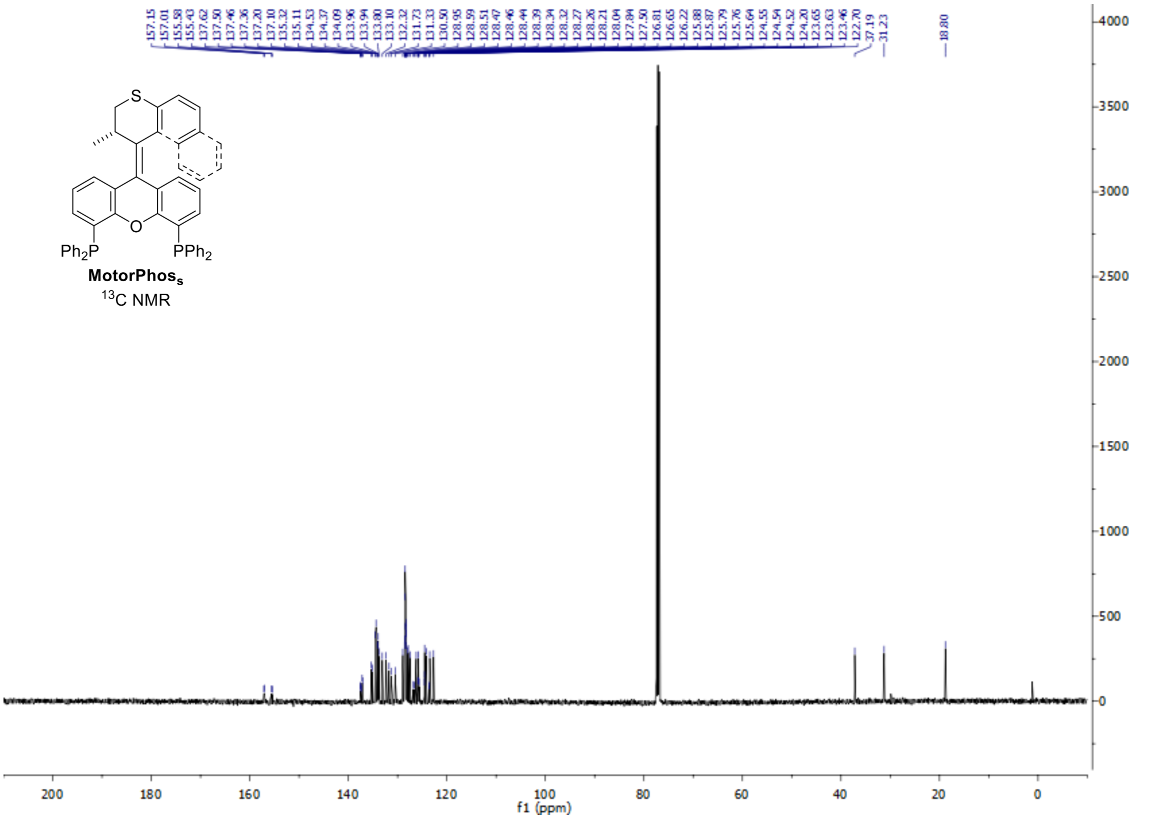
157.15
157.01
155.58
155.43
137.62
137.50
137.46
137.36
137.20
137.10
135.32
135.11
134.53
134.37
134.09
133.96
133.94
133.80
133.10
132.32
131.73
131.33
130.50
128.95
128.59
128.51
128.47
128.46
128.44
128.39
128.34
128.32
128.27
128.26
128.21
128.04
127.84
127.50
126.81
126.65
126.22
125.88
125.87
125.79
125.76
125.64
124.55
124.54
124.52
124.20
123.65
123.63
123.46
122.70
37.19
31.23
18.80
S
Ph2P
O
PPh2
MotorPhos s
13C NMR
f1 (ppm)

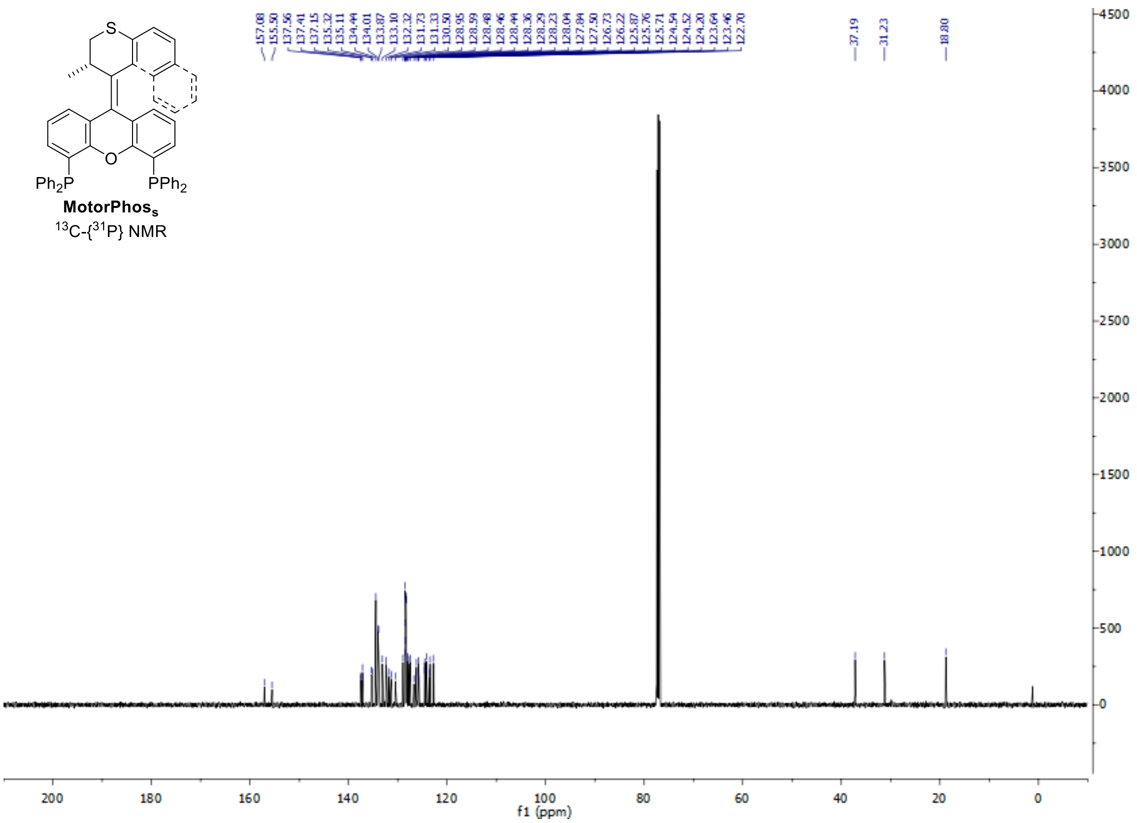
S
O
Ph2P
PPh2
MotorPhoss
13C-{31P} NMR
157.08
155.50
137.56
137.41
137.15
135.32
135.11
134.44
134.01
133.87
133.10
132.32
131.73
131.33
130.50
128.95
128.59
128.48
128.46
128.44
128.36
128.29
128.23
128.04
127.84
127.50
126.73
126.22
125.87
125.76
125.71
124.54
124.52
124.20
123.64
123.46
122.70
37.19
31.23
18.80
f1 (ppm)

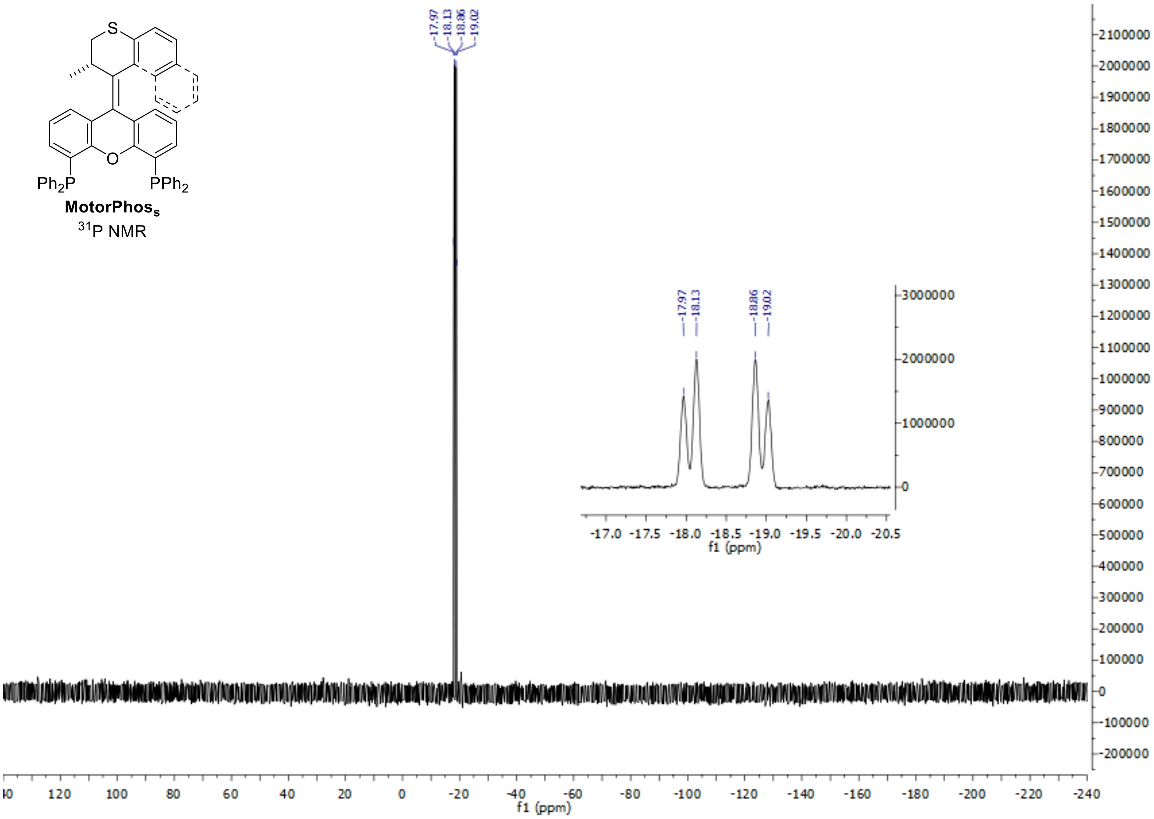
S
O
Ph2P
PPh2
MotorPhoss
31P NMR
-17.97
-18.13
-18.86
-19.02
f1 (ppm)

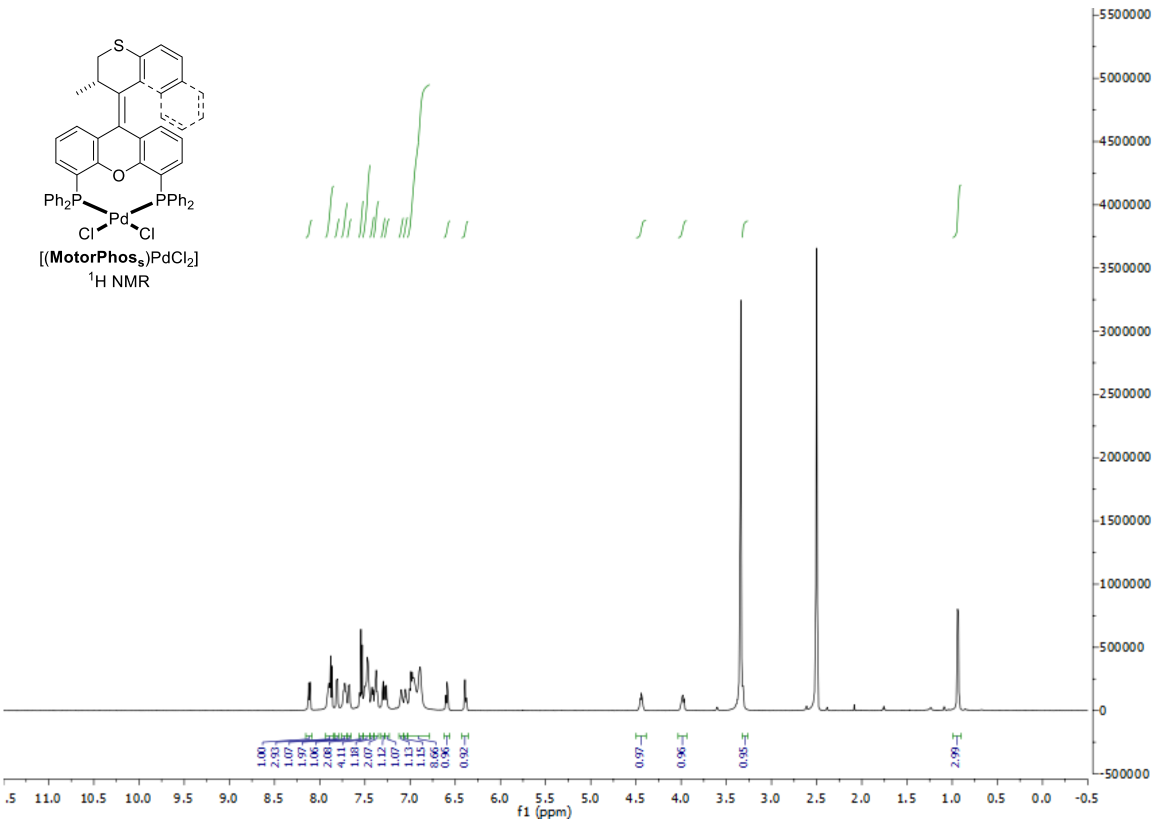
[(MotorPhos$_s$)PdCl$_2$]
^{1}H NMR
f1 (ppm)

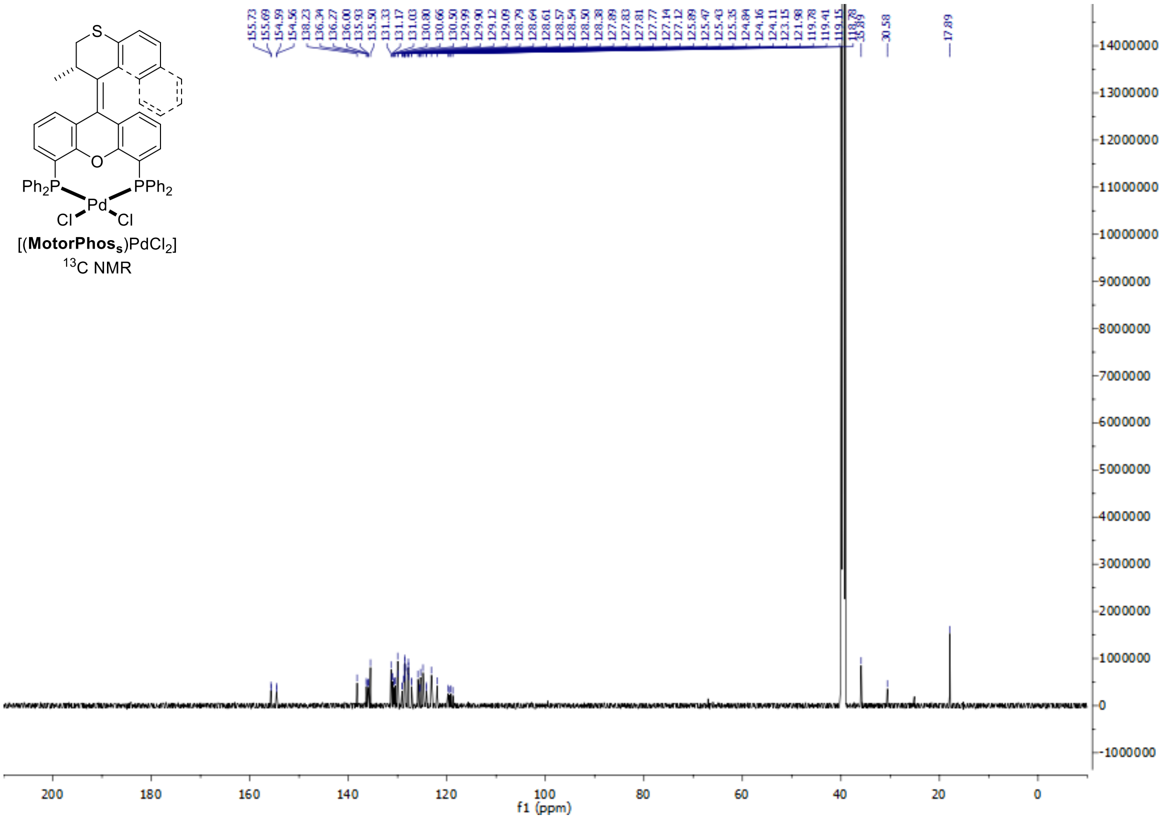
[(MotorPhos$_s$)PdCl$_2$]
^{13}C NMR
f1 (ppm)

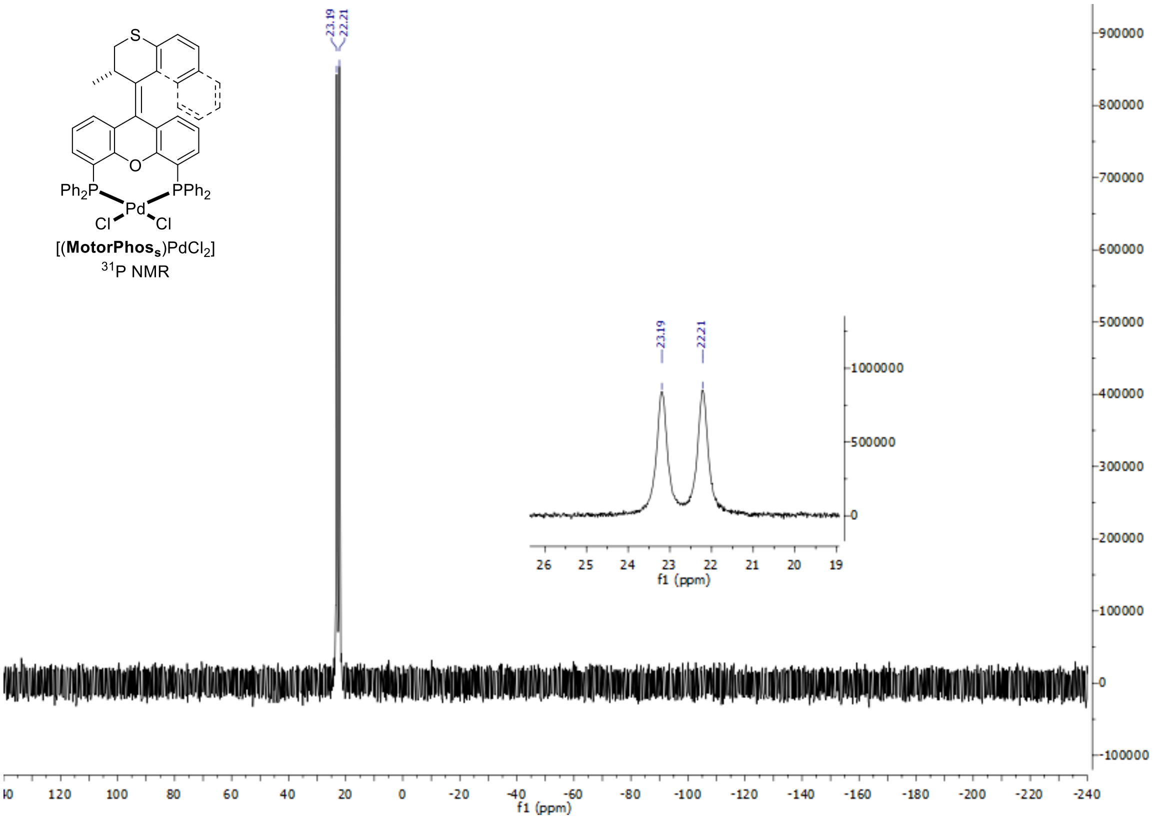

9. Coordinates and Energies of DFT Structures

9.1. PH_2 Analogue of [(MotorPhos)$PdCl_2$]

a-metastable-[eq]-i

C -3.8899054249 -1.6930826731 0.1815896047
C -2.685525012 -1.432927599 -0.3620552096
C -2.3174632785 -0.0995810034 -0.9223175394
C -1.0620561201 0.3726507763 -0.5574505394
O -0.2687991356 -0.3974436881 0.2714638752
C -0.2835582388 -1.7288916249 -0.0821319344
C -1.496571457 -2.3276182318 -0.4142734925
C -4.998200421 -0.7022101133 0.1302032292
C -6.1539145768 -1.0958444539 -0.5185767831
S -6.2751066753 -2.6805001342 -1.2978079026
C -5.236877947 -3.7463036089 -0.2161264629
C -3.0626614356 0.6874027129 -1.8027953261
C -2.5583521529 1.8998096346 -2.2642158809
C -1.2893162307 2.3390887397 -1.8909739805
C 0.9686883474 -3.6880327664 -0.632744302
C -0.2242921128 -4.3091746063 -0.9968033101

C -1.4372990136 -3.629868406 -0.9140599497
C -4.9554814969 0.5609246323 0.7994891558
C -6.0562053776 1.4504384826 0.6597159564
C -7.1937679621 1.0358134025 -0.0822102063
C -7.2586934167 -0.2142705771 -0.6314428953
C -3.8520232737 0.968929446 1.5958453054
C -3.8314844786 2.2048121239 2.1882030368
C -4.9130549923 3.0999710054 2.022776217
C -6.0022258724 2.7250254443 1.2807410031
H -5.8919274752 -4.4608852384 0.2874165371
H -4.5767728983 -4.2992692406 -0.8895173547
H -4.0396595294 0.344699804 -2.1250652279
H -3.1504065217 2.4987352862 -2.9469681972
H -0.8862324536 3.2584798624 -2.3021896806
H 1.9140143532 -4.2056678295 -0.7559209965
H -0.2022008505 -5.3203356727 -1.3875857651
H -2.34620275 -4.1023334251 -1.2703351395
H -8.0326424828 1.7186170318 -0.1816982125
H -8.149434841 -0.5420314582 -1.1575463739
H -3.0209044618 0.2861664345 1.734992089
H -2.9800427034 2.4978562989 2.7943807675
H -4.8818173094 4.0771842993 2.4935788716
H -6.8464963016 3.3982819761 1.1610090339
C -0.517232569 1.5615568561 -1.0279777783
C 0.9470039377 -2.3680306055 -0.1820683034
Pd 2.530886282 0.238557857 -1.63312148
Cl 2.495621421 1.7272453646 -3.4344611118
Cl 3.6717516177 -1.4793498601 -2.7392379358
C -3.4299544999 -3.8952499744 1.5623212192
H -2.823201998 -4.5129166286 0.9005905816
H -2.7520420415 -3.3091035206 2.1885765815
H -3.9966983234 -4.5661757884 2.2145281076
C -4.4063047146 -2.9764676035 0.8221581773
H -5.1119987671 -2.6229244374 1.5826983719
P 2.4080748744 -1.3191492686 0.0216011056
H 2.306800742 -0.8950766662 1.3576828211
H 3.4592663041 -2.2340811664 0.1083345073
P 1.2320256037 1.8383539095 -0.6649384695
H 1.4491894575 3.1573984803 -1.0678501131
H 1.2521718747 1.9749278697 0.7338220687

Energy= -3246.3840321
Zero-point correction= 0.419992 (Hartree/Particle)
Thermal correction to Gibbs Free Energy= 0.357991

Sum of electronic and zero-point Energies= -3245.964040
Sum of electronic and thermal Energies= -3245.933760
Sum of electronic and thermal Enthalpies= -3245.932816
Sum of electronic and thermal Free Energies= -3246.026041

a-metastable-[eq]-c

C -4.0068837678 -1.7283380689 0.0219676496
C -2.8113702616 -1.4777274455 -0.5439403621
C -2.4465766624 -0.155316341 -1.1256332085
C -1.1895977195 0.3245051803 -0.7588080334
O -0.4188035514 -0.4347254171 0.0948497443
C -0.4080312348 -1.7571799076 -0.2783773651
C -1.6217588907 -2.3669784725 -0.6068626208
C -5.1102540314 -0.7318408718 -0.0200753114
C -6.2803279042 -1.1259428928 -0.641982264
S -6.4219612339 -2.718202934 -1.403049725
C -5.3715249711 -3.7781095172 -0.3271016681
C -3.1996745646 0.6060476856 -2.016803138
C -2.7037501011 1.8154421566 -2.4956874436
C -1.4422707009 2.264513762 -2.1172088335
C 0.8282509917 -3.7155998564 -0.8449992322
C -0.3600102013 -4.3429528702 -1.2056388707
C -1.5729657889 -3.6650290047 -1.1105842382
C -5.0459649581 0.5393606041 0.632254138
C -6.144132754 1.4336256208 0.5028767611
C -7.2992869793 1.0160774259 -0.2096900028
C -7.3826425006 -0.2400641177 -0.7426858867
C -3.923939933 0.9508736605 1.4005496263
C -3.8834246932 2.1948518649 1.9747029828
C -4.9628315751 3.0944767943 1.8188315648
C -6.0696212166 2.7162814238 1.10509093
H -6.0220640795 -4.4814814464 0.1975006875
H -4.7281057188 -4.3445477904 -1.005683225
H -4.1756756391 0.2501749334 -2.3282353929
H -3.2993313102 2.4055816557 -3.1832747924
H -1.0600470997 3.1974341312 -2.5206249572
H 1.7697358591 -4.2458822438 -0.9519851569
H -0.3373764899 -5.3562736065 -1.5911818579
H -2.4878170798 -4.1355420989 -1.4545936048
H -8.136543385 1.7022469522 -0.2995247512
H -8.2859904341 -0.5691275783 -1.2460181518
H -3.0939761903 0.2654967128 1.5333175509
H -3.0180324122 2.4904199326 2.5594383407

H -4.9157151287 4.0776970041 2.2754691059
H -6.9123863271 3.3929545098 0.9941829862
C -0.6523665807 1.5081190978 -1.243920163
C 0.8218444246 -2.3905419109 -0.3943353114
C -3.5245682425 -3.9202625314 1.4088157061
H -2.9347689429 -4.5427063968 0.7360306923
H -2.8309955134 -3.3333281945 2.0168177227
H -4.0780908616 -4.5866514493 2.0767495104
C -4.5145931075 -3.0027870935 0.6857508729
H -5.2008968718 -2.6394845871 1.4592764616
Pd 2.9015657374 0.7342678474 -0.3513693749
Cl 3.7706703645 2.8964619621 -0.4285985548
Cl 4.9505224909 -0.2213533253 0.2287686497
P 1.0352190144 2.0152925225 -0.7915914649
H 1.2648028076 2.961718455 -1.7912168408
H 0.8112177145 2.8529984164 0.3074561793
P 2.3663529447 -1.4883584442 -0.0430809044
H 2.5921024533 -1.8101974496 1.3003714548
H 3.2723245873 -2.3471777875 -0.6671334194

Energy= -3246.3780871
Zero-point correction= 0.420023 (Hartree/Particle)
Thermal correction to Gibbs Free Energy= 0.357761
Sum of electronic and zero-point Energies= -3245.958064
Sum of electronic and thermal Energies= -3245.927546
Sum of electronic and thermal Enthalpies= -3245.926601
Sum of electronic and thermal Free Energies= -3246.020326

a-metastable-[eq]-e

C -4.0816196414 -1.7791110867 -0.0356398393
C -2.8734659489 -1.5310339597 -0.5756369292
C -2.5028193046 -0.1909761049 -1.1244804799
C -1.2738627197 0.2943384462 -0.6820831417
O -0.5711128277 -0.4786521456 0.2056904831
C -0.4922364146 -1.7882423759 -0.1786450989
C -1.6736100892 -2.4170769945 -0.5815492808
C -5.1682233244 -0.765064184 -0.0779514923
C -6.336269316 -1.1274920068 -0.7213131548
S -6.499136846 -2.7066200421 -1.5067187701
C -5.4588458849 -3.7984283356 -0.4514820526
C -3.2184181579 0.5949992044 -2.0235474983
C -2.7121453184 1.8298328764 -2.4287471189
C -1.4684315263 2.2737465579 -1.9892972191

C 0.8237868068 -3.7167083648 -0.6549366266
C -0.3316713294 -4.3713048594 -1.0696627101
C -1.5644870203 -3.7165529308 -1.06826631
C -5.0855800629 0.494589188 0.5954883756
C -6.160825243 1.4150223929 0.4586662885
C -7.3148274054 1.0297193562 -0.2739034682
C -7.4189548613 -0.2179747107 -0.8236537994
C -3.9666103732 0.8710897595 1.3862813997
C -3.9001155666 2.1111269723 1.9667943314
C -4.9552470039 3.0377096154 1.8002362193
C -6.0630967673 2.6910978835 1.0718083793
H -6.1156814964 -4.5192475039 0.0405835941
H -4.8020625358 -4.3405312567 -1.1374134677
H -4.1784297841 0.2490014595 -2.3912390538
H -3.2884659314 2.4451342934 -3.1107233723
H -1.0789480377 3.2240386327 -2.3419433239
H 1.7794762485 -4.2304212846 -0.6971699442
H -0.2682236271 -5.3926650037 -1.4286497366
H -2.4431827183 -4.2151416177 -1.4637208402
H -8.1359043113 1.7349411262 -0.3667700186
H -8.3228111268 -0.52106027 -1.3422334607
H -3.156615919 0.1654045986 1.5344309091
H -3.0325447845 2.3798467317 2.5610811234
H -4.8887592119 4.0171380059 2.2626150082
H -6.8881872878 3.3885651115 0.955922318
C -0.7086862486 1.4878972186 -1.1153896456
C 0.7627493643 -2.3842528481 -0.2306927135
C -3.6576536482 -4.0009061306 1.3256956688
H -3.0754927357 -4.6325791598 0.6547353216
H -2.9581690432 -3.4368680293 1.9482157649
H -4.2350721673 -4.6601683474 1.9802003151
C -4.618025754 -3.0558285961 0.5984207694
H -5.3168680399 -2.6968232318 1.3623272017
Pd 1.8798463754 0.6657970451 1.1007669589
Cl 1.4034701019 2.6162001408 2.288337826
Cl 2.6206374925 -0.5021958988 2.9787852613
P 1.0169612029 1.8522232416 -0.6635712619
H 1.6458004654 1.7156450141 -1.9158239711
H 1.0402125431 3.242988499 -0.5344922158
P 2.2349552423 -1.3436916116 0.051550793
H 3.1345898253 -2.2127653825 0.6736640889
H 2.777381164 -1.3263673777 -1.2476973329

Energy= -3246.3708782

Zero-point correction= 0.419128 (Hartree/Particle)
Thermal correction to Gibbs Free Energy= 0.355696
Sum of electronic and zero-point Energies= -3245.951750
Sum of electronic and thermal Energies= -3245.921182
Sum of electronic and thermal Enthalpies= -3245.920238
Sum of electronic and thermal Free Energies= -3246.015182

TS-metastable-i-to-c

C 3.0374188523 1.0180406951 0.3451151469
C 1.8179527654 1.0052063131 -0.2254017535
C 1.2444738418 -0.1965679763 -0.8956001554
C -0.0757053521 -0.487144773 -0.5614418478
O -0.7295489871 0.3239618464 0.3426682291
C -0.5093588614 1.6543873877 0.072478456
C 0.788760827 2.0787145859 -0.2138017886
C 3.9650605466 -0.1379965744 0.2213861333
C 5.1835565336 0.1049299748 -0.3849201374
S 5.5821867617 1.7033466636 -1.0330335996
C 4.7159863406 2.8397957629 0.1257789285
C 1.8647001318 -1.0125852394 -1.8414530658
C 1.1771591498 -2.0863494119 -2.3995852603
C -0.1459773212 -2.3450009992 -2.0503820051
C -1.4160626299 3.8217013118 -0.3354041978
C -0.1376856106 4.2729034294 -0.6530094052
C 0.9499317657 3.4038999457 -0.617153485
C 3.6957865188 -1.4248828685 0.7841686713
C 4.6337013321 -2.4739190827 0.5788233797
C 5.8408134522 -2.199431239 -0.1169397475
C 6.1270987341 -0.9385503168 -0.5609289238
C 2.5232223312 -1.7024967985 1.5368458966
C 2.2816681075 -2.9610374129 2.0231548594
C 3.1996959781 -4.0106118579 1.7904735545
C 4.3522887338 -3.7670407078 1.0906883165
H 5.4713270568 3.3901694127 0.6911847609
H 4.1729240707 3.5490537105 -0.5045052735
H 2.888034686 -0.8050361211 -2.1341953412
H 1.67179584 -2.7188531663 -3.128234387
H -0.6770446702 -3.1692503084 -2.5162932933
H -2.2600420691 4.5015486223 -0.3976802195
H 0.0064792059 5.3027960572 -0.9602633215
H 1.9298435817 3.7475321826 -0.9304922129
H 6.5552745828 -3.0040603737 -0.2652028717
H 7.0717166329 -0.7251920077 -1.0506397442

H 1.8169277389 -0.902380498 1.7295669854
H 1.3808745799 -3.1531059991 2.5974691539
H 2.99386486 -5.0032088984 2.1775875189
H 5.0735604175 -4.5618092464 0.9218281871
C -0.8001205849 -1.5266729646 -1.1247704906
C -1.6204049151 2.4830230175 0.0121308719
C 2.9166850264 3.1535913776 1.8898419013
H 2.4365731029 3.9113423417 1.2708995773
H 2.1365390078 2.6440564379 2.4616702297
H 3.5707710848 3.6705735739 2.5979913076
C 3.7448118294 2.1428869293 1.091419162
H 4.3632946041 1.618803301 1.8290757473
Pd -4.2096648625 -0.093204642 -0.7346490088
Cl -5.4625757757 -1.8961428216 -1.5091357733
Cl -6.1063884701 1.2561745674 -0.6302319261
P -2.5592013026 -1.7097955488 -0.7188539359
H -2.8733983806 -2.8061494965 -1.5224886724
H -2.5227247039 -2.3262395928 0.5416697095
P -3.2753228321 1.772401409 0.2584062768
H -3.3412976784 1.6406789508 1.6542307137
H -4.0476443952 2.9208386258 0.081356169

Energy= -3246.376672
Zero-point correction= 0.419739 (Hartree/Particle)
Thermal correction to Gibbs Free Energy= 0.358867
Sum of electronic and zero-point Energies= -3245.956933
Sum of electronic and thermal Energies= -3245.927215
Sum of electronic and thermal Enthalpies= -3245.926270
Sum of electronic and thermal Free Energies= -3246.017805

TS-metastable-c-to-e

C 3.0309331449 1.0194045892 0.3771299335
C 1.788445484 1.0137515252 -0.1409524909
C 1.1981970797 -0.1884738729 -0.8051137002
C -0.0887567376 -0.5182397346 -0.3784211311
O -0.6754377648 0.2872146644 0.5651906345
C -0.5285255032 1.6156840625 0.2643262459
C 0.750659603 2.0787505375 -0.054776083
C 3.9540741028 -0.1314934218 0.1906561717
C 5.1449483912 0.125603476 -0.4620827003
S 5.5140082765 1.7386587574 -1.0922818134
C 4.6919158884 2.8489766375 0.123251173
C 1.7722865518 -0.9838600715 -1.7922237905

C 1.0767971367 -2.0823665116 -2.2973206739
C -0.2015891041 -2.3894929741 -1.843231052
C -1.5124588122 3.7502179085 -0.1373682908
C -0.2483750976 4.251986617 -0.4298911474
C 0.8662841633 3.4129614976 -0.4367431069
C 3.7085425337 -1.4295005207 0.7389321953
C 4.6355660222 -2.473648278 0.469801053
C 5.8119946627 -2.183351693 -0.2708235449
C 6.0801648077 -0.9131075943 -0.6997166699
C 2.5697959225 -1.7223424964 1.5366393243
C 2.3464568216 -2.9915454023 2.0038333606
C 3.2517912859 -4.036154638 1.706599694
C 4.3740928839 -3.7778857231 0.9640233617
H 5.4680489759 3.3931638989 0.6659350346
H 4.1185742915 3.566304474 -0.4701972063
H 2.7682793109 -0.752187748 -2.1539340458
H 1.5378018536 -2.7028931665 -3.057748936
H -0.727872762 -3.2458431895 -2.2545116481
H -2.379696526 4.4007529906 -0.1978923562
H -0.1362670324 5.2952668755 -0.7038567876
H 1.827323621 3.7925994618 -0.7668407719
H 6.5192034519 -2.984320748 -0.4664601831
H 7.0034929304 -0.689043834 -1.2240584317
H 1.8746522344 -0.9265910548 1.7813199933
H 1.4717550975 -3.1944327431 2.6136833994
H 3.0609297664 -5.0369287374 2.0800515648
H 5.0864459232 -4.5690922242 0.7471152206
C -0.8194797622 -1.5895434777 -0.8736714265
C -1.6733361737 2.3955410166 0.1748302296
C 2.9687324879 3.1223916581 1.9686248417
H 2.4638808652 3.8922842286 1.3852856894
H 2.2127561277 2.6003596676 2.5610876573
H 3.6501637316 3.6261261049 2.6602302084
C 3.7654582038 2.1303850416 1.116820271
H 4.4158933692 1.593785196 1.8168872477
Pd -3.6838700941 -0.4802407072 1.1463716894
Cl -4.3349970917 -2.4822474908 2.1444311129
Cl -4.9193615058 0.7795836849 2.6611912409
P -2.4917744481 -1.9183793878 -0.2253116127
H -3.1630368575 -2.3088788323 -1.3942423695
H -2.324969306 -3.1678418251 0.3769604079
P -3.2954003442 1.5662152771 0.176388311
H -4.1499593515 2.5810988931 0.6136138957
H -3.5719187896 1.6069777867 -1.2029833924

Energy= -3246.3693233
Zero-point correction= 0.419038 (Hartree/Particle)
Thermal correction to Gibbs Free Energy= 0.357410
Sum of electronic and zero-point Energies= -3245.950285
Sum of electronic and thermal Energies= -3245.920415
Sum of electronic and thermal Enthalpies= -3245.919471
Sum of electronic and thermal Free Energies= -3246.011913

TS1-i

C 2.8889056084 1.2297798386 0.0730170425
C 1.5723529458 1.0993430014 -0.2364315705
C 1.0023677834 -0.1858446556 -0.7367791454
C -0.1228755652 -0.6506150102 -0.0736086684
O -0.6015004838 0.0836121366 0.9918315584
C -0.6431495963 1.4326026943 0.7085201944
C 0.4315236327 2.0375467159 0.0437537929
C 3.751411553 0.0045135329 0.0424343877
C 4.9698574452 0.0223831945 -0.6202643499
S 5.7345266105 1.5768425662 -0.9640181306
C 5.091705885 2.335717364 0.5649001648
C 1.4583102958 -0.9339838348 -1.8229152514
C 0.8031490899 -2.1043843998 -2.1942186719
C -0.3338818927 -2.5383125169 -1.5132479055
C -1.9829399655 3.4146985626 0.6563861256
C -0.9570658197 4.0380670205 -0.0444504027
C 0.2144931502 3.3555092001 -0.3683710396
C 3.3663345825 -1.2183121444 0.6998200351
C 4.0714083868 -2.4180778612 0.4054525123
C 5.1989971185 -2.3699423804 -0.4557166291
C 5.6754074806 -1.1711031478 -0.9049001365
C 2.334475925 -1.279492581 1.6728095234
C 1.981391216 -2.4662484513 2.2622284089
C 2.6362423043 -3.6675047857 1.911386654
C 3.6663616696 -3.6366073638 1.008967265
H 5.3590593124 1.6744968228 1.3926083299
H 5.6047290999 3.2895500822 0.7025411328
H 2.3379973118 -0.6011907179 -2.363912945
H 1.1690450228 -2.6772680032 -3.0388911708
H -0.8613888408 -3.4273928113 -1.8420612286
H -2.9122115959 3.93725259 0.8564388635
H -1.0841544619 5.0603165346 -0.3831464771
H 0.9430041097 3.8496400545 -0.9909398903
H 5.7236660996 -3.2917351903 -0.6897252094

H 6.6010273915 -1.1148718132 -1.4685528187
H 1.8234055296 -0.3675671877 1.9592871375
H 1.1963107312 -2.4792750272 3.012199454
H 2.3392514624 -4.602912578 2.3742368571
H 4.2047106576 -4.5452467434 0.7543577043
C -0.8158834243 -1.7986081846 -0.4336361605
C -1.8335799388 2.0777316007 1.0221990099
Pd -3.870454657 -0.4215456997 0.0052833385
Cl -4.4601533755 -1.7919408896 -1.7940696296
Cl -5.2497302191 1.3778189297 -0.5772650546
C 3.3374870625 3.7194192501 -0.5416579146
H 2.9932718911 3.3600837218 -1.513638645
H 2.6142302652 4.4303402946 -0.1437691952
H 4.2622929378 4.274779781 -0.7173350566
C 3.5790208516 2.5527343857 0.4471257105
H 3.2110298247 2.8488103544 1.439804653
P -3.1773671371 1.0233394957 1.6207856824
H -2.6677397686 0.5117473677 2.8262080275
H -4.1217274825 1.9363339188 2.0946306538
P -2.3806781669 -2.0904869715 0.4199649866
H -2.7432596904 -3.3776303161 0.0182569306
H -1.9766141625 -2.3237547451 1.7462701607

Energy= -3246.3699888
Zero-point correction= 0.419639 (Hartree/Particle)
Thermal correction to Gibbs Free Energy= 0.359431
Sum of electronic and zero-point Energies= -3245.950350
Sum of electronic and thermal Energies= -3245.921178
Sum of electronic and thermal Enthalpies= -3245.920233
Sum of electronic and thermal Free Energies= -3246.010558

TS1-c

C -3.1155112145 1.0809026743 -0.2305105013
C -1.9018435624 1.1282026844 0.3779818264
C -1.3167949049 -0.0481199351 1.0803856437
C -0.0218333948 -0.3934457597 0.707292087
O 0.5726467131 0.3464929497 -0.2896673657
C 0.4196879706 1.6941474935 -0.0638567867
C -0.8417629128 2.1887928284 0.3091089281
C -3.8173616265 -0.2393934971 -0.324212458
C -5.1569824652 -0.3475636224 0.020567262
S -6.1566424393 1.1078462022 0.0582495406
C -5.2368898646 1.8943954408 -1.3057524388

C -1.9240766454 -0.7877363912 2.0915702899
C -1.2428079425 -1.8462124514 2.6861953849
C 0.0557706595 -2.1615416203 2.2966220948
C 1.4610543257 3.8288381072 0.1794053877
C 0.2423335551 4.345564363 0.5972319165
C -0.8861703357 3.5317808183 0.6841217781
C -3.1476306363 -1.4314158213 -0.7806898687
C -3.7632035668 -2.6971439 -0.5749609407
C -5.0672922296 -2.7546920185 -0.0169897187
C -5.7724664202 -1.6076133087 0.2127473989
C -1.9104282989 -1.4036673236 -1.4757841803
C -1.2903522089 -2.560657246 -1.8733365499
C -1.8694636934 -3.8189535834 -1.5969141271
C -3.0864685935 -3.8797419461 -0.97083146
H -5.2086886977 1.1820805794 -2.1337769239
H -5.8020201173 2.7734902765 -1.6218423446
H -2.9363928761 -0.5414947363 2.3950291642
H -1.722955181 -2.4237482856 3.4683757255
H 0.5800814959 -2.9778697556 2.7843617835
H 2.3370149465 4.4689550963 0.1378877878
H 0.1702935773 5.3877877485 0.8884261752
H -1.7951777924 3.9475740851 1.0885040919
H -5.5266963973 -3.7244913949 0.1509842769
H -6.809949269 -1.6448678642 0.5288478522
H -1.4518155048 -0.4482745875 -1.7024207165
H -0.3500742566 -2.5062752658 -2.4135753745
H -1.3630491479 -4.7281423305 -1.9038859204
H -3.5644389699 -4.8374929499 -0.7849235432
C 0.6965599659 -1.4222241984 1.2957603551
C 1.5686079797 2.4698935949 -0.1325735447
C -3.9916527212 3.5024761391 0.1371377187
H -3.825993977 3.2108148988 1.1762582453
H -3.3051373404 4.3074046168 -0.1246842781
H -5.0012012013 3.9174291277 0.0823631274
C -3.8388509231 2.2995643952 -0.8273382609
H -3.2683105903 2.6229915387 -1.7093931734
Pd 4.0335799036 -0.3639242443 -0.0175742272
Cl 5.1856823424 -2.3744516727 0.249755941
Cl 5.8752095651 0.7383975023 -0.9348754344
P 2.3998846804 -1.7819098206 0.7748569107
H 2.8289061371 -2.5276734659 1.8739976981
H 2.2083180503 -2.800102181 -0.1667593799
P 3.1723140102 1.7089459429 -0.5471107242
H 3.2106263314 1.8514174547 -1.9392936034

H 4.0168927102 2.7510176191 -0.1617765468

Energy= -3246.3642798
Zero-point correction= 0.419931 (Hartree/Particle)
Thermal correction to Gibbs Free Energy= 0.359805
Sum of electronic and zero-point Energies= -3245.944349
Sum of electronic and thermal Energies= -3245.915030
Sum of electronic and thermal Enthalpies= -3245.914086
Sum of electronic and thermal Free Energies= -3246.004474

TS1-e

C -2.7452231036 1.0708822035 -0.3205510569
C -1.6470112845 1.2414240003 0.4614707212
C -1.1525413618 0.149451167 1.354485837
C 0.1851584114 -0.1898553087 1.1935300861
O 0.874287068 0.4746120854 0.2162147089
C 0.6994985987 1.8282682355 0.2686109782
C -0.6059878347 2.3296207713 0.4108755235
C -3.3573485233 -0.294109198 -0.3909422454
C -4.7211361361 -0.4567781928 -0.2009615734
S -5.777295223 0.9489168495 -0.3870905585
C -4.7488078665 1.6594482135 -1.7163433345
C -1.8758996046 -0.5373216628 2.3252475785
C -1.2596333297 -1.5371584631 3.0773996764
C 0.0895888095 -1.8360771472 2.9056200385
C 1.6958940828 3.9944332308 0.3589516931
C 0.426020912 4.529374349 0.5277726332
C -0.7032690282 3.7088758876 0.5897013009
C -2.5668247831 -1.4682173357 -0.6583996978
C -3.134765997 -2.7513587656 -0.4247992367
C -4.4982761586 -2.8488277902 -0.0428388586
C -5.2902780132 -1.7351985397 0.0055384924
C -1.2459223658 -1.4111183251 -1.1745905628
C -0.5010658937 -2.5476273777 -1.3610686427
C -1.0449492919 -3.8148464535 -1.0520007544
C -2.3386620342 -3.9118474673 -0.6107977704
H -4.6033836313 0.8774621688 -2.4650649267
H -5.3120747489 2.4760545324 -2.1723241112
H -2.9274436331 -0.3104687585 2.4678485021
H -1.8370115691 -2.0834599831 3.8150134597
H 0.554466216 -2.6038665514 3.5165940854
H 2.5644365252 4.6457335036 0.3428607715
H 0.307151549 5.6009745269 0.6456270445

H -1.6580644162 4.1604454112 0.8095344889
H -4.9239828077 -3.8313734014 0.1405619971
H -6.3568226686 -1.8162235754 0.1886590568
H -0.8139246704 -0.4494155138 -1.4244465327
H 0.5145486935 -2.4732996338 -1.7391246974
H -0.4403084733 -4.7051769945 -1.1894762421
H -2.7828181705 -4.8827365459 -0.4084779388
C 0.8489303688 -1.1398140453 1.9589770216
C 1.8507155816 2.6077656492 0.2651380872
C -3.7081975841 3.4828370485 -0.3617740609
H -3.6759850953 3.3212528819 0.7179034992
H -2.9990324478 4.2656449236 -0.6300540684
H -4.7046595825 3.8654667731 -0.5965702277
C -3.4237531434 2.1772255086 -1.1432994728
H -2.7665718301 2.4079598554 -1.9936944363
Pd 3.4972888268 -0.462136741 -0.2018671247
Cl 3.4029604569 -2.6890881031 -0.9028489469
Cl 4.2113594795 0.3409568548 -2.2715788446
P 2.6467731799 -1.3155248567 1.7517648817
H 3.1134620306 -0.7796189372 2.9670992841
H 2.8792176837 -2.6737291227 1.9817695125
P 3.4693151258 1.7673997312 0.345534397
H 4.3047671338 2.5965130146 -0.4070897926
H 3.8756425721 2.1327994139 1.643273359

Energy= -3246.3594925
Zero-point correction= 0.418971 (Hartree/Particle)
Thermal correction to Gibbs Free Energy= 0.358554
Sum of electronic and zero-point Energies= -3245.940522
Sum of electronic and thermal Energies= -3245.911149
Sum of electronic and thermal Enthalpies= -3245.910205
Sum of electronic and thermal Free Energies= -3246.000939

at-I1-[ax]-i

C -4.1555495169 -1.4142927552 -0.3472726781
C -2.8457590209 -1.2159006792 -0.6811717059
C -2.2975802595 0.1438204294 -0.9307839599
C -0.9993595791 0.4093223159 -0.5295162675
O -0.3134497848 -0.5632117471 0.152488144
C -0.503760438 -1.8310298776 -0.3436910311
C -1.7689206831 -2.2552094075 -0.7783748443
C -4.9615598043 -0.3086911178 0.2317858145
C -6.3136066875 -0.1515825007 -0.0628878876

S -7.2650953983 -1.4523237127 -0.7708467854
C -6.257129679 -2.8026412737 -0.0981377613
C -2.9522286467 1.1547247415 -1.642799482
C -2.3184238916 2.3648175957 -1.9014661028
C -1.0017922329 2.5881099439 -1.4965032697
C 0.5534648814 -3.9236308801 -0.8333782716
C -0.6916018503 -4.4079485416 -1.2163771565
C -1.8218515519 -3.5932502949 -1.2016107995
C -4.3768929348 0.6414367294 1.1582181875
C -5.0687595242 1.848772407 1.4513502076
C -6.3750861346 2.0469582482 0.9313906832
C -7.0050169413 1.0443200766 0.2551721949
C -3.15454876 0.4112204201 1.8429655939
C -2.6149912402 1.3513604327 2.6840453833
C -3.263793873 2.5863102905 2.8955892564
C -4.4754139545 2.8178912729 2.299970838
H -6.2772111166 -2.7284731199 0.9922370794
H -6.7455257755 -3.7336547601 -0.3960853738
H -3.9660023325 0.9846405203 -1.9899651492
H -2.8441242483 3.1356625048 -2.4537227537
H -0.5008882996 3.5157995808 -1.7521396276
H 1.4382357328 -4.5464546117 -0.9082522128
H -0.7857125079 -5.4302182771 -1.5655734573
H -2.7462818156 -4.020548915 -1.5578877249
H -6.8943688596 2.976304011 1.1464604068
H -8.0431266952 1.1449442977 -0.0456562955
H -2.6425977495 -0.5342078128 1.7120857723
H -1.6847455916 1.1345856199 3.2000878296
H -2.8201575034 3.3300688209 3.5492643336
H -5.014736513 3.7425502722 2.4855514183
C -0.31966038 1.5905029796 -0.8032403203
C 0.6489558042 -2.6079551105 -0.3882887241
C -4.8585693655 -3.0263395087 -2.1615595932
H -5.6094185364 -2.3938792652 -2.6409858518
H -3.9062522609 -2.8087399734 -2.6453953761
H -5.122974208 -4.0715570318 -2.3557730482
C -4.8296992099 -2.7496835212 -0.6435714838
H -4.261428752 -3.5439279712 -0.1389927047
Pd 2.5705429938 -0.0361566874 -1.5126866022
Cl 3.5418986195 -1.7539067596 -2.7688944492
Cl 2.7946384059 1.6296313119 -3.1360915224
P 1.4429611707 1.6012594729 -0.4005800903
H 1.8289315596 2.920126726 -0.6479626306
H 1.4443930808 1.5791717143 1.0054991768

P 2.1976566921 -1.7345364673 -0.0406453902
H 2.0885454656 -1.431208185 1.3276775509
H 3.1432541922 -2.7617772798 -0.0097673957

Energy= -3246.3769356
Zero-point correction= 0.419878 (Hartree/Particle)
Thermal correction to Gibbs Free Energy= 0.358630
Sum of electronic and zero-point Energies= -3245.957058
Sum of electronic and thermal Energies= -3245.927153
Sum of electronic and thermal Enthalpies= -3245.926209
Sum of electronic and thermal Free Energies= -3246.018306

at-I1-[ax]-c

C -4.2653449848 -1.2850108245 -0.6556131834
C -2.9655935022 -1.0606967736 -1.0118949659
C -2.3973598018 0.30823257 -1.0931120461
C -1.0846758942 0.4950289544 -0.6779276197
O -0.4258673311 -0.574144189 -0.135072364
C -0.6272866859 -1.7437434475 -0.8204608084
C -1.9102000511 -2.0848029185 -1.2929898042
C -5.0280287167 -0.259266525 0.1018586823
C -6.3887348329 -0.0499706665 -0.1096912941
S -7.3924408956 -1.2344017261 -0.9396889294
C -6.3763414504 -2.6733548614 -0.505863604
C -3.0565100311 1.4046480607 -1.6509870476
C -2.4147163306 2.6339298165 -1.7549180613
C -1.0937395051 2.780966199 -1.3390619685
C 0.3606454619 -3.7608784642 -1.638887309
C -0.8936049919 -4.1616977063 -2.0749581893
C -2.005214268 -3.3365942512 -1.9147451732
C -4.3900405627 0.5523239293 1.1208388228
C -5.0484102712 1.7185426467 1.5993056507
C -6.371871526 1.99787568 1.1674908217
C -7.0465320565 1.1003141864 0.3937520505
C -3.1461418526 0.2184026852 1.7179305919
C -2.5556471002 1.0332898106 2.6507921819
C -3.1729946945 2.2380485855 3.0487058357
C -4.4038918227 2.5604894694 2.5411781966
H -6.3453800326 -2.7495878139 0.5840642499
H -6.8919890259 -3.5502188485 -0.9050169315
H -4.0792761079 1.287943734 -1.9942703912
H -2.9399297848 3.4804820135 -2.1831196687
H -0.5965347424 3.7395924852 -1.4534490772

H 1.2247272505 -4.3976039724 -1.8022172042
H -1.0132317346 -5.1200962388 -2.5682090108
H -2.9469972018 -3.6842574806 -2.310162914
H -6.8662919418 2.8960244061 1.5259909106
H -8.0949330146 1.2505432185 0.1558682146
H -2.6561902878 -0.7080019068 1.4448987061
H -1.6093364547 0.7388886961 3.0939441218
H -2.6894312459 2.8837321387 3.7742791767
H -4.9190619641 3.4588052705 2.8697225235
C -0.397938025 1.6943626133 -0.8002397234
C 0.5055136449 -2.5251936777 -1.0030407364
C -5.0771303933 -2.6160135751 -2.6440578442
H -5.8440809665 -1.920273559 -2.992441523
H -4.1464926553 -2.3329800638 -3.1363192519
H -5.3594609401 -3.6223652207 -2.9722354205
C -4.9743658767 -2.5579583631 -1.1051706808
H -4.3960052365 -3.421210277 -0.746371179
Pd 2.9963656558 0.204971429 -0.2244042887
Cl 4.876256012 -1.1419745837 0.0827854484
Cl 4.1747552486 2.1713230899 0.2017508514
P 2.1380268732 -1.9300698165 -0.4444644264
H 2.2681606303 -2.5718422631 0.793232427
H 2.9482320018 -2.7464089501 -1.2351949174
P 1.347315441 1.8191402403 -0.3015747194
H 1.7264380277 2.9282107378 -1.059381703
H 1.2489815146 2.4009164481 0.9679552259

Energy= -3246.3703703
Zero-point correction= 0.419865 (Hartree/Particle)
Thermal correction to Gibbs Free Energy= 0.358479
Sum of electronic and zero-point Energies= -3245.950505
Sum of electronic and thermal Energies= -3245.920374
Sum of electronic and thermal Enthalpies= -3245.919429
Sum of electronic and thermal Free Energies= -3246.011891

at-I1-[ax]-e

C 2.8919559762 1.2376035709 -0.1245690086
C 1.5459567935 1.0442483033 -0.2546782798
C 0.9920905005 -0.2510914068 -0.7365173555
C -0.1483649865 -0.7286626923 -0.1093605143
O -0.6260551874 -0.013890499 0.9416652997
C -0.6347669794 1.3378990673 0.7915083899
C 0.4612632696 1.9826777268 0.1918738624

C 3.8063971336 0.0734630619 -0.0083389991
C 5.0709216389 0.0758256134 -0.5899825677
S 5.8286682582 1.5741847637 -1.1250989873
C 4.9853412037 2.6399838257 0.0795877729
C 1.4870923669 -1.0097883602 -1.7980497643
C 0.8641488272 -2.2022331421 -2.1608592543
C -0.2890429617 -2.6410847242 -1.5110052529
C -1.8029474824 3.3682217244 1.2487508215
C -0.7187890209 4.0553265585 0.7168812798
C 0.3811904776 3.3815213393 0.1822181125
C 3.4214441143 -1.1073966425 0.7400749347
C 4.1630789802 -2.3089361806 0.5707698366
C 5.3480492545 -2.2898121653 -0.2106250607
C 5.8266953505 -1.1153613706 -0.7160360569
C 2.3507469386 -1.1293035465 1.6722523481
C 1.9653876713 -2.2866564045 2.3009346098
C 2.6467181384 -3.4970550601 2.047661802
C 3.7370892358 -3.4979309378 1.2178709166
H 5.2393002382 2.2826706514 1.0806568841
H 5.3961850658 3.6443057288 -0.0498528959
H 2.3779194466 -0.6763458769 -2.3204915002
H 1.2755090697 -2.79309299 -2.9717728144
H -0.7686272994 -3.5635629738 -1.823362844
H -2.6625957513 3.9104509219 1.6295951941
H -0.7300474757 5.1395986856 0.696166832
H 1.1708674793 3.976554836 -0.2521455501
H 5.9094044969 -3.210966401 -0.3390135183
H 6.7884050407 -1.0773338237 -1.2178694798
H 1.8226729805 -0.2116979941 1.899911452
H 1.1290648734 -2.2720878183 2.9939566566
H 2.3210343998 -4.409611577 2.5355253087
H 4.3058316981 -4.4088958361 1.0521357441
C -0.8319569225 -1.8838572738 -0.4701588598
C -1.7744457443 1.9730101326 1.275096806
C 3.1736587525 3.3412152814 -1.4972044934
H 3.8105662319 2.9237885078 -2.2810561121
H 2.1416769723 3.2008325162 -1.8199216122
H 3.3800596304 4.4148606746 -1.4283885191
C 3.4733911997 2.6465731007 -0.1528514334
H 3.0185123224 3.2250746249 0.6645150613
P -2.4084672541 -2.223896185 0.3758768583
H -3.3336271419 -1.8899471839 -0.6321925825
H -2.5093798471 -3.616634912 0.343563615
P -3.17804098 0.9126100558 1.7728187753

H -3.8656929753 1.7050369404 2.6949381484
H -4.0176666695 1.0663498598 0.6519876574
Pd -2.6363298368 -1.2239423435 2.4374734181
Cl -2.6659686409 -0.2455045306 4.5541629767
Cl -1.8642953707 -3.292877851 3.2008551222

Energy= -3246.3658369
Zero-point correction= 0.419195 (Hartree/Particle)
Thermal correction to Gibbs Free Energy= 0.357682
Sum of electronic and zero-point Energies= -3245.946641
Sum of electronic and thermal Energies= -3245.916503
Sum of electronic and thermal Enthalpies= -3245.915558
Sum of electronic and thermal Free Energies= -3246.008154

TS-I1-i-to-c

C -3.1874922995 1.0466010686 -0.0254748909
C -1.8599978135 1.0115693028 0.2950913917
C -1.2190869928 -0.1989712782 0.8692004143
C 0.0796776176 -0.4980437014 0.4815800642
O 0.6631317175 0.2807172951 -0.4819792901
C 0.4256144185 1.6213524974 -0.311447196
C -0.8438547088 2.0887992887 0.0719030039
C -3.941492575 -0.2121025349 -0.257934435
C -5.2754222347 -0.3497536981 0.1185620837
S -6.2856898199 1.0446260044 0.4843380453
C -5.3692960202 2.2142720982 -0.5564994354
C -1.7901685311 -1.0041137189 1.857613633
C -1.077791511 -2.0678573895 2.3994313663
C 0.2319044676 -2.3247976028 1.9983147635
C 1.3631229885 3.8167736838 -0.4469867087
C 0.1149437142 4.3281897565 -0.1172020978
C -0.9624999559 3.4844912104 0.1451994852
C -3.3247148638 -1.3476082641 -0.9164230293
C -3.9516333549 -2.6220421415 -0.845618798
C -5.2320998329 -2.7358139742 -0.2426008319
C -5.903742019 -1.6197208112 0.1607953643
C -2.1357049975 -1.2510817624 -1.6860523078
C -1.5614808823 -2.3563101816 -2.2617307091
C -2.1409812218 -3.6332489224 -2.1052511605
C -3.3226140471 -3.7545406155 -1.4228600092
H -5.406504564 1.8513800968 -1.5868694503
H -5.9031736554 3.1660112785 -0.4995739969
H -2.8001898472 -0.7926338304 2.1930071622

H -1.5363297029 -2.6891222506 3.1607042119
H 0.7879789842 -3.1365026885 2.4571251074
H 2.2029889575 4.482999365 -0.6161826944
H -0.0246237446 5.4009327608 -0.0411801773
H -1.8962499235 3.9421830125 0.4330885895
H -5.7017020493 -3.7131079219 -0.1795290385
H -6.9275870628 -1.6861927613 0.5152467039
H -1.677366925 -0.2813425204 -1.8361652621
H -0.6584573349 -2.2417244172 -2.8533088883
H -1.6695911179 -4.5034039758 -2.5499613385
H -3.8110729261 -4.7205795992 -1.3310429942
C 0.8388591042 -1.5214189227 1.0295311759
C 1.5261415657 2.4343007652 -0.5415217415
C -3.9447704283 3.0417314432 1.3275401882
H -4.6517952698 2.5235818195 1.9797085148
H -2.9728499371 3.0024550856 1.8198087195
H -4.2603626424 4.0879341696 1.2504557577
C -3.930499289 2.3775669018 -0.0655213444
H -3.4177018565 3.0384634091 -0.7787175379
Pd 4.1889045928 -0.0944009964 0.181314087
Cl 6.0523014094 1.2279310132 -0.2647698939
Cl 5.5350045324 -1.8271866043 0.9645856086
P 2.5794645845 -1.717249318 0.547329825
H 2.9754633192 -2.6564218506 1.4995396217
H 2.4959391479 -2.5383118454 -0.5880210101
P 3.1259096284 1.6489111429 -0.9037418635
H 2.9913718461 1.3225243417 -2.2626517963
H 3.9247089723 2.788948938 -1.0040902109

Energy= -3246.3692397
Zero-point correction= 0.419681 (Hartree/Particle)
Thermal correction to Gibbs Free Energy= 0.359610
Sum of electronic and zero-point Energies= -3245.949559
Sum of electronic and thermal Energies= -3245.920220
Sum of electronic and thermal Enthalpies= -3245.919276
Sum of electronic and thermal Free Energies= -3246.009629

TS-I1-c-to-e

C -2.9782439804 0.9537457268 -0.1836126141
C -1.7420703446 1.0831278641 0.3827436848
C -1.1319804341 0.0023143257 1.2017947113
C 0.2388217469 -0.1795654464 1.0948224647
O 0.8977878781 0.5249916114 0.1306082057

C 0.5618045315 1.8466910631 0.0823383061
C -0.7881016614 2.2315235459 0.2124108246
C -3.5674896875 -0.3910606754 -0.4063829976
C -4.9348184555 -0.6231899262 -0.2751210212
S -6.1038858382 0.6936638262 -0.2721919534
C -5.0941587356 1.8394982942 -1.2512496916
C -1.7975483492 -0.7606159955 2.1631709307
C -1.0940204649 -1.6643257505 2.9552528714
C 0.2933438412 -1.7680679964 2.8647434509
C 1.3355625738 4.087105042 -0.2041107529
C 0.0196445793 4.5137312177 -0.1043482382
C -1.0218669931 3.6072976701 0.1036220283
C -2.7416438768 -1.5190286363 -0.7935623439
C -3.2664543152 -2.8364492432 -0.6867603765
C -4.6298179282 -3.0160227561 -0.3339335649
C -5.4565612699 -1.938803915 -0.2038282175
C -1.4321574631 -1.3830605859 -1.3247421435
C -0.6620745316 -2.4774399343 -1.6279229602
C -1.1579772039 -3.7831266872 -1.4259060019
C -2.4427865803 -3.9529512633 -0.982015487
H -4.8966054006 1.3736131779 -2.2200275553
H -5.7017323232 2.734058558 -1.4086288662
H -2.8708738179 -0.6499975528 2.2791051672
H -1.6282103428 -2.2677646093 3.6808339253
H 0.8327995207 -2.4327520123 3.5324221345
H 2.1337314426 4.8076715096 -0.3547408642
H -0.2105159257 5.5708740087 -0.1799534942
H -2.020623906 4.0061859947 0.1960104031
H -5.0236612021 -4.024409188 -0.2452980919
H -6.5224257726 -2.072792801 -0.0481082157
H -1.0308061765 -0.3938137064 -1.506235391
H 0.3350598119 -2.3349006103 -2.0324335845
H -0.5334098948 -4.6410484976 -1.6511611231
H -2.8609255186 -4.9490959962 -0.8667920738
C 0.988758118 -0.9891619726 1.9365594841
C 1.6241078783 2.722904872 -0.1016895563
C -4.1400836741 2.9903729203 0.7569125207
H -4.9217573956 2.4749573821 1.3199756885
H -3.284953714 3.0938987593 1.4252838919
H -4.5112567607 3.9882522511 0.4988320872
C -3.7988770184 2.1936803858 -0.5198997549
H -3.2059449636 2.8268835859 -1.1953010296
Pd 3.9820379822 -0.147387302 0.0579183198
Cl 5.4190060222 0.3790898804 -1.7017538293

Cl 4.7493403902 -2.3394359903 0.144295132
P 3.3198159741 2.056330718 -0.2787024684
H 3.6310499391 2.4646954662 -1.577875414
H 4.0539497645 3.0093509615 0.4440160145
P 2.7936824588 -0.7803072133 1.9224651764
H 2.9421260137 0.1009490921 3.0098258615
H 3.2413032035 -1.9624021771 2.5164641816

Energy= -3246.3623784
Zero-point correction= 0.418991 (Hartree/Particle)
Thermal correction to Gibbs Free Energy= 0.358432
Sum of electronic and zero-point Energies= -3245.943388
Sum of electronic and thermal Energies= -3245.913914
Sum of electronic and thermal Enthalpies= -3245.912969
Sum of electronic and thermal Free Energies= -3246.003946

TS2-i

C -3.9715808656 -0.8712767776 -0.9152867014
C -2.6840171091 -0.7362021765 -1.377958374
C -1.8590234722 0.4470863844 -1.7461722677
C -0.6658434908 0.5920473119 -1.0525647301
O -0.3406683657 -0.3938227987 -0.1434981387
C -0.5234283084 -1.6365347047 -0.7281895426
C -1.6941859265 -1.8837355222 -1.4578448934
C -4.8744557457 0.0880568846 -0.14989864
C -6.1045349223 -0.3691769842 0.3570811937
S -6.9603398086 -1.8504195884 -0.1013103583
C -5.4899243123 -2.8682367461 -0.1690106096
C -2.0600282962 1.274803252 -2.8541727125
C -1.1511275766 2.2887839447 -3.1463533752
C -0.0015440395 2.4636050457 -2.3761177284
C 0.4630531591 -3.7295456953 -1.3440904636
C -0.6626856591 -3.9904970427 -2.1161680742
C -1.7129442475 -3.0766363788 -2.1896222403
C -4.486496655 1.4157419942 0.3473074416
C -5.1227467059 1.9863762167 1.4922812318
C -6.2950489016 1.3920744417 2.0133035388
C -6.8090841609 0.2989476452 1.398382121
C -3.5558512881 2.2693793288 -0.2848461181
C -3.1373548157 3.4658075406 0.2479659764
C -3.6497309309 3.9207557825 1.4743509487
C -4.6558233291 3.1979100482 2.0599000245
H -5.0561637458 -2.9332776202 0.832360134

H -5.7924007302 -3.871465528 -0.4802610266
H -2.9500931817 1.1397378331 -3.4610056824
H -1.3269420389 2.9382381725 -3.996593892
H 0.7196692723 3.2304228536 -2.6383524396
H 1.2966520182 -4.4234837403 -1.3326544525
H -0.7151985008 -4.9059568293 -2.6948961983
H -2.5470288498 -3.2962158364 -2.8453536415
H -6.7904318308 1.8374754892 2.8707024374
H -7.7320292259 -0.1465495345 1.7517269981
H -3.2099182078 2.0194900913 -1.2620217579
H -2.4274951149 4.0687699386 -0.3101814916
H -3.3044420134 4.851774471 1.9113851349
H -5.146596587 3.5605310837 2.9587195415
C 0.2639001479 1.5876889593 -1.3212870412
C 0.5441836428 -2.5222680634 -0.649164739
C -5.0507888946 -2.3641548832 -2.6025327248
H -5.9150940689 -1.7062354214 -2.7254492265
H -4.2912264325 -2.051027639 -3.323644655
H -5.3667742287 -3.3845645882 -2.8458190073
C -4.5019793907 -2.2956873326 -1.1666861855
H -3.6506761544 -2.9697562966 -1.1005376688
P 1.8530630844 1.4651535902 -0.469572398
P 2.0655406124 -1.8959400707 0.1015936874
Pd 3.0114453605 -0.3279669225 -1.2443508671
Cl 4.0283389691 -2.201059661 -2.2101123347
Cl 3.8076248714 1.2311672102 -2.7972884362
H 2.8145932147 -3.0490497089 0.344079614
H 1.6667468621 -1.5471027539 1.4019779455
H 1.4875337523 1.5236411269 0.8853826648
H 2.4316062126 2.7223744346 -0.6538238896

Energy= -3246.328386
Zero-point correction= 0.419721 (Hartree/Particle)
Thermal correction to Gibbs Free Energy= 0.359280
Sum of electronic and zero-point Energies= -3245.908665
Sum of electronic and thermal Energies= -3245.879437
Sum of electronic and thermal Enthalpies= -3245.878493
Sum of electronic and thermal Free Energies= -3245.969106

TS2-c

C -3.7952414272 -1.0752692646 -0.7343295221
C -2.6677216257 -0.6116206525 -1.3496235563
C -2.0231739046 0.7312096307 -1.5071716695

C -0.7936177087 0.8501428349 -0.8551463564
O -0.3097248042 -0.2721699362 -0.2107316957
C -0.3596755022 -1.3268673005 -1.1048763297
C -1.5610506484 -1.5398304935 -1.7882520216
C -4.94724928 -0.4274060353 0.0091060275
C -5.6735451288 -1.2197505969 0.9089355921
S -5.1782800996 -2.7343882469 1.6562470333
C -3.812625013 -3.2149369727 0.5890024702
C -2.3603703671 1.7286281704 -2.4247228808
C -1.5639422886 2.8616142871 -2.5585728563
C -0.3926253015 2.9942796008 -1.8181459298
C 0.7625432186 -2.9974012883 -2.3857872856
C -0.4041880178 -3.2000393042 -3.1151796424
C -1.5533858231 -2.4659542433 -2.8306701356
C -5.5103588706 0.8977053093 -0.2222497805
C -6.8632365831 1.1863242716 0.1252501226
C -7.599627723 0.2519318945 0.8943942685
C -6.9849926028 -0.8704995623 1.3439471288
C -4.761318332 1.9962132619 -0.6934025804
C -5.3226307332 3.2196761617 -0.9720189937
C -6.6979362211 3.436877076 -0.7765164626
C -7.4416444777 2.4356883938 -0.2060214795
H -2.8600417118 -2.9086314196 1.0288075602
H -3.8330527561 -4.3064235747 0.5279426691
H -3.2670896973 1.6254118157 -3.0102404157
H -1.8506899284 3.6409203322 -3.2560651165
H 0.2216667132 3.8814643664 -1.9382053934
H 1.6541429939 -3.5675115433 -2.6281075462
H -0.4152557205 -3.9257837995 -3.9209644503
H -2.4528655616 -2.6133576135 -3.4206537677
H -8.6276523831 0.4711426829 1.1666323274
H -7.5098393888 -1.5588911608 1.9987184732
H -3.6969056322 1.9003983794 -0.751400697
H -4.6843076206 4.0284458495 -1.3157300732
H -7.1469797432 4.3949736208 -1.0159527342
H -8.4879020644 2.5962059196 0.039009469
C 0.0296544188 1.9640728652 -0.971025326
C 0.8069193277 -2.0300881888 -1.3746091374
C -5.2209051562 -3.0456964854 -1.5598671343
H -5.259458457 -4.138585009 -1.6069347945
H -6.1485072394 -2.6941407861 -1.105533857
H -5.1830100194 -2.6614825526 -2.5837947399
C -3.9653625045 -2.6045959274 -0.7980841759
H -3.1285761884 -3.0181286118 -1.3484121763

P 1.6385656444 2.0295280956 -0.1248226951
P 2.3513666961 -1.6521655768 -0.4893760917
Pd 3.225783438 0.3868639839 0.097724834
Cl 4.9844737754 -1.0716755067 0.5770714905
Cl 4.3384426744 2.2731725646 0.9040302081
H 3.2376266007 -2.4215160143 -1.2444005899
H 2.2352233058 -2.4404861391 0.6612867595
H 1.2874354946 2.4914829031 1.1486575857
H 2.1476190552 3.1962299751 -0.6966386591

Energy= -3246.3312556
Zero-point correction= 0.419986 (Hartree/Particle)
Thermal correction to Gibbs Free Energy= 0.359659
Sum of electronic and zero-point Energies= -3245.911270
Sum of electronic and thermal Energies= -3245.881822
Sum of electronic and thermal Enthalpies= -3245.880878
Sum of electronic and thermal Free Energies= -3245.971597

TS2-e

C -3.9880274917 -1.086639147 -0.5845653225
C -2.7734897572 -0.6463179877 -1.0307937387
C -2.050526853 0.6708533202 -1.0018185565
C -0.9148602004 0.6652149643 -0.1834916397
O -0.6051244215 -0.5301676706 0.4215544029
C -0.5544690209 -1.5080456464 -0.5470918982
C -1.6678200619 -1.6148264137 -1.3886391103
C -5.2040011109 -0.4246481833 0.0342779072
C -6.072227103 -1.2220026739 0.7934722492
S -5.7374355818 -2.7836846767 1.5283151794
C -4.2526773538 -3.2663782643 0.6365197085
C -2.2035866359 1.755630863 -1.8643062349
C -1.3430064983 2.8503145843 -1.7842682159
C -0.2721539445 2.8481882876 -0.8979978796
C 0.6548180117 -3.1482858727 -1.7885106335
C -0.4287243032 -3.2676296126 -2.6500894016
C -1.5704690846 -2.4848858556 -2.4716074571
C -5.6862662401 0.9289343828 -0.2134348239
C -7.0636276689 1.2564701057 -0.0429019125
C -7.928451766 0.3236433898 0.5812483171
C -7.4179359828 -0.8365619383 1.0634993217
C -4.8429121931 2.0145755612 -0.5269839304
C -5.3184564818 3.2687246056 -0.8281157459
C -6.6996965091 3.5309635418 -0.8174393054

C -7.5484775065 2.5385135983 -0.3978381749
H -3.3614763482 -3.0049037159 1.2127898613
H -4.2923786607 -4.3538608084 0.5312452517
H -3.0206019973 1.7581071426 -2.5771275725
H -1.5000104086 3.7030666088 -2.4356510738
H 0.3980556497 3.7020093237 -0.8607053256
H 1.5476154399 -3.7425467622 -1.9591642775
H -0.378450783 -3.9598452035 -3.4834664459
H -2.3942357656 -2.5591444328 -3.1751221529
H -8.9759709666 0.5724086645 0.7221814319
H -8.0490190315 -1.5268113563 1.6142230099
H -3.785568282 1.8793761119 -0.4328652943
H -4.6120123536 4.0659180592 -1.0409024856
H -7.07908074 4.5141354267 -1.0751126791
H -8.6126633943 2.7304226765 -0.2921170456
C -0.0132634015 1.721767973 -0.106946805
C 0.6200449241 -2.2275352675 -0.7330566215
C -5.3612980273 -2.969509812 -1.6710449081
H -6.3284729064 -2.5956922619 -1.3320096205
H -5.1731497464 -2.5554100377 -2.6664886436
H -5.4325105929 -4.0579387809 -1.7624735231
C -4.2031656222 -2.6045484532 -0.7349335785
H -3.3165220485 -3.0324231574 -1.1868155967
P 1.5322189637 1.5445342306 0.8430277406
P 2.1037268259 -1.8206803122 0.2427180422
Pd 1.9175187299 -0.3862749846 2.0175440132
Cl 2.2563699615 -2.3061238913 3.2918815812
Cl 1.7357738744 0.982860422 3.8905099282
H 2.6396140048 -3.0739459242 0.5506892478
H 2.9760271246 -1.4354899332 -0.7917732595
H 2.4725142985 1.8782970308 -0.1478754224
H 1.5726164489 2.7100424134 1.6133263593

Energy= -3246.3212568
Zero-point correction= 0.419034 (Hartree/Particle)
Thermal correction to Gibbs Free Energy= 0.355923
Sum of electronic and zero-point Energies= -3245.902223
Sum of electronic and thermal Energies= -3245.872607
Sum of electronic and thermal Enthalpies= -3245.871663
Sum of electronic and thermal Free Energies= -3245.965333

st-I2-[ax]-i

C 3.7849843958 -1.5296695581 0.0853611178
C 2.6405008884 -1.1065101134 -0.4913856263
C 1.5044316721 -2.0187865667 -0.8033655074
C 0.2338483756 -1.5665062267 -0.4688565769
O 0.1025246196 -0.3491725045 0.1497968436
C 0.9285465506 0.6236214313 -0.3727521255
C 2.2466083571 0.3179620336 -0.7188660394
C 4.9429670627 -0.6593856189 0.3934619164
C 5.2563349518 -0.4549802178 1.7239456976
S 4.2970077739 -1.1687497006 3.0198597501
C 3.601518283 -2.6861699698 2.2425534102
C 3.9215758862 -2.8935086866 0.7483411334
C 1.5780973125 -3.2284938158 -1.5010297047
C 0.4254005372 -3.9489872301 -1.8027738102
C -0.8341630027 -3.4603429824 -1.4580249788
C 1.1221151568 2.8963660681 -1.0910447462
C 2.4505694203 2.6386007119 -1.4138939667
C 3.0014362761 1.3697397251 -1.2479278752
C 5.8419880998 -0.1965754325 -0.6189252191
C 6.9524412653 0.6092833787 -0.2444949509
C 7.1754817538 0.8821336046 1.130934864
C 6.3712950572 0.3402778025 2.0931990177
C 5.6757319475 -0.5219109672 -1.9927867631
C 6.5434707201 -0.0419638673 -2.9407218709
C 7.6271532683 0.7856719258 -2.5691814935
C 7.8259413476 1.0982232657 -1.2493428077
C 5.2787773573 -3.5616776005 0.512522365
H 3.9648780607 -3.5380510594 2.822766566
H 2.519678995 -2.611351468 2.3680755967
H 3.1445606695 -3.5664317922 0.382057851
H 2.5465359586 -3.5924462989 -1.8305305314
H 0.5049187509 -4.8833733193 -2.3470712868
H -1.7284023933 -4.0000715067 -1.7509579557
H 0.6877873955 3.8719530426 -1.2816131875
H 3.062946909 3.4291214762 -1.8331065598
H 4.0218477031 1.1976226073 -1.5592886101
H 8.0236716581 1.4991013637 1.4132463298
H 6.5807476254 0.5053792271 3.1451861943
H 4.8417394677 -1.1510507341 -2.2862705051
H 6.3981940544 -0.3008540406 -3.9846544476
H 8.3029654373 1.1634071753 -3.3293450297
H 8.6644282522 1.7214568941 -0.9510854118
H 5.2838230885 -4.5647583114 0.9489345689

H 6.0976885033 -2.9927416318 0.9595708854
H 5.4813455305 -3.6545617385 -0.5586681494
C -0.9393755631 -2.2416825401 -0.7889478636
C 0.3407859951 1.8689184302 -0.5651818466
Pd -2.5449342613 0.5672248826 -1.7705972571
Cl -3.4644419742 -0.8886788165 -3.3557881315
Cl -2.4374324919 2.4381587343 -3.1666962337
P -2.4776840236 -1.3278453211 -0.5115732719
H -3.4705455504 -2.2825111489 -0.7409533588
H -2.5269107453 -1.2178233811 0.8889882469
P -1.4505812252 1.9471952927 -0.3253690416
H -1.6017880285 1.7355178068 1.0557474785
H -1.7335950621 3.3111731677 -0.4211788013

Energy= -3246.3858603
Zero-point correction= 0.419711 (Hartree/Particle)
Thermal correction to Gibbs Free Energy= 0.356598
Sum of electronic and zero-point Energies= -3245.966149
Sum of electronic and thermal Energies= -3245.935708
Sum of electronic and thermal Enthalpies= -3245.934764
Sum of electronic and thermal Free Energies= -3246.029263

st-I2-[ax]-c

C 3.7809670891 -1.4881954813 -0.1667340371
C 2.6704909566 -1.0298091782 -0.7815512039
C 1.5617593001 -1.9155408842 -1.2271348407
C 0.2696609669 -1.4967009854 -0.9109218826
O 0.1172400521 -0.3515951455 -0.1776272316
C 0.9348018891 0.6683741021 -0.6041588961
C 2.2722325632 0.4015910126 -0.9270548314
C 4.9072348813 -0.6345476439 0.2755132837
C 5.1347589811 -0.5240518612 1.634394886
S 4.1155076893 -1.341836904 2.8170572671
C 3.4615821559 -2.7892293783 1.886126939
C 3.8848518432 -2.8942965197 0.4073426237
C 1.6939695412 -3.0558772559 -2.0200299958
C 0.5681446035 -3.759165465 -2.4410924074
C -0.7072885274 -3.3089090874 -2.1132731457
C 1.1571221639 2.9839555261 -1.137904943
C 2.4935809289 2.7608444254 -1.4429546667
C 3.0435191354 1.4837844218 -1.3525560715
C 5.8603431142 -0.0905421388 -0.6424662249
C 6.931427249 0.7007104645 -0.1431898051

C 7.0630911511 0.8782715722 1.2594108172
C 6.2107316364 0.2579090685 2.1279923512
C 5.788937214 -0.3233904387 -2.0430672392
C 6.7075554418 0.2310361568 -2.8980139397
C 7.7502907716 1.0457963605 -2.4019224755
C 7.8584564107 1.2691913655 -1.053659741
C 5.2605099223 -3.5390989841 0.2193651726
H 3.7773962224 -3.6840086333 2.4283790792
H 2.3741862516 -2.7104256923 1.9409248268
H 3.1398727001 -3.5434452974 -0.056037343
H 2.6851160257 -3.3797862644 -2.3230987246
H 0.684263503 -4.6468856984 -3.0530142128
H -1.577212275 -3.8468239821 -2.4780503177
H 0.7353789835 3.9790084472 -1.2429770911
H 3.1151499865 3.5847700951 -1.7758273203
H 4.0754716171 1.3327497053 -1.6365940646
H 7.8805643961 1.4850674982 1.6379261393
H 6.3514933928 0.3503636378 3.2001470759
H 4.9876937895 -0.9437858418 -2.431364794
H 6.6358044423 0.0402860873 -3.9641230513
H 8.4673373943 1.48306677 -3.088889676
H 8.6655005557 1.8806855341 -0.6596945498
H 5.2458614072 -4.5685091155 0.5890653814
H 6.0430059746 -2.9967997362 0.7557584172
H 5.5341349138 -3.560781096 -0.8398329754
C -0.875464232 -2.147984427 -1.3505433975
C 0.3492451922 1.9213356282 -0.7205475711
Pd -3.2124013108 0.7196219618 -0.6899320167
Cl -4.2629619873 2.7478270479 -0.2270909349
Cl -5.2277126657 -0.4544739163 -0.7856530896
P -2.528243545 -1.4630839507 -0.9971931826
H -3.2556247809 -2.1160282463 -1.9934015096
H -2.9146231703 -2.20375004 0.1260731503
P -1.425593668 2.1494688125 -0.3821924764
H -1.4254216864 2.5544759278 0.9581144771
H -1.6171375915 3.3768470802 -1.0179979097

Energy= -3246.3788104
Zero-point correction= 0.419494 (Hartree/Particle)
Thermal correction to Gibbs Free Energy= 0.356118
Sum of electronic and zero-point Energies= -3245.959316
Sum of electronic and thermal Energies= -3245.928610
Sum of electronic and thermal Enthalpies= -3245.927666
Sum of electronic and thermal Free Energies= -3246.022692

st-I2-[ax]-e

C 3.8922065492 -1.5174934408 -0.1540398025
C 2.7661631953 -1.0505407216 -0.7363250114
C 1.6544991454 -1.945767967 -1.1707983491
C 0.3680491515 -1.5340173469 -0.8294901527
O 0.2437077076 -0.4088025976 -0.0737638683
C 1.0258714082 0.6342631401 -0.4866871048
C 2.3618101376 0.3892536369 -0.8324579876
C 5.0385898879 -0.6732588057 0.250770187
C 5.3429651502 -0.5909341958 1.5971285312
S 4.4197826738 -1.4433252858 2.8300108186
C 3.6260669785 -2.8156365787 1.8995904329
C 3.9999488107 -2.9274277496 0.4092304254
C 1.7641485811 -3.0868519482 -1.9660226359
C 0.6248744395 -3.7919389766 -2.3540111601
C -0.646164481 -3.3344867243 -2.0159353331
C 1.2054641861 2.9727846046 -0.9229572987
C 2.5459687876 2.7769897974 -1.2299506382
C 3.1136584621 1.5020157009 -1.2114784166
C 5.935955218 -0.1029963239 -0.7084435666
C 7.029257248 0.6851315169 -0.2545883285
C 7.2376528615 0.8360198281 1.1420003659
C 6.43959951 0.192263929 2.0434626532
C 5.7878601516 -0.3059317581 -2.1077317043
C 6.6541190994 0.2725252006 -3.0009053
C 7.7184899195 1.0836123313 -2.5475987484
C 7.9004091435 1.2789841044 -1.2027619574
C 5.357749912 -3.5917291803 0.1703461637
H 3.8957574961 -3.7380421626 2.4202887486
H 2.5486196015 -2.664497162 1.9883903286
H 3.2279389852 -3.5639560089 -0.0262977542
H 2.7456384296 -3.4189160396 -2.291254486
H 0.7284781307 -4.6892515944 -2.9541590045
H -1.5235108348 -3.8721871947 -2.3623119544
H 0.7729973343 3.9671185948 -0.9797322735
H 3.1609119396 3.6241574569 -1.5132017227
H 4.1459386015 1.3845100112 -1.5090820315
H 8.0699704064 1.4427007377 1.4868605981
H 6.6387006112 0.2669504901 3.1077902386
H 4.9697023103 -0.9228137919 -2.4645560998
H 6.5233774914 0.1031429813 -4.0650891723
H 8.3935453261 1.5398993642 -3.264111816
H 8.7247671221 1.8873616583 -0.8410681568
H 5.3429445287 -4.6194174737 0.5446848771

H 6.168690082 -3.0592621048 0.6730877541
H 5.588708027 -3.6210335135 -0.8988715621
C -0.7932798098 -2.1648765395 -1.2636600448
C 0.4111308945 1.8792708212 -0.5648519332
Pd -2.5012388079 0.2533833444 0.6753150037
Cl -2.6163304453 1.7457567477 2.4580649602
Cl -3.5390433755 -1.4722215464 1.851458401
P -2.3881901524 -1.3219144401 -0.9918210022
H -2.6620699184 -0.8926781649 -2.3048842283
H -3.2951081783 -2.379994159 -0.8952711706
P -1.3998310704 1.9804368519 -0.3697014767
H -1.587639433 3.242301396 0.1994771916
H -1.7776145174 2.2601339309 -1.6969853461

Energy= -3246.3705523
Zero-point correction= 0.419033 (Hartree/Particle)
Thermal correction to Gibbs Free Energy= 0.354873
Sum of electronic and zero-point Energies= -3245.951520
Sum of electronic and thermal Energies= -3245.920836
Sum of electronic and thermal Enthalpies= -3245.919892
Sum of electronic and thermal Free Energies= -3246.015680

TS-I2-i-to-c

C -2.8490106674 1.0437996661 -0.0227845521
C -1.6079727552 0.6812465284 -0.4097837656
C -0.556532986 1.6608434908 -0.7957694891
C 0.7130896318 1.4529219973 -0.2618498634
O 0.8974404621 0.4160584277 0.6133936727
C 0.2697863099 -0.7396382308 0.2070070193
C -1.0240654546 -0.6904406012 -0.3233648824
C -3.9230961963 0.0942298251 0.3475444652
C -4.3555878947 0.0927164092 1.6603652542
S -3.6465941347 1.1567157112 2.8737287386
C -3.0254243984 2.5777301612 1.881781046
C -3.2100529385 2.4736422749 0.3547093803
C -0.6974519771 2.691314776 -1.7276071522
C 0.3895373902 3.4913502446 -2.0693284506
C 1.6470651387 3.247667594 -1.522647732
C 0.4192271454 -3.1071356233 -0.0582483597
C -0.8740042905 -3.1007161672 -0.5681807644
C -1.5830247423 -1.9101395257 -0.7122080912
C -4.6416526848 -0.6650951743 -0.6294631331
C -5.6797189075 -1.5387513742 -0.2031313148

C -6.018217389 -1.5880406743 1.1749510981
C -5.3986327472 -0.7729054705 2.0791236247
C -4.368231565 -0.5702855818 -2.0213095434
C -5.0619825373 -1.3290948237 -2.9296253232
C -6.0693963027 -2.2219403405 -2.4993255883
C -6.3711194935 -2.3180665282 -1.1655183197
C -4.6023747669 2.9040719212 -0.1139795754
H -3.5295844545 3.4715893486 2.2575996113
H -1.9601149444 2.6539854946 2.1075075729
H -2.4803337229 3.1667500399 -0.067473071
H -1.6615487883 2.850349496 -2.2011572413
H 0.2640161964 4.2909655144 -2.7909726488
H 2.494376523 3.8538464767 -1.8278533238
H 0.9707645427 -4.0388343691 0.0195829196
H -1.3340622935 -4.0324139289 -0.8783519108
H -2.5706025753 -1.9357706617 -1.1506416248
H -6.8099648377 -2.2577541354 1.4979766385
H -5.7016831608 -0.7735896754 3.1212448467
H -3.5920388702 0.1089585052 -2.3585271575
H -4.8381949107 -1.2414960525 -3.9880857857
H -6.6071350449 -2.8205820213 -3.2271268977
H -7.1545489 -2.9894205711 -0.8250080718
H -4.7690146176 3.9585404734 0.124551885
H -5.3930374223 2.3192067229 0.3623175373
H -4.6998455025 2.7795729885 -1.1966651047
C 1.8285565003 2.2001395818 -0.6146610129
C 1.0139211725 -1.9038783 0.3286954744
Pd 4.4155107123 -0.3800144906 0.1925223889
Cl 5.6867033578 -2.2987967576 0.5309904509
Cl 6.3287685771 0.8855515303 -0.2258915404
P 3.4702256815 1.7232262164 0.0095563965
H 4.2553295353 2.6016178565 -0.7374704528
H 3.5154891195 2.3556709971 1.2615735646
P 2.7324166186 -1.7949936859 0.9049207735
H 2.5805679213 -1.6600788802 2.2937570655
H 3.0921525088 -3.140692435 0.8240930399

Energy= -3246.3777993
Zero-point correction= 0.419527 (Hartree/Particle)
Thermal correction to Gibbs Free Energy= 0.357817
Sum of electronic and zero-point Energies= -3245.958272
Sum of electronic and thermal Energies= -3245.928417
Sum of electronic and thermal Enthalpies= -3245.927473
Sum of electronic and thermal Free Energies= -3246.019983

TS-I2-c-to-e

C -2.8520292031 1.0741430046 0.156358205
C -1.6472453389 0.7454811828 -0.3567380924
C -0.6178205673 1.757291582 -0.7274967291
C 0.6853742069 1.4715399551 -0.3279212396
O 0.8872117286 0.3830409894 0.4687176858
C 0.2463859494 -0.7418106909 0.0237172857
C -1.0761160001 -0.6378817865 -0.4331465066
C -3.9122517209 0.0991175454 0.4971534524
C -4.2708333672 -0.0298454718 1.8261627922
S -3.5047922346 0.9106016344 3.1030524491
C -2.8595080849 2.3888641351 2.218927154
C -3.1578776655 2.4588767889 0.7087722988
C -0.8014109295 2.8737695713 -1.5443301581
C 0.2836814467 3.6703623294 -1.9088846029
C 1.5799232901 3.3180942582 -1.5412615913
C 0.3402200798 -3.0854850493 -0.4041571374
C -0.9712189322 -3.0246916281 -0.8554788089
C -1.6705214713 -1.8166994736 -0.8814118104
C -4.6895668488 -0.5606555131 -0.5075252536
C -5.7068892424 -1.4721285274 -0.111549291
C -5.9660422253 -1.6577748328 1.2722334269
C -5.2922129151 -0.9353521457 2.2149869342
C -4.4967553991 -0.3281855506 -1.8967213517
C -5.2460477508 -0.9929648203 -2.8343639744
C -6.2318343872 -1.924456939 -2.43778881
C -6.4566660208 -2.15221663 -1.1046403076
C -4.5701832923 2.9563358942 0.3918677743
H -3.2825726632 3.2618238558 2.7224198997
H -1.7784472951 2.3856377572 2.3700161482
H -2.4446469375 3.1847932302 0.3142763815
H -1.7959791262 3.1093956623 -1.9111841539
H 0.12183134 4.5477004496 -2.5254612528
H 2.4209574248 3.910656944 -1.887992698
H 0.871516754 -4.0326661309 -0.4047984977
H -1.4616183251 -3.9266761727 -1.2047821802
H -2.6797713859 -1.8022615454 -1.268406751
H -6.74060762 -2.3573732093 1.572810462
H -5.5348759225 -1.0404947667 3.2676213471
H -3.7381931119 0.3819115107 -2.209374098
H -5.08359676 -0.7995316863 -3.8900331692
H -6.8142186138 -2.4477109324 -3.1889934857
H -7.2232209796 -2.8537446687 -0.7872738745
H -4.6990725458 3.9780896049 0.7605040385

H -5.3378488696 2.3299409938 0.8526333519
H -4.7438929878 2.9586861737 -0.6884946602
C 1.7998706204 2.1760101525 -0.765233679
C 0.9809074407 -1.9208347931 0.0339912031
Pd 3.9138352325 -0.0155414825 1.2047920196
Cl 4.6497823888 -1.3803359694 2.9416720538
Cl 5.1583707892 1.7918107082 1.9774833503
P 3.4370259275 1.4139498607 -0.5289891706
H 3.6129810504 0.8546746859 -1.8089015398
H 4.2967859445 2.5097333516 -0.6345599026
P 2.7103504745 -1.9120300156 0.6229976636
H 2.6573335433 -2.8235062738 1.6804483275
H 3.3224594093 -2.7191097964 -0.3494013869

Energy= -3246.3696787
Zero-point correction= 0.418870 (Hartree/Particle)
Thermal correction to Gibbs Free Energy= 0.356657
Sum of electronic and zero-point Energies= -3245.950808
Sum of electronic and thermal Energies= -3245.920863
Sum of electronic and thermal Enthalpies= -3245.919918
Sum of electronic and thermal Free Energies= -3246.013022

TS3-i-to-e

C 2.8570078039 1.3986338808 -0.4573738444
C 1.5637508371 1.2829931676 -0.8790058414
C 0.4524976086 2.2157883738 -0.4846386546
C -0.8068287507 1.6324800746 -0.2910338312
O -1.1520909592 0.4319821112 -0.8149905795
C -0.202262434 -0.4424835381 -1.2189068002
C 1.1070958924 -0.035486199 -1.4168949917
C 3.688286352 0.2029610311 -0.1674002575
C 4.9740229076 0.0999622326 -0.6624569049
S 5.7275197105 1.3495502751 -1.6516809948
C 4.644011903 2.7977523068 -1.3860141501
C 3.62792179 2.6836930987 -0.2334047802
C 0.5210966312 3.5425385594 -0.020016964
C -0.5210018487 4.1493832208 0.6756284897
C -1.6887912759 3.4554604609 0.9846723292
C 0.2005467906 -2.685116285 -1.9174584901
C 1.4732608772 -2.2715227943 -2.314662898
C 1.9186407109 -0.9775239654 -2.0671536105
C 3.2023440374 -0.8076054508 0.7397695865
C 3.9724173862 -1.9860088111 0.9331953602

C 5.2484543727 -2.0868748758 0.31727137
C 5.7574177896 -1.0584754343 -0.4225776285
C 1.982223409 -0.6878555057 1.4591300246
C 1.5218797041 -1.7035868548 2.2580296188
C 2.2593021124 -2.9010336903 2.3884826873
C 3.4673362866 -3.0274729502 1.753962088
C 4.2556475953 2.7909933196 1.1589031515
H 5.3107236708 3.6441850345 -1.1993673605
H 4.1057540648 2.9785880623 -2.3184386867
H 2.9874164221 3.5486874734 -0.3804717655
H 1.4058273354 4.1371866361 -0.1866451907
H -0.3965323215 5.1707986589 1.0177828406
H -2.4569420249 3.9060853682 1.6037506041
H -0.1037640373 -3.720026814 -2.0328092697
H 2.1435672076 -2.9797924058 -2.7880980438
H 2.9296102862 -0.7052728947 -2.3445870365
H 5.8364843773 -2.9868884713 0.4738983315
H 6.7554831643 -1.122867073 -0.8450350866
H 1.405162412 0.2268779529 1.3953447683
H 0.5819443245 -1.5903744628 2.7890552057
H 1.8686461495 -3.7036896356 3.0046767252
H 4.0634939042 -3.9282431339 1.873519408
H 4.7742087814 3.7484343572 1.2665196875
H 4.9749019503 1.9903792978 1.3440828564
H 3.4794066331 2.7318219515 1.927067297
C -1.8368313084 2.1699011058 0.4810370846
C -0.670431117 -1.745558408 -1.3758164281
Pd -2.5207497624 -1.1176221067 1.4325819352
Cl -2.3985105562 -0.142620578 3.5506410607
Cl -1.7683594252 -3.2230083449 2.1089257357
P -3.1902169021 1.0018434398 0.8268966731
H -3.9803932975 1.705437475 1.7395611143
H -3.9822229925 1.1053299919 -0.3318521139
P -2.3156781521 -2.0401752852 -0.6653485156
H -3.1847624592 -1.5784940498 -1.671531073
H -2.4801375669 -3.4213869 -0.7911462416

Energy= -3246.3688465
Zero-point correction= 0.418873 (Hartree/Particle)
Thermal correction to Gibbs Free Energy= 0.358308
Sum of electronic and zero-point Energies= -3245.949974
Sum of electronic and thermal Energies= -3245.920279
Sum of electronic and thermal Enthalpies= -3245.919335
Sum of electronic and thermal Free Energies= -3246.010539

TS3-c-to-c

C -2.9590287651 1.0651351101 0.5030069632
C -1.666082676 0.7493811824 0.1881167482
C -0.6587242645 1.7635741525 -0.2416258956
C 0.6583571546 1.3601315963 -0.4942445533
O 1.0215684878 0.0656049688 -0.493941461
C 0.2101210482 -0.8908114406 0.0011139406
C -1.1308480458 -0.6438835515 0.3067677782
C -4.076506114 0.0979311822 0.393881502
C -5.014351942 -0.0324246911 1.4064861048
S -4.9974638387 0.8912109613 2.9032804795
C -3.6882991448 2.1137147002 2.5992987944
C -3.3760009396 2.3942523876 1.119189348
C -0.9229526092 3.10185711 -0.5847714353
C 0.083856752 3.9848778966 -0.9580368354
C 1.4073979144 3.5613017346 -1.0413176066
C 0.1435821924 -3.1599357617 0.7411439522
C -1.1923196832 -2.9629086702 1.0814939659
C -1.8051353657 -1.7320802049 0.8886820777
C -4.2755181722 -0.6488490536 -0.8300771426
C -5.3322895543 -1.5980577324 -0.9054642278
C -6.2137346636 -1.7468614569 0.1985819126
C -6.0798106503 -0.9669362129 1.307228125
C -3.4662787208 -0.4788821079 -1.9847165245
C -3.6689024954 -1.2292498285 -3.1162491354
C -4.6927152941 -2.1975410918 -3.1672161956
C -5.5117334613 -2.3676804344 -2.0816541018
C -4.5103478165 3.1090275606 0.3823461527
H -4.0280635608 3.0346007046 3.0819860869
H -2.778747197 1.7771966426 3.1004793699
H -2.5147056251 3.0589209009 1.1778520613
H -1.9409733216 3.4652155664 -0.5977620868
H -0.1717070609 5.0113595934 -1.1964574702
H 2.1922392076 4.2646538149 -1.3033560834
H 0.6175776712 -4.1209403538 0.9176816096
H -1.7606640569 -3.7716132739 1.5271994752
H -2.8280200339 -1.6160659064 1.21675953
H -7.0166984977 -2.4756309059 0.136788963
H -6.7788698125 -1.0593417341 2.1329414815
H -2.6810366448 0.265964373 -1.9809574861
H -3.0362486605 -1.0694156072 -3.9836396841
H -4.8375257717 -2.7899002476 -4.0646410067
H -6.3209425988 -3.0922163936 -2.1080378272
H -4.7683562259 4.0443985818 0.8878524945

H -5.4095049001 2.4902912888 0.3325336972
H -4.2174171134 3.3439873523 -0.6458137422
C 1.7045521849 2.2175860417 -0.8307515727
C 0.8701792349 -2.1021937074 0.2037680798
Pd 4.2622610056 -0.6025447326 -0.6578347849
Cl 5.5043414139 -2.5668470918 -0.526402325
Cl 6.1408168434 0.6722680355 -1.1821151359
P 3.4051362287 1.5466691739 -0.9865832026
H 3.7363697991 1.9775296819 -2.2749791064
H 4.1037959634 2.4904683811 -0.2292024841
P 2.6573072151 -2.2327949096 -0.1798285468
H 3.0631026034 -3.050985527 0.8763704699
H 2.6472275582 -3.1673323371 -1.221404834

Energy= -3246.3638269
Zero-point correction= 0.419312 (Hartree/Particle)
Thermal correction to Gibbs Free Energy= 0.357669
Sum of electronic and zero-point Energies= -3245.944515
Sum of electronic and thermal Energies= -3245.914532
Sum of electronic and thermal Enthalpies= -3245.913588
Sum of electronic and thermal Free Energies= -3246.006158

TS3-e-to-i

C -2.4606753033 1.0374306044 0.2096768173
C -1.3123396322 0.5859658292 -0.3668362795
C -0.305128513 1.5397517129 -0.9213206859
C 1.0274661243 1.1565647137 -0.9822249392
O 1.4003680476 -0.0875690548 -0.6533063521
C 0.5126685417 -1.0768161219 -0.4259005802
C -0.8630559486 -0.8483399633 -0.3510808353
C -3.6531318165 0.201240542 0.4674167746
C -4.2047135643 0.1500616132 1.7361083913
S -3.5569279202 1.0230215592 3.1169446125
C -2.4312852902 2.236873907 2.3450872912
C -2.5889447503 2.4266738519 0.8261549676
C -0.5707536106 2.8021945343 -1.4762139216
C 0.4569751994 3.6376656423 -1.9066074293
C 1.7943460916 3.2357438986 -1.8455850297
C 0.3729210012 -3.4162200809 0.0115925836
C -1.0105943785 -3.2533184164 0.0786649922
C -1.6137256236 -2.0108583081 -0.0976247081
C -4.3543093005 -0.4297697698 -0.6229741421
C -5.5132827102 -1.2084205541 -0.3495139415

C -5.9843300166 -1.3029715565 0.9876962994
C -5.366684516 -0.6251550853 1.9960610254
C -3.9325337095 -0.3218536612 -1.9751452573
C -4.6076378302 -0.9642481195 -2.9829154901
C -5.7435731405 -1.7529515827 -2.7023991536
C -6.1859474503 -1.8654771573 -1.4097505442
C -3.8603513366 3.1796286482 0.4303295203
H -2.622744933 3.1851380008 2.8549243526
H -1.4081953355 1.9257015307 2.5633090743
H -1.7282541935 3.0375411733 0.5543509753
H -1.5987030798 3.1332079177 -1.5805612077
H 0.212426021 4.615693763 -2.3059514128
H 2.5825105262 3.9072208587 -2.1702613037
H 0.8225754089 -4.3915536845 0.1659856772
H -1.6398648038 -4.1125121918 0.2824752099
H -2.6881811998 -1.9577720395 -0.021809505
H -6.8657136811 -1.9034708097 1.1934935523
H -5.756867654 -0.6684397377 3.0081787715
H -3.0640595472 0.2807562121 -2.2124385112
H -4.2651353484 -0.861282181 -4.0077932083
H -6.2654300759 -2.259136501 -3.5078533231
H -7.066334001 -2.4581788946 -1.1770782502
H -3.8772017285 4.1685797986 0.8977370268
H -4.7632003926 2.6445782375 0.7345196285
H -3.9051905006 3.3137385369 -0.6551598619
C 2.0933553322 1.9529551342 -1.3990902707
C 1.1584216511 -2.3022625783 -0.255763675
Pd 4.0610857927 -0.3706893156 0.3937655829
Cl 4.3162704664 -1.736966846 2.2604756112
Cl 5.0136252094 1.5079149382 1.3929636895
P 3.7256190686 1.1237562039 -1.3512188238
H 3.7799536827 0.5986181608 -2.6576309449
H 4.6190315634 2.1897925894 -1.4790462604
P 2.979044559 -2.2464908798 -0.44316051
H 3.3761229721 -3.4951780982 0.0407766561
H 3.1017675768 -2.4688779229 -1.8290267253

Energy= -3246.3660298
Zero-point correction= 0.418909 (Hartree/Particle)
Thermal correction to Gibbs Free Energy= 0.356651
Sum of electronic and zero-point Energies= -3245.947121
Sum of electronic and thermal Energies= -3245.917182
Sum of electronic and thermal Enthalpies= -3245.916238
Sum of electronic and thermal Free Energies= -3246.009379

TS4-i-to-e

C 2.7486584406 0.9417259443 -0.0085636555
C 1.4371724197 0.6035145985 0.1906139114
C 0.8267939522 -0.7169186162 -0.1967041253
C -0.5585631375 -0.7434078634 -0.3795591845
O -1.3417998533 0.3283201091 -0.1412977258
C -0.8996397397 1.3986146075 0.5303616865
C 0.4585653118 1.5820588746 0.7506766295
C 3.7980138936 -0.1063527424 -0.0097095789
C 4.6913024665 -0.2224945053 -1.0632643974
S 4.7598720545 1.0760670337 -2.2552567654
C 4.5930830114 2.3716104559 -0.9857948442
C 1.460653377 -1.9145542484 -0.5828198888
C 0.7502289219 -3.0049824746 -1.0738643537
C -0.6377058232 -2.9790866284 -1.2083990579
C -1.5625504467 3.3346724259 1.7432168802
C -0.2155502586 3.5238526507 2.0656301771
C 0.767222263 2.6548228402 1.6026351088
C 3.9307763517 -1.0088199108 1.1083417762
C 4.854935754 -2.0892120595 1.0190002087
C 5.6545004079 -2.2304264119 -0.1464568692
C 5.6020929446 -1.3019071112 -1.1473202565
C 3.1736194552 -0.8825806123 2.3038811185
C 3.3116203412 -1.781602072 3.3309272373
C 4.2109744265 -2.8646643654 3.2259720758
C 4.9682235958 -3.0082532764 2.0934581105
H 5.3844948186 2.2011625779 -0.2524701229
H 4.7805713852 3.3286842776 -1.4774976105
H 2.5324553282 -2.0092600442 -0.5199654302
H 1.2984936482 -3.8954422383 -1.3606284872
H -1.1730913196 -3.8423227018 -1.5898188716
H -2.3146743309 4.0259758921 2.1094066518
H 0.0687194857 4.3566448751 2.6992300139
H 1.7944259956 2.814603484 1.9070598651
H 6.344875076 -3.0665830848 -0.2108081438
H 6.2612048961 -1.370479103 -2.0064159015
H 2.4799323395 -0.0572674568 2.4078460321
H 2.7259528701 -1.6557181264 4.2360641719
H 4.3060872634 -3.5718114228 4.0434596642
H 5.675030569 -3.8282596589 2.0019832894
C -1.3132354225 -1.8212428199 -0.8482991094
C -1.9251568794 2.2304382199 0.9800199143
C 2.2335246394 3.1118649317 -1.2860868751
H 2.1684896293 2.5713205084 -2.2346357621

H 1.2262459938 3.2112484993 -0.8866734592
H 2.6066213549 4.1200455827 -1.4899990242
C 3.1997555156 2.3841021377 -0.3367652507
H 3.3180377629 2.9744728946 0.5804057167
Pd -3.8933799803 0.5547446529 -1.4479720199
Cl -4.536608329 2.6761054818 -2.1680400601
Cl -4.0812488322 -0.4242074673 -3.5507729741
P -3.1249598048 -1.5563273339 -0.8704409786
H -3.5822120432 -2.6374765998 -1.6274289771
H -3.4605045093 -2.0044891208 0.4226347542
P -3.6184582863 1.6629022543 0.5716970299
H -3.9223891959 0.9356113087 1.7397644941
H -4.3883557683 2.8074029582 0.7916592431

Energy= -3246.3565523
Zero-point correction= 0.418866 (Hartree/Particle)
Thermal correction to Gibbs Free Energy= 0.357220
Sum of electronic and zero-point Energies= -3245.937687
Sum of electronic and thermal Energies= -3245.907984
Sum of electronic and thermal Enthalpies= -3245.907040
Sum of electronic and thermal Free Energies= -3245.999333

TS4-c-to-c

C 3.1509628215 1.1055589119 -0.130188397
C 1.7925776043 0.9369350072 -0.2588751488
C 1.2034224728 -0.3542506317 -0.7407810092
C -0.1811691019 -0.5344574125 -0.7278589574
O -1.0283482571 0.454088165 -0.3876708648
C -0.5870599814 1.6210686418 0.1080471814
C 0.7740426536 1.9529032785 0.1567494772
C 4.0194946996 -0.0776429537 0.1090562777
C 5.1723676297 -0.2913865319 -0.6330785262
S 5.7704655714 1.0005386558 -1.6742795061
C 5.3713268499 2.2959632981 -0.4638021339
C 1.9131406415 -1.4091855997 -1.34316087
C 1.2919795861 -2.5809710391 -1.7523413096
C -0.0816665928 -2.7538213718 -1.6004517442
C -1.3088101008 3.6361311178 1.1740811106
C 0.0288561832 4.0077489514 1.2740973785
C 1.0355849864 3.180322065 0.794590672
C 3.677536305 -1.0402979579 1.1299104652
C 4.4007610371 -2.2642263929 1.1972427655
C 5.4909672458 -2.4824361891 0.314177383

C 5.8988160444 -1.5018878127 -0.5454475665
C 2.644076835 -0.8312716525 2.0822607947
C 2.3235219718 -1.7913083972 3.0078215069
C 3.0147966256 -3.0220379015 3.0388116022
C 4.0377698007 -3.2450316574 2.1560088792
H 5.8772698727 2.0304194087 0.4670781708
H 5.8003385593 3.2299595197 -0.8342158786
H 2.9776816326 -1.3178532582 -1.5011388633
H 1.8909394293 -3.3691166998 -2.1945671854
H -0.5531766739 -3.6868103382 -1.8949390161
H -2.0886908475 4.2831656402 1.5644103003
H 0.2959955785 4.9458087171 1.7478814059
H 2.0521712839 3.5095744941 0.9367920118
H 6.0306895264 -3.4235019784 0.3680367036
H 6.7775110424 -1.6359233267 -1.1678966844
H 2.1061013803 0.1087365521 2.0851421734
H 1.5352091908 -1.5994392084 3.7288238313
H 2.7452456294 -3.7772179209 3.7698207386
H 4.5963558845 -4.1765112458 2.1815299225
C -0.8435924451 -1.7053904052 -1.0962565953
C -1.628142702 2.4151687728 0.5915450197
C 3.306216739 3.2972544368 -1.4338290687
H 3.5577377978 2.8055948655 -2.3778596892
H 2.2245830927 3.4210502714 -1.4047759195
H 3.759191068 4.2936835206 -1.4335850963
C 3.8576735639 2.465349345 -0.2643990866
H 3.7704020352 3.0308262357 0.6732785206
Pd -4.2301989192 -0.2920559043 -0.0774455651
Cl -6.1003397614 0.92876413 0.5870222906
Cl -5.5057162184 -2.1760223932 -0.5704797952
P -3.3545954233 1.7985160763 0.4923619432
H -3.8202703705 2.218063436 1.7404157873
H -3.9361172631 2.7754214539 -0.3231869486
P -2.6616783146 -1.8191957983 -0.8970164063
H -3.0422334102 -2.2240513931 -2.1800075152
H -2.7590359289 -3.0375311762 -0.2195760458

Energy= -3246.3577517
Zero-point correction= 0.419224 (Hartree/Particle)
Thermal correction to Gibbs Free Energy= 0.357942
Sum of electronic and zero-point Energies= -3245.938528
Sum of electronic and thermal Energies= -3245.908800
Sum of electronic and thermal Enthalpies= -3245.907856
Sum of electronic and thermal Free Energies= -3245.999809

TS4-e-to-i

C -2.8717206692 0.9178083734 -0.1249777574
C -1.701863131 1.1365463482 0.55099544
C -1.1052364212 0.0480506348 1.3769078178
C 0.2705330385 -0.0859681768 1.3981354734
O 1.0336340833 0.8033563341 0.7403919815
C 0.5736287416 2.0228036794 0.3995521346
C -0.7960999529 2.3179626044 0.3566365615
C -3.283756499 -0.4725007439 -0.4464109277
C -4.6081249421 -0.8818585314 -0.3535141791
S -5.8976335162 0.3145189744 -0.2217774643
C -5.0492825152 1.5689407466 -1.2294022639
C -1.8096910598 -0.8933379266 2.1372580811
C -1.1425362075 -1.9331069816 2.7787998117
C 0.2481781341 -2.061016871 2.7109131968
C 1.2852454512 4.1741307824 -0.3434331374
C -0.0601708023 4.5345002514 -0.3837609492
C -1.0725493761 3.6383088869 -0.0411815983
C -2.3075021591 -1.4440941262 -0.8920397821
C -2.6615368433 -2.821116629 -0.9092070048
C -4.0007527698 -3.1978367702 -0.6263264744
C -4.963499726 -2.2500700474 -0.4210410478
C -1.002070512 -1.0979487774 -1.3336763102
C -0.0779199775 -2.0556039268 -1.6661051928
C -0.4102148625 -3.4261912589 -1.5873687771
C -1.6828842751 -3.795185088 -1.2395430578
H -4.835983801 1.1220495363 -2.2033250745
H -5.7545518173 2.3919822211 -1.368792149
H -2.8915116363 -0.8318660295 2.1890399941
H -1.7157643029 -2.6654982621 3.3361557034
H 0.7429284596 -2.8935130417 3.2006717897
H 2.0552190074 4.8851590933 -0.6240960543
H -0.3344092493 5.5369329176 -0.6935558998
H -2.0900375716 3.9963897199 -0.0900037592
H -4.26470262 -4.2515607244 -0.6417620305
H -6.0056031929 -2.530839336 -0.3063174131
H -0.7277392245 -0.0533970684 -1.4221332276
H 0.9171744151 -1.7625530304 -1.987212402
H 0.3388939447 -4.1724993163 -1.8294618457
H -1.9696559811 -4.8431904451 -1.2212064637
C 0.987163335 -1.1041550089 2.0203791646
C 1.6158457574 2.8883531133 0.0704854134
C -4.173508307 2.9369116676 0.6695329771
H -4.8946482351 2.3947590328 1.2870385837

H -3.3254506798 3.1798251027 1.310726745
H -4.6416223745 3.8701338632 0.3381184077
C -3.7762965112 2.0719239597 -0.5425532095
H -3.2394726023 2.6914015274 -1.2768673259
P 2.8017751164 -1.0020950668 1.829054983
H 3.1653326545 -0.3315266307 3.0133890628
H 3.2303919841 -2.2950569395 2.1379396873
P 3.2949813144 2.1829112757 0.2810231482
H 4.0992892633 3.0768268976 -0.4299535659
H 3.5793314757 2.5931772802 1.5986087279
Pd 3.5201177809 -0.0844341568 -0.1835438363
Cl 4.0198656624 0.7361309402 -2.3061765916
Cl 3.6168697049 -2.3273618527 -0.8214821135

Energy= -3246.3647148
Zero-point correction= 0.418976 (Hartree/Particle)
Thermal correction to Gibbs Free Energy= 0.359016
Sum of electronic and zero-point Energies= -3245.945739
Sum of electronic and thermal Energies= -3245.916321
Sum of electronic and thermal Enthalpies= -3245.915377
Sum of electronic and thermal Free Energies= -3246.005699

st-I3-[ax]-i

C -3.8950004564 -1.4546764844 0.4038772049
C -2.7377826597 -1.0048328067 -0.149426337
C -2.3289179474 0.4229210945 -0.3754723651
C -0.9804000065 0.6904635096 -0.1054301984
O -0.1329850932 -0.3036482671 0.3352473446
C -0.3289334803 -1.5038255839 -0.2918905238
C -1.6298247497 -1.9201893776 -0.531744085
C -5.1223555988 -0.6157762791 0.3947933141
C -5.7670599683 -0.3053101343 1.5801395841
S -5.2979496191 -1.1994351852 3.0311907875
C -5.1899344791 -2.7761689949 2.1159327573
C -3.9887468121 -2.8045587708 1.1539433096
C -3.079872318 1.5303972481 -0.79386917
C -2.5057246105 2.7901073498 -0.9441541187
C -1.1510731783 3.0002482803 -0.7137838074
C 0.6143253229 -3.4073067248 -1.3798692875
C -0.6800871334 -3.8597721509 -1.636487763
C -1.7875134677 -3.1166523884 -1.2386042221
C -5.7154350279 -0.1879738556 -0.8425800006
C -6.828650911 0.7005527022 -0.804539368

C -7.3608627488 1.0991780741 0.4492376827
C -6.8687704426 0.5801021107 1.614248505
C -5.2405946725 -0.6104957551 -2.1149947802
C -5.814315149 -0.1522782866 -3.2727400922
C -6.8966928777 0.7551742032 -3.2302795717
C -7.3924549526 1.1659083063 -2.021166315
C -2.7358433327 -3.1536152877 1.9746517181
H -5.0977185888 -3.5737531985 2.8560149908
H -6.1403577162 -2.9019803227 1.5930819851
H -4.1858741842 -3.6149969246 0.438719701
H -4.125290932 1.4178668553 -1.0303241714
H -3.1274560389 3.6126191489 -1.2797611344
H -0.7012045134 3.9703288371 -0.895831735
H 1.4701501738 -3.9656565773 -1.7440729345
H -0.8230731359 -4.7851254487 -2.1832449229
H -2.7877645431 -3.459747621 -1.4834460147
H -8.2022842859 1.7858489165 0.467734933
H -7.3251599901 0.8205789776 2.5686590878
H -4.4021018387 -1.2951263645 -2.1651042145
H -5.4322119784 -0.4884018014 -4.2311881502
H -7.3354541041 1.1166983248 -4.1543950883
H -8.2335639453 1.8519427299 -1.9740176944
H -2.9248385689 -4.0576293828 2.5611779888
H -2.5031159169 -2.3387816472 2.6666049081
H -1.857270317 -3.3440900404 1.3632391019
C -0.3714418339 1.9282870805 -0.2879885354
C 0.8007989502 -2.2017896701 -0.7046596447
Pd 2.397037336 0.5973330442 -1.7262765405
Cl 2.2317934688 2.5134368639 -3.0538194041
Cl 3.1745734231 -0.8244696632 -3.415046461
P 2.3780484732 -1.332467376 -0.5202473741
H 2.5338088949 -1.2641567748 0.8753070464
H 3.3269495374 -2.3027556272 -0.8486370802
P 1.433666926 1.9500795055 -0.1666623275
H 1.7460569851 3.3093483377 -0.235926525
H 1.6727014039 1.6872342331 1.1930649378

Energy= -3246.3749563
Zero-point correction= 0.419752 (Hartree/Particle)
Thermal correction to Gibbs Free Energy= 0.357248
Sum of electronic and zero-point Energies= -3245.955204
Sum of electronic and thermal Energies= -3245.925035
Sum of electronic and thermal Enthalpies= -3245.924091
Sum of electronic and thermal Free Energies= -3246.017708

st-I3-[ax]-c

C -3.9311624733 -1.4989414401 0.283756279
C -2.8043988225 -1.0941340975 -0.3571346207
C -2.4191066642 0.306535963 -0.7309963091
C -1.0778566012 0.6226809846 -0.4528448635
O -0.2425004918 -0.315168086 0.1069077516
C -0.3902455169 -1.5520469361 -0.4532694742
C -1.6841037527 -2.0141634952 -0.6763076416
C -5.1586945308 -0.6590566983 0.263359599
C -5.7249507566 -0.2254674102 1.450061449
S -5.1715513915 -0.9816792618 2.948525902
C -5.1260272107 -2.6396500149 2.1831667855
C -3.9833702512 -2.7729608189 1.159591342
C -3.1826446781 1.3328911309 -1.2951407166
C -2.6320896094 2.5846420355 -1.5597747003
C -1.2956371418 2.8482365832 -1.2944441338
C 0.5684737858 -3.5190185321 -1.394550801
C -0.7119591751 -4.0127824381 -1.6297113296
C -1.831249767 -3.2566698823 -1.2924089856
C -5.8280168243 -0.3523050024 -0.9693982364
C -6.9305029628 0.5497069278 -0.952237497
C -7.3771948869 1.0812261718 0.2854421384
C -6.815545652 0.6742216774 1.4636678588
C -5.4439121245 -0.9163911823 -2.2174561734
C -6.0920592401 -0.5773376948 -3.3770200445
C -7.1616340504 0.3463803049 -3.3603430826
C -7.5708031001 0.8915811049 -2.1719963859
C -2.6862776518 -3.0619442029 1.9343953833
H -4.9955486303 -3.3638944249 2.989705365
H -6.106291638 -2.8037128031 1.7307066186
H -4.2283011688 -3.6425117152 0.5336316494
H -4.2147948677 1.1610846156 -1.5548936963
H -3.2573508487 3.3541039653 -1.9991337559
H -0.8725679484 3.8187137296 -1.5353148497
H 1.4328241603 -4.1050657053 -1.6923863939
H -0.8358113136 -4.9816547138 -2.1008555647
H -2.8286470752 -3.6285317515 -1.505961786
H -8.2106107637 1.7777840636 0.2875902908
H -7.2085215285 1.0151612187 2.4156915756
H -4.6158864911 -1.6152337549 -2.2462524456
H -5.7815406376 -1.0223970995 -4.3169307716
H -7.6605921055 0.6126347439 -4.2863138079
H -8.4020062935 1.5905803713 -2.1433169056
H -2.8578843021 -3.8823511104 2.6373311904

H -2.38481314 -2.1792161908 2.5060502383
H -1.8567369259 -3.3548455603 1.2958641361
C -0.4923657234 1.8488367493 -0.7393306216
C 0.7490921795 -2.2591930098 -0.812135395
Pd 3.0790208609 0.6745556634 -0.5837886707
Cl 4.1223316399 2.7504029175 -0.4009408197
Cl 5.1008249502 -0.4924881015 -0.5592898128
P 2.4059987179 -1.5321385459 -0.5884242663
H 2.8191844456 -2.1183376911 0.6136602807
H 3.115830562 -2.3081854232 -1.5059721486
P 1.2831551003 2.1183938737 -0.4352631967
H 1.4661413529 3.2673426277 -1.2057280055
H 1.2864505843 2.6721890713 0.8509673465

Energy= -3246.36774
Zero-point correction= 0.419432 (Hartree/Particle)
Thermal correction to Gibbs Free Energy= 0.356699
Sum of electronic and zero-point Energies= -3245.948308
Sum of electronic and thermal Energies= -3245.917850
Sum of electronic and thermal Enthalpies= -3245.916906
Sum of electronic and thermal Free Energies= -3246.011041

st-I3-[ax]-e

C -2.8106830105 1.0998654155 0.1290493324
C -1.5122454703 0.6989802324 0.1844781359
C -0.9288100755 -0.5675973212 -0.3809935352
C 0.3179786874 -0.3926095078 -1.0053122095
O 0.8740800229 0.8535940685 -1.0759976467
C 0.7848677636 1.5746911101 0.0705913743
C -0.4371826849 1.570486829 0.7344257052
C -3.9077980399 0.1097519076 -0.0281057203
C -4.8115798247 0.2235267915 -1.0718366552
S -4.8417667097 1.7356948488 -1.9832232379
C -4.624794457 2.7418812407 -0.4755601346
C -3.2139087606 2.5934621944 0.1188124009
C -1.4362363742 -1.8706190735 -0.4196744764
C -0.7333253308 -2.9075933639 -1.0330057458
C 0.5225460798 -2.7001172232 -1.5872088549
C 1.7986723642 2.9860330817 1.6980989731
C 0.5855316799 3.0204649129 2.3823995927
C -0.5128269131 2.2933909608 1.9254349913
C -4.1070292011 -0.9327015001 0.941807057
C -5.0943607986 -1.9295278787 0.69329148

C -5.8943357458 -1.8427513375 -0.475819197
C -5.7844557264 -0.7726852983 -1.3189213595
C -3.3623679511 -1.0186855832 2.150624717
C -3.5632728252 -2.0465950325 3.0355332769
C -4.5186748498 -3.052398295 2.7672301973
C -5.2683280189 -2.9881780669 1.6224163317
C -2.2611718994 3.4893418277 -0.691453709
H -4.8061609995 3.7817426043 -0.7547573864
H -5.4031615968 2.4335251986 0.2258485357
H -3.2723627259 2.970013921 1.149376531
H -2.3856270969 -2.1001527488 0.0366785205
H -1.1721376247 -3.8991505724 -1.0512169212
H 1.0730752547 -3.5281476666 -2.0231377303
H 2.6544999334 3.5281905229 2.0885652701
H 0.5040613079 3.5976537631 3.2968915683
H -1.4373998302 2.2906733658 2.4942741025
H -6.634876408 -2.6147774934 -0.6639265415
H -6.449274376 -0.6623049137 -2.1691112076
H -2.617943686 -0.2614746089 2.3667683969
H -2.9837359846 -2.0874600501 3.9523336787
H -4.6620925452 -3.8647139541 3.4721016523
H -6.0179832612 -3.745510429 1.4107159637
H -2.6724439679 4.501452942 -0.7473858748
H -2.1576761856 3.1053877389 -1.7102687893
H -1.267664377 3.5704721671 -0.2576070577
C 1.0781055266 -1.4193462951 -1.5579963696
C 1.9239357557 2.2263433757 0.5308325314
P 3.5081078935 1.88876512 -0.3085524813
H 4.228889536 3.0694228501 -0.1139837728
H 4.1260146701 1.0704391259 0.6567882146
P 2.8018168646 -1.070913818 -2.0458767133
H 3.4899582168 -1.7379588762 -1.0140134127
H 3.018847894 -1.9708722742 -3.0920058265
Pd 3.4348892158 1.1002027886 -2.4651908453
Cl 3.9869129094 3.3022521522 -2.9960177727
Cl 3.3255470671 0.4286606844 -4.6914906466

Energy= -3246.3593971
Zero-point correction= 0.418865 (Hartree/Particle)
Thermal correction to Gibbs Free Energy= 0.355295
Sum of electronic and zero-point Energies= -3245.940532
Sum of electronic and thermal Energies= -3245.910054
Sum of electronic and thermal Enthalpies= -3245.909110
Sum of electronic and thermal Free Energies= -3246.004102

TS-I3-i-to-c

C -2.7691771555 1.0913845206 0.1101206688
C -1.4781709663 0.6790515246 0.2024189636
C -0.888889447 -0.6011899267 -0.3119996499
C 0.3547503816 -0.4455998134 -0.943142222
O 0.9451063186 0.7932117073 -1.048376301
C 0.8306719023 1.5284378157 0.0976621758
C -0.3972154101 1.5377786949 0.750159842
C -3.8695728026 0.1085387156 -0.0752791106
C -4.7207916993 0.2113437765 -1.1625397344
S -4.6848980229 1.7109397388 -2.0972184321
C -4.5430187623 2.73549063 -0.5917911463
C -3.1666374824 2.5859422975 0.0808497936
C -1.3864649297 -1.9092842336 -0.2781829052
C -0.6784434035 -2.971617303 -0.834883368
C 0.5628781439 -2.7767783455 -1.4272019322
C 1.8324800789 2.91855262 1.7520041348
C 0.6152127828 2.9576168331 2.4284798695
C -0.4841509639 2.2544466982 1.9449452966
C -4.1280406189 -0.9086868586 0.905458164
C -5.1161751416 -1.8978870799 0.6320274673
C -5.8604591028 -1.8253935975 -0.5740924287
C -5.6968632543 -0.7749646152 -1.43369677
C -3.442444415 -0.9741918079 2.1500278025
C -3.6984922407 -1.9785542663 3.0474185708
C -4.6534246901 -2.97872295 2.7558913053
C -5.3473274364 -2.9327193423 1.5755922697
C -2.1727337866 3.487351301 -0.6710179263
H -4.7050447232 3.7729953357 -0.8910271787
H -5.3597646709 2.437894736 0.069193291
H -3.2820361842 2.9586928468 1.1082300273
H -2.3288582734 -2.1185246272 0.2017338255
H -1.100768446 -3.969164881 -0.7848690323
H 1.1176165599 -3.6181109576 -1.8303962531
H 2.692537219 3.4397542657 2.1610209373
H 0.5336814349 3.5177148266 3.3534538782
H -1.4195730034 2.2557186627 2.4958226191
H -6.6031217665 -2.5908964217 -0.7795812129
H -6.3215147629 -0.6731285077 -2.3148553487
H -2.6972826094 -0.221924248 2.3812387196
H -3.1628022346 -2.0063154439 3.9908743902
H -4.8400683573 -3.7729807841 3.4711137306
H -6.0955071995 -3.686266781 1.346054736
H -2.5906864745 4.4943425288 -0.7604184415

H -2.0006130906 3.097576609 -1.6786184983
H -1.2112505663 3.5815224551 -0.1725175681
C 1.0983134086 -1.4893003724 -1.4760041974
C 1.9611099666 2.1791238216 0.5717844375
Pd 4.4872526943 0.0396199245 -1.1741702519
Cl 5.7331082951 -1.6469276948 -2.1807919324
Cl 6.4222749822 1.1865698039 -0.5557873563
P 3.5622541709 1.954919396 -0.2650536101
H 3.5403675995 2.9614338319 -1.2424555944
H 4.3915533787 2.5527911005 0.6838357072
P 2.7484987933 -1.132679013 -2.1440263654
H 3.1228795818 -2.4162368239 -2.5436228453
H 2.4532630916 -0.5589569128 -3.3905551401

Energy= -3246.3668438
Zero-point correction= 0.419255 (Hartree/Particle)
Thermal correction to Gibbs Free Energy= 0.357995
Sum of electronic and zero-point Energies= -3245.947589
Sum of electronic and thermal Energies= -3245.917951
Sum of electronic and thermal Enthalpies= -3245.917007
Sum of electronic and thermal Free Energies= -3246.008849

TS-I3-c-to-e

C -2.8986149386 1.1289368215 -0.2862870977
C -1.6908278451 0.7199363202 0.1826892389
C -1.1254030214 -0.6746757892 0.142571649
C 0.2246567733 -0.7311940249 -0.2507829119
O 0.8997930596 0.427019328 -0.5254639025
C 0.6539868953 1.4187871898 0.3737980865
C -0.6717632588 1.6662818846 0.7107164992
C -4.022183687 0.1674129458 -0.4370370907
C -4.640417235 0.0017500234 -1.6652656839
S -4.3062729504 1.1926750136 -2.9262524976
C -4.3567399303 2.5798712339 -1.7396809935
C -3.1469923313 2.5702807987 -0.7887723274
C -1.7283420186 -1.908888347 0.3976662407
C -1.0222254389 -3.1046020609 0.2600360359
C 0.3131828463 -3.1129103487 -0.1147448174
C 1.4601675831 3.1145879269 1.8392602548
C 0.1406613184 3.4283910963 2.1581918621
C -0.9149256187 2.6841658186 1.6335711245
C -4.5457845873 -0.5430279547 0.6965552665
C -5.5404955806 -1.5413898857 0.4883115981

C -6.031665125 -1.773322505 -0.8227896313
C -5.6226498933 -0.9948031501 -1.8693747611
C -4.1203464883 -0.2912783691 2.0303785614
C -4.624907408 -1.0111106462 3.0825671696
C -5.583553284 -2.026744243 2.86665378
C -6.0315828684 -2.279276072 1.5968654532
C -1.9524696221 3.1881618782 -1.5351991232
H -4.3676702214 3.5022111951 -2.3238721432
H -5.3047271778 2.5020761812 -1.2030065805
H -3.4145019725 3.2164387613 0.0590692136
H -2.7558250213 -1.957165968 0.7209796912
H -1.5303433209 -4.0407639909 0.4639131501
H 0.8509275685 -4.0525737404 -0.1995697911
H 2.2741807929 3.6629409948 2.3033763399
H -0.0640651151 4.2334352853 2.8553846093
H -1.9346691584 2.8925021829 1.9423623891
H -6.7804314048 -2.5454864859 -0.9741376495
H -6.0568187079 -1.114742073 -2.856346949
H -3.3760368146 0.4760230214 2.2092148134
H -4.2859160718 -0.7984579455 4.0914515447
H -5.9684746349 -2.5948815206 3.7071053097
H -6.7807096264 -3.045696293 1.4186910051
H -2.2484265409 4.1509139625 -1.9619135926
H -1.6397070778 2.5329963519 -2.3533194858
H -1.0906125296 3.3709996776 -0.8984730636
C 0.9656550737 -1.9017280062 -0.3644149401
C 1.7357863481 2.0660527125 0.9561469815
Pd 3.960188857 0.1188556723 -1.1325381708
Cl 4.7835846905 -1.0324409495 -2.9810091989
Cl 5.2449352244 1.996541848 -1.6182838613
P 3.3886513448 1.3369850134 0.7289047751
H 4.2342034969 2.4140246699 1.0046514194
H 3.4980037877 0.6356549766 1.9446516433
P 2.7293800143 -1.825513825 -0.8373973576
H 3.2829591481 -2.7328079331 0.0805318138
H 2.7508095045 -2.6191936286 -1.9869609174

Energy= -3246.3585393
Zero-point correction= 0.418727 (Hartree/Particle)
Thermal correction to Gibbs Free Energy= 0.357095
Sum of electronic and zero-point Energies= -3245.939812
Sum of electronic and thermal Energies= -3245.910124
Sum of electronic and thermal Enthalpies= -3245.909180
Sum of electronic and thermal Free Energies= -3246.001445

TS5-i

C 2.728997681 1.2043117685 0.1035665194
C 1.4601655778 0.8084478512 0.3462829821
C 0.3247061368 1.6729527143 0.7697988569
C -0.8453452871 1.5500192787 0.0310091961
O -0.8629313468 0.6719726977 -1.02581932
C -0.2663008021 -0.5342290618 -0.7112886587
C 0.9257682149 -0.5559736075 0.0173487556
C 3.7686983185 0.1768340982 -0.1966441039
C 4.2016665712 0.0567289004 -1.5009739386
S 3.6332261237 1.2914973809 -2.6329715839
C 4.0028099326 2.6667773858 -1.4917313313
C 0.2651884302 2.4550030796 1.9246720213
C -0.9022936941 3.1343326606 2.2634890288
C -2.0517577842 3.0112173498 1.484431271
C -2.0344743218 2.1940460091 0.3537540614
C -0.9628257816 -1.674550238 -1.0951511084
C -0.4314285388 -2.9202441537 -0.7639268468
C 0.7591826975 -2.9794507374 -0.0474654967
C 1.4191941836 -1.8197834202 0.3536061067
C 4.3627622829 -0.6314056331 0.8196028202
C 5.2996043841 -1.6335937312 0.4383805474
C 5.6459066817 -1.7792908211 -0.9309268137
C 5.1313602576 -0.9387408167 -1.8803595113
C 4.0440041356 -0.4857147181 2.1972280095
C 4.6132987897 -1.3022710468 3.1401307821
C 5.532862828 -2.3067257767 2.758854762
C 5.8675220256 -2.4643126504 1.4392882995
H 5.0838859972 2.6688335403 -1.344304415
H 3.736517636 3.5763011883 -2.0342315763
H 1.1395561915 2.5193386144 2.5650801563
H -0.9291786959 3.740490961 3.1621304545
H -2.9708450005 3.5022438493 1.7861394138
H -0.966192497 -3.8284982161 -1.0205008615
H 1.1678784036 -3.9437222359 0.2334281203
H 2.3130140646 -1.9068900985 0.9548449241
H 6.3568097799 -2.5510838556 -1.2117239705
H 5.436825877 -1.0151965917 -2.9184542788
H 3.3285679492 0.2746854453 2.4921712355
H 4.3569565634 -1.179011751 4.1874691546
H 5.9729211182 -2.9487631448 3.5148405726
H 6.5772227427 -3.2300668886 1.1388116477
C 2.2627442166 3.7945659806 -0.0765408686
H 1.4365665204 3.6391137271 -0.7769964006

H 1.841886431 3.9671699142 0.9118724964
H 2.7873487036 4.7092865428 -0.3680637886
C 3.2524177943 2.6280534271 -0.1146301509
H 4.0198341492 2.80649932 0.6505952478
Cl -4.3588192791 -2.7390383343 0.3698600061
Cl -5.2301237454 0.4162167408 1.5765946046
Pd -3.9961727029 -0.4981664771 -0.1906217469
P -2.6236463044 -1.4126167437 -1.7596941722
H -2.3905006124 -0.7093581078 -2.9534231929
H -2.9831334219 -2.6692199452 -2.2510857282
P -3.4965728985 1.6854581118 -0.582645244
H -4.4605741581 2.6310901595 -0.2274282084
H -3.1867005192 2.0915981057 -1.8918727374

Energy= -3246.3730561
Zero-point correction= 0.419533 (Hartree/Particle)
Thermal correction to Gibbs Free Energy= 0.358545
Sum of electronic and zero-point Energies= -3245.953523
Sum of electronic and thermal Energies= -3245.924127
Sum of electronic and thermal Enthalpies= -3245.923183
Sum of electronic and thermal Free Energies= -3246.014511

TS5-c

C -2.870463921 1.1689614317 0.3125841259
C -1.7245310707 0.8745644811 -0.3392346144
C -0.6600150902 1.8261812373 -0.7529261183
C 0.6368428585 1.491026151 -0.3668550886
O 0.8080037664 0.355786008 0.3827897258
C 0.1072772433 -0.7055721308 -0.1479102585
C -1.2159616558 -0.5170716605 -0.5701882032
C -3.8708856294 0.0870011089 0.5481416731
C -3.9846239892 -0.4197180208 1.8265628582
S -3.0969410865 0.4375736269 3.0935626426
C -3.6479519315 2.079815547 2.5185186991
C -0.8209381253 2.9048891158 -1.6194709919
C 0.2805448872 3.6568298277 -2.0203863928
C 1.5604244804 3.3118175763 -1.5964458025
C 1.7609369438 2.1981172476 -0.7722838063
C 0.7967105413 -1.901784095 -0.2899019817
C 0.1170438921 -2.9846931096 -0.8572433841
C -1.1942913205 -2.832060073 -1.2883558145
C -1.8505465162 -1.6094492303 -1.1612873368
C -4.7377179925 -0.3939792405 -0.4792345097

C -5.6096873549 -1.4811776143 -0.1879012408
C -5.622774181 -2.0325940129 1.1204458905
C -4.84949562 -1.5001946829 2.1161159368
C -4.7571976277 0.1625531055 -1.7872484252
C -5.5846340328 -0.3458731941 -2.7552071757
C -6.4384546245 -1.4364448769 -2.469276187
C -6.4497865455 -1.9867129979 -1.2142015262
H -4.7332256431 2.1011365202 2.6291755454
H -3.2242890345 2.7963045609 3.2252394118
H -1.8144445749 3.1483374674 -1.9836754327
H 0.1446753137 4.5021561762 -2.6859808038
H 2.413125371 3.890738599 -1.9383611212
H 0.6229410464 -3.936729993 -0.9861986785
H -1.7110066305 -3.6674721227 -1.7478886237
H -2.8556542063 -1.507461648 -1.5458168399
H -6.2867059116 -2.8661594435 1.3307012699
H -4.9002672501 -1.885512771 3.1289156988
H -4.0950163308 0.9917380367 -2.0135464345
H -5.5869296329 0.0911077358 -3.7487021518
H -7.0859478435 -1.8314454999 -3.2452428057
H -7.1077157851 -2.8201710569 -0.9843855338
C -2.1859626285 3.578119325 1.1316580319
H -1.2413673348 3.2142771261 1.5473093837
H -1.9785063562 4.0361829559 0.1666469517
H -2.563845579 4.3666541887 1.7890656724
C -3.2279475746 2.4599675208 1.0568117815
H -4.1311362805 2.8649404166 0.5803870062
Cl 5.3993869671 -2.4432769183 0.5666155247
Cl 6.1796427031 0.831432796 0.0974668741
Pd 4.2509471143 -0.4841172996 0.0384142759
P 2.5451396381 -2.0261957037 0.198432026
H 2.4584279082 -2.4400260669 1.5331938698
H 2.8647035172 -3.2337277359 -0.4237040616
P 3.4315557779 1.6422076865 -0.3039281982
H 4.1765255972 2.3590886883 -1.241461801
H 3.6779113454 2.396398934 0.8493414685

Energy= -3246.3663342
Zero-point correction= 0.419677 (Hartree/Particle)
Thermal correction to Gibbs Free Energy= 0.358633
Sum of electronic and zero-point Energies= -3245.946657
Sum of electronic and thermal Energies= -3245.917123
Sum of electronic and thermal Enthalpies= -3245.916179
Sum of electronic and thermal Free Energies= -3246.007701

TS5-e

C -2.5004128543 1.1408902283 0.5533725872
C -1.488432434 0.9298316073 -0.3175411068
C -0.4401512636 1.9114157997 -0.7158401335
C 0.8734793475 1.4543969147 -0.6283809687
O 1.0625569338 0.1989812002 -0.1287742063
C 0.2409047206 -0.7284620293 -0.7148037371
C -1.1200063949 -0.4229055784 -0.8636417051
C -3.5368139504 0.0827647912 0.7332978908
C -3.5062283652 -0.6618163206 1.8946397377
S -2.3788608433 -0.1241344609 3.1449818435
C -2.8687797267 1.6263430039 2.9877632538
C -0.6470904343 3.1519188 -1.313269567
C 0.4374449919 3.9235396435 -1.7315629906
C 1.7373757698 3.4373806134 -1.6280449353
C 1.9729131809 2.1628561581 -1.0996677115
C 0.8329999704 -1.8958576048 -1.1837499161
C 0.0005135041 -2.8558436509 -1.7694860468
C -1.3553561074 -2.5960099528 -1.9154271636
C -1.9074782455 -1.3850655603 -1.4938410701
C -4.5725329051 -0.1440137657 -0.2232289737
C -5.4762827509 -1.2239587759 -0.0117319248
C -5.3467383852 -2.022603476 1.1552164626
C -4.4012747587 -1.736925639 2.1021660395
C -4.7294677328 0.6560941035 -1.3877648037
C -5.7199739793 0.3874750799 -2.2972590327
C -6.6083785213 -0.6935687073 -2.0928719333
C -6.4876595422 -1.4768592885 -0.9747326305
H -3.9187832066 1.6861089322 3.2783871419
H -2.2815713386 2.1609293032 3.7370236964
H -1.6613085106 3.5146756194 -1.4504709271
H 0.2646877069 4.9004972137 -2.1695372323
H 2.5672570018 4.0349940033 -1.9930892408
H 0.4177273195 -3.7897956525 -2.1340958488
H -1.9963095075 -3.3337837764 -2.3855723254
H -2.9562579692 -1.1953757848 -1.6743229037
H -6.0362815157 -2.8487670884 1.3032070726
H -4.3359772471 -2.3144781903 3.0180810758
H -4.0428000146 1.4796869634 -1.5533392718
H -5.8257287941 1.0091889042 -3.1806832516
H -7.3863452635 -0.8967418705 -2.8215434322
H -7.1699191047 -2.3055682564 -0.8068746539
C -1.5215194234 3.3057923061 1.6966598847
H -0.5539575643 2.8252650176 1.8705944916

H -1.4325309832 3.9471804364 0.8221033801
H -1.7338474828 3.95553608 2.5506139378
C -2.6441972236 2.2726702836 1.576451943
H -3.5757747487 2.8034957884 1.3374326993
Cl 4.2592041621 -2.4114468961 1.4476010169
Cl 5.1234639475 0.8725240636 1.4294825506
Pd 3.9000607673 -0.5714562962 0.0675171437
P 2.6502371967 -2.0458434125 -1.1749969057
H 2.8572703162 -3.3941950425 -0.8727269627
H 2.9045292879 -2.071577907 -2.5591034545
P 3.5842308033 1.3166625075 -1.196338577
H 3.7036011982 1.1794959287 -2.5926014162
H 4.5017189668 2.3480916892 -0.9814518885

Energy= -3246.3573397
Zero-point correction= 0.419016 (Hartree/Particle)
Thermal correction to Gibbs Free Energy= 0.356726
Sum of electronic and zero-point Energies= -3245.938324
Sum of electronic and thermal Energies= -3245.908753
Sum of electronic and thermal Enthalpies= -3245.907809
Sum of electronic and thermal Free Energies= -3246.000614

st-I4-[eq]-i

C 3.6763626513 -1.7849112926 0.0668201926
C 2.5845005855 -1.3818255266 -0.6135443144
C 1.3802621305 -2.2048418422 -0.9203682759
C 0.1654740312 -1.6529607967 -0.5253485192
O 0.1677431955 -0.4313276795 0.1050910371
C 1.0215360734 0.4711855343 -0.5000155131
C 2.28677689 0.0553292761 -0.9157960359
C 4.7782853698 -0.8415492668 0.3953913818
C 4.8806437488 -0.4294966761 1.7100932279
S 3.7053689439 -0.9647608503 2.9155888078
C 3.2683970294 -2.6484570102 2.3154861203
C 1.3227448942 -3.3713124868 -1.6857547884
C 0.1015981656 -3.9792261007 -1.9668971702
C -1.0961111293 -3.412266076 -1.5351889489
C -1.0715578364 -2.2191291062 -0.8130526191
C 0.5162478162 1.7478402114 -0.715409565
C 1.3314117223 2.682569749 -1.3534089507
C 2.6059934611 2.3055514248 -1.7647341555
C 3.0739160915 1.008043309 -1.5661849929
C 5.7988292029 -0.4882978355 -0.5379011633

C 6.8299754759 0.4026063294 -0.1296605364
C 6.8485921188 0.8716388035 1.2101549955
C 5.9172769117 0.4475591401 2.1167028482
C 5.8219341764 -0.9916197429 -1.8671533814
C 6.7997492487 -0.6056478101 -2.748130932
C 7.8098546457 0.2991615093 -2.3479394173
C 7.8235218239 0.7864734316 -1.066809164
H 3.6056359702 -3.3657916323 3.0672087696
H 2.1764506924 -2.6771710767 2.2649232494
H 2.2416006457 -3.7954603323 -2.0744167735
H 0.0794673661 -4.886529044 -2.5601761731
H -2.0440380887 -3.864325837 -1.8071010934
H 0.957508729 3.6787231516 -1.5647482568
H 3.2374962938 3.0236553617 -2.2758245949
H 4.0520149744 0.7341900295 -1.9389848941
H 7.6380433561 1.5514845454 1.5173344705
H 5.9654651361 0.7710942508 3.1513885579
H 5.0442361656 -1.6809995918 -2.1800722483
H 6.8008724836 -0.998396163 -3.7599438444
H 8.5752996737 0.599762148 -3.0558170145
H 8.6019151185 1.4739341584 -0.7474898622
C 3.2753688177 -4.3880326431 0.5141482506
H 2.1850289881 -4.4017263292 0.4898166817
H 3.6500488554 -4.6792741307 -0.4701659172
H 3.6054078154 -5.1472760428 1.2299410196
C 3.8420596134 -3.0324334173 0.9367386245
H 4.9220470439 -3.1699595697 1.0547160197
Cl -2.3712817934 2.5346398506 -3.1443130059
Cl -3.6539868401 -0.7082215237 -3.2290544576
Pd -2.5287705629 0.6863319927 -1.7219806619
P -1.2415358394 1.9836107387 -0.3647098554
H -1.3229480483 1.8006616027 1.0260672947
H -1.4153763387 3.365236353 -0.4670868152
P -2.5195871544 -1.1972822581 -0.4473457352
H -3.5915883269 -2.0775268342 -0.6076828616
H -2.4735564945 -1.0778057563 0.9521799894

Energy= -3246.3766327
Zero-point correction= 0.420022 (Hartree/Particle)
Thermal correction to Gibbs Free Energy= 0.357561
Sum of electronic and zero-point Energies= -3245.956610
Sum of electronic and thermal Energies= -3245.926359
Sum of electronic and thermal Enthalpies= -3245.925415
Sum of electronic and thermal Free Energies= -3246.019071

st-I4-[eq]-c

C 3.7946606815 -1.7626994244 0.0403871292
C 2.7118619275 -1.425323768 -0.6880448895
C 1.5194036646 -2.2752872685 -0.9529170825
C 0.2946905611 -1.7038367171 -0.6026837512
O 0.3034656926 -0.4570755787 -0.0312883501
C 1.1273158586 0.4090222987 -0.7175611102
C 2.4003920406 -0.0234632061 -1.1080028845
C 4.8871483685 -0.7888239103 0.3031942803
C 4.9758184255 -0.2716923588 1.5813154118
S 3.7993683016 -0.7150578726 2.8210552104
C 3.3525786292 -2.4349480678 2.3448821952
C 1.4933402648 -3.484485388 -1.6447696768
C 0.2831650023 -4.122355138 -1.9060053982
C -0.9196511793 -3.5375535422 -1.5221926122
C -0.9312890528 -2.2951677655 -0.8778148794
C 0.6129464595 1.6588963165 -1.0308782099
C 1.4335493144 2.5409590789 -1.7428697489
C 2.7054161503 2.1455083676 -2.1374648667
C 3.1822379288 0.8709912627 -1.8363492641
C 5.9115825781 -0.5018839043 -0.6486017692
C 6.9311131679 0.429569693 -0.3071712078
C 6.9346852091 1.005869386 0.9903001105
C 6.0009605652 0.646579859 1.9217414233
C 5.9520129792 -1.1127698669 -1.9316246435
C 6.9338413315 -0.7906940997 -2.8337116346
C 7.9312599205 0.1548335195 -2.5019090717
C 7.9287013987 0.7457108179 -1.2649819741
H 3.6679736625 -3.0931619367 3.1576864506
H 2.2615636114 -2.4571829355 2.2756513142
H 2.4253643748 -3.9202919505 -1.986933153
H 0.2758932524 -5.0688346941 -2.435293121
H -1.8568699842 -4.0327888692 -1.757824868
H 1.06726968 3.5281552351 -2.0080223921
H 3.3303714906 2.8284856336 -2.7022781476
H 4.1616900122 0.5713085506 -2.1854310015
H 7.7147808022 1.7167495985 1.2469179381
H 6.0379223636 1.053818734 2.9269263135
H 5.1850185848 -1.8353188015 -2.1918394528
H 6.9489769855 -1.267103071 -3.8089086364
H 8.7004121203 0.4036623256 -3.2256710899
H 8.6978939326 1.4646426883 -0.9967481878
C 3.3819377586 -4.3172763153 0.6940853362
H 2.2919337352 -4.3295994081 0.6552629158

H 3.7693463369 -4.6894977631 -0.257511158
H 3.6980732058 -5.0163188475 1.4744412329
C 3.94775589 -2.9331496732 1.0127023754
H 5.0256076609 -3.0612071953 1.1580495786
Cl -3.8421344552 2.9697175457 -0.2354792415
Cl -5.0814295511 -0.1937678365 -0.1688058743
Pd -2.9931863447 0.8102011745 -0.4597429799
P -1.0890600252 2.1055576743 -0.5672168844
H -0.9110797826 2.7207402954 0.677726643
H -1.2575242671 3.2242011606 -1.3842630135
P -2.4893790887 -1.4387328615 -0.4774875353
H -3.3603553601 -2.1759365199 -1.2811274289
H -2.7963027407 -1.9661928501 0.7823186621

Energy= -3246.3699323
Zero-point correction= 0.419895 (Hartree/Particle)
Thermal correction to Gibbs Free Energy= 0.357205
Sum of electronic and zero-point Energies= -3245.950038
Sum of electronic and thermal Energies= -3245.919549
Sum of electronic and thermal Enthalpies= -3245.918605
Sum of electronic and thermal Free Energies= -3246.012727

st-I4-[eq]-e

C 3.8810287612 -1.7952138276 0.0559825137
C 2.7748575698 -1.4609685898 -0.6399759053
C 1.5878029116 -2.3323814267 -0.8805554331
C 0.3672370375 -1.7766267473 -0.4953832355
O 0.3996443615 -0.5511488899 0.1046751165
C 1.1693637354 0.3542808731 -0.5793340906
C 2.4391174412 -0.046110695 -1.0150122196
C 4.9824288005 -0.8215205103 0.2754527916
C 5.1339738227 -0.3115581678 1.5507471518
S 4.0297229373 -0.7574046191 2.8511539126
C 3.5146561406 -2.4559631173 2.3715296765
C 1.5466328508 -3.5405115993 -1.5723123269
C 0.3301861219 -4.1870910442 -1.7935949452
C -0.8692947865 -3.6026348675 -1.39855437
C -0.8681764445 -2.3541550696 -0.7665751351
C 0.6139890737 1.600984567 -0.8425779993
C 1.4106232041 2.5335862211 -1.5170397353
C 2.6845439306 2.1783422305 -1.9408334228
C 3.1883786325 0.895461512 -1.7162879218
C 5.9547217377 -0.5240585047 -0.7273928217

C 6.9883824439 0.4080242115 -0.4332290377
C 7.0561593567 0.9771069283 0.865838122
C 6.1720172905 0.610437355 1.841108955
C 5.9286917654 -1.1221507868 -2.0168884174
C 6.8609836297 -0.7893790683 -2.9666935993
C 7.8730814809 0.1548312765 -2.6797255659
C 7.9337823193 0.7346841378 -1.4388835166
H 3.8315273995 -3.1274914808 3.1727434282
H 2.4220742531 -2.4462820025 2.3310246869
H 2.4685188652 -3.9762550797 -1.9411917102
H 0.3165939972 -5.1412378806 -2.3088867972
H -1.8095874653 -4.1006077523 -1.6152586769
H 1.0251513127 3.5264043936 -1.7291290899
H 3.2936122917 2.9005667604 -2.4733305953
H 4.1661993995 0.6343308711 -2.0993555052
H 7.8454928077 1.6899861569 1.0862127862
H 6.2573883728 1.0147406308 2.8445231712
H 5.15074659 -1.8437255094 -2.2444447755
H 6.8250143745 -1.25691297 -3.9457095691
H 8.6029589649 0.411760035 -3.44041898
H 8.7142863972 1.4533524784 -1.204613371
C 3.4799957007 -4.3469200497 0.7331600279
H 2.3892974521 -4.3537731516 0.737688183
H 3.8294623142 -4.727181309 -0.22992151
H 3.8218835147 -5.0428319811 1.5052553809
C 4.0647020208 -2.9646275625 1.0239617057
H 5.1463143895 -3.0968717049 1.1333243388
Cl -2.1092308597 2.1622877647 2.4812895813
Cl -3.2507361724 -1.0413712839 2.4348371002
Pd -2.2347217916 0.4277079874 0.9344725841
P -1.1413620782 1.8995148198 -0.4504085054
H -1.1538760368 3.231093578 -0.0275133424
H -1.6656394421 2.0564042408 -1.7471899023
P -2.3735255949 -1.3646898642 -0.4888046545
H -2.7395264187 -1.1014977183 -1.8229644611
H -3.3347665339 -2.3256463382 -0.1660214589

Energy= -3246.36141
Zero-point correction= 0.419250 (Hartree/Particle)
Thermal correction to Gibbs Free Energy= 0.355700
Sum of electronic and zero-point Energies= -3245.942160
Sum of electronic and thermal Energies= -3245.911648
Sum of electronic and thermal Enthalpies= -3245.910704
Sum of electronic and thermal Free Energies= -3246.005710

TS-I4-i-to-c

C -2.869203943 1.1516972472 0.1924000259
C -1.7467397266 0.8950767219 -0.5084422456
C -0.6787007035 1.8694341451 -0.8639996426
C 0.6109586475 1.5045221792 -0.4791583778
O 0.7807758678 0.3262072166 0.2037902484
C 0.0714260522 -0.7024947035 -0.3809958275
C -1.2468742099 -0.4868430512 -0.7923198738
C -3.819399155 0.0681789866 0.5585794932
C -3.8412309743 -0.3286587467 1.881732145
S -2.7391740393 0.3904164026 3.0596051575
C -2.5372885762 2.1023691845 2.4165456823
C -0.8106669439 3.0028795171 -1.6656734012
C 0.3043364957 3.7678143124 -1.9983250831
C 1.5759837861 3.3866445613 -1.5784378607
C 1.7478262763 2.2226581239 -0.8226652059
C 0.7539134316 -1.8940192051 -0.5735099121
C 0.0687106736 -2.9502107734 -1.1806895242
C -1.2466365987 -2.7725139313 -1.5946859022
C -1.8956710228 -1.5521631599 -1.4167178322
C -4.7916443825 -0.4470836145 -0.3507662745
C -5.6764369661 -1.4699657897 0.0902564481
C -5.6064879672 -1.9111328187 1.4378675652
C -4.7333480062 -1.3392391536 2.3205914999
C -4.9091908059 0.0230397698 -1.6871945463
C -5.8347249336 -0.5164369251 -2.5438159025
C -6.6960703497 -1.5507640153 -2.1111228048
C -6.6182781726 -2.0110764424 -0.8222697848
H -2.9482943049 2.7852076403 3.1636418412
H -1.4599267744 2.2718749869 2.3380896815
H -1.7911568501 3.2782258783 -2.0375706907
H 0.1854548168 4.6532904937 -2.6129101545
H 2.4397901336 3.9751830571 -1.8717199778
H 0.5693786586 -3.8983818135 -1.3500728764
H -1.7719164358 -3.588154621 -2.0791439542
H -2.9071888368 -1.4286798921 -1.7807526454
H -6.284000339 -2.692237554 1.770400951
H -4.7183607593 -1.6469301176 3.3611091351
H -4.2461200501 0.8132348453 -2.0247683492
H -5.9102533638 -0.1453480862 -3.5610367232
H -7.4214797887 -1.9715818847 -2.7996119296
H -7.2838495163 -2.7975588045 -0.477458992
C -2.8168692121 3.7908589625 0.58880016
H -1.7388796927 3.9495846754 0.5383942219

H -3.2485375714 4.0109935971 -0.3906673495
H -3.2297049043 4.5127194182 1.3001617232
C -3.1873848425 2.3799379962 1.0463189327
H -4.2733759041 2.3716326409 1.1868818111
Cl 5.3940529962 -2.5744227287 -0.7829998235
Cl 6.1818800842 0.6640485305 -1.0027116346
Pd 4.2268116494 -0.5640400165 -0.6730330674
P 2.5060137541 -1.9972543854 -0.1126582169
H 2.4492029751 -2.1280270044 1.2833960359
H 2.7682760034 -3.3181633027 -0.4778306124
P 3.391564922 1.5714886005 -0.3971280267
H 4.1830659505 2.5421496925 -1.0116176544
H 3.5304321788 1.9519856779 0.9464734013

Energy= -3246.3688399
Zero-point correction= 0.419528 (Hartree/Particle)
Thermal correction to Gibbs Free Energy= 0.358087
Sum of electronic and zero-point Energies= -3245.949311
Sum of electronic and thermal Energies= -3245.919578
Sum of electronic and thermal Enthalpies= -3245.918634
Sum of electronic and thermal Free Energies= -3246.010752

TS-I4-c-to-e

C -2.8792739632 1.1604988258 0.2722725808
C -1.7272115211 0.9216941912 -0.3863062485
C -0.6548566417 1.9118151085 -0.6909011707
C 0.6209578835 1.5327130078 -0.2724121053
O 0.7320573492 0.3791869156 0.4534634791
C 0.0869649036 -0.6713743359 -0.149381086
C -1.2057280925 -0.4620964072 -0.6490972967
C -3.8431480684 0.0688764531 0.5703526706
C -3.9378592689 -0.3494817315 1.8838275992
S -2.913604644 0.348607064 3.1393430552
C -2.6409571546 2.0604604523 2.5247980879
C -0.7524430425 3.0494606805 -1.4891815923
C 0.3839019949 3.7989562234 -1.7936554818
C 1.6454770447 3.373251505 -1.3889928244
C 1.7828029845 2.1946149018 -0.6477634453
C 0.7825244503 -1.8675226531 -0.2670020665
C 0.1294337014 -2.9331841022 -0.8991537946
C -1.1496429789 -2.7594431905 -1.4105025353
C -1.8108110555 -1.5343578767 -1.2986416768
C -4.7565157435 -0.4385681274 -0.4027573656

C -5.6585251853 -1.4736219305 -0.0303524422
C -5.6631091791 -1.9364787553 1.3118733937
C -4.8462944176 -1.3742064407 2.2523382828
C -4.7991183052 0.0504126769 -1.7369936418
C -5.6693408065 -0.4815491953 -2.6543876588
C -6.5477675056 -1.5270764951 -2.2885699379
C -6.5418480153 -2.0061877379 -1.0041195273
H -3.0650234067 2.7424749352 3.2652556693
H -1.5577120198 2.2049799111 2.491232679
H -1.7191298154 3.3448521382 -1.8814785974
H 0.2884393209 4.6986957143 -2.3915491288
H 2.5266375896 3.9336926582 -1.6863716714
H 0.6299596229 -3.8910552077 -1.0059813147
H -1.6433082643 -3.5839169341 -1.9131880636
H -2.797097388 -1.420861917 -1.7295592837
H -6.3526092559 -2.7275884552 1.5920517942
H -4.8880245548 -1.7003929569 3.2864776781
H -4.1234969517 0.8497415098 -2.0240577589
H -5.6879143089 -0.0949764422 -3.6685524591
H -7.2293498474 -1.9413498532 -3.0242379628
H -7.2211210988 -2.8015724845 -0.710109899
C -2.8347448553 3.7894222361 0.7250236646
H -1.754868273 3.9428567529 0.7288310755
H -3.2202776603 4.033335505 -0.2680559496
H -3.275822699 4.498284215 1.4323176837
C -3.2328018313 2.3716881227 1.1356390461
H -4.3239223944 2.3654635185 1.2290570649
Cl 4.2849763202 -1.7617440924 2.8775610114
Cl 4.9601224808 1.4632388428 2.2157913254
Pd 3.7080731218 -0.2289394447 1.2224685888
P 2.4740306103 -2.0080785138 0.4036100775
H 2.3267563834 -3.0129642267 1.3631386121
H 3.0998717657 -2.7449556181 -0.6145206545
P 3.3773639331 1.349740886 -0.407997676
H 3.5918463743 0.8914419282 -1.7216216266
H 4.2835378295 2.4125142661 -0.3822608959

Energy= -3246.3607867
Zero-point correction= 0.419066 (Hartree/Particle)
Thermal correction to Gibbs Free Energy= 0.357429
Sum of electronic and zero-point Energies= -3245.941721
Sum of electronic and thermal Energies= -3245.911970
Sum of electronic and thermal Enthalpies= -3245.911026
Sum of electronic and thermal Free Energies= -3246.003358

TS6-i

C -3.7842904075 -1.266638201 -1.0835200272
C -2.561359518 -0.9668668987 -1.6146545984
C -1.8796985587 0.3208657144 -1.9363321699
C -0.7679278962 0.5972218503 -1.1493354961
O -0.3781015345 -0.3608968984 -0.2313117254
C -0.3542063117 -1.6042348756 -0.843030259
C -1.4359590517 -1.9766657442 -1.6451918414
C -4.8239861261 -0.4139666738 -0.3907532238
C -5.9436113455 -1.0346956092 0.1805130423
S -6.6876671896 -2.5512922165 -0.3013104989
C -5.4190770655 -3.1610105325 -1.4128124231
C -4.0333068235 -2.7731294377 -0.912861425
C -2.0875700096 1.1358247884 -3.0510961884
C -1.2929316109 2.259567857 -3.2615312207
C -0.2402561915 2.5625534898 -2.3987856892
C 0.9185194162 -3.5347771608 -1.4552681391
C -0.1211214419 -3.910321134 -2.3007246978
C -1.2720705831 -3.1324175385 -2.4124228511
C -4.6389615295 0.9783125018 0.0175184267
C -5.239192509 1.47026721 1.2123371757
C -6.2201725213 0.6833191499 1.8699840306
C -6.6273718543 -0.4794811182 1.3031239332
C -3.9755823094 1.9439336971 -0.7675970381
C -3.7315455183 3.2242072154 -0.3273182043
C -4.1723255425 3.6358653076 0.9421216431
C -4.9484811556 2.7748158966 1.6774237565
C -3.7006969101 -3.2687586915 0.500339048
H -5.5233139137 -4.2489319631 -1.4360033154
H -5.5824488242 -2.7753965985 -2.4230480087
H -3.3634446668 -3.3001053894 -1.5826229605
H -2.8941849664 0.8989473939 -3.7384930888
H -1.4780018318 2.8946570792 -4.1207020182
H 0.3991368396 3.4165069068 -2.5959837882
H 1.830571727 -4.1204711153 -1.4134790964
H -0.0217002626 -4.80525619 -2.9048438815
H -2.0420389836 -3.421638385 -3.120483528
H -6.6859416659 1.0538410125 2.7781871006
H -7.443423372 -1.0471976027 1.7393926699
H -3.7223166662 1.6947463153 -1.7776728915
H -3.22221608 3.9216223411 -0.9853784584
H -3.9620130297 4.6374136761 1.3023957819
H -5.3908222685 3.0953454294 2.6166040323
H -2.6303345711 -3.1405831748 0.6854602393

H -3.9386364658 -4.3327731972 0.5959423178
H -4.2454407223 -2.7275503943 1.2759188215
C 0.0486125883 1.7056073494 -1.3352465452
C 0.8126360149 -2.3499985101 -0.7252665813
P 1.57867929 1.758368965 -0.3760443646
P 2.197702285 -1.5625916698 0.1296444244
Pd 3.0092910776 0.1562270416 -1.1150628894
Cl 3.689648933 1.8546821297 -2.5742714343
Cl 4.3200855421 -1.5385385791 -2.0573276962
H 3.0730580708 -2.6189320442 0.389109726
H 1.6822748442 -1.3101567873 1.4113890287
H 1.1224769246 1.7241470005 0.9517388087
H 2.002282483 3.0839653425 -0.4885788338

Energy= -3246.3374582
Zero-point correction= 0.419595 (Hartree/Particle)
Thermal correction to Gibbs Free Energy= 0.359498
Sum of electronic and zero-point Energies= -3245.917863
Sum of electronic and thermal Energies= -3245.888596
Sum of electronic and thermal Enthalpies= -3245.887652
Sum of electronic and thermal Free Energies= -3245.977960

TS6-c

C -3.8555657708 -1.3660246149 -1.05635492
C -2.6669890958 -1.0496830831 -1.6498964868
C -2.0228971276 0.2389046326 -2.0340216855
C -0.8889151735 0.5556699451 -1.2880256437
O -0.4801487766 -0.3679502908 -0.3472179112
C -0.4146746208 -1.6074987002 -0.9561114222
C -1.516864596 -2.0263281752 -1.7129031949
C -4.8894173121 -0.5197659165 -0.349417668
C -5.968282843 -1.1506593058 0.285308619
S -6.6838795865 -2.7045683679 -0.1135499247
C -5.4418071819 -3.319387119 -1.2520581966
C -4.0497129065 -2.8723295929 -0.8244847406
C -2.2850482286 1.0013255829 -3.1713807285
C -1.515053203 2.1263479936 -3.4522982203
C -0.4443665305 2.4723117019 -2.631440639
C 0.8787437158 -3.4978575 -1.6240019311
C -0.178806829 -3.9161807356 -2.4234036643
C -1.358980587 -3.1775092479 -2.4832703035
C -4.7249631771 0.8892229661 0.0054373391
C -5.2936648329 1.4058700321 1.2049824256

C -6.2296606268 0.6177149703 1.9238147399
C -6.6260214515 -0.574421989 1.412681599
C -4.1117137327 1.8428927481 -0.8332439424
C -3.8823168652 3.1418113301 -0.4421035124
C -4.2874618113 3.5844522678 0.8287899087
C -5.0173396413 2.7308451861 1.6180282384
C -3.6448608854 -3.3041149229 0.5908879534
H -5.5134856618 -4.4099785478 -1.2298900654
H -5.6569687762 -2.9783476244 -2.268687836
H -3.3900992334 -3.4032504378 -1.5019254593
H -3.1130684848 0.7255683453 -3.8176099508
H -1.740902195 2.7312771946 -4.3236288532
H 0.1522443252 3.3482048142 -2.8676296311
H 1.7975896126 -4.0756182555 -1.5999733843
H -0.0780687439 -4.8158451129 -3.0205741614
H -2.1566955842 -3.4948681435 -3.1474265503
H -6.6711067841 1.0072157884 2.8361553289
H -7.4095131514 -1.1474288794 1.8988138483
H -3.8878025907 1.5666565844 -1.843272791
H -3.4125870833 3.8293970564 -1.1388955252
H -4.0860043742 4.6007563653 1.1504178703
H -5.4317549754 3.0711579029 2.5628716237
H -3.8411186108 -4.3713180006 0.7336615681
H -4.1776399751 -2.7532685834 1.3679164922
H -2.5735298007 -3.1323272008 0.7288481394
C -0.0923522089 1.6626780507 -1.545013236
C 0.7817178625 -2.3085231292 -0.8921579408
P 1.3929889467 1.988739504 -0.5487304827
P 2.1910982459 -1.6554452955 0.057057736
Pd 2.9542407443 0.5043612252 0.2479323138
Cl 3.9311395561 2.570361241 0.7228009927
Cl 4.6544057927 -0.7247533951 1.2718426851
H 3.1813744817 -2.5386957107 -0.3752309505
H 1.9399007921 -2.1532037446 1.3408518012
H 0.8744853245 2.7179132452 0.5273282743
H 1.9605142971 3.0113112591 -1.3102521433

Energy= -3246.3317514
Zero-point correction= 0.419684 (Hartree/Particle)
Thermal correction to Gibbs Free Energy= 0.359497
Sum of electronic and zero-point Energies= -3245.912067
Sum of electronic and thermal Energies= -3245.882626
Sum of electronic and thermal Enthalpies= -3245.881681
Sum of electronic and thermal Free Energies= -3245.972254

TS6-e

C -2.7523204085 0.8984173874 0.3928318577
C -1.6081192242 1.0228223689 1.130113244
C -0.8133815478 0.054412558 1.9441814667
C 0.442152424 -0.2389558777 1.409090509
O 0.7729895989 0.4187017089 0.2496493535
C 0.586913572 1.7701967224 0.4288168462
C -0.6555843076 2.1833468768 0.933310313
C -3.5226997148 -0.3117481603 -0.080441379
C -4.6115493326 -0.1299163443 -0.9442838352
S -5.6456927884 1.2841150048 -1.0745117817
C -4.6729404345 2.4500860834 -0.1193827377
C -3.1828033143 2.195206868 -0.3093317914
C -1.0811073069 -0.3868292629 3.2368237048
C -0.1739369026 -1.2169365401 3.89598906
C 1.0400975682 -1.5520668322 3.3050485709
C 1.4707875907 3.9859236891 0.5067817149
C 0.2610150641 4.4255792916 1.0277458607
C -0.7808373255 3.5284184135 1.2718909257
C -3.0551602725 -1.6943149824 0.0126238227
C -3.352066201 -2.628665855 -1.0202940243
C -4.3203849041 -2.2915987922 -2.0014590681
C -5.0009506648 -1.1232568425 -1.8912009537
C -2.3943852151 -2.226497025 1.1386824902
C -1.8728454497 -3.499498493 1.1628325347
C -2.008221002 -4.3456476587 0.0491867285
C -2.7751994121 -3.9204965609 -1.0068020543
C -2.6986876813 2.2671502923 -1.7632953872
H -4.9356742942 3.4460288282 -0.4855881942
H -4.9386790579 2.388801406 0.9398475711
H -2.715019577 3.0260922238 0.2065985806
H -2.0173539851 -0.1105483206 3.7126843168
H -0.4088044749 -1.5901304085 4.8868873086
H 1.7429762318 -2.1835622167 3.8405253074
H 2.2813892246 4.6917755006 0.3521092946
H 0.1318053962 5.4738560897 1.2741027514
H -1.6921480465 3.8868613549 1.7398834256
H -4.5557575426 -3.0011721601 -2.7888399213
H -5.8093004023 -0.890870369 -2.5774292796
H -2.368687863 -1.6454028536 2.0379079581
H -1.3800365609 -3.8531627978 2.0634200398
H -1.5702079218 -5.3378562611 0.051997458
H -2.9834735755 -4.5849126903 -1.840513977
H -3.0311847207 1.4174627814 -2.361616464

H -1.6053384464 2.2836347418 -1.7825408585
H -3.0629090082 3.1829741214 -2.23917992
C 1.3892792772 -1.0227903373 2.0553108165
C 1.6649683088 2.6254267383 0.2357479492
P 3.3078726299 1.939378739 -0.1552256997
H 3.8067851685 2.8274795371 -1.1118668361
H 4.0275589092 2.3758625633 0.9723696187
P 3.0666300988 -1.1772890676 1.3622334451
H 3.8286699484 -0.6903493941 2.4396409017
H 3.3388289238 -2.5433459828 1.4758856203
Pd 3.5034262016 -0.2787152651 -0.7017088271
Cl 3.916495955 0.5374336225 -2.8437313864
Cl 3.7077288259 -2.5008392712 -1.3668118103

Energy= -3246.3218406
Zero-point correction= 0.419011 (Hartree/Particle)
Thermal correction to Gibbs Free Energy= 0.356564
Sum of electronic and zero-point Energies= -3245.902830
Sum of electronic and thermal Energies= -3245.873280
Sum of electronic and thermal Enthalpies= -3245.872336
Sum of electronic and thermal Free Energies= -3245.965277

a-stable-[ax]-i

C -3.941455431 -1.6534344915 0.0365213693
C -2.709458429 -1.3869524694 -0.4438825491
C -2.2986232084 -0.0498675305 -0.9546156376
C -1.0344298263 0.384282909 -0.5784149327
O -0.2706640104 -0.4122376125 0.2483394095
C -0.3238052114 -1.7455831963 -0.0895340424
C -1.5451527144 -2.3181018146 -0.445023716
C -5.0173528212 -0.6344134641 0.0918403513
C -6.2210737207 -0.931248257 -0.5204707896
S -6.5441781057 -2.4655706244 -1.3313850226
C -5.2764587673 -3.6077632975 -0.6522958832
C -4.4132303568 -3.0246865342 0.4771403778
C -3.0220223365 0.7746676621 -1.8198272828
C -2.4848199652 1.9809317419 -2.2585969968
C -1.2032301266 2.3776632568 -1.8785441186
C 0.8893679256 -3.7622662533 -0.5081434975
C -0.3116903697 -4.369553774 -0.8654822327
C -1.5072132949 -3.6540336388 -0.8566206172
C -4.8836948268 0.5916939899 0.8247681739
C -5.9347632254 1.5472022379 0.7721933523

C -7.1199376133 1.2337711293 0.0549040602
C -7.2763830989 0.0181228689 -0.545770301
C -3.743154087 0.8931280232 1.6158038689
C -3.6375904136 2.0911914227 2.2746795639
C -4.6658686147 3.0557021229 2.1856723942
C -5.7929697409 2.7813939883 1.4560045606
C -5.1436540111 -2.9936036369 1.824932476
H -5.8140176617 -4.4912944659 -0.2982089393
H -4.6437583381 -3.8989175898 -1.4934405606
H -3.5518264839 -3.6887073554 0.5871318517
H -4.0085245524 0.4648924177 -2.1472316438
H -3.0600054971 2.6087084788 -2.9297491399
H -0.7750737434 3.29301947 -2.2731070899
H 1.8225086962 -4.3111250021 -0.5764516497
H -0.3111877528 -5.4030448902 -1.1934278696
H -2.4126449034 -4.133488615 -1.2111217559
H -7.9211705699 1.9662536164 0.0183977557
H -8.2042883018 -0.2320472621 -1.0505422101
H -2.951036657 0.1583351508 1.7044946612
H -2.7579922955 2.2977091663 2.8764073423
H -4.5666418196 4.0023749175 2.706655256
H -6.6019334949 3.504277425 1.3971053024
H -5.3795423764 -4.0131535566 2.1438345709
H -6.0770236211 -2.4283288468 1.7737499839
H -4.5117229955 -2.5342334314 2.5900736511
C -0.4546381426 1.5648397714 -1.0281617865
C 0.8902470725 -2.4216223966 -0.12436697
Pd 2.5627252924 0.1365011962 -1.5756941454
Cl 2.6004697529 1.6056091792 -3.3924375917
Cl 3.6701114048 -1.6260427479 -2.6444268253
P 1.2985764452 1.7867468406 -0.6446158122
H 1.5631910956 3.095087008 -1.0541598361
H 1.3032636534 1.9345271556 0.7533679111
P 2.3700918503 -1.400777206 0.0930500148
H 2.2523701115 -0.9567401502 1.4214026023
H 3.3981591343 -2.3379141154 0.2137277647

Energy= -3246.391912
Zero-point correction= 0.419713 (Hartree/Particle)
Thermal correction to Gibbs Free Energy= 0.357299
Sum of electronic and zero-point Energies= -3245.972199
Sum of electronic and thermal Energies= -3245.941853
Sum of electronic and thermal Enthalpies= -3245.940909
Sum of electronic and thermal Free Energies= -3246.034613

a-stable-[ax]-c

C -4.0585272949 -1.3433399076 -0.497259222
C -2.8237462585 -1.0738824926 -0.9674811026
C -2.2773148145 0.3017009769 -1.1071499936
C -0.9621355363 0.4771871761 -0.6817466527
O -0.2978350697 -0.5983416921 -0.1401342362
C -0.4844412014 -1.7480925862 -0.8664628067
C -1.7723201557 -2.0694843901 -1.3106913618
C -5.0053483704 -0.2807076462 -0.0815808704
C -6.2578928708 -0.2673196262 -0.6667333778
S -6.7809165015 -1.4614843542 -1.8578832938
C -5.6414939336 -2.8796490037 -1.6019666557
C -4.666377583 -2.7296885749 -0.4237455612
C -2.9300890265 1.3974901696 -1.669180992
C -2.2772708972 2.6219985642 -1.7758430766
C -0.9580790764 2.7614246073 -1.3544306551
C 0.4636659786 -3.6609460711 -1.9306940854
C -0.8013662565 -4.0119568195 -2.388066658
C -1.9057769947 -3.2129324324 -2.0989841196
C -4.7014294795 0.6630027656 0.9553322953
C -5.6361533425 1.6933007563 1.2474690815
C -6.8776264526 1.7176285161 0.5579094422
C -7.197504529 0.7478400405 -0.3481517095
C -3.5030992023 0.6092313074 1.7159899554
C -3.2322288809 1.5524779597 2.6738973785
C -4.1443626413 2.6001268824 2.9314662957
C -5.3245459782 2.659774575 2.2375029114
C -5.3281980302 -3.0116895824 0.9304279072
H -6.2704579064 -3.7622803907 -1.459706427
H -5.0853559416 -2.9959668153 -2.5346802376
H -3.8836228335 -3.4792250149 -0.568128311
H -3.951551392 1.2871218827 -2.017089459
H -2.7949644078 3.471066305 -2.2080904948
H -0.4542168159 3.7162795976 -1.4711053665
H 1.3203929544 -4.275054546 -2.1912042253
H -0.9255254405 -4.8990922375 -2.9992683366
H -2.8724658033 -3.4668426268 -2.5195023695
H -7.5902066214 2.5048320416 0.7869091425
H -8.1678966407 0.7459842054 -0.8346453835
H -2.7991651704 -0.1963280397 1.5408179953
H -2.3114457816 1.4888619595 3.2452299248
H -3.9146727615 3.3434147211 3.687859905
H -6.0460345974 3.4460115016 2.4418735322
H -5.6647274158 -4.0518335782 0.9705793891

H -6.192219245 -2.367092572 1.1073852914
H -4.6149450306 -2.8521496266 1.7437374938
C -0.2697860324 1.6730910814 -0.8071512374
C 0.6419947072 -2.499307966 -1.1710975063
Pd 3.1427295221 0.1871171686 -0.3487844848
Cl 4.3164391949 2.1413372624 0.1389289557
Cl 5.0371382382 -1.161108708 -0.1529663057
P 1.4798607741 1.787660235 -0.3198085713
H 1.8382778748 2.9376784357 -1.0245437922
H 1.3936541004 2.3012012005 0.9796240146
P 2.2969183759 -1.9408682141 -0.6457824733
H 2.4763064664 -2.6505844104 0.5473948404
H 3.0744740689 -2.7067091397 -1.5156227894

Energy= -3246.3856692
Zero-point correction= 0.419594 (Hartree/Particle)
Thermal correction to Gibbs Free Energy= 0.356890
Sum of electronic and zero-point Energies= -3245.966075
Sum of electronic and thermal Energies= -3245.935469
Sum of electronic and thermal Enthalpies= -3245.934525
Sum of electronic and thermal Free Energies= -3246.028779

a-stable-[ax]-e

C -4.1405364927 -1.3467240234 -0.5705644173
C -2.8990706522 -1.072424975 -1.0199601904
C -2.3631602525 0.3152340758 -1.1257484467
C -1.0754552708 0.492100781 -0.6282583708
O -0.4636397013 -0.5900853801 -0.0577878236
C -0.5732280827 -1.7371459852 -0.7901388123
C -1.8290493754 -2.072853647 -1.3076125319
C -5.0849201964 -0.2855078426 -0.1491183805
C -6.3342590844 -0.2583759232 -0.7395283629
S -6.8563120767 -1.4390285591 -1.9460780583
C -5.7137733937 -2.8589872852 -1.7087208289
C -4.7508910235 -2.7317443071 -0.5177347529
C -2.990279227 1.4251649361 -1.6872596621
C -2.3379242262 2.6571825324 -1.7151752575
C -1.0372174139 2.7917711137 -1.236332342
C 0.4687886801 -3.6520541423 -1.7530730563
C -0.767108285 -4.0301021059 -2.266940487
C -1.8987502899 -3.2344821836 -2.0757053613
C -4.7755216623 0.6445653348 0.8991086537
C -5.7015835758 1.6814730313 1.1942627299

C -6.9417051938 1.7187060715 0.5026155196
C -7.2680811012 0.7592035091 -0.4129189919
C -3.5793441095 0.5749322504 1.6623230473
C -3.2944141148 1.5159095327 2.6184233514
C -4.1974096942 2.5718710728 2.8764386137
C -5.3798239874 2.642416901 2.1868504971
C -5.424865993 -3.0291769448 0.8268372444
H -6.3419407547 -3.7457399171 -1.5907455419
H -5.1454212657 -2.9551380625 -2.6365889235
H -3.9696550411 -3.4820304774 -0.6658180288
H -3.9951781757 1.3279403955 -2.0843252286
H -2.8445725993 3.5199744265 -2.1331727374
H -0.5363531816 3.7532601396 -1.2966853766
H 1.3445732676 -4.2644742543 -1.9446191083
H -0.8477181579 -4.9404134545 -2.8508201659
H -2.8334339969 -3.5154683784 -2.5492044469
H -7.649075712 2.5093073175 0.7365553673
H -8.2376745149 0.7690425364 -0.9009502763
H -2.8829770645 -0.2379637159 1.4914087925
H -2.3697419647 1.4439024512 3.1824366879
H -3.9579967677 3.3130127316 3.6319112559
H -6.0943829298 3.4341574668 2.3948836171
H -5.7652338566 -4.0685856061 0.8497472675
H -6.2877645589 -2.3835251118 1.0050857558
H -4.7174282223 -2.8835869911 1.6476536381
C -0.3685823287 1.6869277128 -0.6988088878
C 0.5880037035 -2.4652637932 -1.0217864012
Pd 2.0952813799 -0.0733732228 1.0648447364
Cl 1.8660932871 1.4938186602 2.7776951464
Cl 2.6715342964 -1.8177753177 2.5013607426
P 1.3961894845 1.6753636596 -0.249633085
H 1.98718338 1.820055215 -1.51951954
H 1.6159473058 2.9556825742 0.2638387201
P 2.1832517399 -1.7286549069 -0.5249255878
H 2.9711376429 -2.8404834595 -0.2174856882
H 2.7088262215 -1.4052494354 -1.7907814458

Energy= -3246.37875
Zero-point correction= 0.419007 (Hartree/Particle)
Thermal correction to Gibbs Free Energy= 0.355326
Sum of electronic and zero-point Energies= -3245.959743
Sum of electronic and thermal Energies= -3245.929115
Sum of electronic and thermal Enthalpies= -3245.928171
Sum of electronic and thermal Free Energies= -3246.023424

TS-stable-i-to-c

C 3.0296890857 1.0539368533 0.2133350942
C 1.7847193228 1.0215210829 -0.3040370006
C 1.1939556868 -0.177375889 -0.9565147493
C -0.1260566416 -0.4626364529 -0.6215622385
O -0.767456862 0.3367276953 0.2974552593
C -0.5321687991 1.6725310866 0.0780850027
C 0.7642509909 2.1021109357 -0.2182138633
C 3.9452390258 -0.1115120508 0.1687187081
C 5.1941673354 0.070426404 -0.3959973038
S 5.7499160122 1.6186949396 -1.0374756555
C 4.6527807364 2.8558223978 -0.2374731927
C 3.6805785288 2.2868416574 0.8076556129
C 1.8081090356 -0.9950232246 -1.9057458242
C 1.1127556228 -2.0605267166 -2.4690895565
C -0.2135739201 -2.3087793778 -2.1229988218
C -1.4299876505 3.8718904441 -0.1411653572
C -0.153039691 4.3375303794 -0.4387136259
C 0.9280209865 3.4606293762 -0.4978997507
C 3.6174534439 -1.375640825 0.7626560828
C 4.5221150862 -2.4624463598 0.6178460723
C 5.758817838 -2.2499476799 -0.047545586
C 6.1035687254 -1.0133713343 -0.5118830495
C 2.4245854913 -1.5904948301 1.5032613087
C 2.1304948694 -2.8238828161 2.025586602
C 3.0123599713 -3.9124032844 1.8433615129
C 4.1866413798 -3.7286813026 1.1612804309
C 4.3582187626 2.0047025385 2.1539427044
H 5.3076842694 3.5997350893 0.223576746
H 4.0948873464 3.3361018255 -1.0442163499
H 2.9205947138 3.054052885 0.9783441317
H 2.8336369038 -0.7941892138 -2.1963136433
H 1.6024475198 -2.6935937228 -3.2005839142
H -0.7516543995 -3.1250315636 -2.5949396048
H -2.2693094505 4.5601842399 -0.131996267
H -0.0024214918 5.3891904994 -0.6559449726
H 1.9006216153 3.8348130147 -0.7972856538
H 6.4481762111 -3.0826727685 -0.153409288
H 7.071075756 -0.848374291 -0.9756003265
H 1.7430948738 -0.7625865719 1.6625777027
H 1.2144801328 -2.9634141968 2.5910506756
H 2.7644313918 -4.884573598 2.2565967902
H 4.8852714248 -4.5508907699 1.0334799386
H 4.7268262023 2.938853987 2.5877503983

H 5.2028812252 1.3193823194 2.0532236497
H 3.6447910488 1.5614744352 2.8541521153
C -0.8604145009 -1.4917793675 -1.1914382638
C -1.6363174581 2.5114714727 0.1047350913
Pd -4.2528059221 -0.0284981836 -0.7022615748
Cl -5.5354129319 -1.7934635113 -1.5145303341
Cl -6.1376240403 1.3270034975 -0.5103336896
P -2.6208551164 -1.6649443693 -0.7829998721
H -2.9559338081 -2.7172274088 -1.6354761076
H -2.5865524802 -2.3422146048 0.446046864
P -3.2891800315 1.7928143671 0.3476284815
H -3.330482028 1.6011150614 1.7375163882
H -4.0604136792 2.9501915703 0.2357546729

Energy= -3246.3843524
Zero-point correction= 0.419658 (Hartree/Particle)
Thermal correction to Gibbs Free Energy= 0.358423
Sum of electronic and zero-point Energies= -3245.964694
Sum of electronic and thermal Energies= -3245.934926
Sum of electronic and thermal Enthalpies= -3245.933982
Sum of electronic and thermal Free Energies= -3246.025930

TS-stable-c-to-e

C -2.8579011843 0.9981653105 -0.2984351249
C -1.6751754711 1.0190469261 0.3487486476
C -1.1413498206 -0.1343199843 1.1252324598
C 0.2167146998 -0.3880492888 0.9546702282
O 0.8990580624 0.3491768752 0.0216272761
C 0.6577729707 1.6906428076 0.1259582309
C -0.6653942656 2.1171385326 0.2982327785
C -3.7477776431 -0.1871387091 -0.2939255645
C -5.0542915466 -0.0113769247 0.1225811317
S -5.7050861092 1.5518026027 0.6263003901
C -4.5551846505 2.7768939632 -0.1173303382
C -3.4642090559 2.1831527435 -1.0221705582
C -1.8286182375 -0.9151345158 2.0530473961
C -1.1594059012 -1.910362255 2.7630197393
C 0.213683458 -2.0913737453 2.6183436607
C 1.507690141 3.9138232446 0.2420657634
C 0.2101067498 4.374269072 0.4315338984
C -0.865364831 3.4850617832 0.4763469147
C -3.3281375163 -1.4684641491 -0.7846437145
C -4.2202530488 -2.5706548193 -0.6851216158

C -5.5276082257 -2.3588733335 -0.1719987412
C -5.9483290783 -1.1115338945 0.1908362343
C -2.0551362534 -1.6866684199 -1.3758165595
C -1.6761164757 -2.9359803909 -1.7955035431
C -2.550013449 -4.0373268997 -1.6560964083
C -3.7982704861 -3.8525308896 -1.1214249049
C -3.985931691 1.8174116485 -2.4169181245
H -5.1721404119 3.4789530193 -0.6841542662
H -4.0967198538 3.3119016527 0.7173135183
H -2.7060314797 2.9604773346 -1.149487107
H -2.888178377 -0.7461956562 2.2127398293
H -1.7089807169 -2.5277192438 3.4649410167
H 0.7290777948 -2.8309024226 3.2236792477
H 2.3332698549 4.6190622174 0.2238653442
H 0.0316334121 5.4357205574 0.5640125897
H -1.8616023844 3.8641707563 0.6767655598
H -6.206986953 -3.2035841015 -0.1019903181
H -6.9646445615 -0.9508174498 0.536660539
H -1.3767378685 -0.8507918497 -1.5027428393
H -0.6976100262 -3.078023887 -2.2430655662
H -2.234803709 -5.0215600584 -1.9864966903
H -4.4890489049 -4.6862359679 -1.0302607849
H -4.3222050151 2.7196604991 -2.9361481584
H -4.8237712671 1.1179654982 -2.3713961656
H -3.1925960658 1.3568380261 -3.0119126051
C 0.9338627523 -1.2972323098 1.7200370971
C 1.7544244576 2.5421321129 0.1060653173
Pd 3.9712608923 -0.4092996209 -0.0856295173
Cl 4.6386574586 -2.6270143486 -0.2750982901
Cl 5.4295501834 0.2576970539 -1.7772629686
P 2.7503053235 -1.2119921627 1.6917628364
H 2.9740086845 -0.5082428603 2.8897735606
H 3.1195194268 -2.4944655682 2.1038911825
P 3.4315644554 1.8480412435 -0.1134516021
H 3.8210333819 2.4424494535 -1.3161855151
H 4.1623430662 2.6410931123 0.7849712739

Energy= -3246.3768741
Zero-point correction= 0.418875 (Hartree/Particle)
Thermal correction to Gibbs Free Energy= 0.356854
Sum of electronic and zero-point Energies= -3245.957999
Sum of electronic and thermal Energies= -3245.928089
Sum of electronic and thermal Enthalpies= -3245.927145
Sum of electronic and thermal Free Energies= -3246.020020

9.2. [(MotorPhos)PdCl$_2$]

st-l3-[ax]-c

C -3.9684452866 -1.3371895815 0.1615885311
C -2.8625414588 -0.9823686368 -0.5410941066
C -2.4246592218 0.4052420005 -0.8955631738
C -1.0838776928 0.6645555856 -0.5726500145
O -0.2868652148 -0.3201737917 -0.0476857034
C -0.4800737449 -1.538295286 -0.6244889575
C -1.7857516766 -1.9418857738 -0.8766006354
C -5.1446916433 -0.4289412265 0.217581087
C -5.6154333908 0.0285715748 1.4369864374
S -5.0290119915 -0.7721152373 2.899940405
C -5.1051815178 -2.4238785948 2.1232638199
C -4.0189606421 -2.6037197752 1.0488012874
C -3.1373437389 1.4770177797 -1.4407855545
C -2.5392785386 2.7226080799 -1.6033153458
C -1.2170447646 2.944651054 -1.2369378919
C 0.4165410469 -3.5577119904 -1.4824878578
C -0.8780943947 -3.9888108417 -1.7658691978
C -1.9752264317 -3.1818322411 -1.4833786812
C -5.8551571932 -0.0580234872 -0.9734251033
C -6.8873363684 0.9201121887 -0.8888116234
C -7.2313534006 1.4628165269 0.3760831162
C -6.6397250771 0.9993472942 1.5183526495
C -5.581887968 -0.6345680729 -2.2448855711
C -6.2631973771 -0.2339555823 -3.3650703576
C -7.2574553498 0.7674211841 -3.2832348995
C -7.5626825976 1.3251138251 -2.0698124706
C -2.6919059738 -2.9284809326 1.761304604
H -4.978642486 -3.162013932 2.9177509534
H -6.1118002167 -2.5306175858 1.7132585609
H -4.319690711 -3.471463497 0.4452735638
H -4.1616630043 1.347191767 -1.753222044
H -3.1207106638 3.5362244132 -2.023334332
H -0.779497003 3.9270224658 -1.3637958563
H 1.253350168 -4.2003668877 -1.726045244
H -1.0254848233 -4.9606050159 -2.2244410667
H -2.9823018689 -3.5126364978 -1.7186985561
H -8.0119443109 2.2162084756 0.4299533521
H -6.9579453615 1.3526641871 2.4935658922
H -4.8114555104 -1.3927659763 -2.3233475913
H -6.0373005599 -0.6894509901 -4.3239309377
H -7.7822229446 1.0827585421 -4.1790705986

H -8.3368169195 2.083285255 -1.9904644479
H -2.8670685466 -3.6830292936 2.5338053296
H -2.2916955148 -2.0314006143 2.2442448332
H -1.9326450656 -3.3268609208 1.0921400486
C -0.4552598807 1.8928648537 -0.7220940832
C 0.6430304516 -2.2958133554 -0.9177959469
P 1.2917696739 2.024507553 -0.1734891426
P 2.3047218957 -1.5861822124 -0.5832826041
C 1.1965742105 1.8007931622 1.6347333825
C 2.3987540347 1.6571865979 2.3371703098
C -0.0146354711 1.770373963 2.331057402
C 2.3828016152 1.4854314757 3.7163220878
H 3.3454599146 1.6840500543 1.8051519296
C -0.0244368124 1.5865989284 3.71018905
H -0.955419336 1.8866434098 1.8032801847
C 1.1732073574 1.4431313208 4.4038590671
H 3.3200659224 1.3675603642 4.2495463748
H -0.9715523815 1.5554640203 4.2395532632
H 1.1641364616 1.2961555799 5.4794863709
C 1.5209133978 3.8147195233 -0.4200104819
C 1.7863161298 4.2850038203 -1.7106613951
C 1.3323570145 4.7232899819 0.6195818801
C 1.8497240295 5.6493127466 -1.9568672974
H 1.9650045608 3.5799716507 -2.516904434
C 1.4087104879 6.0926254882 0.3708111264
H 1.1295741025 4.3695515832 1.6248318465
C 1.6622162237 6.5565415959 -0.9144674855
H 2.0635284094 6.0054444103 -2.9592726603
H 1.2695486775 6.7942600797 1.1870821354
H 1.7227352108 7.62331527 -1.1058469118
C 2.5210766644 -1.7685869439 1.2198086212
C 3.8068509893 -1.5652745017 1.7411956971
C 1.460945382 -2.033331278 2.0882263487
C 4.0235912577 -1.6535881103 3.1105818783
H 4.6321371196 -1.337528047 1.0712743914
C 1.6820662202 -2.0990316532 3.4610770961
H 0.4582679217 -2.1889942236 1.7064793151
C 2.961646266 -1.9165282042 3.9733018787
H 5.0242700712 -1.5044801929 3.5032871106
H 0.8482943166 -2.2931110381 4.1279967883
H 3.1313565266 -1.9718289748 5.0441353767
C 3.3048313598 -2.9120128519 -1.3301696611
C 3.4925839749 -2.891899375 -2.7160011714
C 3.7699816098 -3.9953336711 -0.5864885224

C 4.1333080335 -3.947910992 -3.3485608666
H 3.156632605 -2.0364061164 -3.2939591673
C 4.4227824458 -5.0490761832 -1.2239863878
H 3.6269114069 -4.0230565264 0.4884536598
C 4.6017645392 -5.0286481715 -2.6023611719
H 4.2830282467 -3.9203806065 -4.4228431632
H 4.7894739348 -5.8856934565 -0.6378667994
H 5.112027984 -5.8492934959 -3.0967796611
Pd 3.0739852451 0.6463374863 -0.9392215535
Cl 4.3429137263 2.5934273763 -0.7936281054
Cl 5.0923817937 -0.3849565907 -1.50344228

Energy= -4170.3125868
Zero-point correction= 0.753357 (Hartree/Particle)
Thermal correction to Gibbs Free Energy= 0.667067
Sum of electronic and zero-point Energies= -4169.559229
Sum of electronic and thermal Energies= -4169.509662
Sum of electronic and thermal Enthalpies= -4169.508718
Sum of electronic and thermal Free Energies= -4169.645519

TS6-c

C -3.8793881966 -1.0164172062 -0.3999482384
C -2.8063439233 -0.7018807811 -1.1827301941
C -2.171504434 0.5897667532 -1.5660970872
C -0.9333908451 0.7992100676 -0.9656034675
O -0.4124425546 -0.22562105 -0.2103364417
C -0.5049914526 -1.3983018185 -0.9285021935
C -1.7360876506 -1.7111832674 -1.5153460314
C -4.7532850955 -0.1885169335 0.5200454862
C -5.7655185412 -0.8298557739 1.2468885595
S -6.5998167074 -2.3227123932 0.8383845144
C -5.5294546643 -2.9262575011 -0.4694039291
C -4.0828490951 -2.5275578624 -0.2099209978
C -2.5647584708 1.484802137 -2.5595902068
C -1.8067236843 2.6282287286 -2.7934954807
C -0.6436699221 2.878023116 -2.0651865282
C 0.5578820091 -3.2782498443 -1.912628917
C -0.6359390803 -3.5894928136 -2.5549844226
C -1.7712600994 -2.8039177711 -2.3784801735
C -4.4754677429 1.179442632 0.960297708
C -4.8670698398 1.6185049894 2.2589909763
C -5.7349452464 0.8082678007 3.0351125833
C -6.2430689346 -0.3259976089 2.4932779986

C -3.929197713 2.1797165715 0.1278314023
C -3.6050048 3.4379309397 0.5809465096
C -3.8309061082 3.7918651992 1.9225511863
C -4.488865686 2.8988353141 2.7313453576
C -3.5029805384 -3.0354692739 1.1166289868
H -5.6289022001 -4.0149978918 -0.4736460936
H -5.8610056348 -2.5437190791 -1.4385564625
H -3.53072991 -3.0404050673 -0.9892741118
H -3.4840805412 1.306365236 -3.1093095351
H -2.1281629488 3.3430073418 -3.5433303034
H -0.090211719 3.7927091053 -2.2409996217
H 1.4256214888 -3.9043050754 -2.0758887418
H -0.6761818207 -4.4508188199 -3.2130104882
H -2.6844563242 -3.0394802102 -2.9164280221
H -6.0387146869 1.1389878821 4.0237520483
H -6.9792786329 -0.9113102781 3.0354303325
H -3.8293685175 1.9783179141 -0.9180589027
H -3.1927664412 4.1604224144 -0.1167641808
H -3.5489543211 4.7722298126 2.2914832819
H -4.7700429701 3.1752216148 3.7439759424
H -3.7076362606 -4.1038327962 1.2371925477
H -3.9057311701 -2.5108986155 1.9848732
H -2.4177059185 -2.8965303569 1.1093298836
C -0.1627275719 1.9350913272 -1.1497737528
C 0.6548746967 -2.1392335787 -1.1016139574
P 1.3927873507 2.0802114265 -0.191767184
P 2.2286464056 -1.5550044006 -0.3486974003
C 0.8516425327 1.945540399 1.544237287
C 1.8177804409 1.6528321889 2.513407582
C -0.4823911074 2.1070213496 1.9299233901
C 1.4466445561 1.5136419487 3.845952735
H 2.8584275241 1.5343195999 2.2277436346
C -0.8482661649 1.9614567317 3.2637408956
H -1.2487303242 2.3388501163 1.1983820599
C 0.1146858677 1.6597226275 4.2221088868
H 2.2023845992 1.2735756897 4.586136683
H -1.8903544978 2.0826040513 3.5416622129
H -0.1726133273 1.537354588 5.2620027015
C 1.725193441 3.8479051476 -0.4788055445
C 2.3765522839 4.2343040369 -1.6548513281
C 1.2406904887 4.8212198324 0.3935975588
C 2.5285655816 5.5808682104 -1.9574860858
H 2.7853461023 3.4794137274 -2.3195408903
C 1.4062243651 6.171525474 0.0921082165

H 0.7403411617 4.5310104293 1.3115217635
C 2.0441080501 6.5524862044 -1.0827828898
H 3.0421839836 5.8724531759 -2.8677101008
H 1.0365325295 6.9241636248 0.7812349945
H 2.1739398647 7.6048948934 -1.3148282115
C 2.0108713463 -1.7678543039 1.4526239189
C 3.1512125648 -1.6157890314 2.2550921649
C 0.7768816678 -2.0125453802 2.0561946693
C 3.0498896458 -1.7217363405 3.636475669
H 4.1145689922 -1.4130928887 1.7941764167
C 0.6790449569 -2.097695705 3.4422506852
H -0.1177273547 -2.1334364523 1.4575159071
C 1.8131372557 -1.9562881816 4.2336898785
H 3.9404733299 -1.6092044912 4.2464274402
H -0.2891877406 -2.2753329612 3.8990333184
H 1.7343608114 -2.0251975882 5.3142393149
C 3.2971515339 -2.9374989265 -0.8619377491
C 3.8730372116 -2.9003137164 -2.1358799311
C 3.4451768387 -4.0760001226 -0.0708583447
C 4.5825312109 -3.9939711946 -2.6127970033
H 3.7828590182 -2.0050499624 -2.7430444146
C 4.1684333833 -5.1672654537 -0.5481020207
H 3.0005113416 -4.1165146879 0.9178916097
C 4.733525859 -5.1293197482 -1.817766012
H 5.0335978235 -3.9537003523 -3.5987765406
H 4.2870860474 -6.0468362826 0.0765433293
H 5.2981128633 -5.9795549524 -2.187356031
Pd 3.212934259 0.6119267673 -0.4754126292
Cl 4.4935141894 2.4965588412 0.0177355763
Cl 5.2536079386 -0.5297341269 -0.5247185005

Energy= -4170.2808311
Zero-point correction= 0.753370 (Hartree/Particle)
Thermal correction to Gibbs Free Energy= 0.670416
Sum of electronic and zero-point Energies= -4169.527462
Sum of electronic and thermal Energies= -4169.478902
Sum of electronic and thermal Enthalpies= -4169.477958
Sum of electronic and thermal Free Energies= -4169.610415

a-stable-[ax]-c

C -3.9842934855 -1.5967951167 -0.6482503468
C -2.7904549681 -1.2328878148 -1.1571873834
C -2.3834312654 0.18484717 -1.3236104851

C -1.0873279589 0.5003744429 -0.9276389224
O -0.3410514072 -0.4690227967 -0.3175225956
C -0.4247340282 -1.6916665246 -0.9243885678
C -1.6459144024 -2.1389772196 -1.4353491684
C -4.9447181546 -0.602016943 -0.122166551
C -6.2319425224 -0.5988228339 -0.6204064213
S -6.7691189582 -1.7382361942 -1.8588996241
C -5.5579049248 -3.1215722424 -1.7468231668
C -4.5313718116 -3.007302139 -0.6072170123
C -3.1473969486 1.1951112515 -1.9036214539
C -2.6069328653 2.4674111829 -2.0565983057
C -1.2919293259 2.7362908211 -1.6854436593
C 0.7157497646 -3.6664490889 -1.5923097364
C -0.4628432289 -4.1115172721 -2.1866968572
C -1.6323580297 -3.3586998677 -2.1142123471
C -4.6087239202 0.247853394 0.9829780951
C -5.5632342844 1.1974471692 1.4373531941
C -6.8433301976 1.2379240207 0.8211578119
C -7.1849641909 0.3442103426 -0.1541128619
C -3.370804386 0.153578957 1.674050886
C -3.0932557016 0.9718272077 2.7389933677
C -4.0251387076 1.9476566366 3.158378387
C -5.2340831803 2.053090912 2.5199892896
C -5.1126463764 -3.3876056872 0.7594028561
H -6.1462977161 -4.0362219991 -1.6363845704
H -5.0377427205 -3.1522590497 -2.7065856297
H -3.7339738834 -3.719079582 -0.8347592452
H -4.1598212601 0.9797030152 -2.2287406408
H -3.2066945746 3.2569863207 -2.4959535901
H -0.8848566873 3.725971519 -1.8528930121
H 1.6067218503 -4.2814474094 -1.6369831927
H -0.4697870975 -5.0619962712 -2.7090440221
H -2.5339332851 -3.7167371855 -2.5999071582
H -7.5694480948 1.9678803142 1.1674530553
H -8.1843380073 0.346606991 -0.5779612497
H -2.642066644 -0.5895645377 1.3709242576
H -2.1484368178 0.8563875795 3.2617639305
H -3.7892790058 2.5989745068 3.9941220749
H -5.9681141338 2.78446836 2.8468617719
H -5.4115444271 -4.4400299717 0.7576741295
H -5.9874932197 -2.7860947458 1.0167028935
H -4.3634757382 -3.2465806848 1.5435880048
C -0.4879531038 1.7347028743 -1.1256368871
C 0.7539938738 -2.4206268437 -0.9598637218

P 2.2048539326 -1.6688118844 -0.1264457898
P 1.2859658063 1.9641031873 -0.701025771
C 1.2499111974 2.4141731911 1.0685464823
C 0.0730323493 2.3922696024 1.8193191103
C 2.4548106639 2.7843980251 1.6832987861
C 0.0999693718 2.7402536659 3.16762107
H -0.8704714658 2.1109879066 1.3628835862
C 2.47108597 3.1385108826 3.0269132451
H 3.3749281116 2.8019173255 1.1050351586
C 1.2943110294 3.1151658313 3.7728905455
H -0.8219624844 2.7300008948 3.74073025
H 3.4079295262 3.4311568695 3.4901634402
H 1.3095324261 3.3908405509 4.8229224933
C 1.5644814359 3.5303627282 -1.5878847184
C 1.7652103893 3.4697771062 -2.9703410272
C 1.4838728229 4.7691570702 -0.9551330324
C 1.87707585 4.6378198727 -3.710706125
H 1.856403827 2.5053663377 -3.4615815667
C 1.6050454306 5.9404071775 -1.7010377375
H 1.3289003967 4.826932312 0.1169455413
C 1.7980138577 5.8769254804 -3.0760894505
H 2.041473796 4.5827379185 -4.7818299129
H 1.5485184006 6.9022980481 -1.2014708936
H 1.8946515799 6.7903321066 -3.6544887059
C 1.6602230982 -1.4902043892 1.6072635471
C 2.3802655209 -0.6177360193 2.4293950109
C 0.5361500556 -2.1450988278 2.119516868
C 1.9667014239 -0.3890485598 3.7375107068
H 3.2596042757 -0.1103813135 2.0436480976
C 0.1213427724 -1.907451912 3.4256242893
H -0.0272172409 -2.8329320072 1.4967367627
C 0.8317552816 -1.0228108072 4.2335670978
H 2.523365758 0.3042385513 4.3583897978
H -0.7597423414 -2.4116159125 3.8102579843
H 0.5018332051 -0.8307840665 5.2498528229
C 3.3216266546 -3.1059622669 -0.1477796085
C 4.0145270592 -3.4040890907 -1.3265210886
C 3.4201426951 -3.9643789349 0.9457846246
C 4.7845502839 -4.5559356041 -1.4103408712
H 3.9691482613 -2.7204598725 -2.1688429017
C 4.2046170322 -5.113305712 0.8617810967
H 2.8933218109 -3.738566579 1.8668400967
C 4.8819517713 -5.4124962205 -0.3142774539
H 5.3248788309 -4.7756723979 -2.3252311264

H 4.2845459969 -5.7718082473 1.7207438232
H 5.4939376563 -6.3067627918 -0.3771966545
Pd 3.11402205 0.4074717169 -0.8073751301
Cl 4.3730688491 2.2958726598 -1.3549216832
Cl 5.1594501466 -0.5896835093 -0.3196666752

Energy= -4170.3310393
Zero-point correction= 0.753302 (Hartree/Particle)
Thermal correction to Gibbs Free Energy= 0.666845
Sum of electronic and zero-point Energies= -4169.577737
Sum of electronic and thermal Energies= -4169.527972
Sum of electronic and thermal Enthalpies= -4169.527027
Sum of electronic and thermal Free Energies= -4169.664195

a-stable-[ax]-i

C -3.92317897 -1.612158867 -0.0500809807
C -2.6777373113 -1.3454685173 -0.4951120104
C -2.2521882659 -0.0038186735 -0.9742581905
C -1.0007667058 0.4251987712 -0.5515901544
O -0.259768496 -0.3970674403 0.2688001265
C -0.3050598539 -1.729303573 -0.0705981721
C -1.5215290864 -2.2837746573 -0.4749214187
C -4.9954140939 -0.5882220416 -0.0082060373
C -6.1806449869 -0.8641971456 -0.6643738032
S -6.4808762886 -2.379897967 -1.5181063749
C -5.2523777416 -3.5466343512 -0.8068166072
C -4.4190393312 -2.9858180487 0.3559143758
C -2.9589124503 0.8321895789 -1.8414652655
C -2.4194899991 2.0518646358 -2.2323505484
C -1.1601039301 2.4540783137 -1.7901282541
C 0.8733348985 -3.7684718577 -0.4448455675
C -0.3182735137 -4.3502219152 -0.8655683887
C -1.4982473161 -3.6147021166 -0.8985806503
C -4.88441155 0.6156027557 0.7634429855
C -5.9320459235 1.5746708389 0.7066272894
C -7.0919936883 1.2881339173 -0.0612334551
C -7.2311541014 0.0899328269 -0.7000217081
C -3.7732514875 0.8864872633 1.6065216116
C -3.7001338103 2.0547323677 2.3211665073
C -4.7222744225 3.0251896357 2.2285340935
C -5.8163842137 2.7829633314 1.4402485691
C -5.1895651446 -2.9701205925 1.6815510297
H -5.813314706 -4.4267639292 -0.4813246447

H -4.59592962 -3.8361296887 -1.6299406939
H -3.5662372486 -3.6578647142 0.4817055668
H -3.9299147328 0.5189941863 -2.2092326687
H -2.9733165879 2.6883425222 -2.9134233231
H -0.7281812564 3.3815287242 -2.1487900748
H 1.7952712165 -4.3357024689 -0.4924661715
H -0.3170988077 -5.3775493559 -1.2126637152
H -2.3982278159 -4.0694103509 -1.2967707392
H -7.8893924456 2.0244782807 -0.1039582714
H -8.1422785236 -0.1430736724 -1.242254033
H -2.9814668624 0.1505071937 1.6932365896
H -2.8520206248 2.230845635 2.9749566377
H -4.6444690538 3.9494486109 2.79172053
H -6.6212617534 3.5098792546 1.3745025161
H -5.441805541 -3.9925552302 1.9784108789
H -6.1174689014 -2.3976887977 1.6119368542
H -4.5775491483 -2.5263848243 2.4719002726
C -0.4262890213 1.6355023261 -0.9338178688
C 0.8979665092 -2.4331249339 -0.0397542502
P 2.4304200834 -1.4682817505 0.2079208498
P 1.3118295152 1.8807724316 -0.457344669
C 2.4814104951 -1.0191308201 1.9839830667
C 3.7387004582 -0.7410895939 2.5362498765
C 1.3601349381 -1.0055005132 2.8143648872
C 3.8735699811 -0.4798060745 3.8928580078
H 4.623986317 -0.7586026067 1.9075433183
C 1.4974899073 -0.7341589178 4.1730144146
H 0.3749837913 -1.2069880884 2.4129659847
C 2.7512157511 -0.480056205 4.7168309851
H 4.8569804484 -0.280866896 4.3067905149
H 0.6163527165 -0.7244346997 4.8062524239
H 2.8536345973 -0.2782019256 5.7784391897
C 3.8115380425 -2.6591129355 0.2070400567
C 4.958454476 -2.4307609582 -0.5538391873
C 3.7763420621 -3.7453147843 1.0910021033
C 6.0494490949 -3.2892688269 -0.4443842033
H 4.9893974075 -1.5990805771 -1.2478960543
C 4.8591253018 -4.6104823719 1.182881743
H 2.9052035985 -3.9082129844 1.719482122
C 5.998903168 -4.3818471561 0.4142686608
H 6.9352873428 -3.1053094205 -1.0432261167
H 4.8175586285 -5.4560632768 1.8618700527
H 6.8477979448 -5.0542243949 0.4895242746
C 1.2568217241 2.100820296 1.3532958871

C 0.0610572638 2.2446731308 2.0585787854
C 2.472373677 2.2795124108 2.0183650119
C 0.0860958998 2.5707015176 3.411773342
H -0.8916853271 2.1212950591 1.552819562
C 2.4932179706 2.6073090953 3.3671261556
H 3.4066384297 2.1676574246 1.4744200163
C 1.298267153 2.7562797823 4.0664706602
H -0.8455072272 2.6884733104 3.9572538592
H 3.4429496237 2.7371996353 3.8750272537
H 1.3143601971 3.0126041296 5.1210841691
C 1.7847604425 3.5777730985 -0.91785017
C 0.9925715873 4.6510005494 -0.4928957775
C 2.9875445144 3.8283673598 -1.5793692538
C 1.3849291558 5.9574136211 -0.7569737183
H 0.0717309256 4.4671672421 0.053535246
C 3.3854771516 5.1392620293 -1.8255690794
H 3.5945181067 2.9999963182 -1.9266964679
C 2.5838865203 6.2024398058 -1.423107189
H 0.7594189659 6.7836858032 -0.4347634863
H 4.3194175284 5.3256329114 -2.345476597
H 2.8928892375 7.22319333 -1.6255866045
Pd 2.3234801076 0.0829951073 -1.5144182153
Cl 1.9604764556 1.3652391161 -3.4419452241
Cl 2.8167646689 -1.7658665975 -2.8713116803

Energy= -4170.3386952
Zero-point correction= 0.753350 (Hartree/Particle)
Thermal correction to Gibbs Free Energy= 0.669476
Sum of electronic and zero-point Energies= -4169.585345
Sum of electronic and thermal Energies= -4169.535899
Sum of electronic and thermal Enthalpies= -4169.534955
Sum of electronic and thermal Free Energies= -4169.669220

9.3. [(MotorPhos)PdCl_2] in DMSO

a-stable-[ax]-i

C -3.9297287271 -1.6477745644 0.0346205567
C -2.6929577754 -1.3837974617 -0.4359657318
C -2.2796829251 -0.048374958 -0.9465459133
C -1.0276109926 0.3968035332 -0.5388573929
O -0.2732766796 -0.405412772 0.2855090977
C -0.3118825749 -1.7422124508 -0.0370860127
C -1.5296786732 -2.3143385938 -0.4103245471

C -5.0120192518 -0.6321431737 0.0529142399
C -6.1968086415 -0.9409111528 -0.590338679
S -6.4691316784 -2.4907204921 -1.3949467136
C -5.256069882 -3.6318764301 -0.6096490507
C -4.4050888214 -3.0080415849 0.505770321
C -2.9980226343 0.7753546154 -1.8166466547
C -2.4732352877 2.0000944475 -2.2177639866
C -1.2139285446 2.4185982029 -1.788739308
C 0.8817878025 -3.7785435484 -0.3842675275
C -0.3135690504 -4.3798157423 -0.7672338482
C -1.5022849969 -3.6555910082 -0.8019277282
C -4.9106075785 0.6001997579 0.7803769221
C -5.967249333 1.5483936092 0.6870267349
C -7.1281333453 1.2238985488 -0.0652884981
C -7.2573961934 0.0005963212 -0.6590775724
C -3.7978794633 0.9153938799 1.6076245576
C -3.7284787556 2.1170230176 2.2675549482
C -4.7599553169 3.0748812058 2.1362441794
C -5.8588881738 2.7894420608 1.3674185054
C -5.1476169435 -2.9388895999 1.8445043715
H -5.8293347461 -4.476096057 -0.2192507006
H -4.6151441085 -3.9889696332 -1.4171071318
H -3.5482219482 -3.6716471956 0.6422308599
H -3.9699374411 0.455659079 -2.1772698093
H -3.0433286199 2.6321210755 -2.8901746329
H -0.8100044711 3.3589446691 -2.1465739604
H 1.8013143829 -4.3511966201 -0.4158108735
H -0.3128284051 -5.4202575695 -1.0736689562
H -2.4039621922 -4.1336071467 -1.1668028899
H -7.9308710673 1.9525797456 -0.1338417052
H -8.1688544055 -0.2558841687 -1.1912504076
H -3.0021619993 0.1886132675 1.7309406014
H -2.8767211699 2.3324184003 2.9048951999
H -4.684698967 4.0243733499 2.6568393151
H -6.6711961209 3.5051039174 1.2735647058
H -5.373737098 -3.9517662476 2.1911990724
H -6.0899070549 -2.3888900967 1.7706704704
H -4.5272600016 -2.4497700913 2.6018182448
C -0.4626211268 1.6067031172 -0.9387410905
C 0.9003724452 -2.4305360499 -0.0184715763
P 2.4101373142 -1.4302039215 0.2057502597
P 1.2765576129 1.8479782335 -0.4635782419
C 2.4834807959 -0.9623668952 1.967321905
C 3.7360907925 -0.5988389968 2.480680377

C 1.3897129411 -1.053763936 2.8301264137
C 3.8939633828 -0.3520487177 3.8378526468
H 4.5976578901 -0.5256197323 1.8235311263
C 1.5509610947 -0.7936195625 4.1892666011
H 0.410897953 -1.3387673362 2.4632077609
C 2.8007150105 -0.4512338062 4.6963064354
H 4.8708731577 -0.0796234092 4.2242790544
H 0.6943879985 -0.868146389 4.8516154124
H 2.9224830505 -0.2584782217 5.7576496123
C 3.8481148466 -2.5438187296 0.1558010724
C 4.9901303863 -2.1921659341 -0.566559274
C 3.872708028 -3.6753203351 0.9814092182
C 6.1348914868 -2.9814985341 -0.4903183169
H 4.9858527689 -1.3039512777 -1.1888050255
C 5.0115353171 -4.4708743897 1.0399030244
H 3.0102957142 -3.931889958 1.5901039922
C 6.1432976888 -4.1254375827 0.3029011327
H 7.0192822214 -2.6999394122 -1.0530510995
H 5.0184366833 -5.3535810071 1.6713201164
H 7.034316509 -4.743442309 0.3554930673
C 1.2523433668 2.102192067 1.3372022547
C 0.0609573432 2.2110346008 2.057566954
C 2.4723572341 2.3602302686 1.970201196
C 0.0937981537 2.5733609078 3.4018672754
H -0.8961752027 2.0348336476 1.5778567333
C 2.4986762579 2.7267936188 3.3100299852
H 3.4027035253 2.2816615159 1.4136399581
C 1.3087288019 2.834867858 4.0278582591
H -0.8339943332 2.6567428865 3.9592378095
H 3.4493279911 2.9209766038 3.7959833919
H 1.3308190118 3.1201099431 5.0750018901
C 1.8183582604 3.5061608841 -0.9721901598
C 1.0302473753 4.6211477165 -0.6618296382
C 3.0893541395 3.6873139355 -1.5218895785
C 1.4951187385 5.9012575744 -0.9438185366
H 0.0599808592 4.4953134128 -0.1897367008
C 3.5572589132 4.9717915349 -1.7855889299
H 3.7104502532 2.8268315766 -1.7478089708
C 2.7581216477 6.0775468684 -1.50658886
H 0.8746186146 6.7612029908 -0.7126204209
H 4.5464616014 5.1061867119 -2.2116909255
H 3.1219693731 7.0780361211 -1.7192995297
Pd 2.2678454586 0.0631746251 -1.5665751663
Cl 1.9048828878 1.3461565389 -3.5593978085

Cl 2.7864966355 -1.8137176614 -2.9661637574

Energy= -4170.4047685
Zero-point correction= 0.753072 (Hartree/Particle)
Thermal correction to Gibbs Free Energy= 0.669321
Sum of electronic and zero-point Energies= -4169.651697
Sum of electronic and thermal Energies= -4169.602166
Sum of electronic and thermal Enthalpies= -4169.601222
Sum of electronic and thermal Free Energies= -4169.735448

a-stable-[ax]-c

C -4.1443398188 -1.2716232724 -0.4963547556
C -2.8896517888 -1.0409024105 -0.9358519569
C -2.3446713766 0.3166833273 -1.1951309776
C -1.0424531746 0.5459807457 -0.7562907955
O -0.385436402 -0.4453708006 -0.0779395181
C -0.5593188015 -1.6822009392 -0.6408081253
C -1.8200504667 -2.0598972551 -1.1072748956
C -5.1184833675 -0.1794638709 -0.2515882474
C -6.3338804238 -0.2383407449 -0.9086227284
S -6.7730113326 -1.5671182362 -1.9881249423
C -5.660637538 -2.9473911402 -1.4895597309
C -4.7504402636 -2.6454211265 -0.290954892
C -2.9872385696 1.3535111949 -1.8703375656
C -2.3419629466 2.5727460968 -2.0486403952
C -1.03431949 2.7621194317 -1.6100738663
C 0.4291150818 -3.7479260953 -1.2805358226
C -0.8012533499 -4.143968431 -1.7963600366
C -1.9125639416 -3.3080982511 -1.7270027439
C -4.8866636236 0.8682245052 0.7005147636
C -5.8429094976 1.9140916206 0.826130749
C -7.0363705209 1.8604673097 0.0567387858
C -7.29371489 0.7976112591 -0.761780269
C -3.7418807301 0.9007423881 1.5429719618
C -3.5478485983 1.9317242224 2.427978292
C -4.4786643708 2.9918328367 2.5187514849
C -5.6053809522 2.9762746004 1.7372592658
C -5.4782762817 -2.7734929104 1.0512944162
H -6.3057518752 -3.8011347414 -1.2683552487
H -5.0583946157 -3.1829904461 -2.3683929379
H -3.9625894457 -3.4019150653 -0.3084356529
H -3.9938824531 1.2068035016 -2.2471556867
H -2.8544895969 3.3836485534 -2.5547984865

H -0.5549575438 3.7166563741 -1.7866954724
H 1.2710761148 -4.4257679215 -1.3426825696
H -0.8929092118 -5.1173352806 -2.2659716119
H -2.8471801405 -3.6266283801 -2.1744495692
H -7.7619873545 2.6628029544 0.1553050424
H -8.2307170903 0.741589833 -1.3081029476
H -3.0227396247 0.0900523582 1.4974967901
H -2.6747650338 1.9288532242 3.0724425975
H -4.3053052238 3.8044973366 3.2171807197
H -6.3422476981 3.7716223672 1.8104455757
H -5.8018406592 -3.808687178 1.1958363459
H -6.3619304118 -2.1319965404 1.1095974782
H -4.8102647525 -2.5068995121 1.8760731695
C -0.3420492877 1.7224792036 -0.9746799678
C 0.5736413799 -2.4781186551 -0.710692496
P 2.1111752601 -1.8158725091 0.0258752214
P 1.413047253 1.830608675 -0.4630977197
C 1.3822839333 2.1222190667 1.3339612399
C 0.2298561898 1.9465483225 2.1029646613
C 2.583848117 2.4820780523 1.9619127851
C 0.2817745493 2.1236647393 3.4842700827
H -0.7144538034 1.6816281096 1.6388572432
C 2.6231945082 2.679803054 3.3364214218
H 3.4874254589 2.6135222737 1.3727804309
C 1.4725481236 2.4937234227 4.1014091192
H -0.6139389821 1.9742401061 4.0788170516
H 3.5555666782 2.9682993115 3.8112252958
H 1.5064440527 2.6338712186 5.1772667488
C 1.8212395662 3.4284329897 -1.2332645299
C 2.0026552086 3.4498256326 -2.6213917489
C 1.8431697177 4.6225787759 -0.5145124415
C 2.2078086775 4.6542914198 -3.2802441097
H 1.983182903 2.5218513564 -3.1862079581
C 2.0610107337 5.8298811828 -1.1790272069
H 1.6869196155 4.6256517791 0.5589675368
C 2.2419865289 5.8480564168 -2.5577965104
H 2.3470855952 4.6631574615 -4.3566758882
H 2.0811830094 6.7557829789 -0.6128203698
H 2.4083568447 6.7894572207 -3.0722892986
C 1.7459189229 -1.7183923501 1.8056125848
C 2.5925547671 -0.9579023389 2.6197344654
C 0.6637745513 -2.3953267864 2.3777995499
C 2.3584348402 -0.8757155035 3.9885396523
H 3.4320998259 -0.424701178 2.1840660904

C 0.4266031756 -2.2992444989 3.745687305
H 0.0020274844 -2.9980885325 1.7640939187
C 1.2712769282 -1.5396323877 4.551729471
H 3.0184248932 -0.2802539125 4.6107521506
H -0.4198805116 -2.8208823395 4.1809001289
H 1.0816542779 -1.4643905769 5.6179855364
C 3.1880219994 -3.2682317617 -0.1841517011
C 3.672594839 -3.5566118828 -1.4657752271
C 3.4742472242 -4.1291495223 0.8739118857
C 4.4325134915 -4.698270693 -1.6835593241
H 3.4544961936 -2.8867890796 -2.2933926869
C 4.2475242655 -5.269002853 0.6529783085
H 3.1026507535 -3.9200612664 1.871806751
C 4.7246652557 -5.5556298682 -0.6215461806
H 4.8027944689 -4.9170872433 -2.68017804
H 4.4717059001 -5.9324998069 1.4823550423
H 5.3240095926 -6.4447860641 -0.7911337866
Pd 3.1681300554 0.2051712787 -0.6377515874
Cl 4.5900391638 2.0081808745 -1.2688671646
Cl 5.2145388623 -0.9333848043 -0.2050207252

Energy= -4170.3929245
Zero-point correction= 0.752298 (Hartree/Particle)
Thermal correction to Gibbs Free Energy= 0.667420
Sum of electronic and zero-point Energies= -4169.640627
Sum of electronic and thermal Energies= -4169.590762
Sum of electronic and thermal Enthalpies= -4169.589818
Sum of electronic and thermal Free Energies= -4169.725504

a-stable-[ax]-e

C 3.8340147393 -1.4785741137 0.5746313646
C 2.6164818069 -0.9734667953 0.8618797075
C 2.3016977559 0.4834112544 0.8099497612
C 1.0773428921 0.7979358569 0.2325431554
O 0.3667848623 -0.2254575581 -0.3383018876
C 0.2353608696 -1.3236214818 0.4633968615
C 1.3812198845 -1.7740335257 1.1300949768
C 4.9785253178 -0.6168382297 0.1898012903
C 6.1476788952 -0.7247627391 0.9205150825
S 6.34737859 -1.8670431355 2.2547339538
C 5.007602194 -3.1035455858 2.0029314207
C 4.2156897554 -2.9392122111 0.6990473084
C 3.0373001355 1.5396593031 1.3482983579

C 2.5410513497 2.8400762334 1.2743622305
C 1.2672916923 3.1023934965 0.7695807825
C -1.118161814 -3.0585191168 1.3632726495
C -0.0138786796 -3.5116146612 2.0750749792
C 1.2210968343 -2.8662880517 1.9817197169
C 4.9424690304 0.2516437108 -0.9524622664
C 6.0517113222 1.1039316818 -1.2100693842
C 7.1989785174 1.0212426843 -0.3755155935
C 7.2618555219 0.1098026394 0.639938285
C 3.8411507671 0.294742173 -1.850437386
C 3.8255243568 1.1689092655 -2.9083709595
C 4.9111338573 2.0462913126 -3.1341519291
C 6.0040905335 2.0041995242 -2.3065346703
C 4.9697315427 -3.4616534182 -0.5275600408
H 5.4871594053 -4.0849332034 2.0339420232
H 4.3380601676 -3.0131976652 2.859795373
H 3.315056788 -3.5465493311 0.813617954
H 3.9957343129 1.3477925803 1.8193447642
H 3.1345326068 3.6602156315 1.6641256579
H 0.8845089372 4.1159857248 0.7943417238
H -2.0547692932 -3.6024736621 1.4272109275
H -0.112573099 -4.382525282 2.7139425327
H 2.0539417207 -3.2230694082 2.577730816
H 8.0435562115 1.6743525024 -0.575851372
H 8.1608425103 0.0242181567 1.2433529847
H 3.0071938802 -0.3822035483 -1.7024678936
H 2.9735821287 1.1820719299 -3.5818177592
H 4.8817853122 2.7381466179 -3.9701124377
H 6.856423506 2.6555610889 -2.4804512081
H 5.1331135275 -4.5388462686 -0.4266656943
H 5.9442546807 -2.9820759023 -0.654413306
H 4.3857552949 -3.2889228119 -1.4367473881
C 0.4843205551 2.0566362442 0.2727456431
C -1.0239540592 -1.9041326107 0.5714838656
P -2.4439433794 -1.228530484 -0.3729129487
P -1.3280292149 2.0414522951 0.035764773
C -3.8331132195 -0.8770885602 0.7405343453
C -4.8473701685 -0.0694650818 0.2092953363
C -3.9906396206 -1.4357779291 2.0103350205
C -6.0171318701 0.1508990416 0.9283169274
H -4.7200211703 0.3865495213 -0.7695711881
C -5.1586427189 -1.201725278 2.730801112
H -3.2078828416 -2.0388969847 2.454934984
C -6.1754058229 -0.4195578543 2.1893466306

H -6.7992654497 0.7735920182 0.5059035864
H -5.2696342467 -1.6308820875 3.7214744583
H -7.0855547812 -0.2451126087 2.7546150994
C -2.9827657641 -2.7235812625 -1.2695903171
C -2.0116763729 -3.5347245308 -1.8693948035
C -4.3318107251 -3.0629554453 -1.3880216064
C -2.3879986805 -4.6720342788 -2.572980208
H -0.9588798676 -3.2796697361 -1.7901307648
C -4.7043829741 -4.2038244969 -2.0965504823
H -5.0996227442 -2.4489360334 -0.9295192984
C -3.7363578907 -5.0089430787 -2.6886423843
H -1.6274610627 -5.2955334229 -3.0323367238
H -5.7554128497 -4.4623051744 -2.1789794054
H -4.0295256087 -5.8987079201 -3.2372542357
C -1.9111607012 1.6531548976 1.727922009
C -3.1985430022 2.0701554286 2.0933752232
C -1.1304972429 0.964110678 2.6602483896
C -3.6849813224 1.8149183098 3.3689939306
H -3.8231396308 2.6028999534 1.3836854104
C -1.6273709739 0.6986007434 3.9346807457
H -0.1220132346 0.6459052117 2.4231413189
C -2.9004755956 1.1275220973 4.2937119767
H -4.6821493134 2.1477958664 3.6379066255
H -1.0062500055 0.1664329089 4.6481007594
H -3.2827876748 0.9268590312 5.2896772538
C -1.9660913001 3.7324606887 -0.1669114439
C -2.8327210709 4.0413069195 -1.2188225746
C -1.7036545281 4.7003802499 0.813315565
C -3.3961322729 5.3113230071 -1.3130304545
H -3.0697037236 3.2919741581 -1.9653932311
C -2.2546513851 5.972029367 0.7056033016
H -1.0952745441 4.4604148273 1.6791594482
C -3.0985529499 6.2806831795 -0.3599285136
H -4.0698940207 5.5386258457 -2.1330007583
H -2.0368517229 6.7167159794 1.4643973176
H -3.5351012401 7.2716231021 -0.4372024925
Pd -1.8564744135 0.5971457641 -1.716296622
Cl -2.4583522336 -0.6064862362 -3.6838737392
Cl -0.7585169574 2.239064808 -3.0857336051

Energy= -4170.3961068
Zero-point correction= 0.752308 (Hartree/Particle)
Thermal correction to Gibbs Free Energy= 0.666386
Sum of electronic and zero-point Energies= -4169.643799

Sum of electronic and thermal Energies= -4169.593899
Sum of electronic and thermal Enthalpies= -4169.592955
Sum of electronic and thermal Free Energies= -4169.729721

a-metastable-[eq]-i

C -3.9140888038 -1.6249862794 0.1467457565
C -2.6983307894 -1.3941926474 -0.3867282674
C -2.2946545413 -0.0678617429 -0.9361681917
C -1.0485999444 0.3882833622 -0.5193865615
O -0.2905399263 -0.409706047 0.3089474183
C -0.3121692274 -1.7408306887 -0.0380233709
C -1.5286169328 -2.3140984008 -0.4052913857
C -5.0011234882 -0.6111533435 0.0705352693
C -6.1554050182 -0.9831674576 -0.5943336612
S -6.3095016573 -2.5811710332 -1.3446341899
C -5.2785989877 -3.6592122382 -0.2610663246
C -3.0093816537 0.7434647341 -1.8195511886
C -2.4910558547 1.9720891299 -2.2193021834
C -1.2443337499 2.4076807502 -1.7718322593
C 0.8942099967 -3.7449071806 -0.4971890156
C -0.2996309438 -4.3376228157 -0.9014108234
C -1.4962886702 -3.6261584955 -0.8784184927
C -4.9442517755 0.6591029608 0.7276588777
C -6.0193911722 1.5748801943 0.5506800459
C -7.1512406935 1.1813517269 -0.2123661361
C -7.235039036 -0.0754784194 -0.7446005175
C -3.8516987784 1.0506855959 1.5498862334
C -3.8182863184 2.2940530336 2.1294706184
C -4.8714284458 3.2154881478 1.9222615686
C -5.9505082637 2.8576829304 1.1559553851
H -5.9454122954 -4.3709079828 0.2300860472
H -4.6125994185 -4.2066883568 -0.9316395629
H -3.9751469494 0.4147775962 -2.1882462158
H -3.0591658558 2.5956323432 -2.901264061
H -0.8474154527 3.3535569044 -2.1230248268
H 1.8187376288 -4.3062224404 -0.5674587752
H -0.2905219796 -5.3595041727 -1.2648489149
H -2.4079086626 -4.0847106804 -1.245363199
H -7.9681835972 1.8855546071 -0.3416716718
H -8.1231178859 -0.3829686438 -1.2885113427
H -3.0412848462 0.3512928374 1.7275508317
H -2.9784560799 2.5739356054 2.7582620633
H -4.8262628993 4.1979939245 2.3815578456

H -6.77513867 3.5487122929 1.0034976691
C -0.4947001615 1.6053464443 -0.9107711164
C 0.9068644954 -2.4157426869 -0.0659262212
P 2.4067181434 -1.4095294946 0.1829165421
P 1.2314047333 1.8701815833 -0.4040035284
C 1.1722229125 2.0752752506 1.4031777243
C -0.0324920927 2.1268514814 2.1079675245
C 2.3739033162 2.3480803332 2.0640625522
C -0.0317773828 2.4443532517 3.4635955183
H -0.9768331996 1.9402073637 1.6082099866
C 2.3685600004 2.6713300865 3.4154782187
H 3.3145613501 2.3150395689 1.5205710505
C 1.1654113839 2.7197843525 4.1175720418
H -0.9713045344 2.4795028048 4.0066137641
H 3.3055313347 2.8771846678 3.9227385961
H 1.1634898217 2.9694089754 5.1740153744
C 1.7453293003 3.55190523 -0.8606465801
C 3.024935223 3.7740978488 -1.3740592916
C 0.9238246716 4.641180495 -0.5456910437
C 3.4696189455 5.0745351447 -1.5964367747
H 3.6699193032 2.9327079718 -1.6053970978
C 1.366159465 5.9376129624 -0.7863870033
H -0.055109357 4.4826385073 -0.1022152791
C 2.6385410076 6.1551965955 -1.3123678441
H 4.4654762685 5.240941 -1.9948135645
H 0.7204105582 6.7778677399 -0.5520240207
H 2.9843580452 7.1683489733 -1.49281167
C 2.4767430356 -0.9797292303 1.9539865738
C 3.7214096118 -0.5869428806 2.4649246179
C 1.3947615776 -1.1283668469 2.8235840987
C 3.8837043957 -0.3667096964 3.8260799912
H 4.5738960192 -0.4697641266 1.8021432509
C 1.5597624562 -0.8932593386 4.187016324
H 0.4232808139 -1.4412460827 2.4594365604
C 2.8021311882 -0.5212339841 4.6911417619
H 4.8546115336 -0.0709489384 4.2104694043
H 0.7125141212 -1.011930524 4.8549003058
H 2.9272689203 -0.3484733915 5.7555191036
C 3.8533333378 -2.5103166961 0.1083983911
C 5.0009291709 -2.1236372241 -0.5868227908
C 3.87652549 -3.6721281617 0.8903896962
C 6.1503598409 -2.9074411396 -0.5271740185
H 4.9978674245 -1.2122210961 -1.1747052913
C 5.0206308943 -4.4613464011 0.9322906149

H 3.0089820369 -3.9586028644 1.4779222778
C 6.1580462993 -4.0807802066 0.2220032953
H 7.0388642597 -2.5989729474 -1.0689291141
H 5.0264616257 -5.3674505378 1.5296472517
H 7.0527579104 -4.6944204404 0.2611464838
Pd 2.2628147924 0.1313852082 -1.5459202999
Cl 1.9302480472 1.472818725 -3.5059437714
Cl 2.8053510937 -1.7032370746 -2.991345926
C -3.5205225218 -3.8254472683 1.5509585727
H -2.9349963766 -4.4771387504 0.9024809919
H -2.8299755345 -3.2499931762 2.1747647874
H -4.1159479554 -4.4648015845 2.2098332963
C -4.4648939528 -2.8925737736 0.7895001723
H -5.1791574082 -2.5244585017 1.5342721897

Energy= -4170.3973561
Zero-point correction= 0.753100 (Hartree/Particle)
Thermal correction to Gibbs Free Energy= 0.669739
Sum of electronic and zero-point Energies= -4169.644256
Sum of electronic and thermal Energies= -4169.594714
Sum of electronic and thermal Enthalpies= -4169.593770
Sum of electronic and thermal Free Energies= -4169.727617

a-metastable-[eq]-c

C 4.0824043258 -1.3407258427 0.3178861652
C 2.8621366302 -0.9479110233 0.7283223347
C 2.4368859502 0.4811448516 0.7914869692
C 1.1616721681 0.7302791615 0.2832841984
O 0.5071229577 -0.3106917478 -0.3292215924
C 0.4903213416 -1.4089171987 0.4969131423
C 1.6918928675 -1.7991464925 1.088823788
C 5.1405596025 -0.3455629381 -0.0011751813
C 6.3130727815 -0.4170681262 0.7284662176
S 6.5372513029 -1.6194751692 2.0104337712
C 5.4759682886 -3.027616381 1.473439251
C 3.1028162888 1.531781048 1.4172565343
C 2.4885826212 2.778216298 1.5247349244
C 1.1811065228 2.9685683457 1.09000106
C -0.7530483588 -3.072703211 1.6492424199
C 0.4165244503 -3.4537071909 2.3013777979
C 1.6298958477 -2.819402994 2.035649805
C 5.0302603481 0.6007698167 -1.0697119519
C 6.0745044238 1.5478904923 -1.2633176228

C 7.2291481212 1.4876995138 -0.4377683536
C 7.3622379743 0.5134125033 0.5125585821
C 3.9129596608 0.6376913612 -1.9484373661
C 3.823599003 1.587356732 -2.9345328741
C 4.8470486335 2.5495926586 -3.1024089608
C 5.9505633818 2.5226866689 -2.2885247457
H 6.1307702634 -3.88634826 1.3113428929
H 4.8101601151 -3.2448810637 2.3116813479
H 4.0918856543 1.3723447435 1.8339391162
H 3.0150680692 3.5968303639 2.0034748198
H 0.6951067897 3.9215020424 1.2663285129
H -1.6793833738 -3.5930760638 1.8662972446
H 0.3812752488 -4.2556158846 3.0311188969
H 2.5256671792 -3.1106555025 2.5737436821
H 8.0233439011 2.2127652841 -0.5914017969
H 8.2666427253 0.4514368705 1.110417221
H 3.1268465976 -0.1015381784 -1.8385919021
H 2.9629805534 1.5969074621 -3.5966968203
H 4.7599992977 3.2986943529 -3.8832420403
H 6.7524877348 3.2444794747 -2.4183508358
C 0.4769857668 1.9199281923 0.4794101733
C -0.7351547327 -2.008153649 0.7400229842
C 3.7231922978 -3.9146524824 -0.1150806722
H 3.1516170958 -4.2628224044 0.7454318716
H 3.0200183551 -3.6408460867 -0.9072674065
H 4.3245681647 -4.7553888801 -0.4739349244
C 4.656483561 -2.7463738797 0.2082477785
H 5.3651520217 -2.6882385996 -0.6253103184
P -2.1385421666 -1.5247887295 -0.3331710261
P -1.3392788786 1.9556483808 0.3134537649
C -3.5731626191 -2.1930859348 0.5524589441
C -4.555745092 -2.9662194496 -0.0734595699
C -3.7214792671 -1.846980131 1.9003915762
C -5.669643127 -3.3887582537 0.6465340332
H -4.4564099373 -3.2379277294 -1.118099853
C -4.839221384 -2.26625883 2.6134786095
H -2.9583205436 -1.2610789731 2.4020384425
C -5.8156270216 -3.0373610142 1.9868375188
H -6.4260063829 -3.9929538791 0.1556243766
H -4.9423289043 -1.9898071518 3.6581873757
H -6.6877910928 -3.3672233881 2.5428498813
C -1.7394107842 -2.6228836151 -1.7354999917
C -1.213889196 -2.0762329996 -2.9092033681
C -1.8145272496 -4.0128539208 -1.5933841102

C -0.7835446492 -2.91070941 -3.9370397002
H -1.1464214364 -0.9986212992 -3.0249668937
C -1.3970529086 -4.8425913448 -2.62904257
H -2.1941278453 -4.4532934659 -0.676098118
C -0.8804391326 -4.293267966 -3.8010960545
H -0.3763327756 -2.477735236 -4.8453276412
H -1.4691005341 -5.9197031627 -2.5158889349
H -0.5504766692 -4.9430106594 -4.6058039719
C -1.9318726393 1.4659377147 1.9646512821
C -3.3124293973 1.5276357426 2.2043400622
C -1.0785670105 0.9950328009 2.9657176207
C -3.8238047604 1.1678225856 3.445546517
H -3.9884292294 1.8585412871 1.4200284909
C -1.6019014398 0.6036525584 4.1972561217
H -0.0087212556 0.9270174827 2.7973514471
C -2.9687282275 0.7009263693 4.4431997535
H -4.892014299 1.2311893976 3.6257520791
H -0.93373965 0.2303474762 4.9667369498
H -3.3697152019 0.4049816364 5.4074824593
C -1.6189836162 3.7525582274 0.188655787
C -2.3623852755 4.4770193385 1.1203624573
C -1.0017726401 4.4261371689 -0.8745916872
C -2.4967229394 5.8588284355 0.9840575762
H -2.8324696148 3.9837315311 1.9632836826
C -1.1298778034 5.802589545 -1.0004782023
H -0.4128770046 3.8744599416 -1.6014023639
C -1.8835679084 6.5222948172 -0.0726909693
H -3.0761193943 6.4129673332 1.7157587082
H -0.6437062939 6.3143392817 -1.8249609282
H -1.9861932934 7.5983747129 -0.1733236639
Pd -2.5847338715 0.6402615331 -1.166409839
Cl -3.2030080724 2.5798281957 -2.3809091124
Cl -4.2426829846 -0.5299899385 -2.4379370854

Energy= -4170.3858309
Zero-point correction= 0.752714 (Hartree/Particle)
Thermal correction to Gibbs Free Energy= 0.666454
Sum of electronic and zero-point Energies= -4169.633116
Sum of electronic and thermal Energies= -4169.583249
Sum of electronic and thermal Enthalpies= -4169.582305
Sum of electronic and thermal Free Energies= -4169.719377

a-metastable-[eq]-e

C 3.8532885128 -1.5936017621 0.3731658231
C 2.6604397736 -1.1046459576 0.765082173
C 2.3651464609 0.3597856912 0.8125937594
C 1.1345130845 0.7143375851 0.2692654892
O 0.4009022755 -0.2782408782 -0.3327386553
C 0.2608947472 -1.390639223 0.449973731
C 1.4177354218 -1.8783904191 1.065867813
C 4.9982287199 -0.6871878471 0.0883783757
C 6.1414084949 -0.8604634991 0.8464260309
S 6.2210599145 -2.0858876482 2.1240251916
C 5.0907649666 -3.4096740357 1.5152926263
C 3.118442947 1.3776301671 1.3965091007
C 2.6364875733 2.685756583 1.3958989258
C 1.3608489019 2.9869267543 0.9204854401
C -1.1000877744 -3.080994654 1.4169214958
C 0.0155762706 -3.5513220382 2.1004458027
C 1.2624296577 -2.941509533 1.9503579483
C 4.9962655431 0.2645211409 -0.9807119236
C 6.1216714907 1.1195949961 -1.1443585084
C 7.2453725426 0.9606630705 -0.289548681
C 7.2712570213 -0.0227494857 0.6606897706
C 3.9072785251 0.3987486253 -1.8850646858
C 3.92197492 1.3557346496 -2.8678090572
C 5.0280091911 2.2270119283 -3.0072323332
C 6.1059755304 2.1035348559 -2.1683782522
H 5.6944142031 -4.3022269403 1.3383158928
H 4.3941905197 -3.6111623161 2.3321225356
H 4.0799816754 1.1518716468 1.8453222475
H 3.2439275014 3.4780180747 1.8202560998
H 0.9898903924 4.001918366 1.0017174737
H -2.0451806586 -3.6039036905 1.5182608371
H -0.0845229315 -4.4085360306 2.757372911
H 2.1155238651 -3.3037281473 2.5140478814
H 8.1018781761 1.6158780978 -0.4207409899
H 8.1516796753 -0.1627419764 1.2807411207
H 3.0580036271 -0.2701342005 -1.7963208983
H 3.0799272734 1.4417695835 -3.5481968607
H 5.0234776028 2.9831817882 -3.7861049707
H 6.9693043898 2.7546298946 -2.2762982779
C 0.5584533328 1.9764394743 0.3821938352
C -1.0090998833 -1.9348318968 0.612153414
C 3.3152092768 -4.1291868497 -0.1347059714
H 2.7101169765 -4.4727411815 0.7042553782

H 2.6431712675 -3.7808333415 -0.9246255437
H 3.8661881461 -4.9931600241 -0.5184360652
C 4.3195180884 -3.0380044079 0.2426323097
H 5.0489092061 -3.0116312234 -0.5742950342
P -2.4450923855 -1.2226439797 -0.2777680453
P -1.2586082237 2.0016216156 0.1915122114
C -3.8061239418 -0.8729580428 0.8712109585
C -4.8103661666 -0.0281760115 0.3800567137
C -3.9556200914 -1.4617007579 2.1284013963
C -5.9616683457 0.2008096351 1.1257769785
H -4.6893357743 0.4512944113 -0.5882993308
C -5.1052719072 -1.220282803 2.8753717457
H -3.1797191479 -2.093087315 2.5441604157
C -6.1120600454 -0.3996542351 2.3736816449
H -6.7354472248 0.8532788368 0.7340732327
H -5.2092601631 -1.6735171011 3.8560262811
H -7.0077884075 -0.2190050485 2.9596452333
C -3.0239682072 -2.6896579272 -1.1946005851
C -4.3808416804 -3.0013476434 -1.2993513245
C -2.0769676824 -3.5057036872 -1.8254353992
C -4.7850745585 -4.1202344461 -2.0252158475
H -5.129687709 -2.3822959591 -0.8166983266
C -2.4850360014 -4.6207007959 -2.546911332
H -1.0184998464 -3.2720718548 -1.7561499038
C -3.8410169202 -4.9301948237 -2.6488019741
H -5.8418010954 -4.3578021154 -2.0968985133
H -1.7432494206 -5.2483498012 -3.030648605
H -4.1588059165 -5.802716277 -3.2112196135
C -1.8018766376 1.5720859835 1.8869325703
C -3.0671064926 2.0055527867 2.3063835056
C -1.0099610175 0.8328991429 2.7702904719
C -3.5203250531 1.7171961344 3.5872391715
H -3.7004011942 2.5765204703 1.635201705
C -1.4744456272 0.5335222203 4.0493416478
H -0.0170884141 0.5004068217 2.4906607148
C -2.7252625895 0.9791123247 4.4625320019
H -4.5004449902 2.063555752 3.8985379308
H -0.8452769605 -0.0383325643 4.723976228
H -3.0820301329 0.7521669959 5.4623273961
C -1.872805087 3.7082675959 0.0539679258
C -1.5789595563 4.6414206135 1.0588585918
C -2.7506562736 4.0636696525 -0.9737153425
C -2.1099585915 5.9246237028 0.9987261186
H -0.9621673331 4.3655191081 1.9078170244

C -3.2937739942 5.3451342397 -1.0202863052
H -3.0117399329 3.3416772202 -1.7389268665
C -2.9648687016 6.2798501989 -0.0432158789
H -1.8678519233 6.6418670636 1.7763524612
H -3.9761701644 5.6084980243 -1.8221248551
H -3.3855384335 7.2798161846 -0.083363924
Pd -1.8582767533 0.6225877042 -1.5881632641
Cl -0.7549377694 2.2779964034 -2.9403332946
Cl -2.5408768887 -0.5143086284 -3.5690180715

Energy= -4170.3883928
Zero-point correction= 0.752363 (Hartree/Particle)
Thermal correction to Gibbs Free Energy= 0.666463
Sum of electronic and zero-point Energies= -4169.636030
Sum of electronic and thermal Energies= -4169.586121
Sum of electronic and thermal Enthalpies= -4169.585177
Sum of electronic and thermal Free Energies= -4169.721930
